# Prevalence and mortality of congenital heart disease in Korean adults

**DOI:** 10.1097/MD.0000000000011348

**Published:** 2018-07-06

**Authors:** Shin Yi Jang, Su Ra Seo, Ju Ryoung Moon, Eun Jeong Cho, EunKyoung Kim, Sung-A Chang, Jinyoung Song, June Huh, I-Seok Kang, Duk-Kyung Kim, Seung Woo Park

**Affiliations:** a Division of Cardiology, Department of Medicine, Heart Vascular Stroke Institute, Samsung Medical Center, Sungkyunkwan University School of Medicine, Seoul; b The National Health Insurance Service, Wonju; c Department of Pediatrics, Heart Vascular Stroke Institute, Samsung Medical Center, Sungkyunkwan University School of Medicine, Seoul; d Division of Cardiology, Department of Internal Medicine, Cardiology Clinic, National Cancer Center, Gyeonggi-do, Republic of Korea.

**Keywords:** adults, congenital heart disease, mortality, prevalence, survival rates

## Abstract

The aim of this study was to assess the age-standardized prevalence (prevalence) and age-standardized mortality (mortality) of congenital heart disease (CHD) by sex in Korean adults. Data were collected from the National Health Insurance Service in Korea from 2006 through 2016. The data consisted of main and secondary diagnoses related to CHD. We calculated the prevalence and mortality of CHD in adults with the direct method using the estimated Korean population in 2015 as the reference. The prevalence of CHD in Korean adults increased from 35.8 cases per 100,000 persons in 2006 to 65.6 cases in 2015. In 2015, the prevalence in the 20- to 44-year-old group, 45- to 64-year-old group, and the older than 65 years group was 54.6, 69.6, and 95.1 cases, respectively. Among women, the prevalence was 34.3 and 31.3 cases in men. The mortality of CHD in adults decreased from 3.061 persons per 100,000 persons in 2007 to 0.551 persons in 2015. The 5-year survival rate (SR) for people with CHD was 0.92 (95% confidence interval [CI] 0.91–0.93). The 5-year SR for the 20- to 44- and 45- to 64-year-old groups was more than 0.95; however, the 5-year SR of the older than 65 years group was only 0.73 (95% CI 0.72–0.74). The prevalence of CHD in Korean adults increased and the related mortality decreased during a decade. The 5-year SR of CHD for those over 65 years was lower than that for younger age groups.

## Abstract 2

Supplemental Digital Content is available in the text

## 1 Introduction

The population of adults with congenital heart disease (CHD) has dramatically increased during the past few decades in developed Western countries^[1,2]^ because of early detection of CHD and increased survival of neonate and infant patients made possible by rapid progress in pediatric cardiology, cardiac surgery, anesthesia, and intensive care.^[3]^ CHD surgery in the young became more in Korea common in 1980s, and the number of adult patients with CHD thus is expected to have increased. Previous studies have shown the distribution of CHD in adult patients who were diagnosed when they were young.^[4,5]^ However, few studies have reported the overall distribution of adult patients with CHD, including patients who were not diagnosis in childhood, and few studies have evaluated the prevalence and mortality of CHD using adult national health data. Therefore, our aim in this study was to assess the overall prevalence of CHD in Korean adults, considering sex differences, using Korean National Health Insurance Service data from 2006 to 2015. We also show mortality and survival rates (SRs) for patients newly diagnosed with CHD from 2007 to 2015.

## 2 Methods

### 2.1 Study population

We collected data from Korean National Health Insurance^[6]^ benefit records from 2006 to 2015, excluding records for medical aid. Data in the National Health Insurance benefit records represent each patient's first diagnosis in that year. CHD diagnoses were extracted from the records after considering the data given for the primary and secondary diagnoses, which depended on the complaint and symptoms. The National Health Insurance benefit records do not contain information confirming diagnoses or describing treatments at medical institutes. Therefore, the final diagnoses could differ from the diagnoses in the data. In this study, we used death data for Koreans from 2007 to 2016.

### 2.2 Diagnosis

As shown in Table 1, the data contain primary and secondary diagnoses related to CHD according to the 10th revision of the International Statistical Classification of Diseases and Related Health Problems (ICD 10). There are 2 types of CHD. Acyanotic CHD comprises

congenital ventricular and/or atrial septal defects, including ventricular septal defect (VSD) (Q21.0, Q21.00, Q21.01, Q21.08, Q21.09), atrial septal defect (ASD) (Q21.1, Q21.10, Q21.11, 21.18, Q21.19), atrioventricular septal defects (Q21.2), and congenital malformation of the cardiac septum, unspecified (Q21.9);
patent ductus arteriosus (Q25.0);
pulmonary artery stenosis (Q22.1, Q25.6);
coarctation of aorta (Q25.1);
pulmonary venous connection (Q26.2, Q26.3, Q26.4);
congenital tricuspid stenosis (Q22.4, Q22.8, Q22.9);
congenital stenosis of aortic valve (Q23.0);
congenital insufficiency of aortic valve (Q23.1);
congenital mitral stenosis (Q23.2, Q23.3);
malformation of coronary vessels (Q24.5, Q24.8, Q24.9); and
stenosis or malformation of aorta (Q24.4, Q25.2, Q25.3, Q25.4, Q25.8, Q25.9).

**Table 1:**
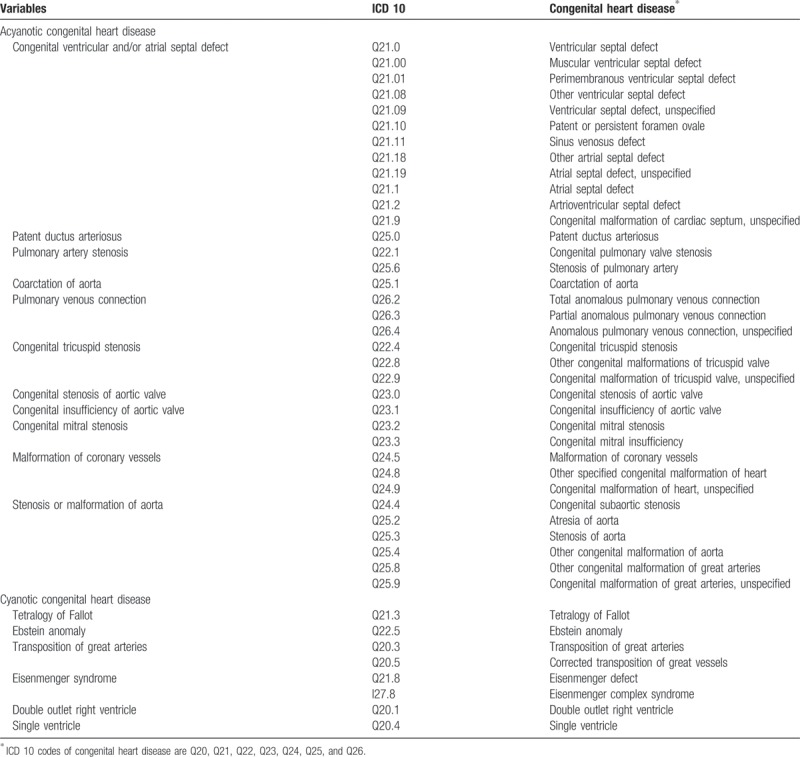
Composition of acyanotic and cyanotic congenital heart disease.

Cyanotic CHD comprises Tetralogy of Fallot (Q21.3), Ebstein anomaly (Q22.5), transposition of great arteries (Q20.3, Q20.5), Eisenmenger syndrome (Q21.81, I21.81), double outlet right ventricle (Q20.1), and single ventricle (Q20.4). The reason for classifying the 2 types of CHD, acyanotic, and cyanotic is that symptoms of acyanotic CHD can range from asymptomatic to severe, whereas the symptoms of cyanotic CHD can be mostly seen as soon as baby is born or during infancy. Because ICD 10 code Q21.0 (VSD or ASD) was subdivided in 2011, we ran additional analyses for the age-standardized prevalence and mortality of VSD (Q21.00, Q21.01, Q21.08, Q21.09) and ASD (Q21.11, Q21.10, Q21.18, Q21.19, Q21.2) from 2011 to 2015.

### 2.3 Statistical methods

We calculated the age-standardized prevalence of CHD in adults with the direct method^[7]^ using the beneficiaries of health insurance from the Korean National Health Insurance Statistical Yearbook from 2006 to 2015 as the patients and the estimated Korean population in 2015 as the reference.^[8]^ We washed out the first year (2006) for newly detected cases to see the age-standardized mortality of newly diagnosed CHD from 2007 to 2015. The Kaplan–Meier method was also used to compare survival among patients with CHD by age group and sex using log-rank tests.

### 2.4 Ethics

This study protocol was exempted by the Institutional Review Board of Samsung Medical Center (IRB File No: 2017-02-032).

## 3 Results

### 3.1 Prevalence of CHD in Korean adults

Table 2     shows the age-standardized prevalence of CHD in Korean adults, which was 35.8 cases in 2006 and 65.6 cases in 2015. The age-standardized prevalence for the 20- to 44-year-old group, 45- to 64-year-old group, and the older than 65 years group increased from 28.3 cases, 31.4 cases, and 45.5 cases, respectively, in 2006 to 54.6 cases, 69.6 cases, and 95.1 cases in 2015. The age-standardized prevalence in women increased from 21.5 cases in 2006 to 34.3 cases in 2015; in men, it rose from 14.2 cases in 2006 to 31.3 cases in 2015 (Table 2     and Supplementary [Supp.] Table 1). The age-standardized prevalence of congenital ventricular and/or atrial septal defects in Korean adults increased from 21.2 cases in 2006 to 31.7 cases in 2015. The age-standardized prevalence for the 20- to 44-year-old group, 45- to 64-year-old group, and the older than 65 years group was 25.7 cases, 34.8 cases, 44.7 cases, respectively, in 2015. The age-standardized prevalence was 17.9 cases for women and 13.7 cases for men in 2015 (Table 2     and Supp. Table 1-1). We also showed the age-standardized prevalence of VSD (Supp. Table 1-1-1) and ASD (Supp. Table 1-1-2) in Korean adults from 2011 to 2015. The age-standardized prevalence of patent ductus arteries in Korean adults increased from 1.94 cases in 2006 to 2.68 cases in 2015 (Table 2     and Supp. Table 1–2). The age-standardized prevalence of pulmonary artery stenosis in Korean adults increased from 0.41 cases in 2006 to 0.74 cases in 2015 (Table 2     and Supp. Table 1–3). The age-standardized prevalence of Coarctation of aorta in Korean adults increased from 0.27 cases in 2006 to 0.58 cases in 2015 (Table 2     and Supp. Table 1–4). The age-standardized prevalence of pulmonary venous connection in Korean adults increased from 0.05 cases in 2006 to 0.16 cases in 2015 (Table 2     and Supp. Table 1–5). The age-standardized prevalence of congenital tricuspid stenosis in Korean adults was 0.25 cases in 2006 and 0.21 cases in 2015 (Table 2     and Supp. Table 1–6). The age-standardized prevalence of congenital stenosis of aortic valve in Korean adults was 0.34 cases in 2006 and 0.50 cases in 2015 (Table 2     and Supp. Table 1–7). The age-standardized prevalence of congenital insufficiency of aortic valve in Korean adults increased from 0.91 cases in 2006 to 6.51 cases in 2015 (Table 2     and Supp. Table 1–8). The age-standardized prevalence of congenital mitral stenosis in Korean adults was 0.63 cases in 2006 and 0.46 cases in 2015 (Table 2     and Supp. Table 1–9). The age-standardized prevalence of malformation of coronary vessels in Korean adults increased from 2.58 cases in 2006 to 9.07 cases in 2015 (Table 2     and Supp. Table 1–10). The age-standardized prevalence of stenosis or malformation of aorta in Korean adults increased from 0.96 cases in 2006 to 1.95 cases in 2015 (Table 2     and Supp. Table 1–11). The age-standardized prevalence of Tetralogy of Fallot in Korean adults increased from 1.32 cases in 2006 to 3.12 cases in 2015 (Table 2     and Supp. Table 1–12). The age-standardized prevalence of Ebstein anomaly in Korean adults increased from 0.60 cases in 2006 to 1.04 cases in 2015 (Table 2     and Supp. Table 1–13). The age-standardized prevalence of transposition of great arteries in Korean adults increased from 0.38 cases in 2006 to 0.83 cases in 2015 (Table 2     and Supp. Table 1–14). The age-standardized prevalence of Eisenmenger syndrome in Korean adults increased from 1.38 cases in 2006 to 1.81 cases in 2015; the 2015 age-standardized prevalence for the 20- to 44-year-old group, 45- to 64-year-old group, and the older than 65 years group was 1.49 cases, 1.75 cases, and 3.05 cases, respectively, and was 1.11 cases for women and 0.70 cases for men (Table 2     and Supp. Table 1–15). In additional analysis, in 2015, the age-standardized prevalence of primary pulmonary hypertension (main diagnoses; ICD 10 code: I27.0 and I27.2) in Korean adults was 4.64 persons; for the 20- to 44-year-old group, 45- to 64-year-old group, and the older than 65 years group, it was 2.33 persons, 5.06 persons, and 14.9 persons, respectively, and was 3.24 persons for women and 1.39 persons for men (Supp. Table 1-15-1). The age-standardized prevalence of double outlet right ventricle in Korean adults increased from 0.19 cases in 2006 to 0.48 cases in 2015 (Table 2     and Supp. Table 1–16). The age-standardized prevalence of single ventricle in Korean adults increased from 0.14 cases in 2006 to 0.65 cases in 2015 (Table 2     and Supp. Table 1–17).

**Table 2:**
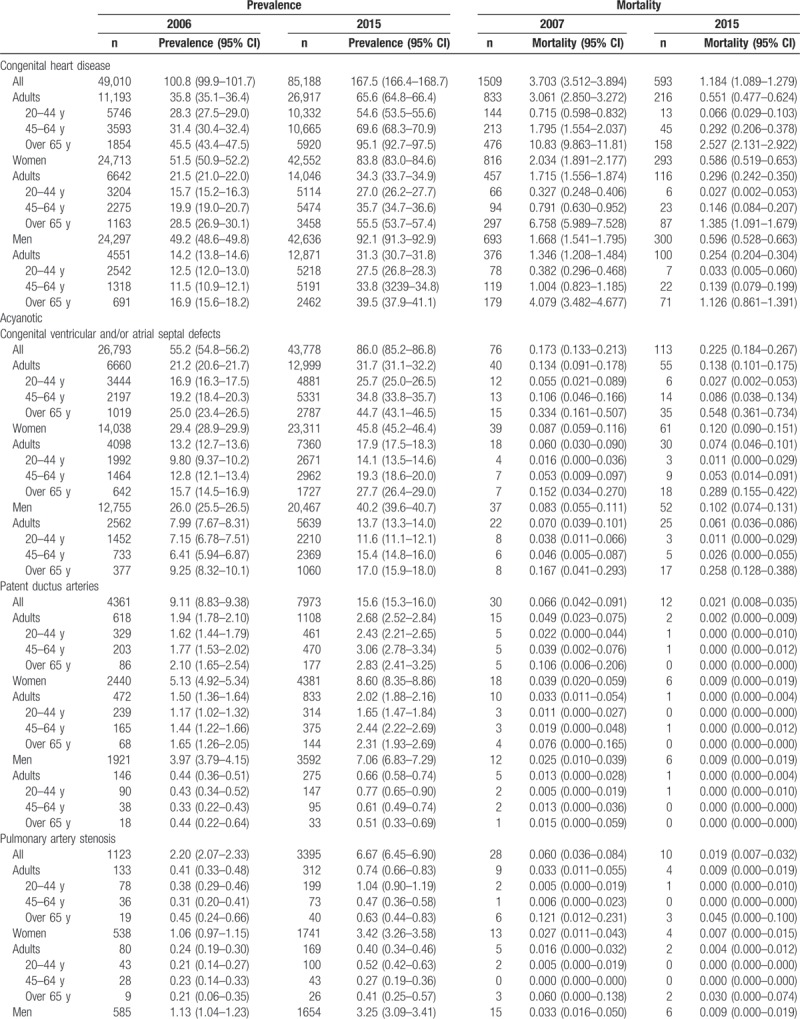
Age-standardized prevalence and mortality of congenital heart disease in adults between 2006 and 2015 (per 100,000).

**Table 2 (Continued):**
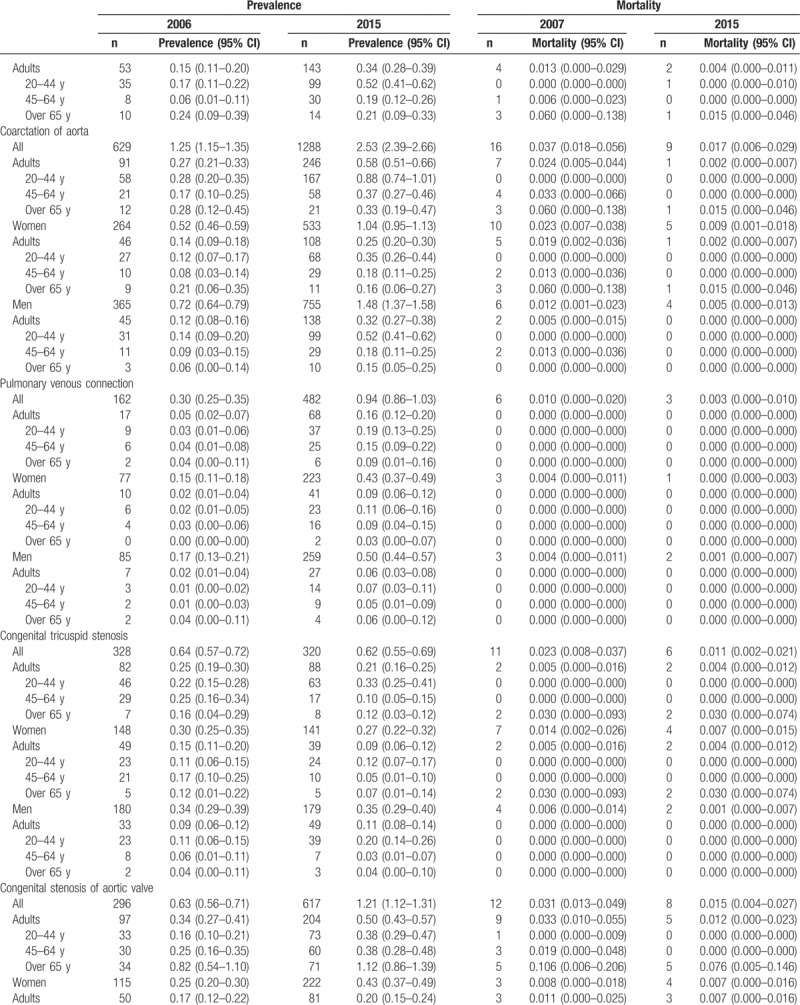
Age-standardized prevalence and mortality of congenital heart disease in adults between 2006 and 2015 (per 100,000).

**Table 2 (Continued):**
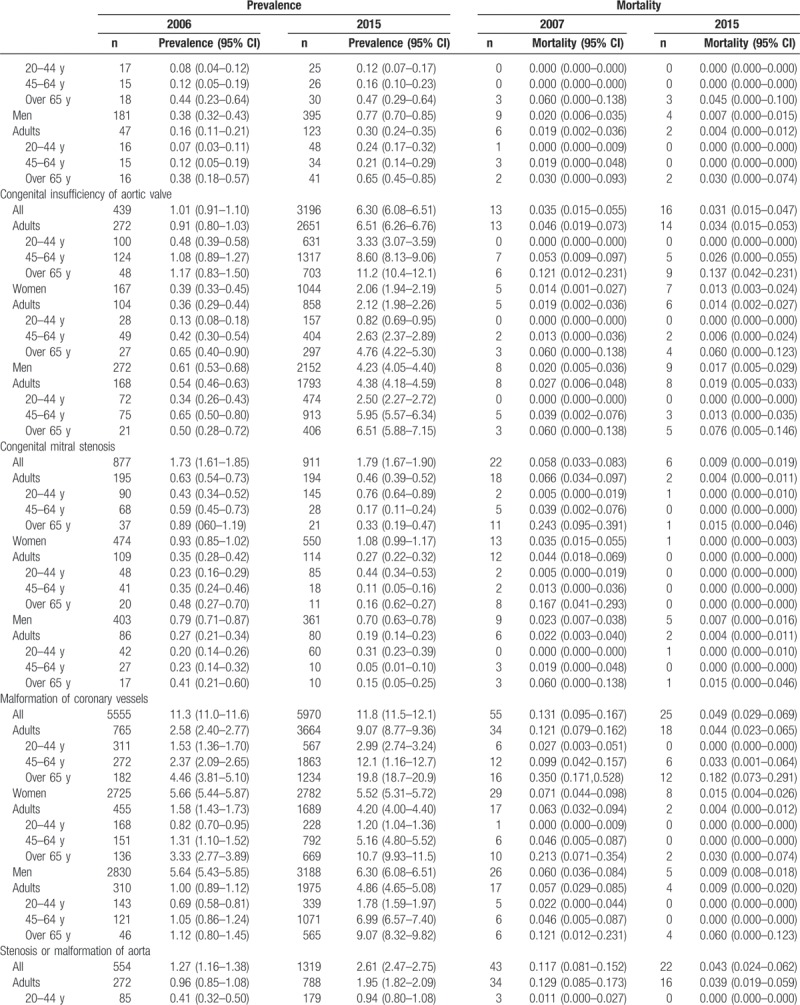
Age-standardized prevalence and mortality of congenital heart disease in adults between 2006 and 2015 (per 100,000).

**Table 2 (Continued):**
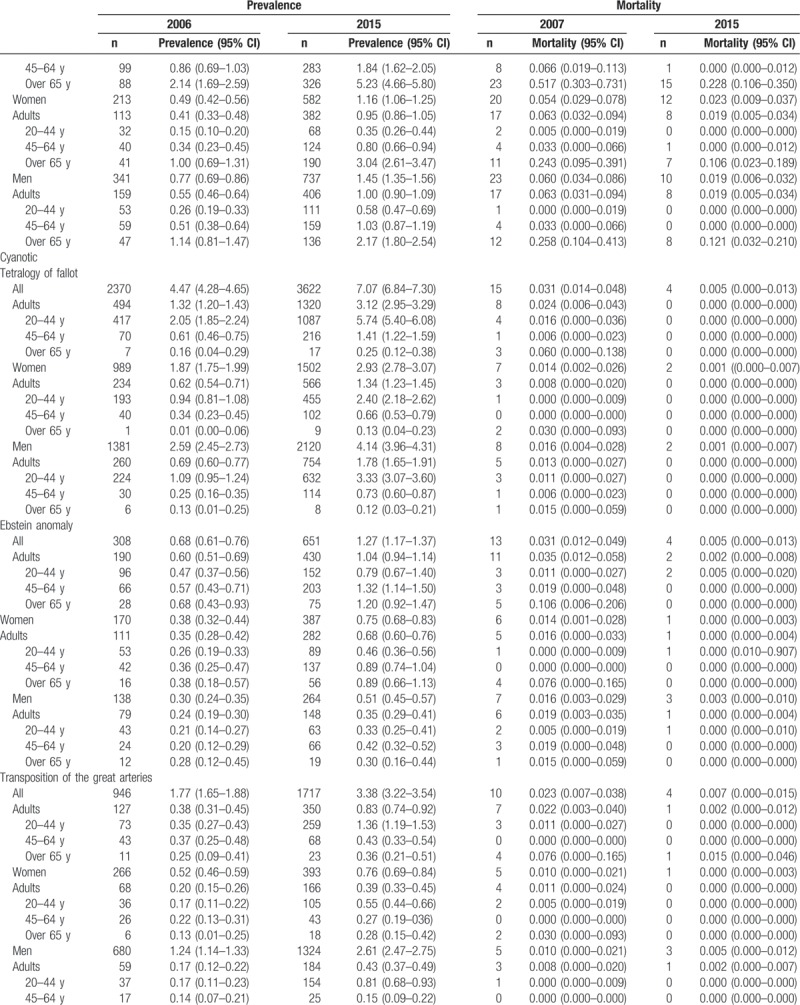
Age-standardized prevalence and mortality of congenital heart disease in adults between 2006 and 2015 (per 100,000).

**Table 2 (Continued):**
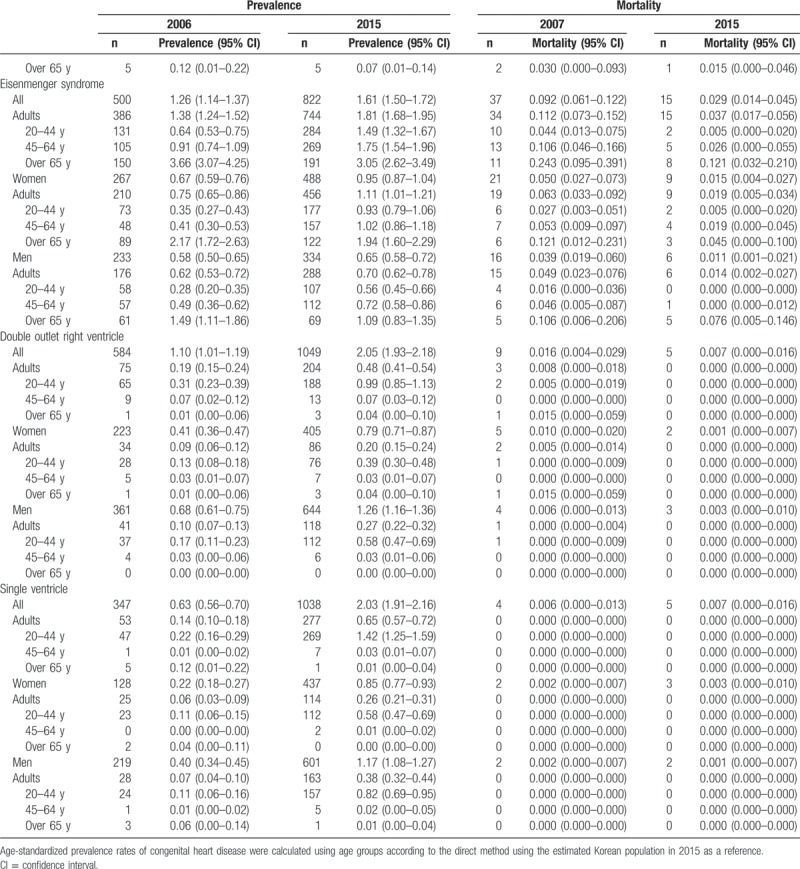
Age-standardized prevalence and mortality of congenital heart disease in adults between 2006 and 2015 (per 100,000).

### 3.2 Mortality and SR for patients newly diagnosed with CHD

Table 2     also shows the age-standardized mortality of adult CHD. The age-standardized mortality of CHD in Korean adults was 3.061 persons in 2007 and 0.551 persons in 2015. The age-standardized mortality for the 20- to 44-year-old group, 45- to 64-year-old group, and the older than 65 years group was 0.066, 0.292, and 2.527 persons, respectively, in 2015. The age-standardized mortality was 0.296 persons for women and 0.245 persons for men in 2015 (Table 2     and Supp. Table 2, 2-1 to 2-17).

Figure 1   and Supp. Table 3 show the cumulative SR for adults with CHD and specific diseases from 2007 through 2016. The 5-year SR for CHD was 0.91 (95% confidence interval [CI] 0.90–0.92). The 5-year SR for the 20- to 44-year-old group, 45- to 64-year-old group, and the older than 65 years group was 0.98 (95% CI 0.97–0.99), 0.95 (95% CI 0.94–0.95), and 0.73 (95% CI 0.72–0.74) (*P*<.001), respectively. The 5-year SR by sex was 0.91 (95% CI 0.90–0.92) for men and 0.92 (95% CI 0.91–0.93) for women (*P*<.001) (Fig. 1  A). The 5-year SR for Korean adults with congenital ventricular and/or atrial septal defect was 0.93 (95% CI 0.92–0.94). The 5-year SR for the 20- to 44-year-old group, 45- to 64-year-old group, and the older than 65 years group was 0.98 (95% CI 0.97–0.99), 0.95 (95% CI 0.94–0.96), and 0.75 (95% CI 0.74–0.77) (*P*<.001), respectively. The 5-year SR by sex was 0.93 (95% CI 0.92–0.94) for men and 0.94 (95% CI 0.93–0.95) (*P*<.001) for women (Fig. 1  B). In additional analyses, we considered VSD and ASD from 2011 to 2016 (6 years). The 5-year SR for Korean adults with VSD was 0.94 (95% CI 0.93–0.95) (Fig. 1  C). The 5-year SR for Korean adults with ASD was 0.93 (95% CI 0.92–0.94) (Fig. 1  D). The 5-year SR for Korean adults with patent ductus arteriosus was 0.95 (95% CI 0.94–0.96) (Fig. 1  E). The 5-year SR for Korean adults with pulmonary artery stenosis was 0.92 (95% CI 0.87–0.92) (Fig. 1  F). The 5-year SR for coarctation of aorta in Korean adults was 0.91 (95% CI 0.87–0.93) (Fig. 1  G). The 5-year SR for Korean adults with congenital stenosis of aortic valve was 0.75 (95% CI 0.71–0.79) (Fig. 1  H). The 5-year SR for Korean adults with congenital insufficiency of aortic valve was 0.93 (95% CI 0.92–0.94) (Fig. 1  I). The 5-year SR for Korean adults with congenital mitral was 0.85 (95% CI 0.81–0.89) (Fig. 1  J). The 5-year SR for Korean adults with malformation of coronary vessels was 0.94 (95% CI 0.93–0.94) (Fig. 1  K). The 5-year SR for Korean adults with stenosis or malformation of aorta was 0.79 (95% CI 0.77–0.80) (Fig. 1  L). The 5-year SR for Korean adults with Tetralogy of Fallot was 0.96 (95% CI 0.95–0.97) (Fig. 1  M). The 5-year SR for Korean adults with the Ebstein anomaly was 0.92 (95% CI 0.89–0.94) (Fig. 1  N). The 5-year SR for Korean adults with transposition of great arteries was 0.85 (95% CI 0.80–0.89) (Fig. 1  O). The 5-year SR for Korean adults with Eisenmenger syndrome was 0.61 (95% CI 0.58–0.63); in the 20- to 44-year-old group, 45- to 64-year-old group, and the older than 65 years group, it was 0.84 (95% CI 0.80–0.87), 0.69 (95% CI 0.64–0.73), and 0.46 (95% CI 0.42–0.79) (*P*<.001), respectively, and it was 0.56 (95% CI 0.52–0.60) for men and 0.64 (95% CI 0.61–0.67) for women (*P *< .001) (Fig. 1  P).

**Figure 1:**
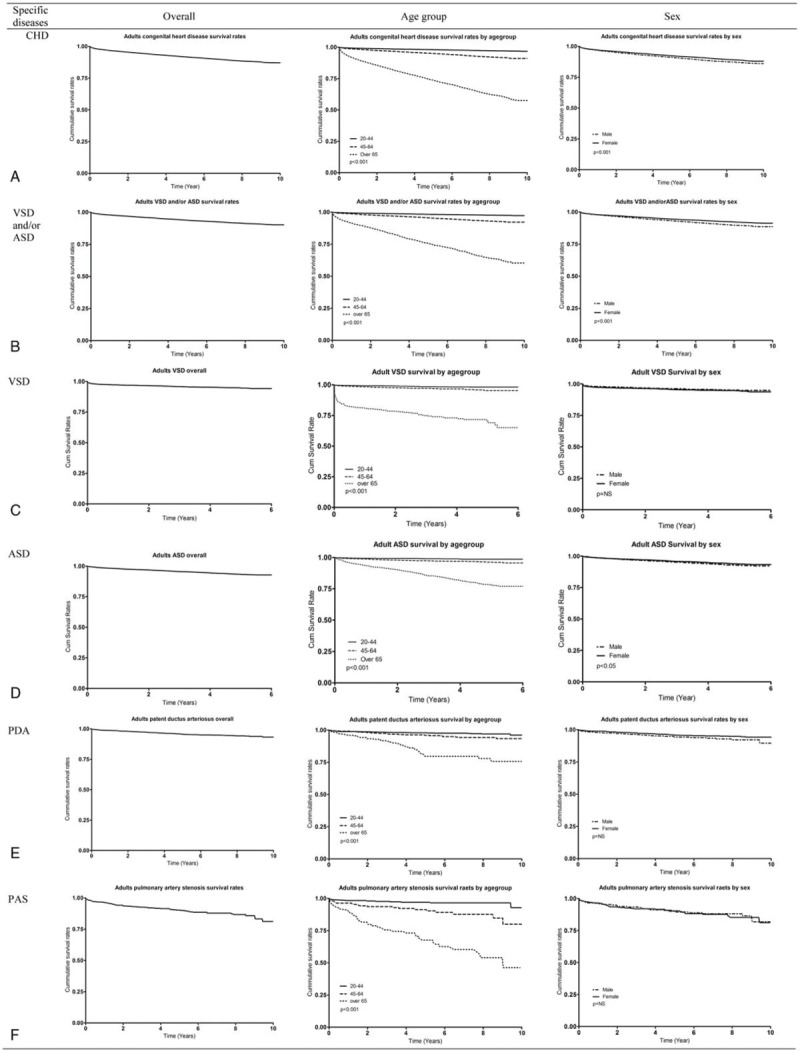
Survival curve of congenital heart disease in Korean adults. AR = congenital insufficiency of aortic valve, AS = congenital stenosis of aortic valve, CHD = congenital heart disease, CoA = coarctation of aorta, MS = congenital mitral stenosis, PAS = pulmonary artery stenosis, PDA = patent ductus arteriosus, TGV = transposition of great vessels, TOF = Tetralogy of Fallot, VSD and/or ASD = ventricular septal defect and/or atrial septal defect.

**Figure 1 (Continued):**
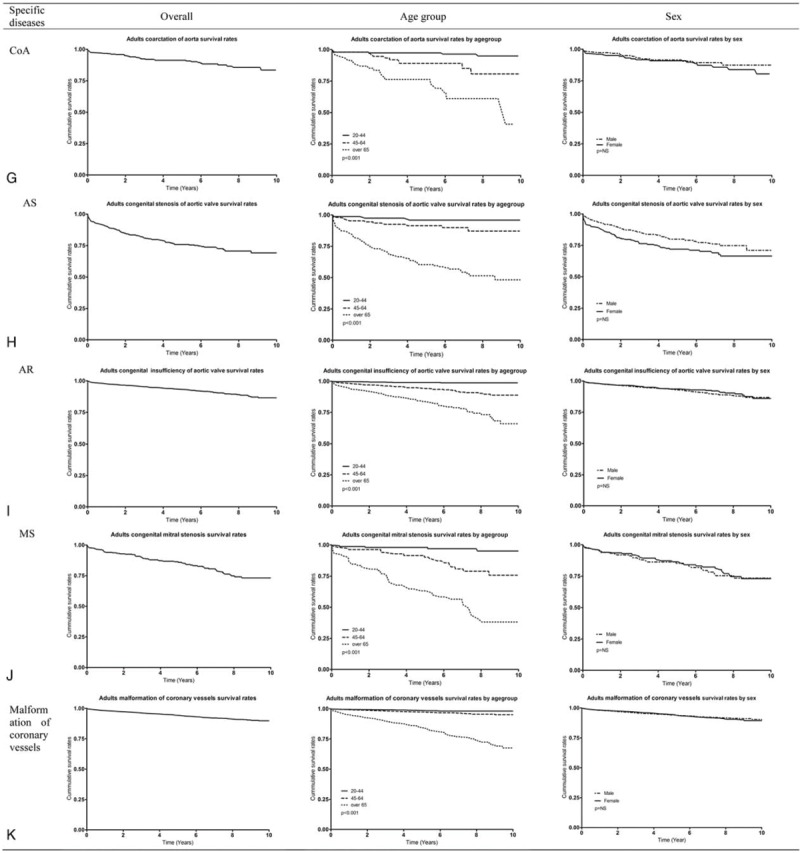
Survival curve of congenital heart disease in Korean adults. AR = congenital insufficiency of aortic valve, AS = congenital stenosis of aortic valve, CHD = congenital heart disease, CoA = coarctation of aorta, MS = congenital mitral stenosis, PAS = pulmonary artery stenosis, PDA = patent ductus arteriosus, TGV = transposition of great vessels, TOF = Tetralogy of Fallot, VSD and/or ASD = ventricular septal defect and/or atrial septal defect.

**Figure 1 (Continued):**
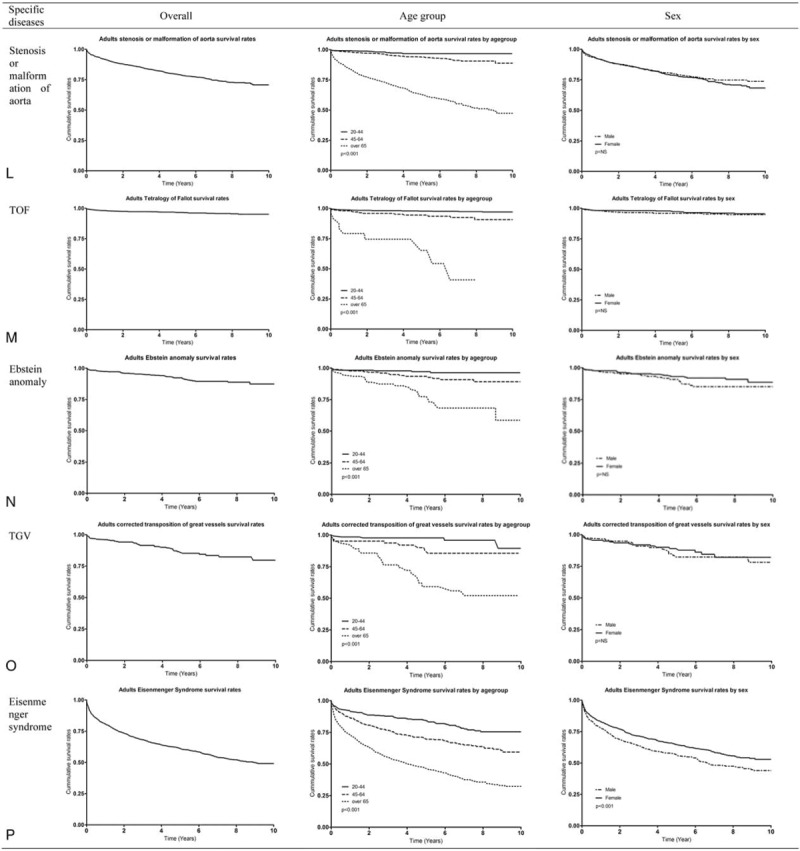
Survival curve of congenital heart disease in Korean adults. AR = congenital insufficiency of aortic valve, AS = congenital stenosis of aortic valve, CHD = congenital heart disease, CoA = coarctation of aorta, MS = congenital mitral stenosis, PAS = pulmonary artery stenosis, PDA = patent ductus arteriosus, TGV = transposition of great vessels, TOF = Tetralogy of Fallot, VSD and/or ASD = ventricular septal defect and/or atrial septal defect.

## 4 Discussion

Our findings show that the overall age-standardized prevalence of CHD in adults increased from 35.8 cases per 100,000 persons in 2006 to 65.6 cases in 2015. Our data include main and secondary diagnoses of CHD based on signs and/or symptoms in the hospital. Therefore, it is difficult to compare our results with previous data. Nevertheless, the overall tendency in the prevalence of CHD in adults is similar to that found in previous studies.^[9–11]^ The prevalence of CHD in adults increased from 17,911 persons in 1985 to 23,536 persons in 2000 in a Quebec study using ICD 9,^[12,13]^ and about 1 million American adult patients with treated or untreated CHD were reported from 1940 to 2002 in a study of CHD patients in the United States (US) who survived childhood.^[4]^ We partially attribute the increasing prevalence of CHD in Korean adults to the increasing role of echocardiography in popular health-screening examinations. There were 21,301 echocardiography devices used to assess the structure and function of the heart available at a total of 62,853 hospitals or clinics registered with the National Health Insurance Service as of mid-2013 in Korea (Supp. Table 4).^[14,15]^ In addition, we had concerns about the accuracy of the data recorded. Therefore, we checked the distribution of both CHD and acquired heart disease by heart surgery status using data from the Korean Heart Foundation from 2006 to 2015.^[16]^ The Korean Heart Foundation has kept statistics since 2001 for interventions in pediatric, medicine, and surgery departments that involve chest surgery for cardiovascular disease, using surveys completed by pediatricians, cardiologists, and thoracic surgeons in 73 hospitals in Korea. The number of persons undergoing CHD surgery increased from 4546 in 2006 to 5723 in 2015. This score included interventions in pediatric departments (971 in 2006 and 1891 in 2015), departments of medicine (71 in 2006 and 483 in 2015), and departments of chest surgery (3504 in 2006 and 3349 in 2015). Further, to determine the accuracy of the data we used in this study,^[15]^ we considered the classification of 298 mutually independent disease categories from the Korean National Health Insurance Service yearbook from 2006 to 2013 (Supp. Fig. 2), including all congenital malformations of the cardio- and neurovascular systems (ICD 10: Q20.0–Q28.9).^[17]^ Whereas the data we used from the National Health Insurance benefit records represent first diagnoses, the classification of the 298 disease categories represents main diagnoses. Congenital malformations of the cardio- and neurovascular systems from the classification of the 298 diseases also showed an increasing trend in adult CHD, with a pattern similar to that demonstrated in our results (Supp. Fig. 2). We also showed CHD data (ICD 10: Q20.0–Q26.9) from a tertiary hospital from 2006 to 2013 (Supp. Fig. 3).

The most frequent type of CHD in adults in this study was ventricular and/or atrial septal defects. Our results correspond well with those from other countries, such as a study of US prevalence of congenital cardiovascular defects distribution conducted in 2002,^[2]^ a nationwide hospital trend study in the US from 2003 to 2012,^[18]^ a CHD study in Quebec, Canada,^[12]^ a hospital-based study of CHD in adults in Thailand,^[19]^ an adult CHD study in Japan,^[11]^ and a Nigerian CHD study.^[20]^ Although the birth prevalence of specific congenital heart defects varies by ethnicity,^[21]^ the prevalence of congenital ventricular and/or atrial septal defects in adults is the highest in all ethnicities.

We found that both overall and among adults, the prevalence of congenital ventricular and/or atrial septal defects, patent ductus arteriosus, the Ebstein anomaly, and Eisenmenger syndrome was higher in females than in males. The age-standardized prevalence of Eisenmeger syndrome revealed a higher distribution in the 65 years and over group. The age-standardized prevalence of primary pulmonary hypertension, which is similar in clinical appearance to Eisenmenger syndrome, also showed a similar distribution to Eisenmenger syndrome in this study. Interestingly, the prevalence of congenital ventricular and/or atrial septal defects in this study greatly increased from 2006 to 2015. We attribute this change to the subdivision of congenital malformations of the cardiac septa (Q21.0) in ICD 10 in 2011. Therefore, we used additional analyses to show that the age-standardized prevalence of both VSD and ASD increased from 2011 to 2015 (Supp. Table 1-1-1, 1-1-2). In children, our finding of a higher prevalence of congenital ventricular and/or atrial septal defects in females was in close agreement with a hospital-based echocardiographic study of CHD in newborns in China.^[22]^ In adults, results for the prevalence of congenital ventricular and/or atrial septal defects are similar to those from a large cohort of adult patients born with the secundum ASD in Europe. That study also found that the prevalence of ASD was higher in women than in men.^[23]^

We showed that the age-standardized mortality of CHD in Korean adults decreased from about 3 persons per 100,000 in 2007 to about 0.5 persons in 2015. Overall, age-standardized mortality decreased from 3.7 persons in 2007 to 1.2 persons in 2015. The mortality patterns of the present study correspond well with those of an earlier study. The overall age-standardized mortality of CHD in the US decreased from 1.37 in 1999 to 1.04 in 2006.^[24]^

The SR across a decade for Korean adults with CHD was about 92%. To compare CHD SR, we calculated the percentage of deaths at each age divided by the total number of deaths per year from 2006 to 2015 using Korean Census data and drew the survival curve (Supp. Fig. 4). In this survival curve, the survival for the age groups from 0 to 4 years to 65 to 69 years was more than 92%, and the rate for the age groups from 70 to 74 years to 80 to 84 years was around 80%. Therefore, Korean adults with CHD had higher SR than the general Korean population, probably because patients with CHD receive regular health care. Interestingly, the 5-year SR for patients older than 65 years who have pulmonary artery stenosis, congenital stenosis of aortic valve, congenital mitral stenosis, stenosis or malformation of aorta, Tetralogy of Fallot, or transposition of great arteries was 0.59 to 0.67. The 5-year SR for Eisenmenger syndrome in those older than 65 years was lower than those among younger people. The 5-year SR for men with Eisenmenger syndrome was significantly lower than those for women. Overall SRs, including both children and adults, for CHD and specific CHD are provided in Supp. Figure 1 and Supp. Table 3.

Our results could have important clinical and public health implications. The increasing prevalence of CHD in adults primarily applied to acyanotic CHD, especially congenital ventricular and/or atrial septal defects, and varied by sex. The age-standardized prevalence in women was higher than that in men; on the contrary, age-standardized mortality was higher in men than in women in our study. However, it is difficult to explain why the prevalence and mortality associated with CHD in adults varies by sex. Therefore, appropriate health resources should be allocated to improve diagnoses, treatments, research, and health policies that affect CHD in adults, with particular attention to gender, as the number of adults with CHD is expected to continue to increase. In addition to considering gender perspectives, CHD health policies should also consider socioeconomic position because of potential associated with disease, poverty, unemployment, or old age.

### 4.1 Study limitations

Our study has several limitations. First, the data included only CHD. We used the main and secondary diagnoses based on signs and symptoms, which could differ from the final diagnosis. Therefore, the prevalence of CHD in this study might be under- or overestimated. Second, the National Health Insurance benefit records might have missed adult patients with CHD who did not use medical services, paid for their own medical expenses, or had Medical Aid.^[15]^ Third, we could not evaluate preexisting complex diseases, the perils of CHD, or corrected transposition of the great arteries because of data limitations. Fourth, we could not determine the cause of death because Korean National Health Insurance benefit data do not include that information. Therefore, because of the increasing prevalence of CHD in adults, Korea needs a well-designed hospital-based CHD registry.

## 5 Conclusion

We found that the age-standardized prevalence of CHD in Korean adults was 65.6 cases per 100,000 persons in 2015, age-standardized mortality was 0.5 persons per 100,000 persons in 2015, and SR across a decade exceeded 90%. Those over the age of 65 and men showed lower SR than younger age groups and women, respectively. Those patterns in prevalence and mortality should be considered in future research designs and policies for cardiovascular health care services, with particular consideration of sex differences.

## Author contributions

**Conceptualization:** Shin Yi Jang, Su Ra Seo, Ju Ryoung Moon, Eun Jeong Cho, EunKyoung Kim, Seung Woo Park.

**Data curation:** Shin Yi Jang, Duk-Kyung Kim.

**Formal analysis:** Shin Yi Jang, Su Ra Seo.

**Investigation:** Shin Yi Jang.

**Methodology:** Shin Yi Jang.

**Supervision:** I-Seok Kang, Duk-Kyung Kim, Seung Woo Park.

**Validation:** Shin Yi Jang, EunKyoung Kim, Sung-A Chang, Jinyoung Song, June Huh, I-Seok Kang, Duk-Kyung Kim, Seung Woo Park.

**Visualization:** Shin Yi Jang, Eun Jeong Cho, EunKyoung Kim, Sung-A Chang, I-Seok Kang, Duk-Kyung Kim, Seung Woo Park.

**Writing – original draft:** Shin Yi Jang, Eun Jeong Cho, EunKyoung Kim, Seung Woo Park.

**Writing – review & editing:** Shin Yi Jang, Seung Woo Park.

## Supplementary Material

Supplemental Digital Content
