# Supplementary material for: Prevalence and mortality of congenital heart disease in Korean adults
Source: Medicine (Baltimore). 2018 Jul 6;97(27):e11348. doi: 10.1097/MD.0000000000011348 (PMC6076156; doi:10.1097/MD.0000000000011348)
Supplement: Supplemental Digital Content [file medi-97-e11348-s001.pdf]

Supplementary Figure 1. Survival curve of congenital heart disease in Korea

<table>
<tr><th>Specific diseases</th><th>Overall</th><th>Age group</th><th>Sex</th></tr>
<tr><td>A. CHD</td><td>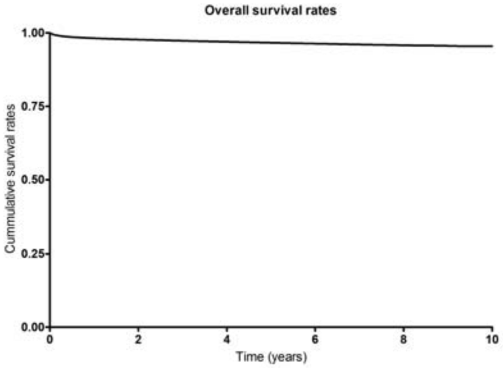
</td><td>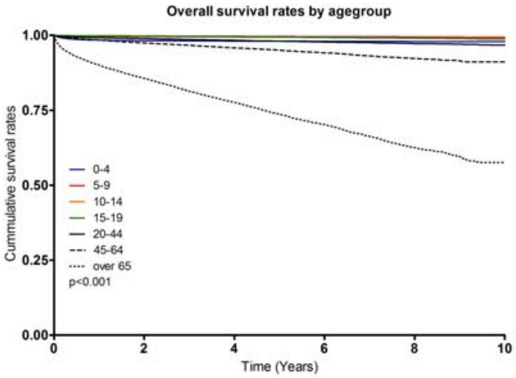
</td><td>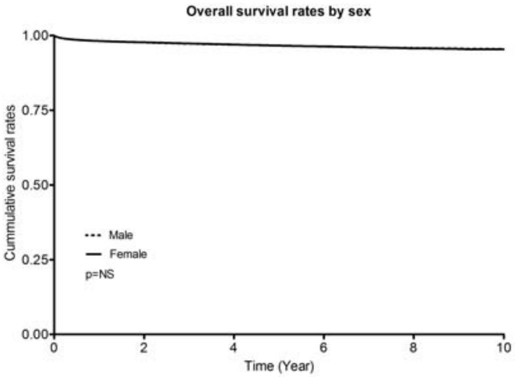
</td></tr>
<tr><td>B. VSD and/or ASD</td><td>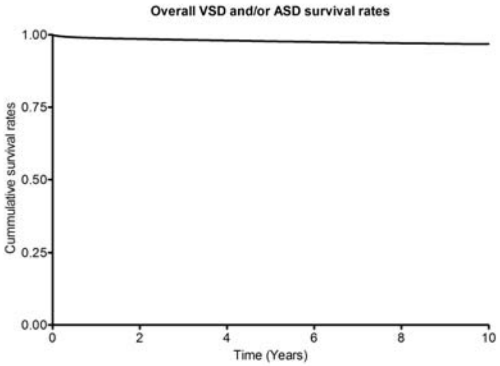
</td><td>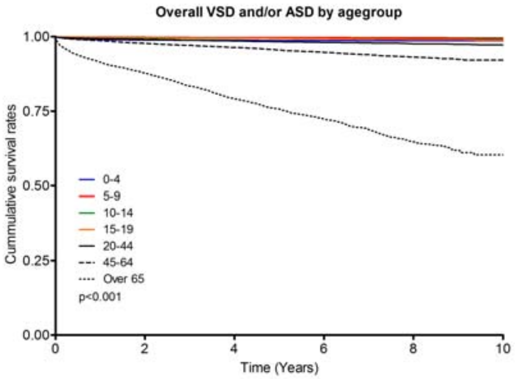
</td><td>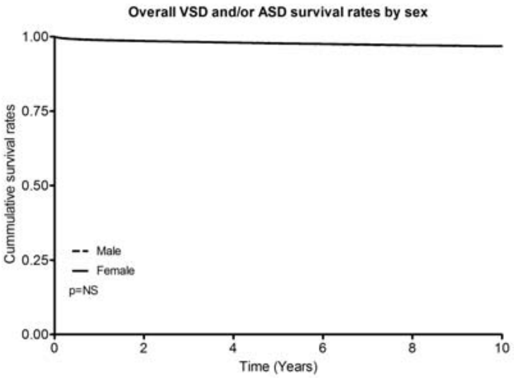
</td></tr>
</table>

C.
VSD

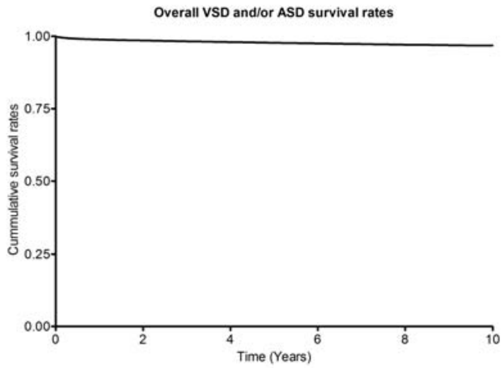
Overall VSD and/or ASD survival rates
Cummulative survival rates
Time (Years)

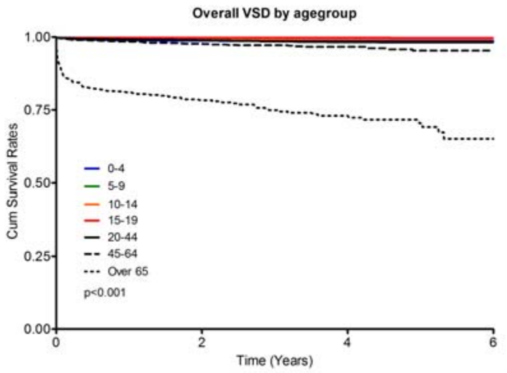
Overall VSD by agegroup
Cum Survival Rates
0-4
5-9
10-14
15-19
20-44
45-64
Over 65
p<0.001
Time (Years)

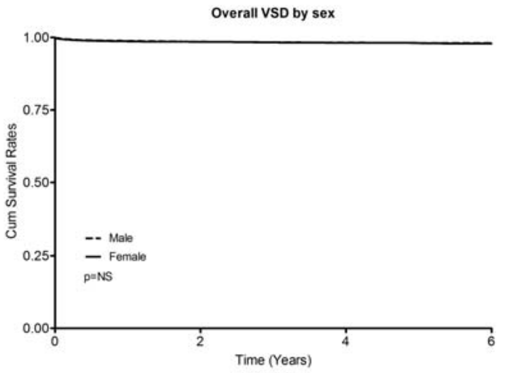
Overall VSD by sex
Cum Survival Rates
Male
Female
p=NS
Time (Years)

D.
ASD

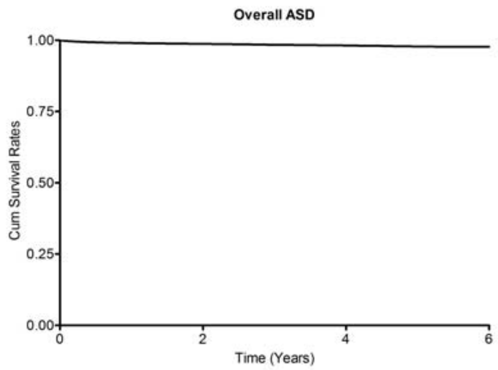
Overall ASD
Cum Survival Rates
Time (Years)

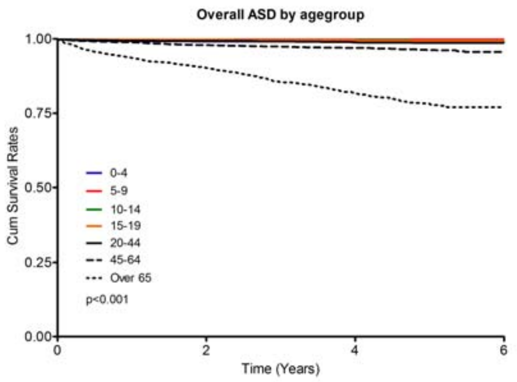
Overall ASD by agegroup
Cum Survival Rates
0-4
5-9
10-14
15-19
20-44
45-64
Over 65
p<0.001
Time (Years)

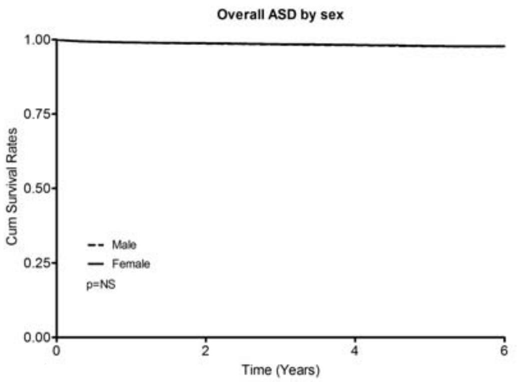
Overall ASD by sex
Cum Survival Rates
Male
Female
p=NS
Time (Years)

E.
PDA

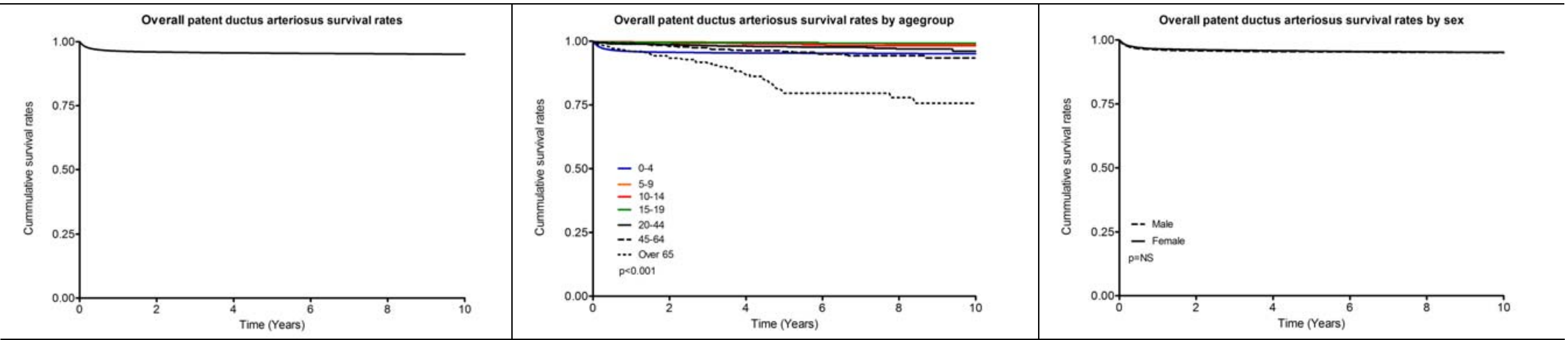

F.
PAS

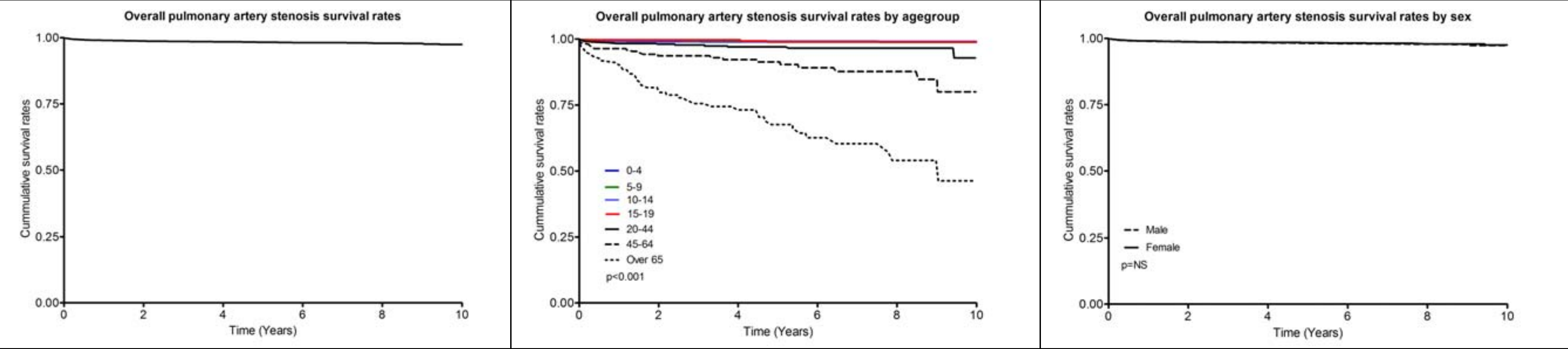

G.
CoA

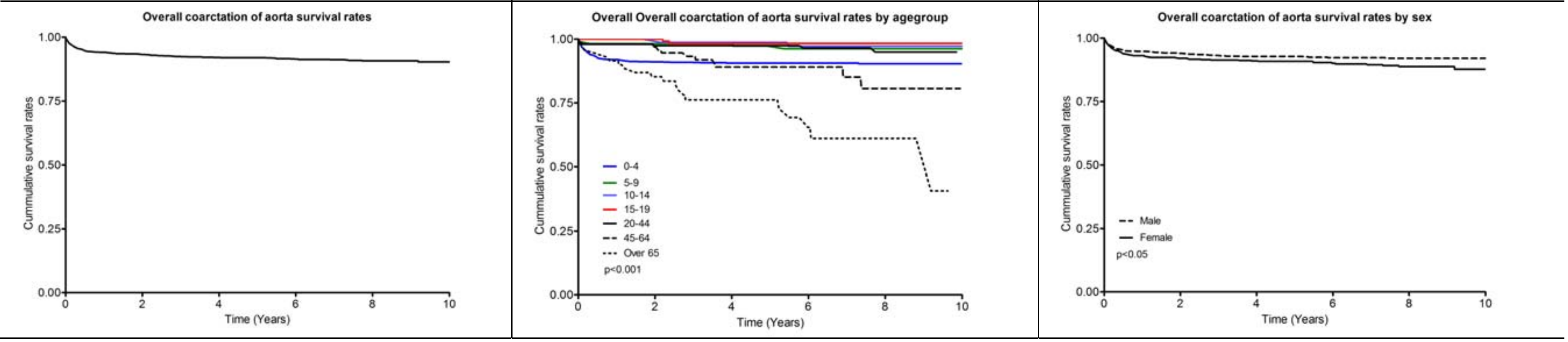

H. PVC

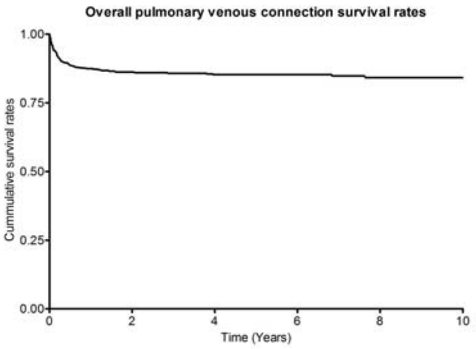
Overall pulmonary venous connection survival rates
Cummulative survival rates
1.00
0.75
0.50
0.25
0.00
0 2 4 6 8 10
Time (Years)

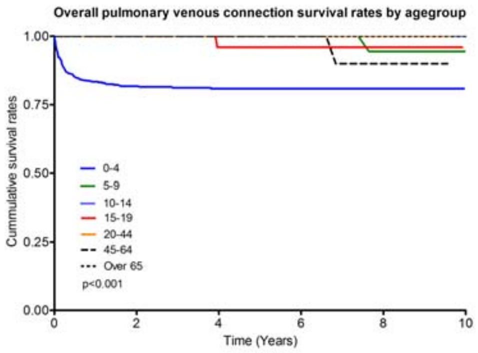
Overall pulmonary venous connection survival rates by agegroup
Cummulative survival rates
1.00
0.75
0.50
0.25
0.00
0 2 4 6 8 10
Time (Years)
0-4
5-9
10-14
15-19
20-44
45-64
Over 65
p<0.001

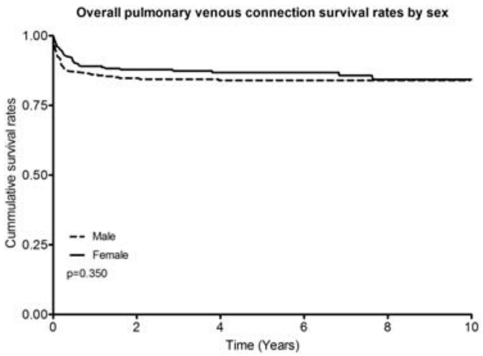
Overall pulmonary venous connection survival rates by sex
Cummulative survival rates
1.00
0.75
0.50
0.25
0.00
0 2 4 6 8 10
Time (Years)
Male
Female
p=0.350

I. TS

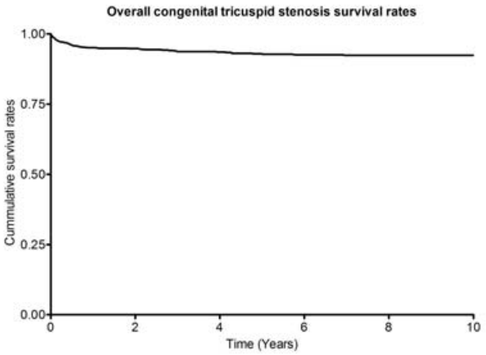
Overall congenital tricuspid stenosis survival rates
Cummulative survival rates
1.00
0.75
0.50
0.25
0.00
0 2 4 6 8 10
Time (Years)

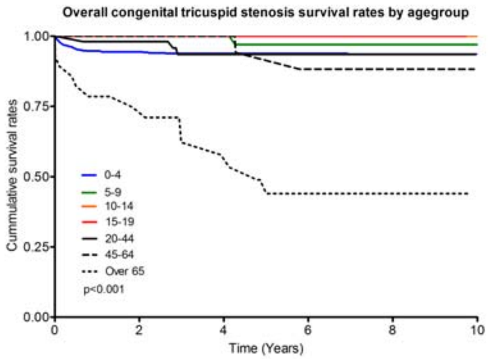
Overall congenital tricuspid stenosis survival rates by agegroup
Cummulative survival rates
1.00
0.75
0.50
0.25
0.00
0 2 4 6 8 10
Time (Years)
0-4
5-9
10-14
15-19
20-44
45-64
Over 65
p<0.001

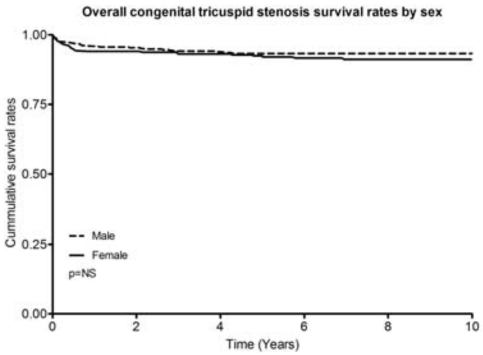
Overall congenital tricuspid stenosis survival rates by sex
Cummulative survival rates
1.00
0.75
0.50
0.25
0.00
0 2 4 6 8 10
Time (Years)
Male
Female
p=NS

J. AS

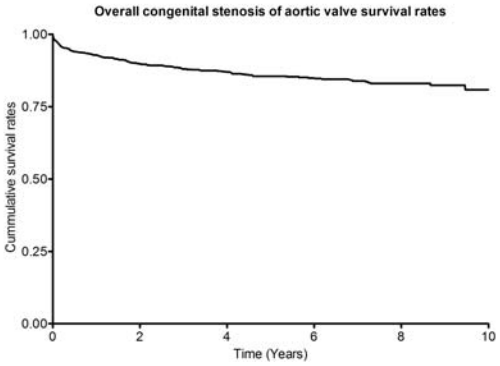
Overall congenital stenosis of aortic valve survival rates
Cummulative survival rates
1.00
0.75
0.50
0.25
0.00
0 2 4 6 8 10
Time (Years)

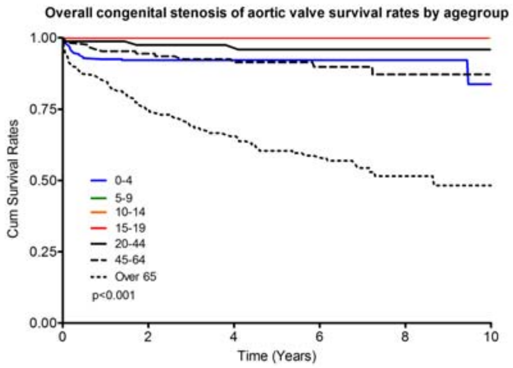
Overall congenital stenosis of aortic valve survival rates by agegroup
Cum Survival Rates
1.00
0.75
0.50
0.25
0.00
0 2 4 6 8 10
Time (Years)
0-4
5-9
10-14
15-19
20-44
45-64
Over 65
p<0.001

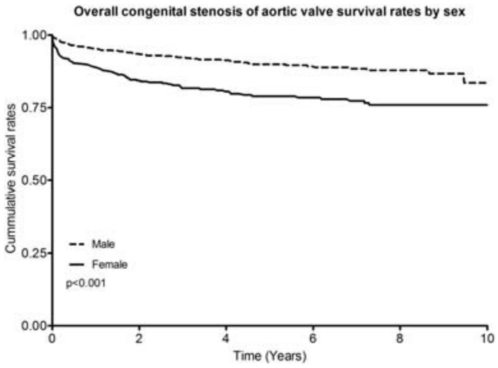
Overall congenital stenosis of aortic valve survival rates by sex
Cummulative survival rates
1.00
0.75
0.50
0.25
0.00
0 2 4 6 8 10
Time (Years)
Male
Female
p<0.001

K. AR

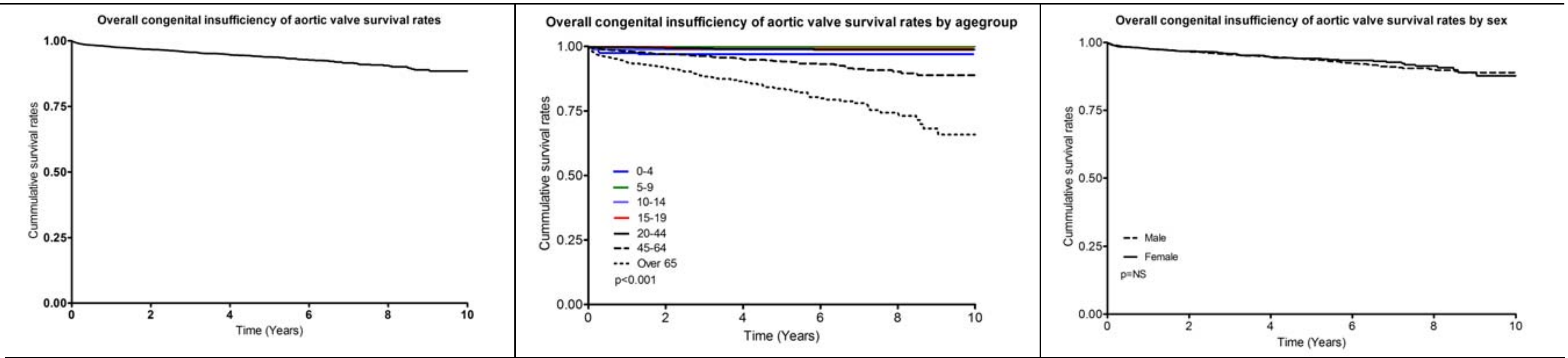
Overall congenital insufficiency of aortic valve survival rates
Cummulative survival rates
Time (Years)
Overall congenital insufficiency of aortic valve survival rates by agegroup
0-4
5-9
10-14
15-19
20-44
45-64
Over 65
p<0.001
Cummulative survival rates
Time (Years)
Overall congenital insufficiency of aortic valve survival rates by sex
Male
Female
p=NS
Cummulative survival rates
Time (Years)

L. MS

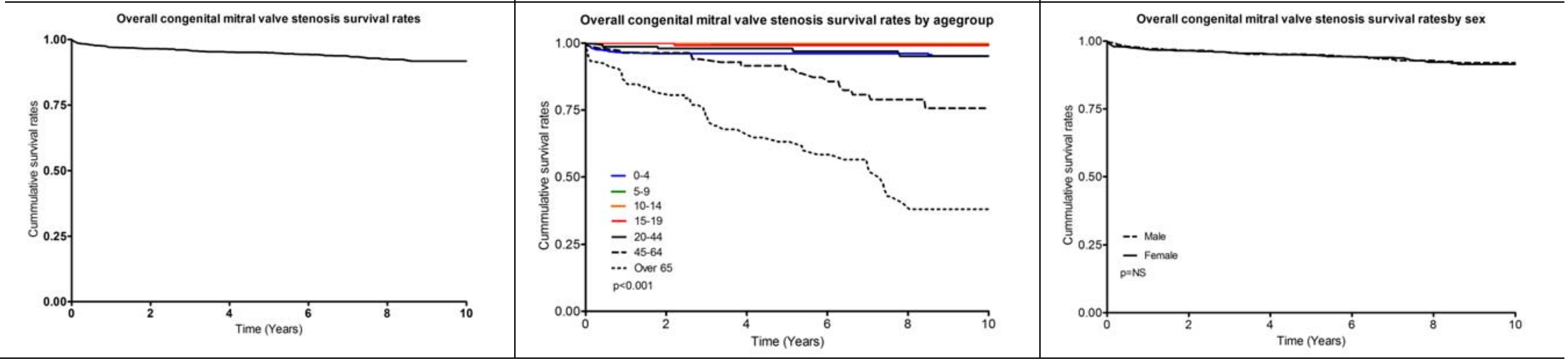
Overall congenital mitral valve stenosis survival rates
Cummulative survival rates
Time (Years)
Overall congenital mitral valve stenosis survival rates by agegroup
0-4
5-9
10-14
15-19
20-44
45-64
Over 65
p<0.001
Cummulative survival rates
Time (Years)
Overall congenital mitral valve stenosis survival ratesby sex
Male
Female
p=NS
Cummulative survival rates
Time (Years)

M. Malformation of coronary vessels

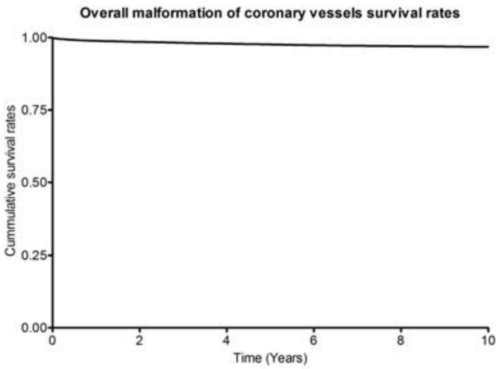

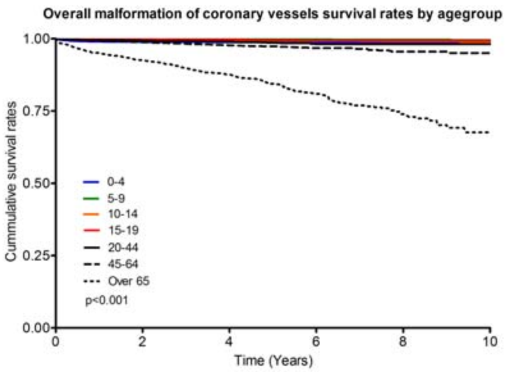

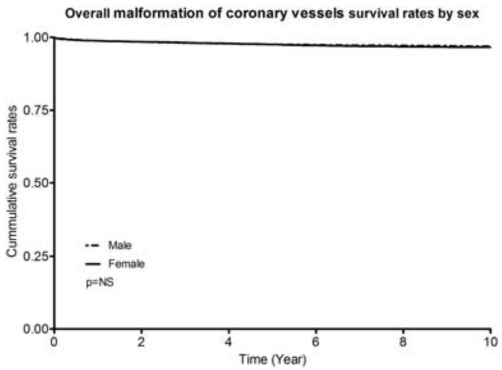

N. Stenosis or malformation of aorta

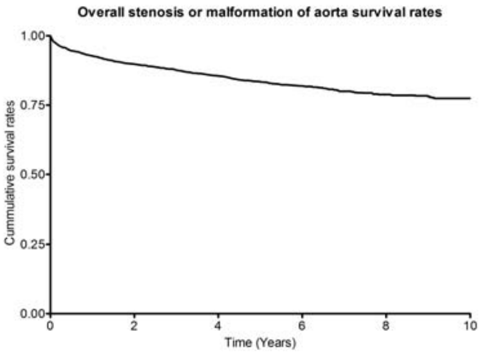

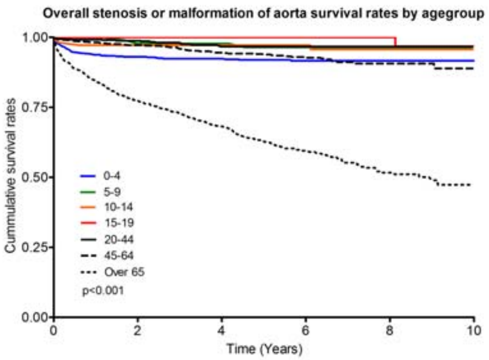

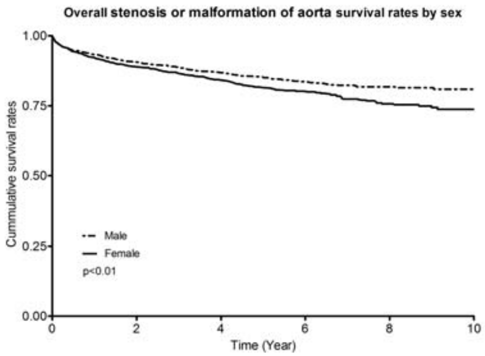

O. TOF

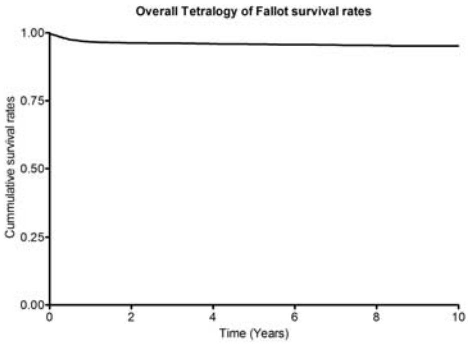

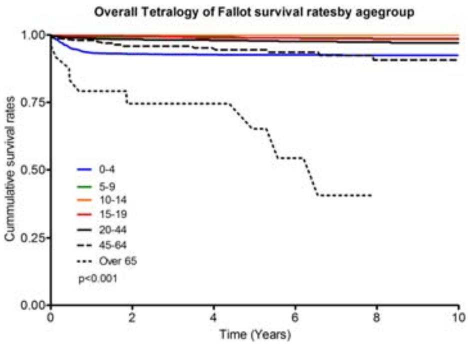

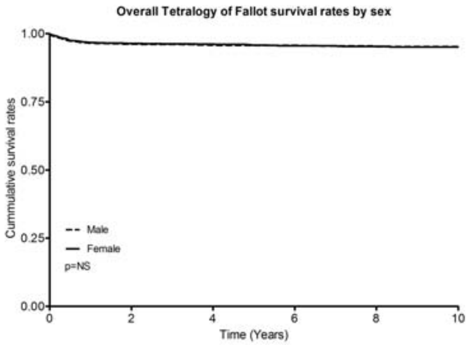

P.
Ebstein
anomaly

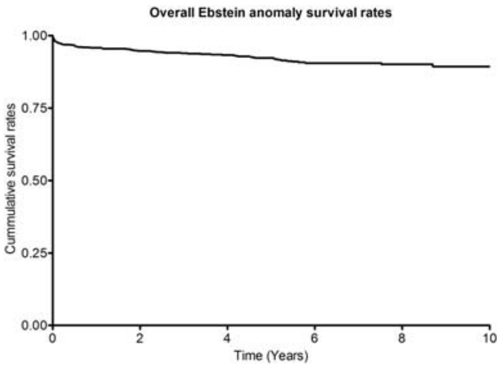

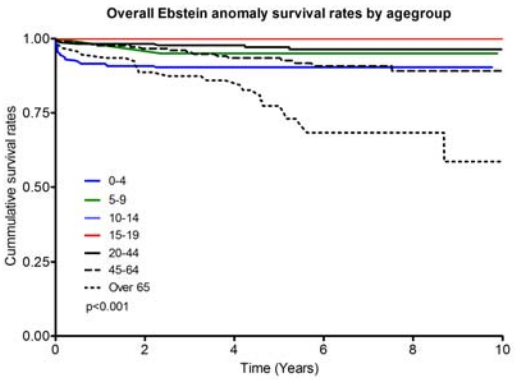

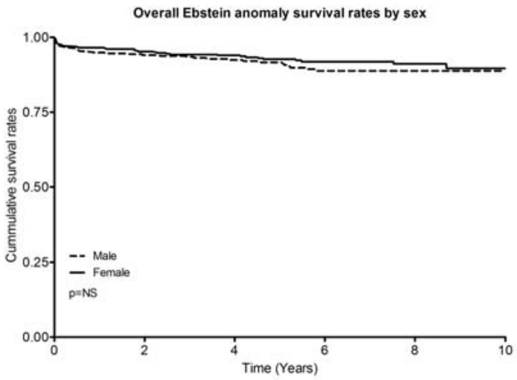

Q.
TGV

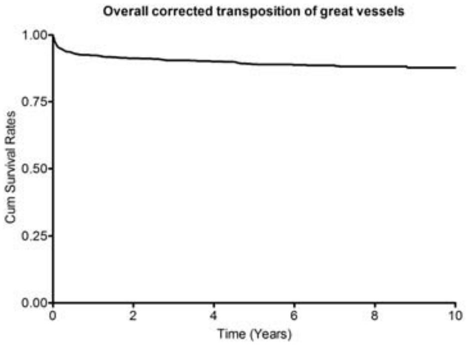

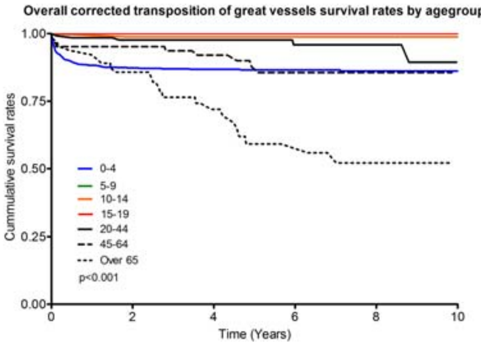

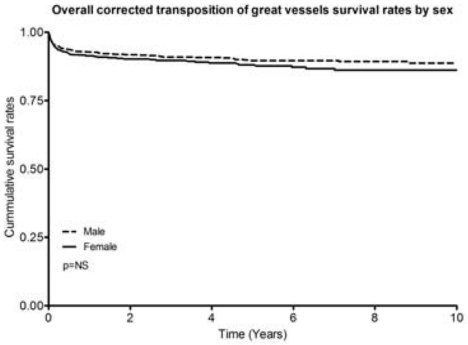

R.
Eisenmenger syndrome

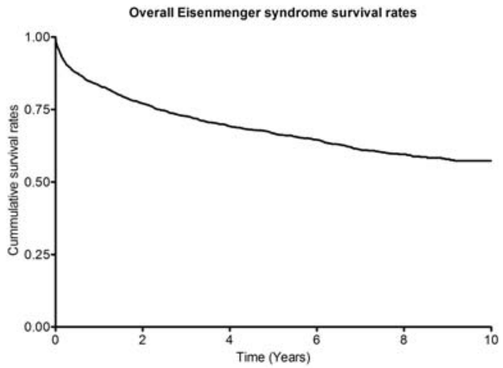

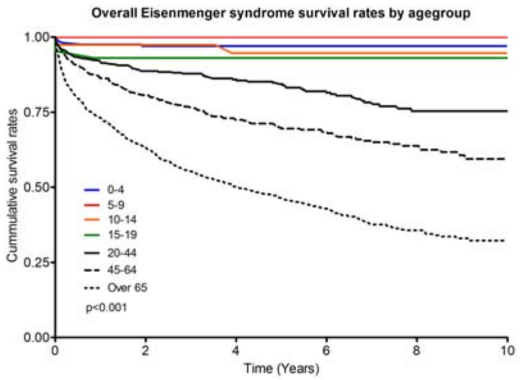

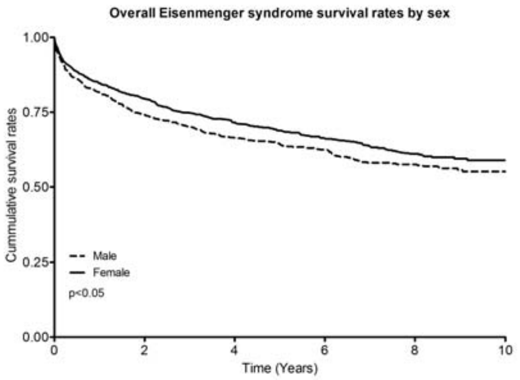

S.
Double outlet right ventricle

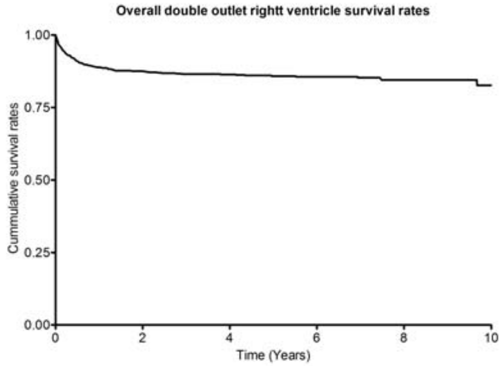

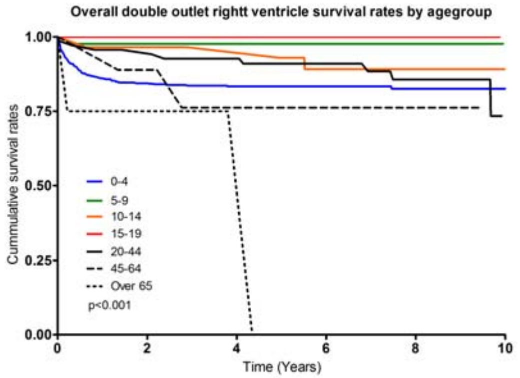

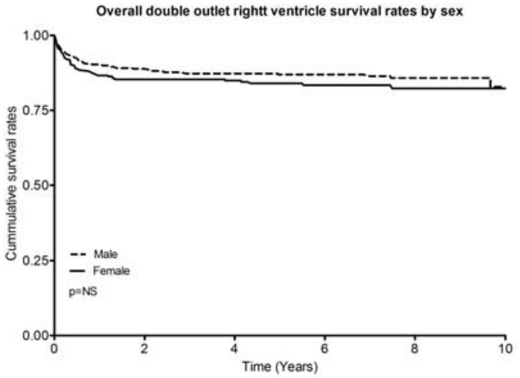

T. Single ventricle

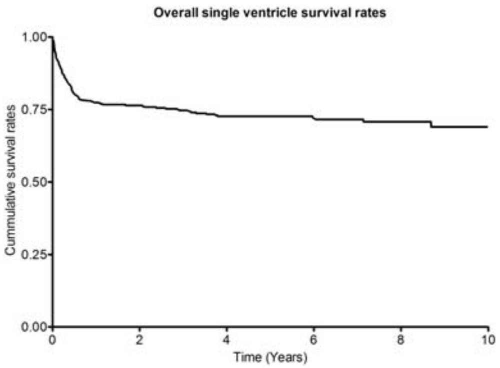

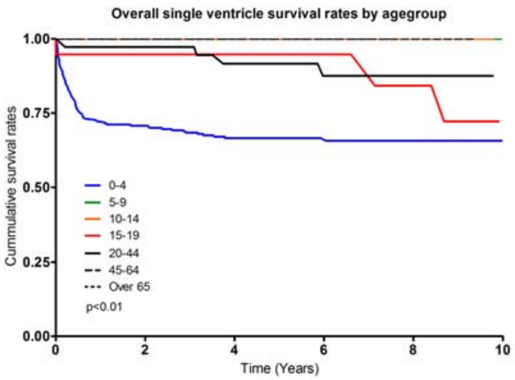

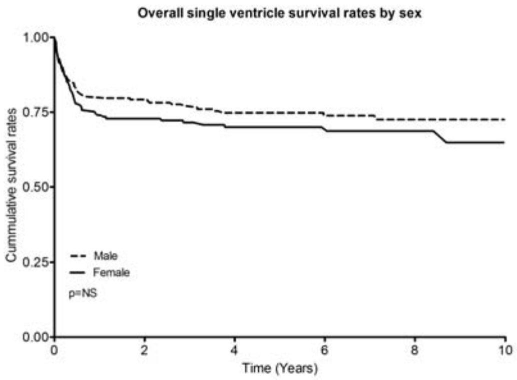

CHD congenital heart disease; VSD and/or ASD ventricular septal defect and/or atrial septal defect; PDA patent ductus arteriosus; PAS pulmonary artery stenosis; CoA Coarctation of aorta; PVC pulmonary venous connection; TS congenital tricuspid stenosis; AS congenital stenosis of aortic valve; AR congenital insufficiency of aortic valve; MS congenital mitral stenosis; TOF Tetralogy of Fallot; TGV Transposition of great vessels.

Supplementary Figure 2. Age-standardized prevalence[a] of congenital malformations of cardiovascular systems* overall (per 100,000) using 298 disease codes from the Korean National Health Insurance Service

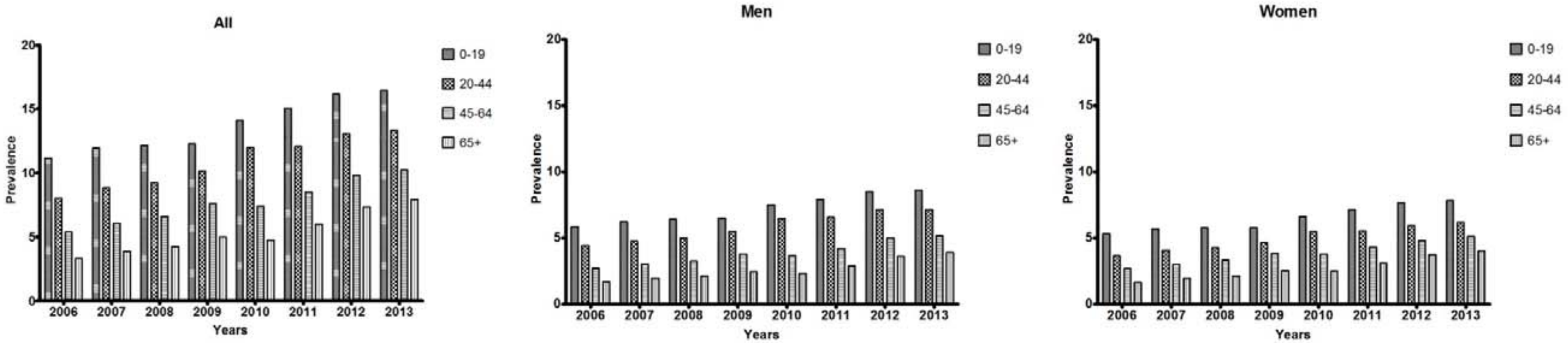

a Age-standardized prevalence rates of congenital malformations of cardiovascular systems (ICD 10: Q20.0–Q28.9) were calculated using age groups according to the

direct method using the estimated Korean population in 2010 as a reference

ac chambers and connections, congenital
aortic and mitral valves, other congenital
congenital malformations of the peripheral

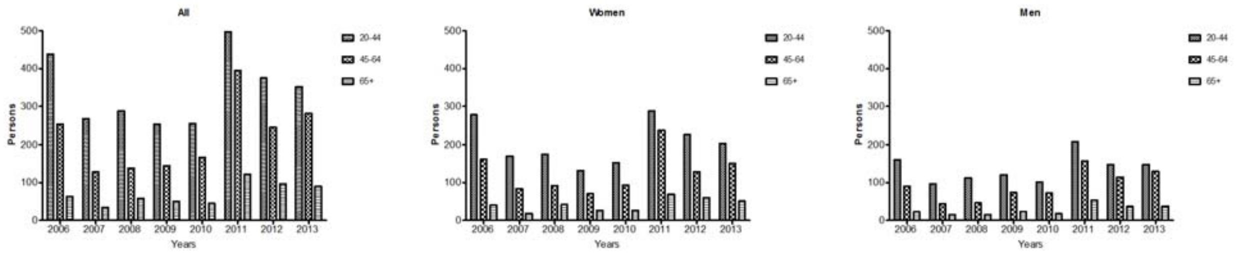

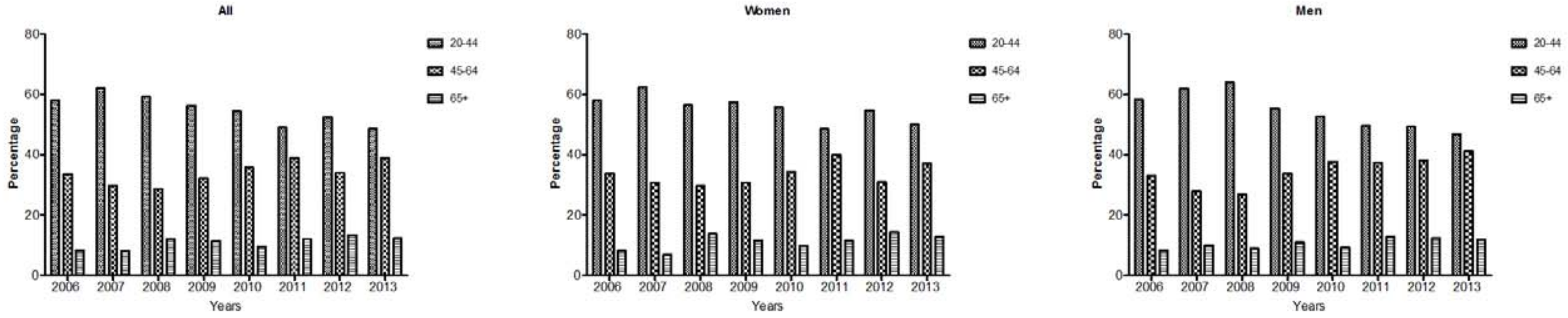

Supplementary Figure 4. Survival curve by year and age group in Korea from 2006 through 2015.

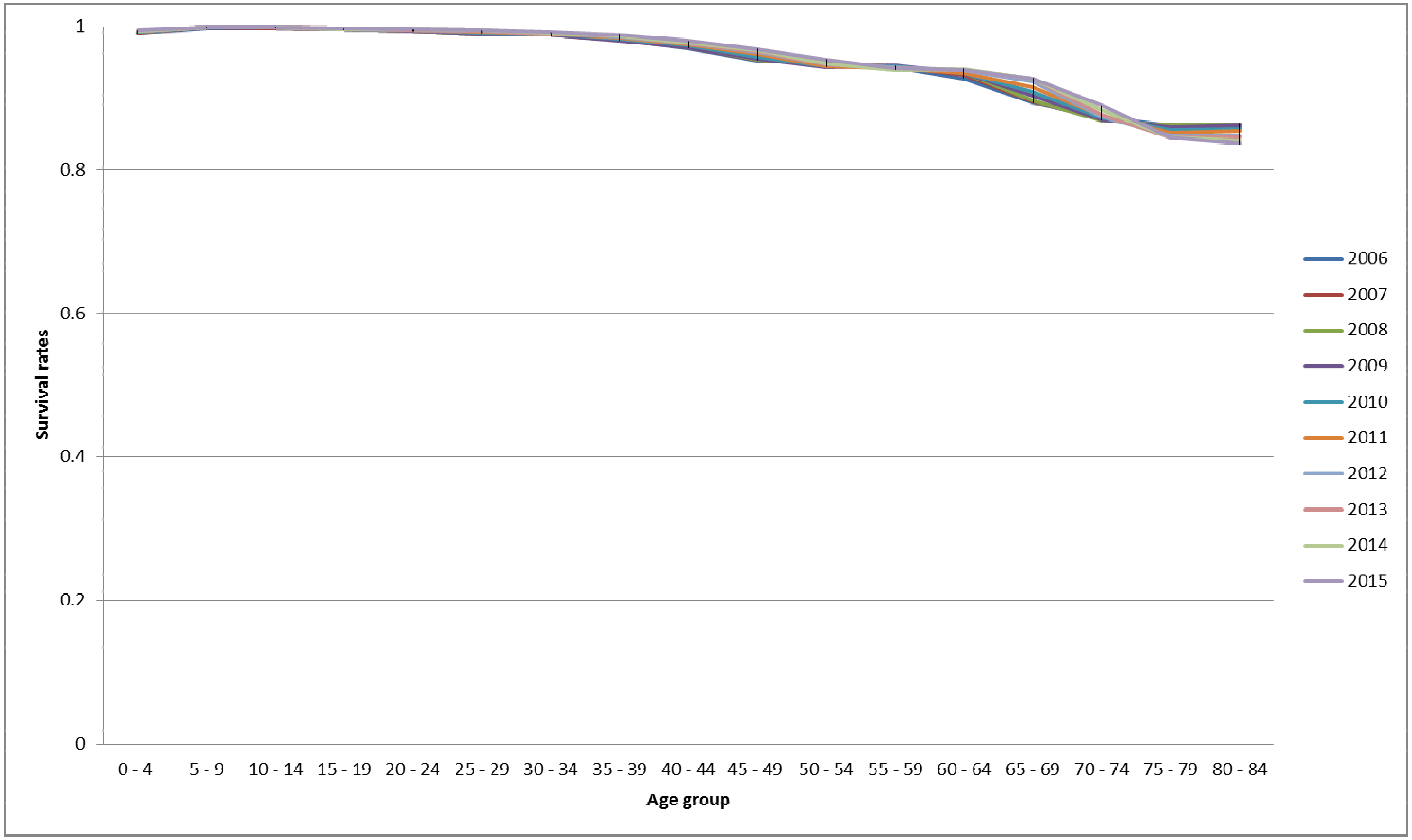

The survival curve was calculated based on the percentage of deaths for each age divided by the total number of deaths per year from 2006 through 2015 from Census in Korean.

Supplementary Table 1. Age-standardized prevalence[a] and 95% confidence interval (CI) of congenital heart disease overall and by sex (per 100,000).

Variables	2006		2007		2008		2009		2010	
	n	Prevalence (95% CI)	n	Prevalence (95% CI)	n	Prevalence (95% CI)	n	Prevalence (95% CI)	n	Prevalence (95% CI)
All	49,010	100.8 (99.9, 101.7)	54,066	110.2 (109.3, 111.1)	54,852	112.2 (111.2, 113.1)	57,882	117.2 (116.2, 118.2)	63,320	127.5 (126.5, 128.5)
Adults	11,193	35.8 (35.1, 36.4)	12,393	38.1 (37.4, 38.8)	13,181	39.4 (38.7, 40.1)	14,684	42.5 (41.8, 43.2)	16,852	47.8 (47.0, 48.5)
0–4 years old	23,838	1065.2 (1051.7, 1078.7)	26,781	1189.1 (1174.9, 1203.3)	27,367	1225.4 (1210.9, 1239.9)	28,135	1260.6 (1245.9, 1275.3)	30,580	1348.2 (1333.1, 1363.3)
5–9 years old	7,081	236.7 (231.2, 242.2)	7,287	259.0 (253.1, 265.0)	6,670	249.0 (243.0, 255.0)	6,830	263.9 (257.7, 270.2)	6,953	290.6 (283.7, 297.4)
10–14 years old	4,249	126.4 (122.6, 130.2)	4,458	135.0 (131.0, 139.0)	4,294	132.9 (128.9, 136.8)	4,603	144.7 (140.5, 148.9)	4,738	151.9 (147.5, 156.2)
15–19 years old	2,649	87.6 (84.2, 90.9)	3,147	100.3 (96.8, 103.8)	3,340	104.0 (100.4, 107.5)	3,630	109.9 (106.4, 113.5)	4,197	124.6 (120.8, 128.4)
20–44 years old	5,746	28.3 (27.5, 29.0)	6,281	31.2 (30.4, 32.0)	6,512	32.6 (31.8, 33.3)	7,103	35.8 (35.0, 36.7)	7,788	39.7 (38.8, 40.5)
45–64 years old	3,593	31.4 (30.4, 32.4)	4,117	34.7 (33.7, 35.8)	4,431	36.2 (35.1, 37.2)	5,091	40.1 (39.0, 41.2)	5,991	45.4 (44.3, 46.6)
Over 65 years	1,854	45.5 (43.4, 47.5)	1,995	45.4 (43.4, 47.4)	2,238	48.6 (46.6, 50.6)	2,490	51.5 (49.5, 53.6)	3,073	61.7 (59.5, 63.9)
Women	24,713	51.5 (50.9, 52.2)	27,249	56.3 (55.6, 56.9)	27,890	57.6 (56.9, 58.3)	29,336	59.9 (59.2, 60.6)	31,994	64.9 (64.2, 65.6)
Adults	6,642	21.5 (21.0, 22.0)	7,302	22.7 (22.2, 23.3)	7,767	23.4 (22.9, 24.0)	8,462	24.7 (24.2, 25.2)	9,594	27.4 (26.8, 27.9)
0–4 years old	11,821	528.2 (518.7, 537.7)	13,334	592.0 (582.0, 602.1)	13,723	614.4 (604.1, 624.7)	14,145	633.8 (623.3, 644.2)	15,428	680.1 (669.4, 690.9)
5–9 years old	3,448	115.2 (111.4, 119.1)	3,482	123.7 (119.6, 127.9)	3,251	121.3 (117.2, 125.5)	3,317	128.1 (123.8, 132.5)	3,334	139.3 (134.5, 144.0)
10–14 years old	1,850	55.0 (52.5, 57.5)	1,950	59.0 (56.4, 61.6)	1,912	59.1 (56.5, 61.8)	2,022	63.5 (60.8, 66.3)	2,150	68.9 (66.0, 71.8)
15–19 years old	952	31.4 (29.4, 33.4)	1,181	37.6 (35.5, 39.8)	1,237	38.5 (36.3, 40.6)	1,390	42.1 (39.8, 44.3)	1,488	44.1 (41.9, 46.4)
20–44 years old	3,204	15.7 (15.2, 16.3)	3,465	17.2 (16.6, 17.8)	3,637	18.2 (17.6, 18.8)	3,880	19.5 (18.9, 20.2)	4,197	21.3 (20.7, 22.0)
45–64 years old	2,275	19.9 (19.0, 20.7)	2,540	21.4 (20.6, 22.2)	2,693	22.0 (21.1, 22.8)	3,030	23.8 (23.0, 24.7)	3,469	26.3 (25.4, 27.2)
Over 65 years	1,163	28.5 (26.9, 30.1)	1,297	29.5 (27.9, 31.1)	1,437	31.2 (29.6, 32.8)	1,552	32.1 (30.5, 33.7)	1,928	38.7 (36.9, 40.4)
Men	24,297	49.2 (48.6, 49.8)	26,817	53.9 (53.2, 54.5)	26,962	54.5 (53.8, 55.2)	28,546	57.2 (56.5, 57.9)	31,326	62.5 (61.8, 63.2)
Adults	4,551	14.2 (13.8, 14.6)	5,091	15.3 (14.9, 15.7)	5,414	15.9 (15.5, 16.3)	6,222	17.8 (17.3, 18.2)	7,258	20.3 (19.8, 20.8)
0–4 years old	12,017	536.9 (527.3, 546.6)	13,447	597.0 (586.9, 607.1)	13,644	610.9 (600.6, 621.1)	13,990	626.8 (616.4, 637.2)	15,152	668.0 (657.3, 678.6)
5–9 years old	3,633	121.4 (117.4, 125.3)	3,805	135.2 (130.9, 139.5)	3,419	127.6 (123.3, 131.9)	3,513	135.7 (131.2, 140.2)	3,619	151.2 (146.2, 156.1)
10–14 years old	2,399	71.3 (68.5, 74.2)	2,508	75.9 (72.9, 78.9)	2,382	73.7 (70.7, 76.6)	2,581	81.1 (78.0, 84.3)	2,588	82.9 (79.7, 86.1)
15–19 years old	1,697	56.1 (53.4, 58.8)	1,966	62.7 (59.9, 65.4)	2,103	65.4 (62.6, 68.2)	2,240	67.8 (65.0, 70.6)	2,709	80.4 (77.3, 83.4)
20–44 years old	2,542	12.5 (12.0, 13.0)	2,816	14.0 (13.4, 14.5)	2,875	14.3 (13.8, 14.9)	3,223	16.2 (15.7, 16.8)	3,591	18.3 (17.7, 18.9)
45–64 years old	1,318	11.5 (10.9, 12.1)	1,577	13.3 (12.6, 13.9)	1,738	14.2 (13.5, 14.8)	2,061	16.2 (15.5, 16.9)	2,522	19.1 (18.4, 19.8)
Over 65 years	691	16.9 (15.6, 18.2)	698	15.9 (14.7, 17.0)	801	17.4 (16.1, 18.6)	938	19.4 (18.1, 20.6)	1,145	22.9 (21.6, 24.3)

Cont. Suppl. Table 1.

Variables	2011		2012		2013		2014		2015	
	n	Prevalence (95% CI)	n	Prevalence (95% CI)	n	Prevalence (95% CI)	n	Prevalence (95% CI)	n	Prevalence (95% CI)
All	66,818	133.2 (132.2, 134.2)	74,509	147.7 (146.6, 148.8)	77,526	153.1 (152.0, 154.1)	80,832	158.6 (157.5, 159.7)	85,188	167.5 (166.4, 168.7)
Adults	18,891	52.0 (51.2, 52.7)	21,980	58.7 (57.9, 59.5)	23,380	60.4 (59.6, 61.2)	25,003	62.5 (61.8, 63.3)	26,917	65.6 (64.8, 66.4)
0–4 years old	32,359	1406.5 (1391.1, 1421.8)	36,622	1595.2 (1579.2, 1611.9)	37,815	1659.7 (1643.0, 1676.5)	39,329	1726.8 (1709.7, 1743.8)	41,353	1838.2 (1820.5, 1855.9)
5–9 years old	6,554	286.0 (279.1, 293.0)	6,708	291.7 (284.7, 298.6)	6,949	304.7 (297.5, 311.8)	7,144	316.2 (308.8, 323.5)	7324	319.3 (312.0, 326.6)
10–14 years old	4,707	156.5 (152.0, 161.0)	4,734	166.2 (161.5, 171.0)	4,814	177.3 (172.3, 182.3)	4,657	180.4 (175.3, 185.6)	4687	196.9 (191.3, 202.6)
15–19 years old	4,307	128.0 (124.2, 131.9)	4,465	134.2 (13.02, 138.1)	4,568	139.7 (135.7, 143.8)	4,699	147.5 (143.3, 151.8)	4907	157.6 (153.2, 162.0)
20–44 years old	8,245	42.1 (41.2, 43.0)	9,023	46.4 (45.4, 47.3)	9,402	48.7 (47.8, 49.7)	9,725	50.8 (49.8, 51.8)	10,332	54.6 (53.5, 55.6)
45–64 years old	7,027	51.6 (50.4, 52.8)	8,474	60.5 (59.2, 61.8)	9,096	63.0 (61.7, 64.2)	9,842	66.0 (64.7, 67.3)	10,665	69.6 (68.3, 70.9)
Over 65 years	3,619	69.8 (67.5, 72.0)	4,483	81.9 (79.5, 84.3)	4,882	85.0 (82.6, 87.4)	5,436	90.5 (88.1, 92.9)	5,920	95.1 (92.7, 97.5)
Women	33,671	67.5 (66.8, 68.2)	37,654	74.9 (74.2, 75.7)	38,443	77.4 (76.6, 78.1)	40,545	79.6 (78.9, 80.4)	42,552	83.8 (83.0, 84.6)
Adults	10,371	28.7 (28.2, 29.3)	11,895	31.9 (31.3, 32.5)	10,884	32.4 (31.8, 33.0)	13,222	33.2 (32.6, 33.7)	14,046	34.3 (33.7, 34.9)
0–4 years old	16,448	714.9 (703.9, 725.8)	18,816	819.7 (808.0, 831.4)	18,571	844.6 (832.7, 856.5)	20,011	878.5 (866.4, 890.7)	20,859	927.2 (914.6, 939.8)
5–9 years old	3,136	136.8 (132.0, 141.6)	3,158	137.3 (132.5, 142.1)	3,503	151.0 (146.0, 156.1)	3,428	151.7 (146.6, 156.7)	3,641	160.5 (155.4, 165.7)
10–14 years old	2,180	72.4 (69.4, 75.5)	2,213	77.6 (74.4, 80.9)	2,580	82.2 (78.8, 85.7)	2,168	84.0 (80.4, 87.5)	2,193	92.1 (88.3, 96.0)
15–19 years old	1,536	45.6 (43.3, 47.9)	1,572	47.2 (44.9, 49.5)	2,905	50.8 (48.4, 53.3)	1,716	53.8 (51.3, 56.4)	1,813	58.2.4 (55.5, 60.9)
20–44 years old	4,274	21.8 (21.1, 22.5)	4,657	23.9 (23.2, 24.6)	4,599	24.9 (24.2, 25.6)	4,856	25.3 (24.6, 26.1)	5,114	27.0 (26.2, 27.7)
45–64 years old	3,890	28.6 (27.7, 29.4)	4,566	32.3 (31.6, 33.5)	4,318	33.0 (32.1, 34.0)	5,192	34.8 (33.9, 35.8)	5,474	35.7 (34.7, 36.6)
Over 65 years	2,207	42.5 (40.7, 44.3)	2,672	48.8 (47.0, 50.7)	1,967	50.7 (48.9, 52.6)	3,174	52.8 (51.0, 54.6)	3,458	55.5 (53.7, 57.4)
Men	33,147	65.6 (64.9, 66.3)	36,855	72.7 (72.0, 73.5)	38,443	75.7 (74.9, 76.4)	40,287	78.9 (78.1, 79.7)	42,636	92.1 (91.3, 92.9)
Adults	8,520	23.2 (22.7, 23.7)	10,085	26.8 (26.2, 27.3)	10,884	28.0 (27.4, 28.5)	11,781	29.3 (28.8, 29.9)	12,871	31.3 (30.7, 31.8)
0–4 years old	15,911	691.5 (680.8, 702.3)	17,806	775.7 (764.3, 787.1)	18,571	815.1 (803.3, 826.8)	19,318	848.1 (836.2, 860.1)	20,494	910.9 (898.5, 923.4)
5–9 years old	3,418	149.1 (144.1, 154.1)	3,550	154.3 (149.2, 159.4)	3,503	153.6 (148.5, 158.7)	3,716	164.4 (159.2, 169.7)	3,683	346.6 (338.9, 354.2)
10–14 years old	2,527	84.0 (80.7, 87.3)	2,521	88.5 (85.0, 91.9)	2,580	95.0 (91.3, 98.6)	2,489	96.4 (92.6, 100.2)	2,494	104.8 (100.7, 108.9)
15–19 years old	2,771	82.4 (79.3, 85.4)	2,893	86.9 (83.7, 90.1)	2,905	88.8 (85.6, 92.1)	2,983	93.6 (90.3, 97.0)	3,094	99.4 (95.9, 102.9)
20–44 years old	3,971	20.3 (19.6, 20.9)	4,366	22.4 (21.7, 23.1)	4,599	23.8 (23.1, 24.5)	4,869	25.4 (24.7, 26.1)	5,218	27.5 (26.8, 28.3)
45–64 years old	3,137	23.0 (22.2, 23.8)	3,908	27.9 (27.0, 28.8)	4,318	29.9 (29.0, 30.7)	4,650	31.2 (30.3, 32.1)	5,191	33.8 (3239, 34.8)
Over 65 years	1,412	27.2 (25.8, 28.6)	1,811	33.1 (31.5, 34.6)	1,967	34.2 (32.7, 35.7)	2,262	37.6 (36.1, 39.2)	2,462	39.5 (37.9, 41.1)

a Age-standardized prevalence rates of congenital heart disease (ICD 10 codes: Q20, Q21, Q22, Q23, Q24, Q25, and Q26) were calculated using age groups according to the direct method using the estimated Korean population in 2015 as a reference.

Supplementary Table 1-1. Age-standardized prevalence[a] and 95% confidence interval (CI) of congenital ventricular and/or atrial septal defects overall and by sex (per 100,000).

Variables	2006		2007		2008		2009		2010	
	n	Prevalence (95% CI)	n	Prevalence (95% CI)	n	Prevalence (95% CI)	n	Prevalence (95% CI)	n	Prevalence (95% CI)
All	26,793	55.2 (54.8, 56.2)	29,210	60.0 (59.3, 60.7)	29,676	61.1 (60.4, 61.8)	31,932	65.1 (64.4. 65.8)	35,656	72.1 (71.3, 72.8)
Adults	6,660	21.2 (20.6, 21.7)	7,365	22.5 (22.0, 23.0)	7,671	22.9 (22.4, 23.4)	8,411	24.4 (23.9, 24.9)	9,497	26.9 (26.3, 27.4)
0–4 years old	13,086	584.7 (574.7, 594.7)	14,632	649.6 (63.91, 660.2)	15,236	682.2 (671.4, 693.0)	16,328	731.5 (720.3, 742.8)	18,624	821.1 (809.3, 832.9)
5–9 years old	3,635	121.5 (117.5, 125.4)	3,605	128.1 (124.0, 132.3)	3,233	120.7 (116.5, 124.8)	3,393	131.1 (126.7, 135.5)	3,468	144.9 (140.0, 149.7)
10–14 years old	1,995	59.3 (56.7, 61.9)	1,995	60.4 (57.7, 63.0)	1,872	57.9 (55.3, 60.5)	2,056	64.6 (61.8, 67.4)	2,022	64.8 (62.0, 67.6)
15–19 years old	1,417	46.8 (44.4, 49.3)	1,613	51.4 (48.9, 53.9)	1,664	51.8 (49.3, 54.3)	1,744	52.8 (50.3, 55.3)	2,045	60.7 (58.0, 63.3)
20–44 years old	3,444	16.9 (16.3, 17.5)	3,749	18.6 (18.0, 19.2)	3,731	18.6 (18.0, 19.2)	3,964	20.0 (19.4, 20.6)	4,332	22.0 (21.4, 22.7)
45–64 years old	2,197	19.2 (18.4, 20.3)	2,550	21.5 (20.7, 22.3)	2,730	22.3 (21.4, 23.1)	3,084	24.2 (23.4, 25.1)	3,525	26.7 (25.8, 27.6)
Over 65 years	1,019	25.0 (23.4, 26.5)	1,066	24.2 (22.8, 25.7)	1,210	26.3 (24.8, 27.7)	1,363	28.2 (26.7, 29.7)	1,640	32.9 (31.3, 34.5)
Women	14,038	29.4 (28.9, 29.9)	15,253	31.7 (31.2, 32.2)	15,705	32.6 (32.1, 33.1)	16,893	34.7 (34.2, 35.2)	18,733	38.1 (37.6, 38.7)
Adults	4,098	13.2 (12.7, 13.6)	4,539	14.0 (13.6, 14.4)	4,692	14.1 (13.7, 14.5)	5,061	14.8 (14.3, 15.2)	5,652	16.1 (15.7, 16.5)
0–4 years old	6,668	297.9 (290.8, 305.1)	7,456	331.0 (323.5, 338.5)	7,835	350.8 (343.0, 358.5)	8,445	378.3 (370.2, 386.4)	9,661	425.9 (417.4, 434.4)
5–9 years old	1,853	61.9 (59.0, 64.7)	1,807	64.2 (61.2, 67.1)	1,707	63.7 (60.7, 66.7)	1,759	67.9 (64.7, 71.1)	1,750	73.1 (69.6, 76.5)
10–14 years old	922	27.4 (25.6, 29.1)	886	26.8 (25.0, 28.6)	871	26.9 (25.1, 28.7)	971	30.5 (28.5, 32.4)	992	31.7 (29.8, 33.7)
15–19 years old	497	16.4 (14.9, 17.8)	565	18.0 (16.5, 19.4)	600	18.6 (17.1, 20.1)	657	19.9 (18.3, 21.4)	678	20.1 (18.6, 21.6)
20–44 years old	1,992	9.80 (9.37, 10.2)	2,177	10.8 (10.3, 11.2)	2,168	10.8 (10.3, 11.3)	2,269	11.4 (10.9, 11.9)	2,463	12.5 (12.0, 13.0)
45–64 years old	1,464	12.8 (12.1, 13.4)	1,660	14.0 (13.3, 14.7)	1,736	14.1 (13.5, 14.8)	1,931	15.2 (14.5, 15.8)	2,139	16.2 (15.5, 16.9)
Over 65 years	642	15.7 (14.5, 16.9)	702	15.9 (14.8, 17.1)	788	17.1 (15.9, 18.2)	861	17.8 (16.6, 19.0)	1,050	21.0 (19.8, 22.3)
Men	12,755	26.0 (25.5, 26.5)	13,957	61.1 (60.4, 61.8)	13,971	28.5 (28.0, 28.9)	15,039	30.4 (29.9, 30.8)	16,923	33.9 (33.4, 34.4)
Adults	2,562	7.99 (7.67, 8.31)	2,826	8.38 (8.06, 8.69)	2,979	8.78 (8.46, 9.10)	3,350	9.62 (9.29, 9.95)	3,845	10.7 (10.4, 11.1)
0–4 years old	6,418	286.7 (279.7, 293.8)	7,176	318.6 (311.2, 325.9)	7,401	331.3 (323.8, 338.9)	7,883	353.1 (345.3, 360.9)	8,963	395.1 (386.9, 403.3)
5–9 years old	1,782	59.5 (56.8, 62.3)	1,798	63.9 (60.9, 66.8)	1,526	56.9 (54.0, 59.8)	1,634	63.1 (60.0, 66.1)	1,718	71.7 (68.3, 75.1)
10–14 years old	1,073	31.9 (30.0, 33.8)	1,109	33.5 (31.5, 35.5)	1,001	30.9 (29.0, 32.8)	1,085	34.1 (32.0, 36.1)	1,030	32.9 (30.9, 35.0)
15–19 years old	920	30.4 (28.4, 32.4)	1,048	33.4 (31.1, 35.4)	1,064	33.1 (31.1, 35.1)	1,087	32.9 (30.9, 34.8)	1,367	40.5 (38.4, 42.7)
20–44 years old	1,452	7.15 (6.78, 7.51)	1,572	7.81 (7.43, 8.20)	1,563	7.82 (7.43, 8.21)	1,695	8.56 (5.15, 8.96)	1,869	9.52 (9.09, 9.95)
45–64 years old	733	6.41 (5.94, 6.87)	890	7.51 (7.02, 8.01)	994	8.12 (7.61, 8.62)	1,153	9.07 (8.55, 9.60)	1,386	10.5 (9.96, 11.0)
Over 65 years	377	9.25 (8.32, 10.1)	364	8.29 (7.44, 9.14)	422	9.16 (8.28, 10.0)	502	10.3 (9.48, 11.3)	590	11.8 (10.8, 12.8)

Cont. Suppl. Table 1-1.

Variables	2011		2012		2013		2014		2015	
	n	Prevalence (95% CI)	n	Prevalence (95% CI)	n	Prevalence (95% CI)	n	Prevalence (95% CI)	n	Prevalence (95% CI)
All	33,383	67.0 (66.3, 67.8)	38,431	76.5 (75.7, 77.2)	39,865	78.9 (78.1, 79.7)	40,990	80.4 (79.6, 81.2)	43,778	86.0 (85.2, 86.8)
Adults	10,034	27.6 (27.1, 28.2)	11,308	30.2 (29.6, 30.7)	11,841	30.5 (30.0, 31.1)	12,082	30.2 (29.7, 30.7)	12,999	31.7 (31.1, 32.2)
0–4 years old	18,901	821.5 (809.7, 833.2)	22,464	978.7 (965.9, 991.5)	23,299	1022.6 (1009.5, 1035.7)	24,424	1072.3 (1058,9, 1085.8)	26,143	1162.1 (1148.0, 1176.2)
5–9 years old	1,933	82.3 (80.5, 88.0)	2,071	90.0 (86.1, 93.9)	2,155	94.5 (90.5, 98.4)	2,078	91.9 (88.0, 95.9)	2,075	90.4 (86.5, 94.3)
10–14 years old	1,209	40.1 (37.9, 42.4)	1,238	43.4 (41.0, 45.8)	1,228	45.2 (42.7, 47.7)	1,112	43.0 (40.5, 45.6)	1,203	50.5 (47.6, 53.3)
15–19 years old	1,306	38.8 (36.7, 40.9)	1,350	40.5 (38.3, 42.7)	1,342	41.0 (38.8, 43.2)	1,294	40.6 (38.4, 42.8)	1,358	43.6 (41.3, 45.9)
20–44 years old	4,304	22.0 (21.3, 22.6)	4,682	24.0 (23.3, 24.7)	4,786	24.8 (24.1, 25.5)	4,605	24.0 (23.3, 24.7)	4,881	25.7 (25.0, 26.5)
45–64 years old	3,840	28.2 (27.3, 29.1)	4,362	31.1 (30.2, 32.1)	4,646	32.1 (31.2, 33.1)	4,907	32.9 (32.0, 33.8)	5,331	34.8 (33.8, 35.7)
Over 65 years	1,890	36.4 (34.8, 38.0)	2,264	41.3 (39.6, 43.0)	2,409	41.9 (40.2, 43.6)	2,570	42.7 (41.1, 44.4)	2,787	44.7 (43.1, 46.5)
Women	18,082	36.5 (35.9, 37.0)	20,793	41.5 (40.9, 42.1)	21,445	42.5 (41.9, 43.1)	21,966	43.1 (42.5, 43.7)	23,311	45.8 (45.2, 46.4)
Adults	5,853	16.2 (15.8, 16.6)	6,610	17.7 (17.2, 18.1)	6,807	17.6 (17.2, 18.0)	6,916	17.3 (16.9, 17.7)	7,360	17.9 (17.5, 18.3)
0–4 years old	10,363	450.4 (441.7, 459.1)	12,274	534.7 (525.2, 544.2)	12,629	554.3 (544.6, 563.9)	13,192	579.1 (569.2, 589.0)	13,947	619.9 (609.6, 630.2)
5–9 years old	942	41.1 (38.4, 43.7)	961	41.7 (39.1, 44.4)	1,042	45.6 (42.8, 48.4)	970	42.9 (40.2, 45.6)	991	43.1 (40.4, 45.8)
10–14 years old	558	18.5 (16.9, 20.0)	578	20.2 (18.6, 21.9)	581	21.3 (19.6, 23.1)	502	19.4 (17.7, 21.1)	577	24.2 (22.2, 26.2)
15–19 years old	366	10.8 (9.76, 11.9)	370	11.1 (9.96, 12.2)	386	11.7 (10.6, 12.9)	386	12.1 (10.9, 13.3)	436	14.0 (12.6, 15.3)
20–44 years old	2,404	12.2 (11.7, 12.7)	2,636	13.5 (13.0, 14.0)	2,664	13.8 (13.2, 14.3)	2,541	13.2 (12.7, 13.8)	2,671	14.1 (13.5, 14.6)
45–64 years old	2,267	16.6 (15.9, 17.3)	2,561	18.3 (17.5, 19.0)	2,632	18.2 (17.5, 18.9)	2,780	18.6 (17.9, 19.3)	2,962	19.3 (18.6, 20.0)
Over 65 years	1,182	22.7 (21.4, 24.0)	1,413	25.8 (24.4, 27.1)	1,511	26.3 (24.9, 27.6)	1,595	26.5 (25.2, 27.8)	1,727	27.7 (26.4, 29.0)
Men	15,301	30.5 (30.0, 31.0)	17,638	34.9 (34.4, 35.4)	18,420	36.3 (35.8, 36.8)	19,024	37.2 (36.7, 37.8)	20,467	40.2 (39.6, 40.7)
Adults	4,181	11.4 (11.0, 11.7)	4,698	12.4 (12.1, 12.8)	5,034	12.9 (12.5, 13.3)	5,166	12.8 (12.5, 13.2)	5,639	13.7 (13.3, 14.0)
0–4 years old	8,538	371.0 (363.2, 378.9)	10,190	443.9 (435.3, 452.5)	10,670	468.3 (459.4, 477.2)	11,232	493.1 (484.0, 502.2)	12,196	542.1 (532.5, 551.7)
5–9 years old	991	43.2 (40.5, 45.9)	1,110	48.2 (45.4, 51.0)	1,113	48.7 (45.9, 51.6)	1,108	49.0 (46.1, 51.9)	1,084	47.2 (44.4, 50.0)
10–14 years old	651	21.6 (19.9, 23.2)	660	23.1 (21.3, 24.9)	647	23.8 (21.9, 25.6)	610	23.6 (21.7, 25.4)	626	26.2 (24.2, 28.3)
15–19 years old	940	27.9 (26.1, 29.7)	980	29.4 (27.6, 31.3)	956	29.2 (27.3, 31.0)	908	28.5 (26.6, 30.3)	922	29.6 (27.7, 31.5)
20–44 years old	1,900	9.71 (9.27, 10.1)	2,046	10.5 (10.0, 10.9)	2,122	11.0 (10.5, 11.4)	2,064	10.7 (10.3, 11.2)	2,210	11.6 (11.1, 12.1)
45–64 years old	1,573	11.5 (10.9, 12.1)	1,801	12.8 (12.2, 13.4)	2,014	13.9 (13.3, 14.5)	2,127	14.2 (13.6, 14.8)	2,369	15.4 (14.8, 16.0)
Over 65 years	708	13.6 (12.6, 14.6)	851	15.5 (14.5, 16.6)	898	15.6, 14.6, 16.6)	975	16.2 (15.2, 17.2)	1,060	17.0 (15.9, 18.0)

a Age-standardized prevalence rates of congenital ventricular and/or atrial septal defects were calculated using age groups according to the direct method using the estimated Korean population in 2015 as a reference.

Supplementary Table 1-1-1. Age-standardized prevalence[a] and 95% confidence interval (CI) of congenital ventricular septal defects overall and by sex (per 100,000).

Variables	2011		2012		2013		2014		2015	
	n	Prevalence (95% CI)	n	Prevalence (95% CI)	n	Prevalence (95% CI)	n	Prevalence (95% CI)	n	Prevalence (95% CI)
All	13,221	25.8 (25.3, 26.2)	14,584	28.3 (27.9, 28.8)	14,844	28.9 (28.4, 29.4)	14,916	29.0 (28.6, 29.5)	15,761	30.9 (30.4, 31.4)
Adults	2,803	7.32 (7.05, 7.59)	3,039	7.76 (7.48, 8.04)	3,118	7.79 (7.51, 8.06)	3,076	7.51 (7.24, 7.78)	3,287	7.90 (7.63, 8.17)
0–4 years old	6,194	269.2 (262.5, 275.9)	7,075	308.2 (301.0, 315.4)	7,139	313.3 (306.0, 320.5)	7,456	327.3 (319.9, 334.8)	7,945	353.1 (345.3, 360.9)
5–9 years old	1,833	79.9 (769.3, 83.6)	1,981	86.1 (82.3, 89.9)	2,091	91.7 (87.7, 95.6)	2,034	90.0 (86.1, 93.9)	2,024	88.2 (84.3, 92.0)
10–14 years old	1,147	38.1 (35.9, 40.3)	1,185	41.5 (39.2, 43.9)	1,185	43.6 (41.1, 46.1)	1,088	42.1 (39.6, 44.6)	1,181	49.6 (46.7, 52.4)
15–19 years old	1,244	36.9 (34.9, 39.0)	1,304	39.1 (37.0, 41.3)	1,311	40.0 (37.9, 42.2)	1,262	39.6 (37.4, 41.8)	1,324	42.5 (40.2, 44.8)
20–44 years old	1,794	9.17 (8.74, 9.59)	1,895	9.74 (9.30, 10.1)	1,911	9.91 (9.47, 10.3)	1,843	9.63 (9.19, 10.0)	1,923	10.1 (9.70, 10.6)
45–64 years old	765	5.62 (5.22, 6.01)	875	6.25 (5.88, 6.66)	917	6.34 (5.93, 6.75)	933	6.25 (5.85, 6.66)	1,013	6.61 (6.20, 7.01)
Over 65 years	244	4.70 (4.11, 5.29)	269	4.914 (4.32, 5.50)	290	5.03 (4.45, 5.62)	300	4.99 (4.42, 5.55)	351	5.63 (5.04, 6.22)
Women	6,849	13.4 (13.1, 13.7)	7,465	14.5 (14.2, 14.9)	7,687	15.0 (14.6, 15.3)	7,631	14.8 (14.5, 15.2)	8,055	15.8 (15.4, 16.1)
Adults	1,418	3.74 (3.54, 3.94)	1,538	3.96 (3.76, 4.16)	1,584	3.98 (3.78, 4.18)	1,515	3.71 (3.52, 3.90)	1,598	3.85 (3.66, 4.04)
0–4 years old	3,675	159.7 (154.5, 164.8)	4,086	178.0 (172.5, 183.4)	4,151	182.1 (176.6, 187.7)	4,287	188.1 (182.5, 193.8)	4,492	199.6 (193.8, 205.4)
5–9 years old	880	38.3 (35.8, 40.9)	925	40.2 (37.6, 42.8)	1,014	44.4 (41.6, 47.1)	955	42.2 (39.5, 44.9)	971	42.3 (39.6, 44.9)
10–14 years old	530	17.6 (16.1, 19.1)	557	19.5 (17.9, 21.1)	561	20.6 (18.9, 22.3)	496	19.1 (17.4, 20.8)	567	23.8 (21.87, 25.7)
15–19 years old	346	10.2 (9.19, 11.3)	359	10.7 (9.67, 11.9)	377	11.5 (10.3, 12.6)	378	11.8 (10.6, 13.0)	427	13.7 (12.4, 15.0)
20–44 years old	854	4.36 (4.07, 4.65)	905	4.65 (4.35, 4.95)	916	4.74 (4.44, 5.05)	862	4.50 (4.20, 4.80)	899	4.74 (4.43, 5.06)
45–64 years old	406	2.98 (2.68, 3.27)	456	3.25 (2.96, 3.55)	468	3.23 (2.94, 3.53)	463	3.10 (2.82, 3.38)	479	3.12 (2.84, 3.40)
Over 65 years	158	3.04 (2.56, 3.52)	177	3.22 (2.75, 3.70)	200	3.47 (2.98, 3.95)	190	3.15 (2.70, 3.60)	220	3.53 (3.06, 3.99)
Men	6,372	12.3 (12.0, 12.6)	7,119	13.8 (13.4, 14.1)	7,157	13.9 (13.5, 14.2)	7,285	14.1 (13.8, 14.5)	7,706	15.1 (14.7, 15.4)
Adults	1,385	3.57 (3.38, 3.76)	1,501	3.80 (3.60, 3.99)	1,534	3.80 (3.61, 3.99)	1,561	3.79 (3.60, 3.98)	1,689	4.04 (3.85, 4.24)
0–4 years old	2,519	109.4 (105.1, 113.7)	2,989	130.2 (125.5, 134.8)	2,988	131.1 (126.4, 135.8)	3,169	139.1 (134.2, 143.9)	3,453	153.4 (148.3, 158.6)
5–9 years old	953	41.5 (38.9, 44.2)	1,056	45.8 (43.1, 48.6)	1,077	47.2 (44.4, 50.0)	1,079	47.7 (44.9, 50.6)	1,053	45.8 (43.1, 48.6)
10–14 years old	617	20.5 (18.8, 22.1)	628	22.0 (20.3, 23.7)	624	22.9 (21.1, 24.7)	592	22.9 (21.0, 24.7)	614	25.8 (23.7, 27.8)
15–19 years old	898	26.6 (24.9, 28.4)	945	28.3 (26.5, 30.1)	934	28.5 (26.7, 30.4)	884	27.7 (25.9, 29.5)	897	28.7 (26.9, 30.6)
20–44 years old	940	4.80 (4.49, 5.11)	990	5.09 (4.77, 5.41)	995	5.16 (4.83, 5.48)	981	5.12 (4.80, 5.44)	1,024	5.40 (5.07, 5.74)
45–64 years old	359	2.63 (2.36, 2.90)	419	2.99 (2.70, 3.28)	449	3.10 (2.81, 3.39)	470	3.15 (2.86, 3.43)	534	3.48 (3.18, 3.78)
Over 65 years	86	1.64 (1.29, 1.99)	92	1.67 (1.33, 2.01)	90	1.56 (1.24, 1.89)	110	1.82 (1.48, 2.16)	131	2.10 (1.74, 2.46)

a Age-standardized prevalence rates of congenital ventricular septal defect were calculated using age groups according to the direct method using the estimated Korean population in 2015 as a reference.

Supplementary Table 1-1-2. Age-standardized prevalence[a] and 95% confidence interval (CI) of congenital atrial septal defects overall and by sex (per 100,000).

Variables	2011		2012		2013		2014		2015	
	n	Prevalence (95% CI)	n	Prevalence (95% CI)	n	Prevalence (95% CI)	n	Prevalence (95% CI)	n	Prevalence (95% CI)
All	21,302	43.2 (42.6, 43.8)	25,274	50.7 (50.0, 51.3)	26,851	53.4 (52.8, 54.0)	28,176	55.4 (54.8, 56.1)	30,405	59.8 (59.1, 60.5)
Adults	6,935	19.5 (19.0, 19.9)	8,074	21.9 (21.4, 22.3)	8,532	22.3 (21.8, 22.7)	8,840	22.3 (21.8, 22.7)	9,539	23.3 (22.9, 23.8)
0–4 years old	12,014	522.1 (512.8, 531.5)	14,634	637.5 (627.2, 647.8)	15,694	688.8 (678.0, 699.6)	16,567	727.3 (716.3, 738.4)	17,901	795.7 (784.0, 807.3)
5–9 years old	1,130	49.3 (46.4, 52.1)	1,201	52.1 (49.2, 55.1)	1,285	56.3 (53.2, 59.4)	1,422	62.9 (59.6, 66.2)	1,577	68.7 (65.3, 72.1)
10–14 years old	607	20.1 (18.5, 21.7)	651	22.8 (21.0, 24.5)	659	24.2 (22.4, 26.1)	655	25.3 (23.4, 27.2)	625	26.2 (24.1, 28.3)
15–19 years old	616	18.2 (16.8, 19.7)	714	21.4 (19.8, 23.0)	681	20.8 (19.2, 22.3)	692	21.7 (20.1, 23.3)	763	24.5 (22.7, 26.2)
20–44 years old	2,394	12.2 (11.7, 12.7)	2,702	13.8 (13.3, 14.4)	2,793	14.4 (13.9, 15.0)	2,697	14.1 (13.5, 14.6)	2,879	15.2 (14.6, 15.7)
45–64 years old	2,962	21.7 (20.9, 22.5)	3,427	24.4 (23.6, 25.3)	3,662	25.3 (24.5, 26.1)	3,912	26.2 (25.4, 27.0)	4,267	27.8 (27.0, 28.6)
Over 65 years	1,579	30.4 (28.9, 31.9)	1,945	35.5 (33.9, 37.1)	2,077	36.1 (34.6, 37.7)	2,231	37.1 (35.6, 38.6)	2,393	38.4 (36.9, 39.9)
Women	11,908	24.2 (23.8, 24.7)	14,157	28.4 (28.0, 28.9)	14,857	29.6 (29.1, 30.0)	15,547	30.6 (30.1, 31.1)	16,625	32.7 (32.2, 33.2)
Adults	4,259	11.9 (11.6, 12.3)	4,951	13.4 (13.0, 13.8)	5,111	13.3 (13.0, 13.7)	5,296	13.3 (13.0, 13.7)	5,655	13.8 (13.5, 14.2)
0–4 years old	6,352	276.0 (269.2, 282.8)	7,829	341.0 (333.5, 348.6)	8,266	362.7 (354.9, 370.6)	8,721	382.8 (374.8, 390.9)	9,293	413.0 (404.6, 421.4)
5–9 years old	653	28.4 (26.3, 30.6)	694	30.1 (27.8, 32.3)	785	34.3 (31.9, 36.8)	828	36.6 (34.1, 39.1)	959	41.8 (39.1, 44.4)
10–14 years old	370	12.2 (11.0, 13.5)	372	13.0 (11.6, 14.3)	394	14.5 (13.0, 15.9)	384	14.8 (13.3, 16.3)	368	15.4 (13.8, 17.0)
15–19 years old	274	8.13 (7.17, 9.10)	311	9.33 (8.29, 10.3)	301	9.21 (8.16, 10.2)	318	9.96 (8.86, 11.0)	350	11.2 (10.0, 12.4)
20–44 years old	1,479	7.56 (7.17, 7.94)	1,680	8.63 (8.22, 9.05)	1,696	8.79 (8.38, 9.21)	1,638	8.56 (8.15, 8.98)	1,726	9.12 (8.69, 9.55)
45–64 years old	1,797	13.2 (12.5, 13.8)	2,065	14.7 (14.1, 15.3)	2,129	14.7 (14.1, 15.3)	2,283	15.3 (14.6, 15.9)	2,454	16.0 (15.3, 16.6)
Over 65 years	983	18.9 (17.7, 20.1)	1,206	22.0 (20.7, 23.2)	1,286	22.3 (21.1, 23.6)	1,375	22.8 (21.6, 24.1)	1,475	23.7 (22.4, 24.9)
Men	9,394	19.0 (18.6, 19.3)	11,117	22.2 (21.8, 22.6)	11,994	23.8 (23.4, 24.2)	12,629	24.8 (24.4, 25.2)	13,780	27.1 (26.6, 27.5)
Adults	2,676	7.52 (7.24, 7.81)	3,123	8.48 (8.18, 8.78)	3,421	8.94 (8.64, 9.24)	3,544	8.94 (8.64, 9.23)	3,884	9.50 (9.20, 9.80)
0–4 years old	5,662	246.0 (239.6, 252.4)	6,805	296.4 (289.4, 303.5)	7,428	326.0 (318.6, 333.4)	7,846	344.4 (336.8, 352.0)	8,608	382.6 (374.5, 390.7)
5–9 years old	477	20.8 (18.9, 22.6)	507	22.0 (20.0, 23.9)	500	21.9 (20.0, 23.8)	594	26.2 (24.1, 28.3)	618	26.9 (24.8, 29.0)
10–14 years old	237	7.85 (6.85, 8.86)	279	9.75 (8.60, 10.9)	265	9.75 (8.58, 10.9)	271	10.5 (9.25, 11.7)	257	10.7 (9.47, 12.1)
15–19 years old	342	10.1 (9.07, 11.2)	403	12.1 (10.9, 13.2)	380	11.6 (10.4, 12.7)	374	11.7 (10.5, 12.9)	413	13.2 (11.9, 14.5)
20–44 years old	915	4.67 (4.37, 4.98)	1,022	5.25 (4.93, 5.57)	1,097	5.68 (5.35, 6.02)	1,059	5.53 (5.20, 5.87)	1,153	6.09 (5.74, 6.44)
45–64 years old	1,165	8.56 (8.06, 9.05)	1,362	9.73 (9.21, 10.2)	1,533	10.6 (10.0, 11.1)	1,629	10.9 (10.4, 11.4)	1,813	11.8 (11.2, 12.3)
Over 65 years	596	11.4 (10.5, 12.4)	739	13.5 (12.5, 14.4)	791	13.7 (12.8, 14.7)	856	14.2 (13.2, 15.2)	918	14.7 (13.7, 15.7)

a Age-standardized prevalence rates of congenital atrial septal defect were calculated using age groups according to the direct method using the estimated Korean population in 2015 as a reference.

Supplementary Table 1-2. Age-standardized prevalence[a] and 95% confidence interval (CI) of patent ductus arteries overall and by sex (per 100,000).

Variables	2006		2007		2008		2009		2010	
	n	Prevalence (95% CI)	n	Prevalence (95% CI)	n	Prevalence (95% CI)	n	Prevalence (95% CI)	n	Prevalence (95% CI)
All	4,361	9.11 (8.83, 9.38)	5,259	10.8 (10.5, 11.1)	5,598	11.5 (11.2, 11.8)	5,987	12.2 (11.9, 12.5)	6,481	13.0 (12.7, 13.3)
Adults	618	1.94 (1.78, 2.10)	627	1.87 (1.72, 2.02)	678	1.98 (1.82, 2.13)	760	2.15 (1.99, 2.30)	823	2.28 (2.12, 2.44)
0–4 years old	3,173	141.7 (136.8, 146.6)	3,989	177.1(171.6, 182.6)	4,339	194.2 (188.5, 200.0)	4,561	204.3 (198.4, 210.2)	4,926	217.1 (211.0, 223.2)
5–9 years old	335	11.1 (9.98, 12.3)	362	12.8 (11.5, 14.1)	322	11.9 (10.6, 13.2)	361	13.9 (12.4, 15.3)	382	15.9 (14.3, 17.5)
10–14 years old	140	4.13 (3.44, 4.82)	155	4.67 (3.93, 5.41)	138	4.25 (3.54, 4.97)	154	4.83 (4.07, 5.60)	200	6.40 (5.52, 7.29)
15–19 years old	95	3.12 (2.49, 3.75)	126	4.00 (3.30, 4.70)	121	3.75 (3.08, 4.42)	151	4.57 (3.84, 5.30)	150	4.44 (3.73, 5.16)
20–44 years old	329	1.62 (1.44, 1.79)	345	1.71 (1.53, 1.89)	363	1.81 (1.62, 2.00)	400	2.01 (1.82, 2.21)	413	2.10 (1.90, 2.30)
45–64 years old	203	1.77 (1.53, 2.02)	215	1.81 (1.57, 2.05)	240	1.95 (1.70, 2.20)	285	2.24 (1.98, 2.50)	321	2.43 (2.16, 2.70)
Over 65 years	86	2.10 (1.65, 2.54)	67	1.52 (1.15, 1.88)	75	1.62 (1.26, 1.99)	75	1.55 (1.20, 1.90)	89	1.78 (1.41, 2.15)
Women	2,440	5.13 (4.92, 5.34)	2,902	5.99 (5.77, 6.21)	3,102	9.42 (6.19, 6.65)	3,292	6.74 (6.51, 6.98)	3,538	7.14 (6.90, 7.38)
Adults	472	1.50 (1.36, 1.64)	490	1.47 (1.34, 1.61)	523	1.52 (1.39, 1.66)	595	1.69 (1.55, 1.83)	635	1.76 (1.63, 1.90)
0–4 years old	1,654	73.9 (70.3, 77.4)	2,044	90.7 (86.7, 94.6)	2,256	101.0 (96.8, 105.1)	2,320	103.9 (99.6, 108.1)	2,514	110.8 (106.4, 115.1)
5–9 years old	199	6.61 (5.68, 7.53)	215	7.63 (6.61, 8.65)	190	7.05 (6.04, 8.06)	226	8.70 (7.56, 9.83)	223	9.27 (8.05, 10.5)
10–14 years old	82	2.43 (1.91, 2.96)	93	2.81 (2.23, 3.38)	76	2.31 (1.78, 2.84)	87	2.72 (2.15, 3.30)	111	3.55 (2.89, 4.21)
15–19 years old	33	1.07 (0.69, 1.44)	60	1.89 (1.40, 2.37)	57	1.76 (1.30, 2.22)	64	1.92 (1.44, 2.39)	55	1.60 (1.17, 2.04)
20–44 years old	239	1.17 (1.02, 1.32)	264	1.30 (1.15, 1.46)	277	1.38 (1.21, 1.54)	301	1.52 (1.34, 1.69)	308	1.57 (1.39, 1.74)
45–64 years old	165	1.44 (1.22, 1.66)	166	1.39 (1.18, 1.61)	188	1.53 (1.31, 1.75)	234	1.84 (1.60, 2.07)	257	1.94 (1.71, 2.18)
Over 65 years	68	1.65 (1.26, 2.05)	60	1.35 (1.00, 1.70)	58	1.24 (0.92, 1.57)	60	1.23 (0.91, 1.54)	70	1.40 (1.07, 1.73)
Men	1,921	3.97 (3.79, 4.15)	2,357	4.82 (4.62, 5.01)	2,496	5.13 (4.92, 5.33)	2,695	5.48 (5.27, 5.69)	2,943	5.88 (5.67, 6.10)
Adults	146	0.44 (0.36, 0.51)	137	0.39 (0.33, 0.46)	155	0.44 (0.37, 0.52)	165	0.45 (0.38, 0.52)	188	0.51 (0.43, 0.58)
0–4 years old	1,519	67.8 (64.4, 71.2)	1,945	86.3 (82.4, 90.1)	2,083	93.2 (89.2, 97.2)	2,241	100.3 (96.2, 104.5)	2,412	106.3 (102.0, 110.5)
5–9 years old	136	4.52 (3.76, 5.29)	147	5.19 (4.34, 6.03)	132	4.92 (4.08, 5.76)	135	5.19 (4.31, 6.07)	159	6.61 (5.58, 7.64)
10–14 years old	58	1.69 (1.25, 2.13)	62	1.86 (1.39, 2.32)	62	1.90 (1.42, 2.37)	67	2.06 (1.56, 2.57)	89	2.85 (2.26, 3.44)
15–19 years old	62	2.05 (1.53, 2.56)	66	2.08 (1.57, 2.58)	64	1.98 (1.49, 2.47)	87	2.61 (2.06, 3.17)	95	2.80 (2.24, 3.37)
20–44 years old	90	0.43 (0.34, 0.52)	81	0.39 (0.31, 0.48)	86	0.42 (0.33, 0.51)	99	0.49 (0.40, 0.59)	105	0.53 (0.43, 0.63)
45–64 years old	38	0.33 (0.22, 0.43)	49	0.41 (0.29, 0.52)	52	0.41 (0.30, 0.53)	51	0.39 (0.28, 0.50)	64	0.48 (0.36, 0.60)
Over 65 years	18	0.44 (0.22, 0.64)	7	0.15 (0.03, 0.27)	17	0.36 (0.18, 0.54)	15	0.30 (0.14, 0.46)	19	0.38 (0.20, 0.55)

Cont. Suppl. Table 1-2.

Variables	2011		2012		2013		2014		2015	
	n	Prevalence (95% CI)	n	Prevalence (95% CI)	n	Prevalence (95% CI)	n	Prevalence (95% CI)	n	Prevalence (95% CI)
All	6,932	13.7 (13.3, 14.0)	7,343	14.4 (14.1, 14.7)	7,720	15.1 (14.8, 15.5)	7,726	15.1 (14.7, 15.4)	7,973	15.6 (15.3, 16.0)
Adults	903	2.43 (2.27, 2.59)	922	2.42 (2.26, 2.58)	994	2.53 (2.37, 2.69)	1,052	2.61 (2.45, 2.76)	1,108	2.68 (2.52, 2.84)
0–4 years old	5,268	228.9 (222.7, 235.1)	5,570	242.6 (236.2, 249.0)	5,849	256.6 (250.1, 263.2)	5,811	255.1 (248.5, 261.6)	5,916	262.9 (256.2, 269.6)
5–9 years old	397	17.3 (15.6, 19.0)	433	18.8 (17.0, 20.5)	498	21.8 (19.9, 23.7)	495	21.8 (19.9, 23.8)	570	24.8 (22.7, 26.8)
10–14 years old	185	6.12 (5.23, 7.07)	227	7.93 (6.90, 8.97)	207	7.60 (6.57, 8.64)	175	6.78 (5.77, 7.78)	204	8.56 (7.38, 9.73)
15–19 years old	179	5.29 (4.51, 6.07)	191	5.74 (4.92, 6.55)	172	5.23 (4.44, 6.02)	193	6.05 (5.20, 6.91)	175	5.61 (4.78, 6.44)
20–44 years old	431	2.20 (1.399, 2.41)	413	2.11 (1.91, 2.32)	443	2.29 (2.08, 2.51)	444	2.31 (2.10, 2.53)	461	2.43 (2.21, 2.65)
45–64 years old	372	2.73 (2.45, 3.01)	392	2.80 (2.52, 3.07)	423	2.92 (2.64, 3.20)	447	3.00 (2.72, 3.27)	470	3.06 (2.78, 3.34)
Over 65 years	100	1.91 (1.54, 2.29)	117	2.13 (1.74, 2.51)	128	2.22 (1.83, 2.60)	161	2.67 (2.26, 3.09)	177	2.83 (2.41, 3.25)
Women	3,812	7.58 (7.34, 7.82)	4,085	8.05 (7.80, 8.29)	4,290	8.44 (8.19, 8.70)	4,269	8.35 (8.10, 8.60)	4,381	8.60 (8.35, 8.86)
Adults	693	1.88 (1.74, 2.02)	710	1.87 (1.73, 2.01)	781	2.00 (1.85, 2.14)	804	2.00 (1.86, 2.14)	833	2.02 (1.88, 2.16)
0–4 years old	2,740	119.0 (114.6, 123.5)	2,919	127.1 (122.5, 131.7)	3,033	133.0 (128.3, 137.8)	3,022	132.6 (127.9, 137.4)	3,057	135.8 (131.0, 140.6)
5–9 years old	215	9.36 (8.11, 10.6)	245	10.6 (9.31, 11.9)	288	12.6 (11.1, 14.0)	277	12.2 (10.8, 13.6)	309	13.4 (11.9, 14.9)
10–14 years old	96	3.18 (2.54, 3.82)	142	4.96 (4.14, 5.78)	118	4.34 (3.55, 5.12)	102	3.92 (3.16, 4.69)	125	5.25 (4.33, 6.17)
15–19 years old	68	2.01 (1.53, 2.49)	69	2.05 (1.56, 2.54)	70	2.11 (1.61, 2.61)	64	1.98 (1.49, 2.48)	57	1.82 (1.35, 2.30)
20–44 years old	307	1.56 (1.38, 1.74)	302	1.55 (1.37, 1.72)	329	1.70 (1.51, 1.88)	305	1.59 (1.41, 1.77)	314	1.65 (1.47, 1.84)
45–64 years old	302	2.21 (1.96, 2.46)	313	2.23 (1.98, 2.48)	344	2.38 (2.12, 2.63)	363	2.43 (2.18, 2.68)	375	2.44 (2.22, 2.69)
Over 65 years	84	1.61 (1.26, 1.96)	95	1.73 (1.38, 2.08)	108	1.87 (1.51, 2.22)	136	2.25 (1.87, 2.63)	144	2.31 (1.93, 2.69)
Men	3,120	6.13 (5.91, 6.34)	3,258	6.37 (6.15, 6.59)	3,430	6.72 (6.49, 6.94)	3,457	6.74 (6.52, 6.97)	3,592	7.06 (6.83, 7.29)
Adults	210	0.55 (0.47, 0.62)	212	0.55 (0.47, 0.62)	213	0.53 (0.46, 0.60)	248	0.60 (0.53, 0.68)	275	0.66 (0.58, 0.74)
0–4 years old	2,528	109.8 (105.5, 114.1)	2,651	115.4 (111.0, 119.8)	2,816	123.6 (119.0, 128.1)	2,789	122.4 (117.8, 126.9)	2,859	127.0 (122.3, 131.7)
5–9 years old	182	7.90 (6.74, 9.05)	188	8.16 (6.99, 9.33)	210	9.18 (7.94, 10.4)	218	9.63 (8.35, 10.9)	261	11.3 (9.98, 12.7)
10–14 years old	89	2.93 (2.32, 3.55)	85	2.97 (2.34, 3.61)	89	3.26 (2.58, 3.94)	73	2.81 (2.16, 6.46)	79	3.30 (2.57, 4.04)
15–19 years old	111	3.28 (2.66, 3.89)	122	3.65 (3.00, 4.30)	102	3.09 (2.48, 3.69)	129	4.03 (3.33, 4.73)	118	3.78 (3.10, 4.46)
20–44 years old	124	0.63 (0.52, 0.74)	111	0.56 (0.46, 0.67)	114	0.58 (0.48, 0.69)	139	0.72 (0.60, 0.84)	147	0.77 (0.65, 0.90)
45–64 years old	70	0.51 (0.39, 0.63)	79	0.55 (0.43, 0.68)	79	0.54 (0.42, 0.66)	84	0.55 (0.43, 0.67)	95	0.61 (0.49, 0.74)
Over 65 years	16	0.30 (0.15, 0.45)	22	0.39 (0.22, 0.56)	20	0.33 (0.18, 0.48)	25	0.41 (0.24, 0.57)	33	0.51 (0.33, 0.699)

a Age-standardized prevalence rates of patent ductus arteries were calculated using age groups according to the direct method using the estimated Korean population in 2015 as a reference.

Supplementary table 1-3. Age-standardized prevalence[a] and 95% confidence interval (CI) of pulmonary artery stenosis overall and by sex (per 100,000).

Variables	2006		2007		2008		2009		2010	
	n	Prevalence (95% CI)	n	Prevalence (95% CI)	n	Prevalence (95% CI)	n	Prevalence (95% CI)	n	Prevalence (95% CI)
All	1,123	2.20 (2.07, 2.33)	1,828	3.60 (3.43, 3.77)	1,915	3.81 (3.63, 3.98)	2,046	4.02 (3.85, 4.20)	2,361	4.65 (4.45, 4.83)
Adults	133	0.41 (0.33, 0.48)	176	0.51 (0.43, 0.59)	191	0.55 (0.47, 0.63)	197	0.53 (0.45, 0.61)	243	0.65 (0.57, 0.73)
0–4 years old	563	25.1 (23.0, 27.2)	1,072	47.5 (44.7, 50.4)	1,124	50.3 (47.3, 53.2)	1,212	54.2 (51.2, 57.3)	1,422	62.6 (59.4, 65.9)
5–9 years old	210	7.01 (6.06, 7.96)	307	10.8 (9.65, 12.0)	323	12.0 (10.7, 13.3)	332	12.8 (11.4, 14.2)	334	13.9 (12.4, 15.4)
10–14 years old	151	4.46 (3.74, 5.18)	182	5.49 (4.69, 6.30)	162	5.00 (4.23, 5.77)	195	6.11 (5.25, 6.98)	193	6.16 (5.28, 7.03)
15–19 years old	66	2.17 (1.64, 2.70)	91	2.90 (2.30, 3.49)	115	3.56 (2.90, 4.21)	110	3.31 (2.68, 3.93)	169	5.01 (4.25, 5.77)
20–44 years old	78	0.38 (0.29, 0.46)	108	0.53 (0.43, 0.63)	113	0.56 (0.45, 0.66)	133	0.67 (0.55, 0.78)	157	0.79 (0.67, 0.92)
45–64 years old	36	0.31 (0.20, 0.41)	44	0.36 (0.25, 0.47)	48	0.38 (0.27, 0.49)	41	0.31 (0.22, 0.41)	59	0.44 (0.33, 0.56)
Over 65 years	19	0.45 (0.24, 0.66)	24	0.53 (0.31, 0.75)	30	0.63 (0.40, 0.87)	23	0.47 (0.27, 0.66)	27	0.53 (0.32, 0.73)
Women	538	1.06 (0.97, 1.15)	880	1.75 (1.63, 1.86)	950	1.90 (1.78, 2.02)	1,013	2.00 (1.88, 2.13)	1,172	2.31 (2.18, 2.45)
Adults	80	0.24 (0.19, 0.30)	85	0.25 (0.20, 0.31)	105	0.30 (0.24, 0.36)	108	0.30 (0.24, 0.35)	136	0.37 (0.30, 0.43)
0–4 years old	267	11.8 (10.4, 13.3)	550	24.3 (22.3, 26.4)	586	26.2 (24.0, 28.3)	628	28.1 (25.9, 30.3)	732	32.2 (29.9, 34.5)
5–9 years old	102	3.37 (2.71, 4.03)	137	4.83 (4.02, 5.65)	156	5.81 (4.90, 6.72)	149	5.72 (4.80, 6.65)	149	6.21 (5.21, 7.21)
10–14 years old	63	1.86 (1.39, 2.32)	78	2.35 (1.83, 2.88)	58	1.77 (1.31, 2.24)	88	2.72 (2.15, 3.30)	89	2.85 (2.26, 3.44)
15–19 years old	26	0.85 (0.52, 1.18)	30	0.94 (0.60, 1.28)	45	1.38 (0.97, 1.79)	40	1.19 (0.82, 1.57)	66	1.95 (1.48, 2.42)
20–44 years old	43	0.21 (0.14, 0.27)	44	0.21 (0.15, 0.28)	59	0.29 (0.21, 0.36)	67	0.33 (0.25, 0.41)	77	0.38 (0.30, 0.47)
45–64 years old	28	0.23 (0.14, 0.33)	26	0.21 (0.13, 0.30)	29	0.23 (0.14, 0.31)	24	0.18 (0.11, 0.26)	39	0.29 (0.19, 0.38)
Over 65 years	9	0.21 (0.06, 0.35)	15	0.33 (0.16, 0.50)	17	0.36 (0.18, 0.54)	17	0.35 (0.18, 0.51)	20	0.39 (0.21, 0.57)
Men	585	1.13 (1.04, 1.23)	948	1.85 (1.73, 1.96)	965	1.90 (1.78, 2.02)	1,033	2.01 (1.89, 2.14)	1,189	2.32 (2.19, 2.45)
Adults	53	0.15 (0.11, 0.20)	91	0.25 (0.20, 0.31)	86	0.24 (0.19, 0.29)	89	0.23 (0.18, 0.28)	107	0.27 (0.22, 0.33)
0–4 years old	296	13.1 (11.6, 14.7)	522	23.1 (21.1, 25.1)	538	24.0 (22.0, 26.1)	584	26.1 (24.0, 28.2)	690	30.4 (28.1, 32.6)
5–9 years old	108	3.59 (2.91, 4.27)	170	6.03 (5.12, 6.94)	167	6.21 (5.26, 7.16)	183	7.05 (6.03, 8.08)	185	7.72 (6.60, 8.83)
10–14 years old	88	2.60 (2.05, 3.15)	104	3.14 (2.53, 3.74)	104	3.18 (2.56, 3.80)	107	3.34 (2.71, 3.98)	104	3.30 (2.66, 3.94)
15–19 years old	40	1.29 (0.88, 1.70)	61	1.92 (1.43, 2.41)	70	2.17 (1.66, 2.68)	70	2.11 (1.61, 2.61)	103	3.02 (2.43, 3.61)
20–44 years old	35	0.17 (0.11, 0.22)	64	0.31 (0.23, 0.39)	54	0.26 (0.19, 0.33)	66	0.33 (0.25, 0.41)	80	0.40 (0.31, 0.49)
45–64 years old	8	0.06 (0.01, 0.11)	18	0.14 (0.07, 0.21)	19	0.15 (0.08, 0.22)	17	0.13 (0.06, 0.19)	20	0.14 (0.07, 0.21)
Over 65 years	10	0.24 (0.09, 0.39)	9	0.19 (0.06, 0.33)	13	0.27 (0.12, 0.42)	6	0.12 (0.02, 0.22)	7	0.13 (0.03, 0.24)

Cont. Suppl. Table 1-3.

Variables	2011		2012		2013		2014		2015	
	n	Prevalence (95% CI)	n	Prevalence (95% CI)	n	Prevalence (95% CI)	n	Prevalence (95% CI)	n	Prevalence (95% CI)
All	2,686	5.24 (5.04, 5.44)	2,967	5.77 (5.56, 5.98)	3,107	6.05 (5.84, 6.26)	3,237	6.31 (6.09, 6.52)	3,395	6.67 (6.45, 6.90)
Adults	255	0.68 (0.59, 0.76)	283	0.72 (0.63, 0.80)	294	0.73 (0.64, 0.81)	318	0.77 (0.68, 0.86)	312	0.74 (0.66, 0.83)
0–4 years old	1,666	72.3 (68.9, 75.8)	1,969	85.7 (81.9, 89.5)	2,006	88.0 (84.1, 91.8)	2,089	91.7 (87.7, 95.6)	2,211	98.2 (94.1, 102.3)
5–9 years old	351	15.3 (13.7, 16.9)	324	14.0 (12.5, 15.6)	379	16.6 (14.9, 18.2)	419	18.5 (16.7, 20.2)	428	18.6 (16.8, 20.4)
10–14 years old	224	7.44 (6.46, 8.41)	208	7.27 (6.28, 8.27)	236	8.68 (7.57, 9.79)	223	8.64 (7.50, 9.77)	231	9.67 (8.42, 10.9)
15–19 years old	190	5.64 (4.84, 6.44)	183	5.48 (4.69, 6.28)	192	5.86 (5.03, 6.69)	188	5.89 (5.05, 6.74)	213	6.84 (5.92, 7.76)
20–44 years old	146	0.74 (0.62, 0.86)	179	0.91 (0.78, 1.05)	192	0.99 (0.85, 1.13)	198	1.03 (0.88, 1.17)	199	1.04 (0.90, 1.19)
45–64 years old	72	0.52 (0.40, 0.64)	73	0.51 (0.39, 0.63)	75	0.51 (0.40, 0.63)	86	0.05 (0.45, 0.69)	73	0.47 (0.36, 0.58)
Over 65 years	37	0.70 (0.47, 0.93)	31	0.56 (0.36, 0.76)	27	0.45 (0.27, 0.63)	34	0.56 (0.37, 0.75)	40	0.63 (0.44, 0.83)
Women	1,406	2.75 (2.61, 2.90)	1,580	3.08 (2.93, 3.23)	1,602	3.12 (2.97, 3.28)	1,699	3.31 (3.15, 3.47)	1,741	3.42 (3.26, 3.58)
Adults	145	0.39 (0.33, 0.46)	161	0.41 (0.35, 0.48)	160	0.40 (0.33, 0.46)	169	0.41 (0.34, 0.47)	169	0.40 (0.34, 0.46)
0–4 years old	931	40.4 (37.8, 43.0)	1,120	48.7 (45.9, 51.6)	1,086	47.6 (44.8, 50.4)	1,147	50.3 (47.4, 53.2)	1,188	52.7 (49.7, 55.7)
5–9 years old	152	6.61 (5.55, 7.66)	137	5.94 (4.95, 6.94)	179	7.81 (6.66, 8.96)	210	9.27 (8.01, 10.5)	215	9.36 (8.11, 10.6)
10–14 years old	108	3.55 (2.87, 4.23)	98	3.43 (2.75, 4.11)	107	3.92 (3.18, 4.67)	98	3.76 (3.01, 4.51)	88	3.68 (2.90, 4.45)
15–19 years old	70	2.08 (1.59, 2.56)	64	1.89 (1.42, 2.36)	70	2.11 (1.61, 2.61)	75	2.33 (1.80, 2.86)	81	2.58 (2.01, 3.15)
20–44 years old	67	0.33 (0.25, 0.42)	92	0.47 (0.37, 0.56)	96	0.49 (0.39, 0.59)	100	0.52 (0.41, 0.62)	100	0.52 (0.42, 0.63)
45–64 years old	50	0.36 (0.26, 0.46)	46	0.32 (0.23, 0.42)	43	0.29 (0.20, 0.38)	47	0.31 (0.22, 0.40)	43	0.27 (0.19, 0.36)
Over 65 years	28	0.53 (0.33, 0.73)	23	0.41 (0.23, 0.58)	21	0.36 (0.20, 0.52)	22	0.36 (0.21, 0.51)	26	0.41 (0.25, 0.57)
Men	1,280	2.48 (2.34, 2.61)	1,387	2.69 (2.54, 2.83)	1,505	2.92 (2.77, 3.07)	1,538	2.99 (2.84, 3.14)	1,654	3.25 (3.09, 3.41)
Adults	110	0.28 (0.22, 0.33)	122	0.30 (0.25, 0.36)	134	0.32 (0.27, 0.38)	149	0.35 (0.30, 0.41)	143	0.34 (0.28, 0.39)
0–4 years old	735	31.9 (29.6, 34.2)	849	36.9 (34.4, 39.4)	920	40.3 (37.7, 42.9)	942	41.3 (38.6, 43.9)	1,023	45.4 (42.6, 48.2)
5–9 years old	199	8.65 (7.44, 9.86)	187	8.12 (6.95, 9.28)	200	8.74 (7.52, 9.95)	209	9.23 (7.97, 10.4)	213	9.27 (8.02, 10.5)
10–14 years old	116	3.84 (3.14, 4.54)	110	3.84 (3.12, 4.56)	129	4.71 (3.89, 5.53)	125	4.83 (3.98, 5.68)	143	5.99 (5.01, 6.98)
15–19 years old	120	3.56 (2.92, 4.20)	119	3.56 (2.92, 4.20)	122	3.72 (3.05, 4.38)	113	3.53 (2.87, 4.18)	132	4.22 (3.50, 4.95)
20–44 years old	79	0.39 (0.31, 0.48)	87	0.44 (0.34, 0.53)	96	0.49 (0.39, 0.59)	98	0.51 (0.40, 0.61)	99	0.52 (0.41, 0.62)
45–64 years old	22	0.15 (0.09, 0.22)	27	0.19 (0.12, 0.26)	32	0.21 (0.14, 0.29)	39	0.25 (0.17, 0.34)	30	0.19 (0.12, 0.26)
Over 65 years	9	0.16 (0.05, 0.28)	8	0.13 (0.03, 0.23)	6	0.09 (0.01, 0.17)	12	0.19 (0.08, 0.31)	14	0.21 (0.09, 0.33)

a Age-standardized prevalence rates of pulmonary artery stenosis were calculated using age groups according to the direct method using the estimated Korean population in 2015 as a reference.

Supplementary Table 1-4. Age-standardized prevalence[a] and 95% confidence interval (CI) of Coarctation of aorta overall and by sex (per 100,000).

Variables	2006		2007		2008		2009		2010	
	n	Prevalence (95% CI)	n	Prevalence (95% CI)	n	Prevalence (95% CI)	n	Prevalence (95% CI)	n	Prevalence (95% CI)
All	629	1.25 (1.15, 1.35)	740	1.46 (1.35, 1.57)	762	1.50 (1.39, 1.61)	868	1.69 (1.58, 1.80)	986	1.92 (1.80, 2.04)
Adults	91	0.27 (0.21, 0.33)	113	0.32 (0.26, 0.38)	115	0.32 (0.26, 0.38)	136	0.37 (0.30, 0.43)	163	0.43 (0.36, 0.50)
0–4 years old	330	14.7 (13.1, 16.3)	390	17.3 (15.5, 19.0)	401	17.9 (16.1, 19.6)	398	17.8 (16.0, 19.5)	431	18.9 (17.1, 20.7)
5–9 years old	97	3.24 (2.59, 3.88)	111	3.90 (3.17, 4.64)	109	4.03 (3.27, 4.80)	158	6.08 (5.12, 7.03)	199	8.30 (7.14, 9.45)
10–14 years old	71	2.10 (1.61, 2.60)	75	2.23 (1.71, 2.74)	84	2.56 (2.00, 3.11)	105	3.26 (2.63, 3.89)	108	3.43 (2.77, 4.08)
15–19 years old	40	1.29 (0.88, 1.70)	51	1.60 (1.16, 2.05)	53	1.64 (1.19, 2.08)	71	2.14 (1.64, 2.64)	85	2.52 (1.98, 3.05)
20–44 years old	58	0.28 (0.20, 0.35)	74	0.36 (0.28, 0.45)	80	0.39 (0.31, 0.48)	89	0.44 (0.35, 0.54)	107	0.54 (0.44, 0.64)
45–64 years old	21	0.17 (0.10, 0.25)	26	0.21 (0.13, 0.30)	20	0.15 (0.08, 0.23)	29	0.22 (0.14, 0.30)	38	0.28 (0.19, 0.37)
Over 65 years	12	0.28 (0.12, 0.45)	13	0.28 (0.12, 0.45)	15	0.31 (0.15, 0.48)	18	0.36 (0.19, 0.53)	18	0.35 (0.18, 0.51)
Women	264	0.52 (0.46, 0.59)	296	0.59 (0.52, 0.66)	311	0.61 (0.54, 0.68)	347	0.68 (0.61, 0.75)	378	0.74 (0.66, 0.81)
Adults	46	0.14 (0.09, 0.18)	57	0.17 (0.12, 0.21)	57	0.16 (0.11, 0.20)	64	0.18 (0.13, 0.22)	70	0.19 (0.14, 0.23)
0–4 years old	131	5.81 (4.81, 6.81)	153	6.75 (5.67, 7.83)	157	7.02 (5.92, 8.12)	158	7.06 (5.96, 8.17)	168	7.38 (6.26, 8.50)
5–9 years old	46	1.50 (1.06, 1.95)	47	1.64 (1.16, 2.12)	48	1.77 (1.26, 2.28)	66	2.53 (1.91, 3.14)	76	3.15 (2.43, 3.86)
10–14 years old	29	0.82 (0.51, 1.14)	26	0.78 (0.48, 1.08)	31	0.95 (0.61, 1.28)	36	1.11 (0.74, 1.48)	42	1.32 (0.91, 1.73)
15–19 years old	12	0.37 (0.15, 0.60)	13	0.41 (0.18, 0.63)	18	0.53 (0.27, 0.79)	23	0.69 (0.40, 0.97)	22	0.63 (0.35, 0.90)
20–44 years old	27	0.12 (0.07, 0.17)	33	0.16 (0.10, 0.21)	37	0.18 (0.12, 0.24)	37	0.18 (0.12, 0.24)	43	0.21 (0.15, 0.28)
45–64 years old	10	0.08 (0.03, 0.14)	13	0.10 (0.04, 0.16)	10	0.07 (0.02, 0.13)	13	0.09 (0.04, 0.15)	15	0.11 (0.05, 0.17)
Over 65 years	9	0.21 (0.06, 0.35)	11	0.24 (0.09, 0.39)	10	0.21 (0.07, 0.34)	14	0.28 (0.13, 0.44)	12	0.22 (0.09, 0.36)
Men	365	0.72 (0.64, 0.79)	444	0.87 (0.79, 0.95)	451	0.88 (0.80, 0.97)	521	1.00 (0.92, 1.09)	608	1.18 (1.08, 1.27)
Adults	45	0.12 (0.08, 0.16)	56	0.15 (0.11, 0.19)	58	0.15 (0.11, 0.19)	72	0.19 (0.14, 0.23)	93	0.24 (0.19, 0.29)
0–4 years old	199	8.85 (7.62, 10.0)	237	10.5 (9.17, 11.8)	244	10.9 (9.54, 12.2)	240	10.7 (9.37, 12.0)	263	11.5 (10.1, 12.9)
5–9 years old	51	1.68 (1.21, 2.15)	64	2.26 (1.70, 2.82)	61	2.26 (1.69, 2.85)	92	3.55 (2.82, 4.27)	123	5.10 (4.19, 6.01)
10–14 years old	42	1.24 (0.86, 1.61)	49	1.44 (1.03, 1.86)	53	1.61 (1.17, 2.05)	69	2.15 (1.63, 2.66)	66	2.10 (1.59, 2.61)
15–19 years old	28	0.91 (0.57, 1.25)	38	1.19 (0.81, 1.58)	35	1.07 (0.71, 1.43)	48	1.45 (1.03, 1.86)	63	1.86 (1.39, 2.32)
20–44 years old	31	0.14 (0.09, 0.20)	41	0.19 (0.13, 0.26)	43	0.21 (0.14, 0.27)	52	0.26 (0.18, 0.33)	64	0.32 (0.24, 0.40)
45–64 years old	11	0.09 (0.03, 0.15)	13	0.10 (0.04, 0.16)	10	0.07 (0.02, 0.13)	16	0.11 (0.05, 0.18)	23	0.17 (0.10, 0.24)
Over 65 years	3	0.06 (0.00, 0.14)	2	0.03 (0.00, 0.09)	5	0.10 (0.01, 0.20)	4	0.07 (0.00, 0.15)	6	0.10 (0.01, 0.20)

Cont. Suppl. Table 1-4.

Variables	2011		2012		2013		2014		2015	
	n	Prevalence (95% CI)	n	Prevalence (95% CI)	n	Prevalence (95% CI)	n	Prevalence (95% CI)	n	Prevalence (95% CI)
All	1,078	2.09 (1.96, 2.22)	1,182	2.29 (2.16, 2.42)	1,191	2.31 (2.17, 2.44)	1,222	2.37 (2.24, 2.51)	1,288	2.53 (2.39, 2.66)
Adults	182	0.47 (0.40, 0.54)	223	0.56 (0.49, 0.64)	217	0.53 (0.46, 0.61)	234	0.56 (0.49, 0.64)	246	0.58 (0.51, 0.66)
0–4 years old	447	19.4 (17.6, 21.2)	510	22.1 (20.2, 24.1)	457	20.0 (18.2, 21.8)	502	22.0 (20.0, 23.9)	513	22.7 (20.7, 24.7)
5–9 years old	234	10.2 (8.90, 11.5)	236	10.2 (8.94, 11.5)	247	10.8 (9.47, 12.1)	223	9.85 (8.55, 11.1)	234	10.1 (8.85, 11.4)
10–14 years old	114	3.76 (3.06, 4.45)	129	4.50 (3.72, 5.28)	153	5.62 (4.73, 6.51)	147	5.66 (4.74, 6.58)	169	7.07 (5.99, 8.14)
15–19 years old	101	2.99 (2.41, 3.58)	84	2.52 (1.98, 3.06)	117	3.56 (2.91, 4.21)	116	3.62 (2.96, 4.29)	126	4.03 (3.33, 4.74)
20–44 years old	120	0.61 (0.50, 0.72)	144	0.73 (0.61, 0.85)	143	0.73 (0.61, 0.85)	152	0.79 (0.66, 0.91)	167	0.88 (0.74, 1.01)
45–64 years old	45	0.32 (0.22, 0.42)	54	0.38 (0.28, 0.48)	50	0.34 (0.24, 0.44)	58	0.38 (0.28, 0.48)	58	0.37 (0.27, 0.46)
Over 65 years	17	0.31 (0.16, 0.47)	25	0.45 (0.27, 0.63)	24	0.41 (0.24, 0.57)	24	0.39 (0.23, 0.55)	21	0.33 (0.19, 0.47)
Women	433	0.84 (0.76, 0.92)	476	0.92 (0.84, 1.00)	462	0.89 (0.81, 0.97)	480	0.93 (0.84, 1.01)	533	1.04 (0.95, 1.13)
Adults	75	0.19 (0.15, 0.24)	100	0.25 (0.20, 0.30)	85	0.21 (0.16, 0.25)	101	0.24 (0.19, 0.29)	108	0.25 (0.20, 0.30)
0–4 years old	186	8.05 (6.89, 9.21)	206	8.94 (7.72, 10.1)	182	7.96 (6.80, 9.12)	202	8.85 (7.63, 10.0)	222	9.84 (8.54, 11.1)
5–9 years old	91	3.95 (3.13, 4.76)	88	3.81 (3.01, 4.61)	88	3.81 (3.01, 4.62)	82	3.59 (2.80, 4.38)	88	3.81 (3.01, 4.61)
10–14 years old	48	1.57 (1.11, 2.02)	51	1.77 (1.28, 2.26)	68	2.48 (1.88, 3.07)	65	2.48 (1.86, 3.09)	74	3.10 (2.39, 3.81)
15–19 years old	33	0.97 (0.64, 1.31)	31	0.91 (0.58, 1.24)	39	1.16 (0.79, 1.54)	30	0.91 (0.57, 1.25)	41	1.29 (0.88, 1.69)
20–44 years old	46	0.23 (0.16, 0.30)	60	0.30 (0.22, 0.38)	53	0.27 (0.19, 0.34)	61	0.31 (0.23, 0.39)	68	0.35 (0.26, 0.44)
45–64 years old	17	0.11 (0.06, 0.17)	24	0.16 (0.09, 0.23)	20	0.13 (0.07, 0.19)	27	0.17 (0.11, 0.24)	29	0.18 (0.11, 0.25)
Over 65 years	12	0.22 (0.09, 0.35)	16	0.28 (0.14, 0.43)	12	0.19 (0.07, 0.31)	13	0.21 (0.09, 0.33)	11	0.16 (0.06, 0.27)
Men	645	1.24 (1.15, 1.34)	706	1.36 (1.26, 1.46)	729	1.41 (1.31, 1.51)	742	1.44 (1.33, 1.54)	755	1.48 (1.37, 1.58)
Adults	107	0.27 (0.22, 0.32)	123	0.30 (0.25, 0.36)	132	0.32 (0.27, 0.38)	133	0.32 (0.26, 0.37)	138	0.32 (0.27, 038)
0–4 years old	261	11.3 (9.94, 12.6)	304	13.2 (11.7, 14.7)	275	12.0 (10.6, 13.4)	300	13.1 (11.6, 14.6)	291	12.9 (11.4, 14.4)
5–9 years old	143	6.21 (5.19, 7.23)	148	6.43 (5.39, 7.47)	159	6.96 (5.88, 8.05)	141	6.21 (5.18, 7.24)	146	6.34 (5.31, 7.38)
10–14 years old	66	2.19 (1.66, 2.72)	78	2.72 (2.12, 3.33)	85	3.10 (2.43, 3.76)	82	3.14 (2.45, 3.83)	95	3.96 (3.16, 4.77)
15–19 years old	68	2.01 (1.53, 2.49)	53	1.57 (1.14, 2.00)	78	2.36 (1.83, 2.89)	86	2.68 (2.11, 3.25)	85	2.71 (2.13, 3.29)
20–44 years old	74	0.37 (0.29, 0.46)	84	0.42 (0.33, 0.51)	90	0.46 (0.36, 0.56)	91	0.47 (0.37, 0.56)	99	0.52 (0.41, 0.62)
45–64 years old	28	0.19 (0.12, 0.27)	30	0.21 (0.13, 0.28)	30	0.20 (0.13, 0.28)	31	0.20 (0.13, 0.27)	29	0.18 (0.11, 0.25)
Over 65 years	5	0.09 (0.01, 0.17)	9	0.15 (0.04, 0.25)	12	0.19 (0.09, 0.31)	11	0.18 (0.07, 0.29)	10	0.15 (0.05, 0.25)

a Age-standardized prevalence rates of Coarctation of aorta were calculated using age groups according to the direct method using the estimated Korean population in 2015 as a reference.

Supplementary Table 1-5. Age-standardized prevalence[a] and 95% confidence interval (CI) of pulmonary venous connection overall and by sex (per 100,000).

Variables	2006		2007		2008		2009		2010	
	n	Prevalence (95% CI)	n	Prevalence (95% CI)	n	Prevalence (95% CI)	n	Prevalence (95% CI)	n	Prevalence (95% CI)
All	162	0.30 (0.25, 0.35)	224	0.44 (0.38, 0.50)	253	0.50 (0.44, 0.56)	281	0.55 (0.49, 0.62)	348	0.68 (0.61, 0.75)
Adults	17	0.05 (0.02, 0.07)	26	0.07 (0.04, 0.10)	29	0.08 (0.05, 0.11)	27	0.07 (0.04, 0.10)	31	0.08 (0.05, 0.11)
0–4 years old	107	4.74 (3.83, 5.64)	138	6.08 (5.06, 7.10)	141	6.30 (5.26, 7.34)	166	7.42 (6.29, 8.55)	192	8.45 (7.25, 9.65)
5–9 years old	25	0.79 (0.47, 1.12)	35	1.24 (0.83, 1.65)	53	1.95 (1.42, 2.48)	49	1.86 (1.33, 2.39)	78	3.24 (2.51, 3.96)
10–14 years old	8	0.20 (0.04, 0.37)	14	0.41 (0.19, 0.63)	16	0.45 (0.21, 0.69)	20	0.62 (0.34, 0.89)	21	0.66 (0.37, 0.94)
15–19 years old	5	0.15 (0.12, 0.30)	11	0.34 (0.13, 0.55)	14	0.41 (0.18, 0.63)	19	0.56 (0.30, 0.82)	26	0.75 (0.46, 1.05)
20–44 years old	9	0.03 (0.01, 0.06)	14	0.06 (0.03, 0.10)	15	0.07 (0.03, 0.11)	13	0.06 (0.02, 0.09)	16	0.07 (0.03, 0.11)
45–64 years old	6	0.04 (0.01, 0.08)	10	0.07 (0.02, 0.13)	13	0.09 (0.04, 0.15)	11	0.08 (0.03, 0.13)	14	0.09 (0.04, 0.15)
Over 65 years	2	0.04 (0.00, 0.11)	2	0.03 (0.00, 0.09)	1	0.01 (0.00, 0.05)	3	0.06 (0.00, 0.13)	1	0.01 (0.00, 0.05)
Women	77	0.15 (0.11, 0.18)	109	0.21 (0.17, 0.25)	123	0.24 (0.19, 0.28)	134	0.26 (0.22, 0.31)	168	0.33 (0.28, 0.38)
Adults	10	0.02 (0.01, 0.04)	12	0.03 (0.01, 0.05)	19	0.05 (0.02, 0.07)	19	0.05 (0.02, 0.07)	18	0.04 (0.02, 0.07)
0–4 years old	45	1.96 (1.38, 2.55)	66	2.90 (2.20, 3.61)	61	2.72 (2.04, 3.41)	71	3.17 (2.43, 3.91)	85	3.71 (2.91, 4.50)
5–9 years old	14	0.44 (0.19, 0.68)	19	0.66 (0.36, 0.96)	29	1.06 (0.67, 1.45)	28	1.06 (0.66, 1.46)	45	1.86 (1.31, 2.41)
10–14 years old	6	0.16 (0.02, 0.30)	8	0.20 (0.03, 0.37)	9	0.24 (0.06, 0.43)	11	0.33 (0.12, 0.53)	9	0.24 (0.05, 0.43)
15–19 years old	2	0.06 (0.00, 0.15)	4	0.12 (0.01, 0.25)	5	0.12 (0.00, 0.26)	5	0.12 (0.00, 0.25)	11	0.31 (0.12, 0.50)
20–44 years old	6	0.02 (0.01, 0.05)	6	0.02 (0.01, 0.05)	9	0.04 (0.01, 0.07)	8	0.03 (0.01, 0.06)	9	0.04 (0.01, 0.07)
45–64 years old	4	0.03 (0.00, 0.06)	5	0.03 (0.01, 0.07)	10	0.07 (0.02, 0.13)	9	0.06 (0.02, 0.11)	8	0.05 (0.01, 0.10)
Over 65 years	0	0.00 (0.00, 0.00)	1	0.01 (0.00, 0.05)	0	0.00 (0.00, 0.00)	2	0.03 (0.00, 0.08)	1	0.01 (0.00, 0.050
Men	85	0.17 (0.13, 0.21)	115	0.23 (0.18, 0.27)	130	0.25 (0.21, 0.30)	147	0.29 (0.24, 0.33)	180	0.35 (0.30, 0.40)
Adults	7	0.02 (0.01, 0.04)	14	0.04 (0.01, 0.06)	10	0.02 (0.01, 0.04)	8	0.02 (0.01, 0.03)	13	0.03 (0.01, 0.05)
0–4 years old	62	2.72 (2.03, 3.41)	72	3.17 (2.43, 3.91)	80	3.57 (2.79, 4.36)	95	4.24 (3.39, 5.10)	107	4.69 (3.80, 5.59)
5–9 years old	11	0.35 (0.13, 0.57)	16	0.53 (0.25, 0.81)	24	0.88 (0.52, 1.24)	21	0.79 (0.45, 1.14)	33	1.37 (0.90, 1.84)
10–14 years old	2	0.04 (0.00, 0.12)	6	0.16 (0.01, 0.31)	7	0.20 (0.04, 0.36)	9	0.24 (0.06, 0.43)	12	0.37 (0.15, 0.58)
15–19 years old	3	0.09 (0.00, 0.20)	7	0.22 (0.05, 0.38)	9	0.25 (0.06, 0.43)	14	0.41 (0.18, 0.63)	15	0.44 (0.21, 0.66)
20–44 years old	3	0.01 (0.00, 0.02)	8	0.03 (0.01, 0.06)	6	0.02 (0.00, 0.05)	5	0.02 (0.01, 0.04)	7	0.03 (0.01, 0.05)
45–64 years old	2	0.01 (0.00, 0.03)	5	0.03 (0.01, 0.07)	3	0.01 (0.00, 0.04)	2	0.01 (0.00, 0.03)	6	0.03 (0.01, 0.07)
Over 65 years	2	0.04 (0.00, 0.11)	1	0.01 (0.00, 0.05)	1	0.01 (0.00, 0.05)	1	0.01 (0.00, 0.05)	0	0.00 (0.00, 0.00)

Cont. Suppl. Table 1-5.

Variables	2011		2012		2013		2014		2015	
	n	Prevalence (95% CI)	n	Prevalence (95% CI)	n	Prevalence (95% CI)	n	Prevalence (95% CI)	n	Prevalence (95% CI)
All	392	0.76 (0.68, 0.83)	411	0.79 (0.72, 0.87)	429	0.83 (0.75, 0.91)	476	0.92 (0.84, 1.01)	482	0.94 (0.86, 1.03)
Adults	41	0.10 (0.07, 0.14)	47	0.12 (0.08, 0.15)	53	0.13 (0.09, 0.17)	69	0.16 (0.12, 0.20)	68	0.16 (0.12, 0.20)
0–4 years old	199	8.63 (7.43, 9.83)	207	8.99 (7.76, 10.2)	211	9.26 (8.01, 10.5)	231	10.1 (8.80, 11.4)	235	10.4 (9.08, 11.7)
5–9 years old	87	3.77 (2.97, 4.57)	94	4.08 (3.25, 4.91)	101	4.39 (3.53, 5.25)	105	4.61 (3.72, 5.50)	97	4.21 (3.37, 5.05)
10–14 years old	43	1.40 (0.97, 1.83)	42	1.44 (1.00, 1.89)	42	1.52 (1.06, 1.99)	42	1.61 (1.12, 2.10)	50	2.06 (1.48, 2.65)
15–19 years old	22	0.63 (0.35, 0.90)	21	0.63 (0.36, 0.90)	22	0.66 (0.38, 0.94)	29	0.88 (0.55, 1.21)	32	1.00 (0.65, 1.36)
20–44 years old	20	0.09 (0.05, 0.14)	20	0.09 (0.05, 0.14)	23	0.11 (0.06, 0.16)	36	0.18 (0.12, 0.24)	37	0.19 (0.13, 0.25)
45–64 years old	18	0.12 (0.06, 0.18)	22	0.15 (0.08, 0.21)	25	0.17 (0.10, 0.24)	27	0.17 (0.11, 0.24)	25	0.15 (0.09, 0.22)
Over 65 years	3	0.04 (0.00, 0.11)	5	0.09 (0.01, 0.17)	5	0.07 (0.00, 0.15)	6	0.09 (0.01, 0.17)	6	0.09 (0.01, 0.16)
Women	184	0.35 (0.30, 0.40)	191	0.37 (0.31, 0.42)	203	0.39 (0.33, 0.44)	220	0.42 (0.37, 0.48)	223	0.43 (0.37, 0.49)
Adults	24	0.06 (0.03, 0.08)	29	0.07 (0.04, 0.10)	31	0.07 (0.05, 0.10)	39	0.09 (0.06, 0.12)	41	0.09 (0.06, 0.12)
0–4 years old	88	3.80 (3.00, 4.60)	83	3.57 (2.80, 4.35)	89	3.89 (3.08, 4.70)	97	4.24 (3.40, 5.09)	94	4.16 (3.31, 5.00)
5–9 years old	38	1.64 (1.11, 2.16)	45	1.95 (1.38, 2.52)	49	2.13 (1.52, 2.73)	47	2.04 (1.44, 2.63)	48	2.08 (1.49, 2.67)
10–14 years old	25	0.82 (0.50, 1.15)	23	0.78 (0.45, 1.11)	24	0.86 (0.51, 1.22)	23	0.86 (0.50, 1.23)	25	1.03 (0.62, 1.44)
15–19 years old	9	0.25 (0.07, 0.42)	11	0.31 (0.12, 0.51)	10	0.28 (0.09, 0.47)	14	0.41 (0.17, 0.64)	15	0.47 (0.22, 0.71)
20–44 years old	12	0.06 (0.02, 0.09)	11	0.05 (0.02, 0.08)	13	0.06 (0.02, 0.10)	20	0.09 (0.05, 0.14)	23	0.11 (0.06, 0.16)
45–64 years old	10	0.07 (0.02, 0.11)	15	0.10 (0.05, 0.16)	16	0.10 (0.05, 0.16)	17	0.11 (0.05, 0.16)	16	0.09 (0.04, 0.15)
Over 65 years	2	0.03 (0.00, 0.08)	3	0.04 (0.00, 0.10)	2	0.03 (0.00, 0.07)	2	0.03 (0.00, 0.07)	2	0.03 (0.00, 0.07)
Men	208	0.40 (0.35, 0.46)	220	0.42 (0.37, 0.48)	226	0.43 (0.37, 0.49)	256	0.49 (0.43, 0.56)	259	0.50 (0.44, 0.57)
Adults	17	0.04 (0.02, 0.06)	18	0.04 (0.02, 0.06)	22	0.05 (0.03, 0.07)	30	0.07 (0.04, 0.09)	27	0.06 (0.03, 0.08)
0–4 years old	111	4.78 (3.88, 5.68)	124	5.36 (4.41, 6.31)	122	5.32 (4.37, 6.27)	134	5.86 (4.86, 6.85)	141	6.26 (5.22, 7.29)
5–9 years old	49	2.13 (1.53, 2.72)	49	2.13 (1.53, 2.72)	52	2.26 (1.64, 2.88)	58	2.53 (1.86, 3.19)	49	2.13 (1.53, 2.72)
10–14 years old	18	0.57 (0.30, 0.85)	19	0.66 (0.36, 0.96)	18	0.66 (0.35, 0.96)	19	0.70 (0.37, 1.03)	25	1.03 (0.62, 1.44)
15–19 years old	13	0.37 (0.16, 0.58)	10	0.28 (0.09, 0.47)	12	0.34 (0.13, 0.55)	15	0.44 (0.20, 0.68)	17	0.53 (0.27, 0.79)
20–44 years old	8	0.03 (0.01, 0.06)	9	0.04 (0.01, 0.07)	10	0.04 (0.01, 0.08)	16	0.08 (0.04, 0.12)	14	0.07 (0.03, 0.11)
45–64 years old	8	0.05 (0.01, 0.09)	7	0.04 (0.01, 0.08)	9	0.05 (0.01, 0.10)	10	0.06 (0.02, 0.10)	9	0.05 (0.01, 0.09)
Over 65 years	1	0.01 (0.00, 0.05)	2	0.03 (0.00, 0.08)	3	0.04 (0.00, 0.10)	4	0.06 (0.00, 0.12)	4	0.06 (0.00, 0.12)

a Age-standardized prevalence rates of pulmonary venous connection were calculated using age groups according to the direct method using the estimated Korean population in 2015 as a reference.

Supplementary Table 1-6. Age-standardized prevalence[a] and 95% confidence interval (CI) of congenital tricuspid stenosis overall and by sex (per 100,000).

Variables	2006		2007		2008		2009		2010	
	n	Prevalence (95% CI)	n	Prevalence (95% CI)	n	Prevalence (95% CI)	n	Prevalence (95% CI)	n	Prevalence (95% CI)
All	328	0.64 (0.57, 0.72)	369	0.72 (0.65, 0.80)	402	0.79 (0.71, 0.87)	334	0.66 (0.59, 0.73)	308	0.59 (0.53, 0.66)
Adults	82	0.25 (0.19, 0.30)	72	0.21 (0.16, 0.26)	75	0.20 (0.16, 0.25)	78	0.21 (0.16, 0.26)	66	0.18 (0.13, 0.22)
0–4 years old	114	5.05 (4.11, 5.99)	161	7.11 (6.00, 8.21)	198	8.85 (7.62, 10.0)	143	6.39 (5.34, 7.44)	102	4.47 (3.60, 5.34)
5–9 years old	67	2.21 (1.68, 2.75)	62	2.17 (1.62, 2.72)	59	2.17 (1.61, 2.73)	46	1.77 (1.26, 2.28)	44	1.81 (1.27, 2.36)
10–14 years old	44	1.28 (0.89, 1.66)	53	1.57 (1.13, 2.00)	41	1.24 (0.85, 1.62)	33	1.03 (0.67, 1.38)	45	1.40 (0.98, 1.82)
15–19 years old	21	0.69 (0.39, 0.99)	21	0.66 (0.37, 0.94)	29	0.88 (0.55, 1.21)	34	1.00 (0.66, 1.35)	51	1.51 (1.09, 1.92)
20–44 years old	46	0.22 (0.15, 0.28)	41	0.19 (0.13, 0.26)	48	0.23 (0.17, 0.30)	44	0.22 (0.15, 0.28)	39	0.19 (0.13, 0.25)
45–64 years old	29	0.25 (0.16, 0.34)	21	0.17 (0.09, 0.24)	22	0.17 (0.10, 0.25)	20	0.15 (0.08, 0.22)	16	0.11 (0.06, 0.17)
Over 65 years	7	0.16 (0.04, 0.29)	10	0.21 (0.07, 0.35)	5	0.10 (0.01, 0.20)	14	0.28 (0.13, 0.44)	11	0.21 (0.08, 0.34)
Women	148	0.30 (0.25, 0.35)	168	0.33 (0.28, 0.39)	179	0.35 (0.30, 0.41)	171	0.34 (0.29, 0.40)	140	0.27 (0.22, 0.32)
Adults	49	0.15 (0.11, 0.20)	41	0.12 (0.08, 0.16)	42	0.11 (0.08, 0.15)	51	0.15 (0.10, 0.19)	37	0.10 (0.06, 0.13)
0–4 years old	50	2.19 (1.57, 2.81)	70	3.08 (2.35, 3.81)	81	3.62 (2.83, 4.41)	70	3.13 (2.39, 3.86)	49	2.14 (1.54, 2.75)
5–9 years old	29	0.93 (0.57, 1.28)	28	0.97 (0.60, 1.34)	25	0.93 (0.56, 1.29)	22	0.84 (0.48, 1.19)	16	0.66 (0.33, 0.99)
10–14 years old	14	0.41 (0.19, 0.63)	19	0.53 (0.27, 0.79)	17	0.49 (0.24, 0.74)	12	0.37 (0.15, 0.58)	16	0.49 (0.24, 0.74)
15–19 years old	6	0.18 (0.30, 0.34)	10	0.31 (0.11, 0.51)	14	0.41 (0.18, 0.63)	16	0.47 (0.23, 0.71)	22	0.63 (0.35, 0.90)
20–44 years old	23	0.11 (0.06, 0.15)	19	0.09 (0.05, 0.13)	26	0.12 (0.07, 0.17)	25	0.12 (0.07, 0.17)	20	0.09 (0.05, 0.14)
45–64 years old	21	0.17 (0.10, 0.25)	14	0.11 (0.05, 0.17)	13	0.09 (0.04, 0.15)	12	0.09 (0.03, 0.14)	9	0.06 (0.02, 0.11)
Over 65 years	5	0.12 (0.01, 0.22)	8	0.16 (0.04, 0.29)	3	0.06 (0.00, 0.13)	14	0.28 (0.13, 0.44)	8	0.15 (0.04, 0.26)
Men	180	0.34 (0.29, 0.39)	201	0.38 (0.33, 0.44)	223	0.44 (0.38, 0.49)	163	0.31 (0.26, 0.36)	168	0.32 (0.27, 0.37)
Adults	33	0.09 (0.06, 0.12)	31	0.08 (0.05, 0.11)	33	0.08 (0.05, 0.12)	27	0.06 (0.04, 0.09)	29	0.07 (0.04, 0.10)
0–4 years old	64	2.81 (2.11, 3.51)	91	4.02 (3.19, 4.85)	117	5.23 (4.28, 6.18)	73	3.26 (2.51, 4.01)	53	2.32 (1.69, 2.95)
5–9 years old	38	1.24 (0.83, 1.64)	34	1.19 (0.79, 1.60)	34	1.24 (0.81, 1.66)	24	0.88 (0.51, 1.25)	28	1.15 (0.72, 1.58)
10–14 years old	30	0.86 (0.54, 1.18)	34	0.99 (0.64, 1.33)	24	0.70 (0.40, 1.00)	21	0.62 (0.33, 0.90)	29	0.90 (0.57, 1.24)
15–19 years old	15	0.47 (0.22, 0.72)	11	0.34 (0.13, 0.55)	15	0.44 (0.20, 0.67)	18	0.53 (0.28, 0.78)	29	0.85 (0.53, 1.16)
20–44 years old	23	0.11 (0.06, 0.15)	22	0.10 (0.05, 0.15)	22	0.10 (0.05, 0.15)	19	0.09 (0.05, 0.13)	19	0.09 (0.05, 0.13)
45–64 years old	8	0.06 (0.01, 0.11)	7	0.05 (0.01, 0.09)	9	0.07 (0.02, 0.12)	8	0.05 (0.01, 0.10)	7	0.04 (0.01, 0.08)
Over 65 years	2	0.04 (0.00, 0.11)	2	0.03 (0.00, 0.09)	2	0.03 (0.00, 0.09)	0	0.00 (0.00, 0.00)	3	0.04 (0.00, 0.11)

Cont. Suppl. Table 1-6.

Variables	2011		2012		2013		2014		2015	
	n	Prevalence (95% CI)	n	Prevalence (95% CI)	n	Prevalence (95% CI)	n	Prevalence (95% CI)	n	Prevalence (95% CI)
All	287	0.54 (0.48, 0.61)	310	0.59 (0.52, 0.66)	293	0.56 (0.50, 0.63)	298	0.57 (0.51, 0.64)	320	0.62 (0.55, 0.69)
Adults	65	0.16 (0.12, 0.20)	68	0.17 (0.13, 0.21)	72	0.17 (0.13, 0.21)	83	0.20 (0.15, 0.24)	88	0.21 (0.16, 0.25)
0–4 years old	91	3.93 (3.12, 4.74)	101	4.38 (3.52, 5.24)	104	4.56 (3.68, 5.44)	87	3.80 (2.99, 4.60)	115	5.09 (4.16, 6.03)
5–9 years old	37	1.59 (1.07, 2.11)	41	1.77 (1.22, 2.32)	39	1.68 (1.14, 2.22)	38	1.64 (1.10, 2.17)	32	1.37 (0.89, 1.85)
10–14 years old	48	1.57 (1.11, 2.02)	51	1.77 (1.28, 2.26)	43	1.57 (1.09, 2.04)	45	1.73 (1.22, 2.24)	34	1.40 (0.92, 1.88)
15–19 years old	46	1.35 (0.96, 1.75)	49	1.45 (1.03, 1.86)	35	1.04 (0.68, 1.39)	45	1.38 (0.97, 1.80)	51	1.60 (1.15, 2.05)
20–44 years old	47	0.23 (0.16, 0.30)	47	0.23 (0.16, 0.30)	49	0.24 (0.17, 0.32)	55	0.28 (0.20, 0.35)	63	0.33 (0.25, 0.41)
45–64 years old	13	0.09 (0.04, 0.14)	12	0.07 (0.03, 0.12)	13	0.08 (0.03, 0.13)	17	0.11 (0.05, 0.16)	17	0.10 (0.05, 0.15)
Over 65 years	5	0.09 (0.01, 0.17)	9	0.15 (0.04, 0.25)	10	0.16 (0.05, 0.27)	11	0.18 (0.07, 0.29)	8	0.12 (0.03, 0.12)
Women	130	0.24 (0.20, 0.29)	134	0.25 (0.21, 0.30)	131	0.25 (0.21, 0.29)	129	0.24 (0.20, 0.29)	141	0.27 (0.22, 0.32)
Adults	37	0.09 (0.06, 0.12)	37	0.09 (0.06, 0.12)	35	0.08 (0.05, 0.11)	39	0.09 (0.06, 0.12)	39	0.09 (0.06, 0.12)
0–4 years old	40	1.69 (1.16, 2.23)	45	1.92 (1.35, 2.49)	43	1.87 (1.31, 2.44)	35	1.52 (1.01, 2.03)	52	2.28 (1.65, 2.90)
5–9 years old	17	0.71 (0.35, 1.06)	19	0.79 (0.42, 1.17)	23	0.97 (0.56, 1.38)	21	0.88 (0.49, 1.28)	17	0.71 (0.35, 1.06)
10–14 years old	18	0.57 (0.30, 0.85)	16	0.53 (0.26, 0.81)	13	0.45 (0.19, 0.71)	18	0.66 (0.33, 0.98)	13	0.53 (0.24, 0.83)
15–19 years old	18	0.50 (0.25, 0.75)	17	0.50 (0.26, 0.74)	17	0.50 (0.25, 0.75)	16	0.47 (0.22, 0.71)	20	0.63 (0.34, 0.91)
20–44 years old	26	0.12 (0.07, 0.17)	25	0.12 (0.07, 0.17)	23	0.11 (0.06, 0.16)	26	0.13 (0.08, 0.18)	24	0.12 (0.07, 0.17)
45–64 years old	7	0.04 (0.01, 0.08)	6	0.03 (0.01, 0.07)	8	0.05 (0.01, 0.09)	10	0.06 (0.02, 0.10)	10	0.05 (0.01, 0.10)
Over 65 years	4	0.07 (0.01, 0.15)	6	0.10 (0.01, 0.19)	4	0.06 (0.00, 0.12)	3	0.04 (0.00, 0.10)	5	0.07 (0.01, 0.14)
Men	157	0.29 (0.25, 0.34)	176	0.33 (0.28, 038)	162	0.31 (0.26, 0.36)	169	0.32 (0.27, 0.37)	179	0.35 (0.29, 0.40)
Adults	28	0.07 (0.04, 0.09)	31	0.07 (0.04, 0.10)	37	0.09 (0.06, 0.12)	44	0.10 (0.07, 0.13)	49	0.11 (0.08, 0.14)
0–4 years old	51	2.19 (1.58, 2.80)	56	2.41 (1.77, 3.05)	61	2.63 (1.96, 3.31)	52	2.28 (1.66, 2.90)	63	2.77 (2.08, 3.46)
5–9 years old	20	0.84 (0.46, 1.22)	22	0.93 (0.53, 1.33)	16	0.66 (0.32, 1.00)	17	0.71 (0.35, 1.06)	15	0.62 (0.29, 0.95)
10–14 years old	30	0.99 (0.63, 1.34)	35	1.19 (0.79, 1.60)	30	1.07 (0.67, 1.47)	27	1.03 (0.63, 1.42)	21	0.86 (0.49, 1.24)
15–19 years old	28	0.82 (0.51, 1.12)	32	0.94 (0.61, 1.27)	18	0.53 (0.28, 0.79)	29	0.88 (0.55, 1.21)	31	0.97 (0.62, 1.32)
20–44 years old	21	0.10 (0.05, 0.15)	22	0.11 (0.06, 0.15)	26	0.13 (0.08, 0.18)	29	0.14 (0.09, 0.20)	39	0.20 (0.14, 0.26)
45–64 years old	6	0.03 (0.01, 0.07)	6	0.03 (0.01, 0.07)	5	0.03 (0.01, 0.06)	7	0.04 (0.01, 0.08)	7	0.03 (0.01, 0.07)
Over 65 years	1	0.01 (0.00, 0.05)	3	0.04 (0.00, 0.10)	6	0.09 (0.01, 0.17)	8	0.12 (0.02, 0.21)	3	0.04 (0.00, 0.10)

a Age-standardized prevalence rates of congenital tricuspid stenosis were calculated using age groups according to the direct method using the estimated Korean population in 2015 as a reference.

Supplementary Table 1-7. Age-standardized prevalence[a] and 95% confidence interval (CI) of congenital stenosis of aortic valve overall and by sex (per 100,000).

Variables	2006		2007		2008		2009		2010	
	n	Prevalence (95% CI)	n	Prevalence (95% CI)	n	Prevalence (95% CI)	n	Prevalence (95% CI)	n	Prevalence (95% CI)
All	296	0.63 (0.56, 0.71)	334	0.70 (0.62, 0.77)	365	0.75 (0.68, 0.83)	416	0.84 (0.76, 0.93)	466	0.95 (0.86, 1.04)
Adults	97	0.34 (0.27, 0.41)	116	0.39 (0.32, 0.47)	133	0.42 (0.34, 0.49)	142	0.43 (0.36, 0.51)	160	0.47 (0.40, 0.55)
0–4 years old	88	3.89 (3.07, 4.71)	82	3.62 (2.83, 4.41)	96	4.29 (3.43, 5.15)	105	4.69 (3.79, 5.59)	133	5.86 (4.86, 6.85)
5–9 years old	44	1.46 (1.03, 1.89)	61	2.13 (1.58, 2.67)	66	2.44 (1.84, 3.03)	74	2.84 (2.18, 3.49)	81	3.37 (2.63, 4.11)
10–14 years old	38	1.11 (0.75, 1.47)	53	1.57 (1.13, 2.00)	44	1.32 (0.92, 1.72)	64	1.98 (1.49, 2.47)	45	1.40 (0.98, 1.82)
15–19 years old	29	0.94 (0.59, 1.29)	22	0.69 (0.40, 0.98)	26	0.78 (0.47, 1.09)	31	0.91 (0.58, 1.24)	47	1.38 (0.98, 1.78)
20–44 years old	33	0.16 (0.10, 0.21)	37	0.18 (0.12, 0.24)	52	0.25 (0.18, 0.32)	50	0.24 (0.17, 0.31)	57	0.28 (0.21, 0.36)
45–64 years old	30	0.25 (0.16, 0.35)	32	0.26 (0.17, 0.35)	34	0.27 (0.17, 0.36)	34	0.26 (0.17, 0.35)	46	0.34 (0.24, 0.44)
Over 65 years	34	0.82 (0.54, 1.10)	47	1.06 (0.75, 1.37)	47	1.01 (0.72, 1.31)	58	1.18 (0.87, 1.49)	57	1.14 (0.84, 1.43)
Women	115	0.25 (0.20, 0.30)	141	0.30 (0.25, 0.36)	141	0.29 (0.24, 0.34)	159	0.33 (0.27, 0.38)	183	0.38 (0.32, 0.43)
Adults	50	0.17 (0.12, 0.22)	65	0.22 (0.16, 0.28)	57	0.18 (0.13, 0.23)	60	0.19 (0.14, 0.23)	75	0.22 (0.17, 0.27)
0–4 years old	30	1.29 (0.81, 1.77)	30	1.29 (0.82, 1.77)	35	1.56 (1.04, 2.08)	38	1.69 (1.15, 2.24)	45	1.96 (1.38, 2.54)
5–9 years old	18	0.57 (0.29, 0.85)	22	0.75 (0.42, 1.08)	23	0.84 (0.49, 1.19)	26	0.97 (0.59, 1.36)	28	1.15 (0.72, 1.58)
10–14 years old	12	0.33 (0.12, 0.53)	21	0.62 (0.34, 0.89)	21	0.62 (0.34, 0.89)	25	0.78 (0.47, 1.09)	21	0.66 (0.37, 0.94)
15–19 years old	5	0.15 (0.01, 0.30)	3	0.09 (0.00, 0.20)	5	0.12 (0.00, 0.26)	10	0.28 (0.09, 0.47)	14	0.41 (0.19, 0.62)
20–44 years old	17	0.08 (0.04, 0.12)	18	0.08 (0.04, 0.13)	19	0.09 (0.05, 0.13)	18	0.08 (0.04, 0.13)	23	0.11 (0.06, 0.16)
45–64 years old	15	0.12 (0.05, 0.19)	18	0.14 (0.07, 0.21)	14	0.11 (0.05, 0.17)	10	0.07 (0.02, 0.12)	20	0.14 (0.07, 0.21)
Over 65 years	18	0.44 (0.23, 0.64)	29	0.65 (0.41, 0.89)	24	0.51 (0.30, 0.72)	32	0.65 (0.42, 0.88)	32	0.63 (0.41, 0.86)
Men	181	0.38 (0.32, 0.43)	193	0.39 (0.33, 0.44)	224	0.46 (0.39, 0.52)	257	0.51 (0.45, 0.58)	283	0.57 (0.50, 0.63)
Adults	47	0.16 (0.11, 0.21)	51	0.16 (0.12, 0.49)	76	0.23 (0.17, 0.28)	82	0.24 (0.19, 0.30)	85	0.24 (0.19, 0.29)
0–4 years old	58	2.54 (1.88, 3.21)	52	2.28 (1.65, 2.90)	61	2.72 (2.04, 3.41)	67	2.99 (2.227, 3.71)	88	3.84 (3.03, 4.65)
5–9 years old	26	0.84 (0.50, 1.17)	39	1.37 (0.94, 1.81)	43	1.59 (1.11, 2.07)	48	1.81 (1.29, 2.34)	53	2.17 (1.57, 2.77)
10–14 years old	26	0.74 (0.44, 1.04)	32	0.95 (0.61, 1.28)	23	0.70 (0.41, 0.99)	39	1.19 (0.81, 1.58)	24	0.74 (0.43, 1.05)
15–19 years old	24	0.78 (0.47, 1.10)	19	0.59 (0.32, 0.87)	21	0.63 (0.35, 0.91)	21	0.63 (0.35, 0.90)	33	0.97 (0.64, 1.31)
20–44 years old	16	0.07 (0.03, 0.11)	19	0.09 (0.05, 0.13)	33	0.16 (0.10, 0.21)	32	0.16 (0.10, 0.21)	34	0.17 (0.11, 0.23)
45–64 years old	15	0.12 (0.05, 0.19)	14	0.11 (0.05, 0.17)	20	0.15 (0.08, 0.23)	24	0.18 (0.11, 0.26)	26	0.19 (0.11, 0.26)
Over 65 years	16	0.38 (0.18, 0.57)	18	0.39 (0.20, 0.58)	23	0.48 (0.28, 0.69)	26	0.53 (0.32, 0.73)	25	0.48 (0.29, 0.68)

Cont. Suppl. Table 1-7.

Variables	2011		2012		2013		2014		2015	
	n	Prevalence (95% CI)	n	Prevalence (95% CI)	n	Prevalence (95% CI)	n	Prevalence (95% CI)	n	Prevalence (95% CI)
All	499	1.00 (0.91, 1.09)	554	1.11 (1.02, 1.21)	555	1.09 (1.00, 1.18)	641	1.26 (1.16, 1.35)	617	1.21 (1.12, 1.31)
Adults	161	0.46 (0.38, 0.53)	212	0.59 (0.51, 0.67)	189	0.49 (0.42, 0.57)	216	0.54 (0.47, 0.62)	204	0.50 (0.43, 0.57)
0–4 years old	141	6.12 (5.11, 7.14)	136	5.90 (4.90, 6.90)	136	5.94 (4.94, 6.95)	153	6.71 (5.64, 7.77)	144	6.39 (5.35, 7.44)
5–9 years old	91	3.95 (3.13, 4.76)	105	4.52 (3.65, 5.40)	106	4.61 (3.73, 5.50)	124	5.45 (4.49, 6.42)	127	5.50 (4.54, 6.46)
10–14 years old	59	1.94 (1.44, 2.44)	54	1.86 (1.35, 2.36)	77	2.81 (2.17, 3.44)	93	3.59 (2.86, 4.33)	92	3.84 (3.05, 4.63)
15–19 years old	47	1.38 (0.98, 1.78)	47	1.38 (0.98, 1.79)	47	1.41 (1.00, 1.83)	55	1.70 (1.24, 2.15)	50	1.57 (1.13, 2.02)
20–44 years old	52	0.26 (0.18, 0.33)	65	0.33 (0.25, 0.41)	65	0.33 (0.25, 0.41)	73	0.37 (0.28, 0.46)	73	0.38 (0.29, 0.47)
45–64 years old	53	0.38 (0.28, 0.49)	51	0.35 (0.25, 0.45)	50	0.34 (0.24, 0.44)	62	0.41 (0.30, 0.51)	60	0.38 (0.28, 0.48)
Over 65 years	56	1.06 (0.78, 1.34)	96	1.75 (1.39, 2.10)	74	1.27 (0.98, 1.57)	81	1.33 (1.04, 1.63)	71	1.12 (0.86, 1.39)
Women	176	0.35 (0.30, 0.40)	206	0.41 (0.36, 0.47)	196	0.39 (0.33, 0.44)	241	0.47 (0.41, 0.53)	222	0.43 (0.37, 0.49)
Adults	65	0.18 (0.13, 0.23)	89	0.24 (0.19, 0.30)	73	0.19 (0.14, 0.23)	101	0.25 (0.20, 0.30)	81	0.20 (0.15, 0.24)
0–4 years old	42	1.78 (1.23, 2.34)	44	1.87 (1.31, 2.44)	44	1.92 (1.35, 2.49)	46	2.01 (1.42, 2.59)	47	2.05 (1.46, 2.65)
5–9 years old	32	1.37 (0.89, 1.85)	34	1.46 (0.96, 1.96)	35	1.50 (1.00, 2.01)	41	1.77 (1.21, 2.33)	43	1.86 (1.30, 2.42)
10–14 years old	22	0.70 (0.39, 1.00)	24	0.82 (0.48, 1.16)	26	0.95 (0.58, 1.31)	33	1.24 (0.80, 1.67)	35	1.44 (0.95, 1.93)
15–19 years old	15	0.44 (0.21, 0.66)	15	0.44 (0.21, 0.66)	18	0.53 (0.28, 0.79)	20	0.59 (0.32, 0.87)	16	0.50 (0.25, 0.75)
20–44 years old	22	0.11 (0.06, 0.15)	24	0.12 (0.07, 0.17)	22	0.11 (0.06, 0.15)	31	0.16 (0.10, 0.21)	25	0.12 (0.07, 0.17)
45–64 years old	20	0.14 (0.08, 0.21)	21	0.14 (0.08, 0.21)	21	0.13 (0.07, 0.20)	26	0.17 (0.10, 0.24)	26	0.16 (0.10, 0.23)
Over 65 years	23	0.44 (0.26, 0.62)	44	0.79 (0.55, 1.02)	30	0.51 (0.33, 0.70)	44	0.73 (0.51, 0.94)	30	0.47 (0.29, 0.64)
Men	323	0.64 (0.57, 0.72)	348	0.69 (0.62, 0.77)	359	0.70 (0.63, 0.78)	400	0.78 (0.70, 0.86)	395	0.77 (0.70, 0.85)
Adults	96	0.27 (0.21, 0.32)	123	0.33 (0.27, 0.40)	116	0.30 (0.24, 0.36)	115	0.28 (0.23, 0.34)	123	0.30 (0.24, 0.35)
0–4 years old	99	4.29 (3.44, 5.14)	92	3.98 (3.16, 4.80)	92	4.02 (3.20, 4.85)	107	4.69 (3.08, 5.58)	97	4.29 (3.43, 5.15)
5–9 years old	59	2.57 (1.91, 3.23)	71	3.06 (2.34, 3.78)	71	3.10 (2.38, 3.83)	83	3.63 (2.84, 4.43)	84	3.63 (2.85, 4.42)
10–14 years old	37	1.19 (0.80, 1.59)	30	1.03 (0.65, 1.41)	51	1.86 (1.34, 2.37)	60	2.31 (1.72, 2.90)	57	2.35 (1.73, 2.97)
15–19 years old	32	0.94 (0.61, 1.27)	32	0.94 (0.61, 1.27)	29	0.88 (0.56, 1.20)	35	1.07 (0.70, 1.43)	34	1.07 (0.70, 1.43)
20–44 years old	30	0.14 (0.09, 0.20)	41	0.21 (0.14, 0.27)	43	0.22 (0.15, 0.28)	42	0.21 (0.14, 0.28)	48	0.24 (0.17, 0.32)
45–64 years old	33	0.23 (0.15, 0.32)	30	0.21 (0.13, 0.28)	29	0.19 (0.12, 0.27)	36	0.23 (0.16, 0.31)	34	0.21 (0.14, 0.29)
Over 65 years	33	0.62 (0.40, 0.84)	52	0.94 (0.68, 1.20)	44	0.76 (0.53, 0.98)	37	0.60 (0.41, 0.80)	41	0.65 (0.45, 0.85)

a Age-standardized prevalence rates of congenital stenosis of aortic valve were calculated using age groups according to the direct method using the estimated Korean population in 2015 as a reference.

Supplementary Table 1-8. Age-standardized prevalence[a] and 95% confidence interval (CI) of congenital insufficiency of aortic valve overall and by sex (per 100,000).

Variables	2006		2007		2008		2009		2010	
	n	Prevalence (95% CI)	n	Prevalence (95% CI)	n	Prevalence (95% CI)	n	Prevalence (95% CI)	n	Prevalence (95% CI)
All	439	1.01 (0.91, 1.10)	553	1.27 (1.16, 1.38)	676	1.52 (1.41, 1.64)	811	1.78 (1.66, 1.91)	1,100	2.40 (2.25, 2.54)
Adults	272	0.91 (0.80, 1.03)	385	1.26 (1.13, 1.39)	475	1.50 (1.36, 1.64)	589	1.79 (1.64, 1.93)	819	2.42 (2.25, 2.59)
0–4 years old	35	1.52 (1.00, 2.03)	37	1.61 (1.08, 2.13)	50	2.23 (1.61, 2.85)	64	2.86 (2.16, 3.56)	87	3.80 (2.99, 4.60)
5–9 years old	37	1.19 (0.79, 1.59)	51	1.77 (1.27, 2.27)	49	1.81 (1.30, 2.33)	46	1.77 (1.26, 2.28)	61	2.53 (1.89, 3.16)
10–14 years old	38	1.11 (0.75, 1.47)	43	1.28 (0.89, 1.67)	44	1.32 (0.92, 1.72)	53	1.65 (1.20, 2.10)	58	1.81 (1.34, 2.29)
15–19 years old	57	1.86 (1.37, 2.35)	37	1.16 (0.78, 1.54)	58	1.79 (1.33, 2.26)	59	1.76 (1.31, 2.22)	75	2.20 (1.70, 2.71)
20–44 years old	100	0.48 (0.39, 0.58)	126	0.62 (0.51. 0.73)	157	0.78 (0.65, 0.90)	195	0.98 (0.84, 1.12)	253	1.28 (1.12, 1.44)
45–64 years old	124	1.08 (0.89, 1.27)	172	1.44 (1.23, 1.66)	207	1.68 (1.45, 1.91)	256	2.01 (1.76, 2.26)	357	2.70 (2.42, 2.98)
Over 65 years	48	1.17 (0.83, 1.50)	87	1.97 (1.56, 2.39)	111	2.40 (1.95, 2.85)	138	2.84 (2.36, 3.32)	209	4.18 (3.61, 4.75)
Women	167	0.39 (0.33, 0.45)	210	0.49 (0.43, 0.56)	252	0.59 (0.51, 0.66)	291	0.66 (0.58, 0.73)	400	0.90 (0.81, 0.99)
Adults	104	0.36 (0.29, 0.44)	147	0.50 (0.42, 0.59)	188	0.61 (0.52, 0.70)	215	0.67 (0.58, 0.76)	312	0.95)0.84, 1.06)
0–4 years old	14	0.58 (0.25, 0.90)	16	0.67 (0.32, 1.01)	21	0.93 (0.53, 1.34)	24	1.07 (0.64, 1.50)	28	1.20 (0.75, 1.66)
5–9 years old	13	0.39 (0.16, 0.63)	17	0.57 (0.28, 0.86)	15	0.53 (0.24, 0.81)	17	0.62 (0.30, 0.93)	22	0.88 (0.50, 1.27)
10–14 years old	20	0.57 (0.31, 0.83)	18	0.53 (0.28, 0.78)	14	0.41 (0.18, 0.64)	14	0.41 (0.18, 0.64)	19	0.57 (0.30, 0.82)
15–19 years old	16	0.50 (0.24, 0.76)	12	0.37 (0.16, 0.59)	14	0.41 (0.18, 0.63)	21	0.63 (0.35, 0.90)	19	0.53 (0.28, 0.78)
20–44 years old	28	0.13 (0.08, 0.18)	36	0.17 (0.11, 0.23)	48	0.23 (0.17, 0.30)	52	0.26 (0.18, 0.33)	71	0.36 (0.27, 0.44)
45–64 years old	49	0.42 (0.30, 0.54)	62	0.51 (0.38, 0.64)	79	0.64 (0.50, 0.78)	91	0.71 (0.56, 0.85)	129	0.97 (0.80, 1.14)
Over 65 years	27	0.65 (0.40, 0.90)	49	1.11 (0.79, 1.42)	61	1.32 (0.99, 1.65)	72	1.47 (1.13, 1.82)	112	2.23 (1.82, 2.65)
Men	272	0.61 (0.53, 0.68)	343	0.77 (0.68, 0.85)	424	0.93 (0.84, 1.02)	520	1.12 (1.02, 1.22)	700	1.49 (1.38, 1.61)
Adults	168	0.54 (0.46, 0.63)	238	0.76 (0.66, 0.86)	287	0.88 (0.78, 0.99)	374	1.11 (0.99, 1.22)	507	1.46 (1.34, 1.59)
0–4 years old	21	0.89 (0.49, 1.29)	21	0.89 (0.49, 1.29)	29	1.29 (0.82, 1.76)	40	1.78 (1.23, 2.34)	59	2.59 (1.93, 3.25)
5–9 years old	24	0.79 (0.47, 1.12)	34	1.19 (0.79, 1.60)	34	1.24 (0.81, 1.66)	29	1.10 (0.70, 1.51)	39	1.59 (1.08, 2.10)
10–14 years old	18	0.49 (0.24, 0.74)	25	0.74 (0.44, 1.04)	30	0.90 (0.57, 1.24)	39	1.19 (0.81, 1.58)	39	1.24 (0.84, 1.63)
15–19 years old	41	1.35 (0.94, 1.77)	25	0.78 (0.47, 1.10)	44	1.35 (0.95, 1.76)	38	1.13 (0.76, 1.50)	56	1.64 (1.20, 2.07)
20–44 years old	72	0.34 (0.26, 0.43)	90	0.44 (0.35, 0.53)	109	0.54 (0.44, 0.64)	143	0.72 (0.60, 0.83)	182	0.92 (0.79, 1.06)
45–64 years old	75	0.65 (0.50, 0.80)	110	0.92 (0.75, 1.09)	128	1.04 (0.86, 1.22)	165	1.29 (1.09, 1.49)	228	1.72 (1.50, 1.95)
Over 65 years	21	0.50 (0.28, 0.72)	38	0.85 (0.57, 1.12)	50	1.08 (0.77, 1.38)	66	1.35 (1.02, 1.68)	97	1.93 (1.54, 2.32)

Cont. Suppl. Table 1-8.

Variables	2011		2012		2013		2014		2015	
	n	Prevalence (95% CI)	n	Prevalence (95% CI)	n	Prevalence (95% CI)	n	Prevalence (95% CI)	n	Prevalence (95% CI)
All	1,480	3.15 (2.99, 3.32)	1,848	3.88 (3.70, 4.06)	2,188	4.48 (4.30, 4.67)	2,670	5.35 (5.14, 5.55)	3,196	6.30 (6.08, 6.51)
Adults	1,162	3.29 (3.10, 3.48)	1,502	4.11 (3.90, 4.32)	1,780	4.69 (4.47, 4.91)	2,189	5.55 (5.31, 5.78)	2,651	6.51 (6.26, 6.76)
0–4 years old	94	4.07 (3.24, 4.89)	94	4.07 (3.24, 4.89)	123	5.36 (4.41, 6.32)	147	6.44 (5.39, 7.48)	173	7.64 (6.50, 8.79)
5–9 years old	67	2.88 (2.18, 3.58)	90	3.90 (3.09, 4.71)	98	4.26 (3.41, 5.11)	126	5.54 (4.57, 6.52)	141	6.12 (5.11, 7.14)
10–14 years old	80	2.64 (2.06, 3.22)	73	2.56 (1.97, 3.15)	91	3.34 (2.66, 4.03)	94	3.63 (2.90, 4.37)	95	3.96 (3.16, 4.77)
15–19 years old	77	2.27 (1.75, 2.78)	89	2.64 (2.09, 3.20)	96	2.93 (2.34, 3.52)	114	3.56 (2.90, 4.22)	136	4.35 (3.16, 4.77)
20–44 years old	353	1.80 (1.61, 1.99)	406	2.08 (1.88, 2.28)	452	2.34 (2.12, 2.55)	549	2.86 (2.62, 3.10)	631	3.33 (3.07, 3.59)
45–64 years old	537	3.94 (3.61, 4.27)	723	5.16 (4.79, 5.54)	872	6.03 (5.63, 6.44)	1,059	7.10 (6.67, 7.53)	1,317	8.60 (8.13, 9.06)
Over 65 years	272	5.23 (4.61, 5.86)	373	6.81 (6.12, 7.51)	456	7.93 (7.20, 8.66)	581	9.66 (8.87, 10.4)	703	11.2 (10.4, 12.1)
Women	516	1.12 (1.02, 1.22)	614	1.31 (1.21, 1.41)	710	1.47 (1.36, 1.58)	879	1.77 (1.65, 1.89)	1,044	2.06 (1.94, 2.19)
Adults	402	1.16 (1.05, 1.28)	502	1.40 (1.28, 1.52)	576	1.54 (1.41, 1.67)	699	1.79 (1.65, 1.92)	858	2.12 (1.98, 2.26)
0–4 years old	34	1.47 (0.97, 1.97)	35	1.52 (1.01, 2.02)	50	2.19 (1.58, 2.80)	66	2.86 (2.16, 3.56)	64	2.81 (2.12, 3.51)
5–9 years old	30	1.28 (0.81, 1.75)	36	1.55 (1.04, 2.06)	33	1.42 (0.92, 1.92)	47	2.04 (1.44, 2.63)	49	2.13 (1.53, 2.72)
10–14 years old	23	0.74 (0.43, 1.05)	22	0.74 (0.42, 1.06)	32	1.15 (0.74, 1.56)	38	1.44 (0.97, 1.91)	40	1.65 (1.13, 2.17)
15–19 years old	27	0.78 (0.48, 1.09)	19	0.56 (0.31, 0.82)	19	0.56 (0.30, 0.82)	29	0.88 (0.55, 1.21)	33	1.04 (0.67, 1.40)
20–44 years old	96	0.48 (0.39, 0.58)	92	0.47 (0.37, 0.56)	100	0.51 (0.41, 0.61)	127	0.66 (0.54, 0.77)	157	0.82 (0.69, 0.95)
45–64 years old	173	1.27 (1.08, 1.45)	244	1.74 (1.52, 1.96)	267	1.84 (1.62, 2.07)	326	2.18 (1.95, 2.42)	404	2.63 (2.37, 2.89)
Over 65 years	133	2.55 (2.12, 2.99)	166	3.02 (2.56, 3.49)	209	3.63 (3.14, 4.13)	246	4.09 (3.58, 4.60)	297	4.76 (4.22, 5.30)
Men	964	2.03 (1.90, 2.16)	1,234	2.56 (2.42, 2.71)	1,478	3.01 (2.85, 3.16)	1,791	3.57 (3.40, 3.74)	2,152	4.23 (4.05, 4.40)
Adults	760	2.12 (1.97, 2.27)	1,000	2.70 (2.54, 2.87)	1,204	3.14 (2.96, 3.32)	1,490	3.75 (3.56, 3.94)	1,793	4.38 (4.18, 4.59)
0–4 years old	60	2.59 (1.93, 3.25)	59	2.54 (1.89, 3.20)	73	3.17 (2.44, 3.91)	81	3.53 (2.75, 4.30)	109	4.83 (3.92, 5.72)
5–9 years old	37	1.59 (1.07, 2.11)	54	2.30 (1.68, 2.93)	65	2.84 (2.14, 3.53)	79	3.46 (2.69, 4.23)	92	3.99 (3.17, 4.81)
10–14 years old	57	1.86 (1.36, 2.35)	51	1.77 (1.28, 2.26)	59	2.15 (1.59, 2.70)	56	2.15 (1.58, 2.71)	55	2.27 (1.66, 2.88)
15–19 years old	50	1.48 (1.07, 1.89)	70	2.08 (1.58, 2.57)	77	2.33 (1.80, 2.86)	85	2.64 (2.08, 3.21)	103	3.28 (2.64, 3.91)
20–44 years old	257	1.30 (1.14, 1.47)	314	1.61 (1.43, 1.79)	352	1.82 (1.63, 2.01)	422	2.20 (1.99, 2.41)	474	2.50 (2.27, 2.72)
45–64 years old	364	2.67 (2.39, 2.94)	479	3.41 (3.11, 3.72)	605	4.19 (3.85, 4.52)	733	4.91 (4.55, 5.27)	913	5.95 (5.57, 6.34)
Over 65 years	139	2.67 (2.23, 3.12)	207	3.77 (3.25, 4.29)	247	4.29 (3.75, 4.82)	335	5.57 (4.97, 6.16)	406	6.51 (5.88, 7.15)

a Age-standardized prevalence rates of congenital insufficiency of aortic valve were calculated using age groups according to the direct method using the estimated Korean population in 2015 as a reference.

Supplementary Table 1-9. Age-standardized prevalence[a] and 95% confidence interval (CI) of congenital mitral stenosis overall and by sex (per 100,000).

Variables	2006		2007		2008		2009		2010	
	n	Prevalence (95% CI)	n	Prevalence (95% CI)	n	Prevalence (95% CI)	n	Prevalence (95% CI)	n	Prevalence (95% CI)
All	877	1.73 (1.61, 1.85)	786	1.54 (1.43, 1.65)	809	1.58 (1.47, 1.69)	778	1.48 (1.38, 1.59)	824	1.57 (1.46, 1.67)
Adults	195	0.63 (0.54, 0.73)	155	0.48 (0.40, 0.56)	165	0.49 (0.41, 0.57)	128	0.36 (0.29, 0.42)	130	0.35 (0.29, 0.41)
0–4 years old	288	12.8 (11.3, 14.3)	249	11.0 (9.67, 12.4)	245	10.9 (9.58, 12.3)	209	9.34 (8.07, 10.6)	222	9.75 (8.46, 11.0)
5–9 years old	212	7.05 (6.10, 8.01)	187	6.61 (5.66, 7.56)	196	7.27 (6.25, 8.30)	203	7.81 (6.73, 8.89)	197	8.21 (7.06, 9.36)
10–14 years old	132	3.88 (3.21, 4.55)	121	3.63 (2.98, 4.29)	126	3.88 (3.20, 4.56)	133	4.17 (3.46, 4.88)	172	5.49 (4.67, 6.32)
15–19 years old	50	1.64 (1.18, 2.09)	74	2.33 (1.79, 2.87)	77	2.39 (1.86, 2.93)	105	3.15 (2.54, 3.76)	103	3.02 (2.43, 3.61)
20–44 years old	90	0.43 (0.34, 0.52)	72	0.35 (0.27, 0.43)	80	0.39 (0.31, 0.48)	72	0.36 (0.27, 0.44)	84	0.42 (0.33, 0.51)
45–64 years old	68	0.59 (0.45, 0.73)	52	0.43 (0.31, 0.55)	50	0.40 (0.29, 0.51)	38	0.29 (0.20, 0.39)	25	0.18 (0.11, 0.26)
Over 65 years	37	0.89 (060, 1.19)	31	0.70 (0.45, 0.94)	35	0.74 (0.49, 0.99)	18	0.36 (0.19, 0.53)	21	0.41 (0.23, 0.59)
Women	474	0.93 (0.85, 1.02)	439	0.85 (0.77, 0.94)	458	0.90 (0.81, 0.98)	467	0.89 (0.81, 0.97)	494	0.94 (0.85, 1.02)
Adults	109	0.35 (0.28, 0.42)	85	0.26 (0.21, 0.32)	103	0.31 (0.25, 0.37)	78	0.22 (0.17, 0.27)	71	0.19 (0.14, 0.24)
0–4 years old	141	6.26 (5.22, 7.30)	130	5.77 (4.77, 6.76)	130	5.81 (4.81, 6.81)	117	5.23 (4.28, 6.18)	135	5.94 (4.94, 6.95)
5–9 years old	119	3.95 (3.25, 4.66)	116	4.08 (3.33, 4.83)	114	4.21 (3.43, 4.99)	128	4.92 (4.06, 5.78)	119	4.97 (4.07, 5.86)
10–14 years old	73	2.15 (1.65, 2.64)	63	1.90 (1.43, 2.37)	69	2.10 (1.60, 2.61)	79	2.48 (1.93, 3.02)	107	3.39 (2.74, 4.04)
15–19 years old	32	1.04 (0.67, 1.40)	45	1.41 (0.99, 1.83)	42	1.29 (0.89, 1.68)	65	1.95 (1.47, 2.43)	62	1.82 (1.37, 2.28)
20–44 years old	48	0.23 (0.16, 0.29)	36	0.17 (0.11, 0.23)	47	0.23 (0.16, 0.30)	40	0.19 (0.13, 0.26)	41	0.20 (0.14, 0.26)
45–64 years old	41	0.35 (0.24, 0.46)	33	0.27 (0.17, 0.36)	33	0.26 (0.17, 0.35)	24	0.18 (0.11, 0.26)	17	0.12 (0.06, 0.18)
Over 65 years	20	0.48 (0.27, 0.70)	16	0.35 (0.17, 0.52)	23	0.48 (0.28, 0.69)	14	0.28 (0.13, 0.44)	13	0.25 (0.11, 0.40)
Men	403	0.79 (0.71, 0.87)	347	0.67 (0.60, 0.75)	351	0.68 (0.61, 0.75)	311	0.59 (0.52, 0.65)	330	0.62 (0.55, 0.69)
Adults	86	0.27 (0.21, 0.34)	70	0.21 (0.16, 0.26)	62	0.18 (0.13, 0.22)	50	0.13 (0.09, 0.17)	59	0.15 (0.11, 0.44)
0–4 years old	147	6.53 (5.46, 7.59)	119	5.27 (4.32, 6.22)	115	5.14 (4.20, 6.08)	92	4.11 (3.27, 4.95)	87	3.80 (2.99, 4.60)
5–9 years old	93	3.10 (2.47, 3.73)	71	2.48 (1.89, 3.07)	82	3.01 (2.35, 3.68)	75	2.88 (2.22, 3.54)	78	3.24 (2.51, 3.96)
10–14 years old	59	1.73 (1.28, 2.18)	58	1.73 (1.28, 2.18)	57	1.73 (1.27, 2.19)	54	1.69 (1.24, 2.14)	65	2.06 (1.56, 2.57)
15–19 years old	18	0.56 (0.29, 0.84)	29	0.91 (0.57, 1.25)	35	1.07 (0.71, 1.43)	40	1.19 (0.82, 1.57)	41	1.19 (0.82, 1.57)
20–44 years old	42	0.20 (0.14, 0.26)	36	0.17 (0.11, 0.23)	33	0.16 (0.10, 0.21)	32	0.16 (0.10, 0.21)	43	0.21 (0.15, 0.28)
45–64 years old	27	0.23 (0.14, 0.32)	19	0.15 (0.08, 0.23)	17	0.13 (0.06, 0.19)	14	0.10 (0.04, 0.16)	8	0.05 (0.01, 0.10)
Over 65 years	17	0.41 (0.21, 0.60)	15	0.33 (0.16, 0.50)	12	0.25 (0.11, 0.40)	4	0.07 (0.00, 0.15)	8	0.15 (0.04, 0.26)

Cont. Suppl. Table 1-9.

Variables	2011		2012		2013		2014		2015	
	n	Prevalence (95% CI)	n	Prevalence (95% CI)	n	Prevalence (95% CI)	n	Prevalence (95% CI)	n	Prevalence (95% CI)
All	799	1.52 (1.42, 1.63)	777	1.48 (1.38, 1.59)	802	1.54 (1.43, 1.65)	917	1.78 (1.66, 1.89)	911	1.79 (1.67, 1.90)
Adults	146	0.38 (0.31, 0.44)	129	0.32 (0.27, 0.38)	144	0.35 (0.30, 0.41)	217	0.52 (0.45, 0.60)	194	0.46 (0.39, 0.52)
0–4 years old	211	9.17 (7.93, 10.4)	194	8.41 (7.22, 9.59)	192	8.41 (7.21, 9.60)	207	9.08 (7.84, 10.3)	232	10.2 (8.96, 11.6)
5–9 years old	188	8.16 (6.99, 9.34)	179	7.76 (6.62, 8.90)	173	7.54 (6.41, 8.67)	181	7.98 (6.82, 9.15)	178	7.72 (6.58, 8.86)
10–14 years old	153	5.08 (4.27, 5.89)	157	5.49 (4.63, 6.36)	164	6.03 (5.11, 6.96)	175	6.78 (5.77, 7.78)	177	7.40 (6.30, 8.49)
15–19 years old	101	2.99 (2.41, 3.58)	118	3.53 (2.89, 4.17)	129	3.94 (3.26, 4.62)	137	4.28 (3.56, 5.01)	130	4.16 (3.44, 4.88)
20–44 years old	101	0.51 (0.41, 0.61)	83	0.42 (0.32, 0.51)	96	0.49 (0.39, 0.59)	141	0.73 (0.61, 0.85)	145	0.76 (0.64, 0.89)
45–64 years old	25	0.17 (0.10, 0.25)	26	0.17 (0.10, 0.25)	26	0.17 (0.11, 0.24)	44	0.29 (0.20, 0.37)	28	0.17 (0.11, 0.24)
Over 65 years	20	0.38 (0.21, 0.54)	20	0.36 (0.20, 0.52)	22	0.38 (0.22, 0.54)	32	0.53 (0.34, 0.71)	21	0.33 (0.19, 0.47)
Women	473	0.90 (0.82, 0.98)	478	0.91 (0.83, 0.99)	480	0.92 (0.83, 1.00)	559	1.08 (0.99, 1.17)	550	1.08 (0.99, 1.17)
Adults	86	0.22 (0.17, 0.27)	75	0.19 (0.14, 0.23)	82	0.20 (0.15, 0.24)	126	0.30 (0.25, 0.36)	114	0.27 (0.22, 0.32)
0–4 years old	119	5.14 (4.21, 6.07)	118	5.09 (4.17, 6.02)	111	4.83 (3.92, 5.73)	124	5.41 (4.45, 6.37)	129	5.72 (4.73, 6.71)
5–9 years old	113	4.92 (4.01, 5.83)	108	4.66 (3.77, 5.54)	109	4.74 (3.85, 5.64)	112	4.92 (4.00, 5.84)	115	4.97 (4.05, 5.88)
10–14 years old	95	3.14 (2.50, 3.77)	105	3.68 (2.97, 4.38)	106	3.88 (3.14, 4.63)	117	4.50 (3.68, 5.32)	111	4.63 (3.76, 5.49)
15–19 years old	60	1.76 (1.31, 2.21)	72	2.14 (1.64, 2.64)	72	2.17 (1.66, 2.68)	80	2.49 (1.94, 3.04)	81	2.58 (2.01, 3.15)
20–44 years old	54	0.27 (0.19, 0.34)	48	0.24 (0.17, 0.31)	55	0.28 (0.20, 0.35)	79	0.41 (0.31, 0.50)	85	0.44 (0.34, 0.53)
45–64 years old	17	0.11 (0.60, 0.17)	16	0.11 (0.05, 0.16)	17	0.11 (0.05, 0.16)	28	0.18 (0.11, 0.25)	18	0.11 (0.05, 0.16)
Over 65 years	15	0.28 (0.14, 0.43)	11	0.19 (0.07, 0.31)	10	0.16 (0.05, 0.27)	19	0.30 (0.16, 0.44)	11	0.16 (0.62, 0.27)
Men	326	0.62 (0.55, 0.68)	299	0.57 (0.50, 0.63)	322	0.62 (0.55, 0.69)	358	0.69 (0.62, 0.76)	361	0.70 (0.63, 0.78)
Adults	60	0.15 (0.11, 0.19)	54	0.13 (0.10, 0.17)	62	0.15 (0.11, 0.19)	91	0.21 (0.17, 0.26)	80	0.19 (0.14, 0.23)
0–4 years old	92	3.98 (3.16, 4.79)	76	3.31 (2.56, 4.05)	81	3.53 (2.75, 4.30)	83	3.62 (2.83, 4.40)	103	4.56 (3.67, 5.44)
5–9 years old	75	3.24 (2.49, 3.98)	71	3.06 (2.34, 3.78)	64	2.79 (2.10, 3.48)	69	3.01 (2.29, 3.73)	63	2.70 (2.02, 3.38)
10–14 years old	58	1.90 (1.40, 2.39)	52	1.81 (1.32, 2.31)	58	2.10 (1.55, 2.65)	58	2.23 (1.65, 2.81)	66	2.77 (2.10, 3.43)
15–19 years old	41	1.19 (0.82, 1.57)	46	1.35 (0.95, 1.75)	57	1.73 (1.28, 2.18)	57	1.76 (1.30, 2.23)	49	1.54 (1.10, 1.98)
20–44 years old	47	0.23 (0.16, 0.30)	35	0.17 (0.11, 0.23)	41	0.21 (0.14, 0.27)	62	0.32 (0.24, 0.40)	60	0.31 (0.23, 0.39)
45–64 years old	8	0.05 (0.01, 0.09)	10	0.06 (0.02, 0.11)	9	0.05 (0.01, 0.10)	16	0.10 (0.05, 0.15)	10	0.05 (0.01, 0.10)
Over 65 years	5	0.09 (0.01, 0.17)	9	0.15 (0.04, 0.25)	12	0.19 (0.07, 0.31)	13	0.21 (0.09, 0.33)	10	0.15 (0.05, 0.25)

a Age-standardized prevalence rates of congenital mitral stenosis were calculated using age groups according to the direct method using the estimated Korean population in 2015 as a reference.

Supplementary Table 1-10. Age-standardized prevalence[a] and 95% confidence interval (CI) of malformation of coronary vessels overall and by sex (per 100,000).

Variables	2006		2007		2008		2009		2010	
	n	Prevalence (95% CI)	n	Prevalence (95% CI)	n	Prevalence (95% CI)	n	Prevalence (95% CI)	n	Prevalence (95% CI)
All	5555	11.3 (11.0, 11.6)	6127	12.3 (12.0, 12.6)	5588	11.3 (11.0, 11.6)	4833	9.93 (9.65, 10.2)	4296	8.82 (8.55, 9.08)
Adults	765	2.58 (2.40, 2.77)	859	2.73 (2.54, 2.91)	917	2.85 (2.66, 3.04)	1186	3.61 (3.40, 3.82)	1349	4.01 (3.80, 4.23)
0–4 years old	3187	142.3 (137.4, 147.3)	3498	155.3 (150.1, 160.4)	3175	142.1 (137.2, 147.1)	2542	113.8 (109.4, 118.3)	1937	85.3 (81.5, 89.2)
5–9 years old	840	28.0 (16.1, 29.9)	883	31.3 (29.3, 33.4)	687	25.6 (23.7, 27.5)	487	18.8 (17.1, 20.4)	412	17.1 (15.5, 18.8)
10–14 years old	560	16.6 (15.2, 18.0)	594	17.9 (16.5, 19.4)	518	16.0 (14.6, 17.3)	411	12.9 (11.6, 14.1)	372	11.9 (10.6, 13.1)
15–19 years old	203	6.68 (5.76, 7.61)	293	9.33 (8.26, 10.4)	291	9.05 (8.01, 10.0)	207	6.24 (5.39, 7.09)	226	6.68 (5.81, 7.56)
20–44 years old	311	1.53 (1.36, 1.70)	369	1.83 (1.64, 2.01)	361	1.80 (1.61, 1.99)	387	1.95 (1.75, 2.14)	387	1.96 (1.77, 2.16)
45–64 years old	272	2.37 (2.09, 2.65)	316	2.66 (2.37, 2.96)	340	2.77 (2.47, 3.06)	507	3.99 (3.64, 4.33)	605	4.58 (4.22, 4.95)
Over 65 years	182	4.46 (3.81, 5.10)	174	3.95 (3.36, 4.54)	216	4.68 (4.06, 5.31)	292	6.04 (5.35, 6.73)	357	7.17 (6.42, 7.91)
Women	2725	5.66 (5.44, 5.87)	3082	6.26 (6.04, 6.49)	2761	5.67 (5.45, 5.88)	2410	4.99 (4.79, 5.19)	2121	4.38 (4.20, 4.57)
Adults	455	1.58 (1.43, 1.73)	478	1.55 (1.40, 1.69)	521	1.63 (1.48, 1.77)	639	1.95 (1.80, 2.11)	706	2.11 (1.95, 2.27)
0–4 years old	1583	70.7 (67.2, 74.2)	1776	78.8 (75.1, 82.4)	1585	70.9 (67.4, 74.4)	1278	57.2 (54.1, 60.4)	978	43.0 (40.3, 45.7)
5–9 years old	393	13.1 (11.8, 14.4)	416	14.7 (13.3, 16.2)	328	12.2 (10.8, 13.5)	239	9.23 (8.06, 10.4)	197	8.21 (7.06, 9.36)
10–14 years old	207	6.11 (5.28, 6.95)	277	8.35 (7.36, 9.34)	225	6.94 (6.03, 7.85)	167	5.25 (4.45, 6.04)	158	5.04 (4.25, 5.83)
15–19 years old	87	2.87 (2.26, 3.47)	135	4.28 (3.56, 5.01)	102	3.15 (2.53, 3.77)	87	2.61 (2.06, 3.17)	82	2.42 (1.90, 2.95)
20–44 years old	168	0.82 (0.70, 0.95)	191	0.94 (0.81, 1.08)	205	1.02 (0.88, 1.16)	211	1.06 (0.92, 1.20)	204	1.03 (0.89, 1.18)
45–64 years old	151	1.31 (1.10, 1.52)	167	1.41 (1.19, 1.62)	178	1.45 (1.23, 1.66)	248	1.94 (1.70, 2.19)	288	2.18 (1.92, 2.43)
Over 65 years	136	3.33 (2.77, 3.89)	120	2.72 (2.23, 3.21)	138	2.99 (2.49, 3.49)	180	3.71 (3.16, 4.25)	214	4.29 (3.71, 4.86)
Men	2830	5.64 (5.43, 5.85)	3045	6.07 (0.85, 6.29)	2827	5.71 (5.49, 5.92)	2423	4.94 (4.74, 5.13)	2175	4.43 (4.24, 4.61)
Adults	310	1.00 (0.89, 1.12)	381	1.18 (1.05, 1.30)	396	1.22 (1.10, 1.34)	547	1.65 (1.51, 1.79)	643	1.90 (1.75, 2.04)
0–4 years old	1604	71.6 (68.1, 75.1)	1722	76.4 (72.8, 80.0)	1590	71.1 (67.6, 74.6)	1264	56.6 (53.5, 59.7)	959	42.2 (39.5, 44.9)
5–9 years old	447	14.9 (13.5, 16.2)	467	16.6 (15.0, 18.1)	359	13.4 (12.0, 14.7)	248	9.54 (8.35, 10.7)	215	8.96 (7.76, 10.1)
10–14 years old	353	10.5 (9.40, 11.5)	317	9.59 (8.53, 10.6)	293	9.05 (8.01, 10.0)	244	7.64 (6.68, 8.61)	214	6.82 (5.90, 7.74)
15–19 years old	116	3.81 (3.11, 4.51)	158	5.01 (4.22, 5.80)	189	5.86 (5.02, 6.70)	120	3.62 (2.97, 4.27)	144	4.25 (3.55, 4.95)
20–44 years old	143	0.69 (0.58, 0.81)	178	0.88 (0.75, 1.01)	156	0.77 (0.65, 0.89)	176	0.88 (0.75, 1.01)	183	0.93 (0.79, 1.06)
45–64 years old	121	1.05 (0.86, 1.24)	149	1.25 (1.05, 1.45)	162	1.32 (1.11, 1.52)	259	2.03 (1.78, 2.28)	317	2.40 (2.13, 2.66)
Over 65 years	46	1.12 (0.80, 1.45)	54	1.21 (0.88, 1.54)	78	1.68 (1.31, 2.06)	112	2.31 (1.88, 2.74)	143	2.86 (2.39, 3.33)

Cont. Suppl. Table 1-10.

Variables	2011		2012		2013		2014		2015	
	n	Prevalence (95% CI)	n	Prevalence (95% CI)	n	Prevalence (95% CI)	n	Prevalence (95% CI)	n	Prevalence (95% CI)
All	4415	9.15 (8.88, 9.42)	5564	11.5 (11.2, 11.8)	5624	11.4 (11.1, 11.7)	5964	11.9 (11.6, 12.2)	5970	11.8 (11.5, 12.1)
Adults	1980	5.73 (5.48, 5.98)	3071	8.54 (8.24, 8.84)	3274	8.75 (8.45, 9.05)	3530	9.04 (8.74, 9.34)	3664	9.07 (8.77, 9.36)
0–4 years old	1628	70.7 (67.2, 74.1)	1753	76.3 (72.7, 79.9)	1596	70.0 (66.5, 73.4)	1678	73.6 (70.1, 77.1)	1649	73.2 (69.7, 76.8)
5–9 years old	307	13.3 (11.8, 14.8)	299	12.9 (11.4, 14.4)	271	11.8 (10.4, 13.2)	293	12.9 (11.4, 14.4)	253	11.0 (9.64, 12.3)
10–14 years old	293	9.71 (8.60, 10.8)	251	8.80 (7.71, 9.89)	264	9.71 (8.54, 10.8)	238	9.22 (8.04, 10.3)	189	7.93 (6.80, 9.07)
15–19 years old	207	6.15 (5.31, 6.98)	190	5.70 (4.89, 6.52)	219	6.68 (5.79, 7.57)	225	7.06 (6.14, 7.98)	215	6.90 (5.98, 7.83)
20–44 years old	441	2.25 (2.04, 2.46)	592	3.04 (2.79, 3.28)	574	2.97 (2.73, 3.21)	601	3.14 (2.88, 3.39)	567	2.99 (2.74, 3.24)
45–64 years old	974	7.15 (6.70, 7.60)	1598	11.4 (10.8, 11.9)	1682	11.6 (11.0, 12.2)	1779	11.9 (11.3, 12.4)	1863	12.1 (1.16, 12.7)
Over 65 years	565	10.8 (9.98, 11.7)	881	16.1 (15.0, 17.1)	1018	17.7 (16.6, 18.8)	1150	19.1 (18.0, 20.2)	1234	19.8 (18.7, 20.9)
Women	2095	4.37 (4.18, 4.56)	2589	5.38 (5.17, 5.59)	2647	5.42 (5.21, 5.63)	2803	5.62 (5.41, 5.83)	2782	5.52 (5.31, 5.72)
Adults	944	2.76 (2.58, 2.94)	1429	4.00 (3.79, 4.21)	1543	4.15 (3.94, 4.36)	1656	4.26 (4.05, 4.46)	1689	4.20 (4.00, 4.40)
0–4 years old	804	34.9 (32.5, 37.3)	842	36.6 (34.2, 39.1)	777	34.0 (31.6, 36.4)	815	35.7 (33.2, 38.1)	819	36.3 (33.8, 38.8)
5–9 years old	139	6.03 (5.02, 7.04)	131	5.68 (4.70, 6.65)	129	5.63 (4.66, 6.61)	135	5.94 (4.93, 6.95)	131	5.68 (4.70, 6.65)
10–14 years old	123	4.05 (3.32, 4.77)	112	3.92 (3.19, 4.65)	118	4.34 (3.55, 5.12)	117	4.50 (3.68, 5.32)	72	3.01 (2.31, 3.71)
15–19 years old	85	2.52 (1.98, 3.06)	75	2.23 (1.72, 2.74)	80	2.42 (1.89, 2.96)	80	2.49 (1.94, 3.04)	71	2.27 (1.74, 2.80)
20–44 years old	187	0.95 (0.81, 1.09)	256	1.31 (1.15, 1.47)	246	1.27 (1.11, 1.43)	250	1.30 (1.14, 1.46)	228	1.20 (1.04, 1.36)
45–64 years old	435	3.19 (2.89, 3.49)	692	4.94 (4.57, 5.31)	728	5.04 (4.67, 5.40)	796	5.34 (4.96, 5.71)	792	5.16 (4.80, 5.52)
Over 65 years	322	6.21 (5.53, 6.88)	481	8.78 (7.99, 9.56)	569	9.91 (9.09, 10.7)	610	10.1 (9.34, 10.9)	669	10.7 (9.93, 11.5)
Men	2320	4.77 (4.58, 4.97)	2975	6.13 (5.91, 6.35)	2977	6.04 (5.82, 6.26)	3161	6.31 (6.09, 6.53)	3188	6.30 (6.08, 6.51)
Adults	1036	2.96 (2.78, 3.14)	1642	4.53 (4.31, 4.75)	1731	4.59 (4.38, 4.81)	1874	4.78 (4.56, 4.99)	1975	4.86 (4.65, 5.08)
0–4 years old	824	35.7 (33.3, 38.2)	911	39.6 (37.1, 42.2)	819	35.9 (33.4, 38.3)	863	37.8 (35.3, 40.4)	830	36.8 (34.3, 39.3)
5–9 years old	168	7.32 (6.21, 8.43)	168	7.27 (6.17, 8.38)	142	6.21 (5.18, 7.23)	158	6.96 (5.87, 8.05)	122	5.28 (4.33, 6.22)
10–14 years old	170	5.62 (4.77, 6.47)	139	4.87 (4.06, 5.69)	146	5.37 (4.50, 6.24)	121	4.67 (3.83, 5.50)	117	4.87 (3.98, 5.77)
15–19 years old	122	3.62 (2.98, 4.27)	115	3.43 (2.80, 4.06)	139	4.22 (3.51, 4.93)	145	4.54 (3.80, 5.28)	144	4.60 (3.84, 5.36)
20–44 years old	254	1.29 (1.13, 1.45)	336	1.72 (1.54, 1.91)	328	1.69 (1.51, 1.88)	351	1.83 (1.63, 2.02)	339	1.78 (1.59, 1.97)
45–64 years old	539	3.95 (3.62, 4.29)	906	6.47 (6.04, 6.89)	954	6.60 (6.18, 7.02)	983	6.59 (6.18, 7.01)	1071	6.99 (6.57, 7.40)
Over 65 years	243	4.67 (4.08, 5.26)	400	7.30 (6.59, 8.02)	449	7.80 (7.08, 8.53)	540	8.98 (8.22, 9.74)	565	9.07 (8.32, 9.82)

a Age-standardized prevalence rates of malformation of coronary vessels were calculated using age groups according to the direct method using the estimated Korean population in 2015 as a reference.

Supplementary Table 1-11. Age-standardized prevalence[a] and 95% confidence interval (CI) of stenosis or malformation of aorta overall and by sex (per 100,000).

Variables	2006		2007		2008		2009		2010	
	n	Prevalence (95% CI)	n	Prevalence (95% CI)	n	Prevalence (95% CI)	n	Prevalence (95% CI)	n	Prevalence (95% CI)
All	554	1.27 (1.16, 1.38)	651	1.45 (1.33, 1.56)	686	1.54 (1.42, 1.65)	741	1.61 (1.49, 1.72)	1113	2.46 (2.31, 2.61)
Adults	272	0.96 (0.85, 1.08)	310	1.05 (0.93, 1.17)	364	1.20 (1.08, 1.33)	379	1.19 (1.07, 1.31)	685	2.13 (1.97, 2.29)
0–4 years old	161	7.15 (6.04, 8.26)	182	8.05 (6.87, 9.22)	159	7.11 (6.00, 8.21)	177	7.91 (6.74, 9.08)	213	9.34 (8.08, 10.6)
5–9 years old	58	1.90 (1.40, 2.40)	75	2.66 (2.05, 3.26)	67	2.48 (1.88, 3.08)	76	2.92 (2.26, 3.58)	93	3.86 (3.07, 4.65)
10–14 years old	39	1.15 (0.79, 1.52)	47	1.40 (0.99, 1.81)	51	1.57 (1.13, 2.00)	57	1.77 (1.31, 2.24)	70	2.23 (1.70, 2.75)
15–19 years old	24	0.78 (0.47, 1.10)	37	1.16 (0.78, 1.54)	45	1.38 (0.97, 1.79)	52	1.54 (1.11, 1.97)	52	1.51 (1.09, 1.93)
20–44 years old	85	0.41 (0.32, 0.50)	96	0.47 (0.38, 0.57)	98	0.48 (0.39, 0.58)	105	0.52 (0.42, 0.62)	130	0.66 (0.54, 0.77)
45–64 years old	99	0.86 (0.69, 1.03)	105	0.88 (0.71, 1.05)	122	0.99 (0.81, 1.16)	125	0.98 (0.81, 1.15)	258	1.95 (1.71, 2.19)
Over 65 years	88	2.14 (1.69, 2.59)	109	2.48 (2.01, 2.94)	144	3.12 (2.60, 3.63)	149	3.07 (2.57, 3.57)	297	5.95 (5.27, 6.63)
Women	213	0.49 (0.42, 0.56)	281	0.65 (0.57, 0.73)	302	0.70 (0.62, 0.78)	330	0.73 (0.65, 0.81)	539	1.22 (1.11, 1.32)
Adults	113	0.41 (0.33, 0.48)	164	0.56 (0.47, 0.65)	186	0.63 (0.54, 0.72)	187	0.60 (0.51, 0.68)	368	1.15 (1.03, 1.27)
0–4 years old	53	2.32 (1.68, 2.96)	65	2.86 (2.16, 3.56)	59	2.63 (1.96, 3.31)	77	3.44 (2.67, 4.21)	93	4.07 (3.23, 4.90)
5–9 years old	25	0.79 (0.47, 1.12)	23	0.79 (0.46, 1.13)	27	0.97 (0.59, 1.35)	31	1.15 (0.73, 1.57)	34	1.42 (0.94, 1.89)
10–14 years old	16	0.45 (0.22, 0.68)	19	0.53 (0.27, 0.79)	15	0.45 (0.21, 0.68)	19	0.57 (0.31, 0.84)	30	0.95 (0.60, 1.29)
15–19 years old	6	0.18 (0.03, 0.34)	10	0.31 (0.11, 0.51)	15	0.44 (0.20, 0.67)	16	0.47 (0.23, 0.71)	14	0.41 (0.19, 0.62)
20–44 years old	32	0.15 (0.10, 0.20)	44	0.21 (0.15, 0.28)	42	0.20 (0.14, 0.26)	45	0.22 (0.15, 0.28)	60	0.30 (0.22, 0.38)
45–64 years old	40	0.34 (0.23, 0.45)	57	0.47 (0.35, 0.60)	52	0.41 (0.30, 0.53)	53	0.41 (0.29, 0.52)	134	1.01 (0.84, 1.18)
Over 65 years	41	1.00 (0.69, 1.31)	63	1.43 (1.07, 1.78)	92	1.99 (1.58, 2.40)	89	1.84 (1.45, 2.22)	174	3.48 (2.96, 4.00)
Men	341	0.77 (0.69, 0.86)	370	0.79 (0.71, 0.88)	384	0.83 (0.74, 0.91)	411	0.87 (0.78, 0.95)	574	1.24 (1.14, 1.34)
Adults	159	0.55 (0.46, 0.64)	146	0.48 (0.40, 0.56)	178	0.57 (0.48, 0.65)	192	0.59 (0.50, 0.67)	317	0.97 (0.86, 1.08)
0–4 years old	108	4.78 (3.87, 5.69)	117	5.18 (4.24, 6.13)	100	4.47 (3.59, 5.35)	100	4.47 (3.59, 5.35)	120	5.27 (4.33, 6.22)
5–9 years old	33	1.06 (0.68, 1.44)	52	1.81 (1.31, 2.32)	40	1.46 (1.00, 1.92)	45	1.73 (1.22, 2.23)	59	2.44 (1.81, 3.07)
10–14 years old	23	0.66 (0.38, 0.94)	28	0.82 (0.51, 1.14)	36	1.07 (0.71, 1.43)	38	1.15 (0.77, 1.53)	40	1.28 (0.88, 1.67)
15–19 years old	18	0.56 (0.29, 0.84)	27	0.85 (0.52, 1.17)	30	0.91 (0.58, 1.24)	36	1.07 (0.71, 1.42)	38	1.10 (0.74, 1.46)
20–44 years old	53	0.26 (0.19, 033)	52	0.25 (0.18, 0.32)	56	0.27 (0.20, 0.35)	60	0.29 (0.22, 0.37)	70	0.35 (0.27, 0.43)
45–64 years old	59	0.51 (0.38, 0.64)	48	0.39 (0.28, 0.51)	70	0.57 (0.43, 0.70)	72	0.56 (0.43, 0.69)	124	0.93 (0.77, 1.10)
Over 65 years	47	1.14 (0.81, 1.47)	46	1.03 (0.73, 1.33)	52	1.12 (0.81, 1.43)	60	1.23 (0.91, 1.54)	123	2.46 (2.02, 2.90)

Cont. Suppl. Table 1-11.

Variables	2011		2012		2013		2014		2015	
	n	Prevalence (95% CI)	n	Prevalence (95% CI)	n	Prevalence (95% CI)	n	Prevalence (95% CI)	n	Prevalence (95% CI)
All	1074	2.31 (2.17, 2.45)	1116	2.31 (2.18, 2.45)	1164	2.37 (2.23, 2.51)	1231	2.46 (2.32, 2.60)	1319	2.61 (2.47, 2.75)
Adults	650	1.93 (1.78, 2.07)	657	1.83 (1.69, 1.97)	696	1.86 (1.72, 1.99)	747	1.91 (1.77, 2.04)	788	1.95 (1.82, 2.09)
0–4 years old	201	8.72 (7.51, 9.93)	236	10.2 (8.93, 11.5)	238	10.4 (9.09, 11.7)	236	10.3 (9.01, 11.6)	257	11.4 (10.0, 12.8)
5–9 years old	90	3.90 (3.09, 4.71)	98	4.26 (3.41, 5.10)	105	4.57 (3.69, 5.45)	103	4.52 (3.64, 5.40)	114	4.92 (4.01, 5.83)
10–14 years old	65	2.15 (1.62, 2.67)	70	2.43 (1.86, 3.01)	65	2.35 (1.77, 2.93)	78	3.01 (2.34, 3.68)	84	3.51 (2.75, 4.26)
15–19 years old	68	2.01 (1.53, 2.49)	55	1.64 (1.20, 2.07)	60	1.82 (1.36, 2.29)	67	2.08 (1.57, 2.58)	76	2.42 (1.87, 2.97)
20–44 years old	119	0.60 (0.49, 0.71)	157	0.80 (0.67, 0.93)	168	0.87 (0.73, 1.00)	189	0.98 (0.84, 1.12)	179	0.94 (0.80, 1.08)
45–64 years old	259	1.90 (1.67, 2.13)	248	1.76 (1.54, 1.98)	264	1.82 (1.60, 2.04)	272	1.82 (1.60, 2.03)	283	1.84 (1.62, 2.05)
Over 65 years	272	5.23 (4.61, 5.86)	252	4.59 (4.02, 5.16)	264	4.59 (4.04, 5.15)	286	4.74 (4.19, 5.30)	326	5.23 (4.66, 5.80)
Women	499	1.09 (0.99, 1.18)	503	1.06 (0.96, 1.15)	543	1.12 (1.02, 1.21)	544	1.09 (1.00, 1.18)	582	1.16 (1.06, 1.25)
Adults	330	0.99 (0.88, 1.09)	329	0.93 (0.82, 1.03)	359	0.97 (0.87, 1.07)	364	0.93 (0.84, 1.03)	382	0.95 (0.86, 1.05)
0–4 years old	91	3.93 (3.12, 4.74)	101	4.38 (3.52, 5.24)	97	4.24 (3.40, 5.09)	96	4.20 (3.36, 5.04)	105	4.65 (3.75, 5.54)
5–9 years old	29	1.24 (0.78, 1.70)	29	1.24 (0.78, 1.70)	42	1.81 (1.26, 2.37)	28	1.19 (0.73, 1.65)	42	1.81 (1.26, 2.37)
10–14 years old	31	0.99 (0.62, 1.35)	26	0.90 (0.55, 1.26)	23	0.82 (0.48, 1.17)	34	1.28 (0.83, 1.72)	32	1.32 (0.85, 1.78)
15–19 years old	18	0.50 (0.25, 0.75)	18	0.53 (0.28, 0.78)	22	0.66 (0.38, 0.94)	22	0.66 (0.37, 0.95)	21	0.66 (0.37, 0.95)
20–44 years old	50	0.25 (0.18, 0.32)	69	0.34 (0.26, 0.43)	70	0.36 (0.27, 0.44)	76	0.39 (0.30, 0.48)	68	0.35 (0.26, 0.44)
45–64 years old	129	0.94 (0.78, 1.10)	112	0.79 (0.64, 0.94)	125	0.86 (0.71, 1.01)	128	0.85 (0.70, 1.00)	124	0.80 (0.66, 0.94)
Over 65 years	151	2.90 (2.44, 3.37)	148	2.69 (2.25, 3.13)	164	2.84 (2.40, 3.28)	160	2.66 (2.25, 3.07)	190	3.04 (2.61, 3.47)
Men	575	1.22 (1.12, 1.32)	613	1.25 (1.15, 1.35)	621	1.25 (1.15, 1.34)	687	1.36 (1.26, 1.46)	737	1.45 (1.35, 1.56)
Adults	320	0.93 (0.83, 1.04)	328	0.90 (0.80, 1.00)	337	0.88 (0.79, 0.98)	383	0.97 (0.87, 1.06)	406	1.00 (0.90, 1.09)
0–4 years old	110	4.74 (3.84, 5.63)	135	5.86 (4.86, 6.85)	141	6.17 (5.15, 7.19)	140	6.12 (5.11, 7.14)	152	6.75 (5.68, 7.82)
5–9 years old	61	2.61 (1.95, 3.28)	69	2.97 (2.26, 3.68)	63	2.75 (2.06, 3.43)	75	3.28 (2.53, 4.03)	72	3.10 (2.38, 3.83)
10–14 years old	34	1.11 (0.73, 1.49)	44	1.52 (1.07, 1.98)	42	1.52 (1.06, 1.99)	44	1.69 (1.19, 2.19)	52	2.15 (1.55, 2.74)
15–19 years old	50	1.48 (1.07, 1.89)	37	1.10 (0.74, 1.46)	38	1.13 (0.76, 1.50)	45	1.38 (0.97, 1.80)	55	1.76 (1.29, 2.23)
20–44 years old	69	0.34 (0.26, 0.43)	88	0.44 (0.35, 0.54)	98	0.50 (0.40, 0.60)	113	0.58 (0.47, 0.69)	111	0.58 (0.47, 0.69)
45–64 years old	130	0.95 (0.78, 1.11)	136	0.97 (0.80, 1.13)	139	0.95 (0.79, 1.11)	144	0.96 (0.80, 1.12)	159	1.03 (0.87, 1.19)
Over 65 years	121	2.32 (1.91, 2.74)	104	1.88 (1.52, 2.25)	100	1.73 (1.39, 2.07)	126	2.08 (1.71, 2.45)	136	2.17 (1.80, 2.54)

a Age-standardized prevalence rates of stenosis or malformation of aorta were calculated using age groups according to the direct method using the estimated Korean population in 2015 as a reference.

Supplementary Table 1-12. Age-standardized prevalence[a] and 95% confidence interval (CI) of Tetralogy of Fallot overall and by sex (per 100,000).

Variables	2006		2007		2008		2009		2010	
	n	Prevalence (95% CI)	n	Prevalence (95% CI)	n	Prevalence (95% CI)	n	Prevalence (95% CI)	n	Prevalence (95% CI)
All	2,370	4.47 (4.28, 4.65)	2,559	4.82 (4.63, 5.01)	2,540	4.83 (4.64, 5.02)	2,949	5.59 (5.38, 5.79)	3,074	5.84 (5.64, 6.05)
Adults	494	1.32 (1.20, 1.43)	544	1.42 (1.30, 1.55)	627	1.63 (1.50, 1.76)	760	1.95 (1.81, 2.09)	813	2.06 (1.92, 2.21)
0–4 years old	699	31.2 (28.9, 33.5)	702	31.1 (28.8, 33.4)	675	30.1 (27.9, 32.4)	733	32.8 (30.4, 35.2)	737	32.4 (30.1, 34.8)
5–9 years old	493	16.4 (15.0, 17.9)	511	18.1 (16.5, 19.7)	466	17.3 (15.8, 18.9)	530	20.4 (18.7, 22.2)	540	22.5 (20.6, 24.4)
10–14 years old	427	12.6 (11.4, 13.8)	457	13.8 (12.5, 15.0)	430	13.2 (12.0, 14.5)	477	14.9 (13.6, 16.3)	495	15.8 (14.4, 17.2)
15–19 years old	257	8.48 (7.44, 9.52)	345	10.9 (9.81, 12.1)	342	10.6 (9.50, 11.7)	449	13.5 (12.3, 14.8)	489	14.5 (13.2, 15.7)
20–44 years old	417	2.05 (1.85, 2.24)	463	2.30 (2.09, 2.51)	519	2.59 (2.37, 2.82)	636	3.21 (2.96, 3.46)	672	3.42 (3.16, 3.68)
45–64 years old	70	0.61 (0.46, 0.75)	77	0.64 (0.49, 0.79)	100	0.81 (0.65, 0.97)	110	0.86 (0.70, 1.02)	124	0.97 (0.77, 1.10)
Over 65 years	7	0.16 (0.04, 0.29)	4	0.07 (0.00, 0.16)	8	0.16 (0.04, 0.28)	14	0.28 (0.13, 0.44)	17	0.33 (0.17, 0.49)
Women	989	1.87 (1.75, 1.99)	1,074	2.04 (1.91, 2.16)	1,091	2.08 (1.96, 2.21)	1,212	2.31 (2.18, 2.44)	1,290	2.46 (2.33, 2.60)
Adults	234	0.62 (0.54, 0.71)	245	0.64 (0.56, 0.73)	292	0.76 (0.67, 0.85)	357	0.92 (0.82, 1.01)	371	0.95 (0.85, 1.04)
0–4 years old	305	13.5 (12.0, 15.1)	319	14.1 (12.5, 15.6)	299	13.3 (11.8, 14.8)	317	14.1 (12.6, 15.7)	305	13.4 (11.9, 14.9)
5–9 years old	203	6.74 (5.81, 7.68)	223	7.90 (6.85, 8.94)	203	7.54 (6.50, 8.58)	217	8.34 (7.22, 9.46)	251	10.4 (9.17, 11.7)
10–14 years old	162	4.79 (4.05, 5.53)	164	4.96 (4.20, 5.72)	174	5.37 (4.57, 6.17)	172	5.37 (4.56, 6.18)	182	5.83 (4.98, 6.67)
15–19 years old	85	2.80 (2.20, 3.40)	123	3.91 (3.21, 4.60)	123	3.81 (3.13, 4.49)	149	4.51 (3.78, 5.23)	181	5.36 (4.57, 6.14)
20–44 years old	193	0.94 (0.81, 1.08)	201	0.99 (0.86, 1.13)	233	1.16 (1.01, 1.31)	291	1.46 (1.29, 1.63)	299	1.52 (1.34, 1.69)
45–64 years old	40	0.34 (0.23, 0.45)	41	0.34 (0.23, 0.45)	53	0.43 (0.31, 0.54)	57	0.44 (032, 0.56)	60	0.45 (0.33, 0.56)
Over 65 years	1	0.01 (0.00, 0.06)	3	0.06 (0.00, 0.13)	6	0.12 (0.01, 0.22)	9	0.18 (0.06, 0.30)	12	0.22 (0.09, 0.36)
Men	1,381	2.59 (2.45, 2.73)	1,485	2.78 (2.64, 2.92)	1,449	2.74 (2.60, 2.89)	1,737	3.27 (3.12, 3.43)	1,784	3.37 (3.22, 3.53)
Adults	260	0.69 (0.60, 0.77)	299	0.77 (0.68, 0.86)	335	0.86 (0.77, 0.95)	403	1.02 (0.92, 1.12)	442	1.11 (1.01, 1.22)
0–4 years old	394	17.5 (15.8, 19.3)	383	16.9 (15.2, 18.7)	376	16.8 (15.1, 18.5)	416	18.6 (16.8, 20.4)	432	19.0 (17.2, 20.8)
5–9 years old	290	9.67 (8.56, 10.7)	288	10.2 (9.02, 11.3)	263	9.80 (8.62, 10.9)	313	12.0 (10.7, 13.4)	289	12.0 (10.6, 13.4)
10–14 years old	265	7.85 (6.90, 8.80)	293	8.84 (7.83, 9.86)	256	7.89 (6.92, 8.86)	305	9.59 (8.51, 10.6)	313	10.0 (8.89, 11.1)
15–19 years old	172	5.67 (4.82, 6.52)	222	7.06 (6.13, 7.99)	219	6.81 (5.90, 7.71)	300	9.08 (8.05, 10.1)	308	9.14 (8.12, 10.1)
20–44 years old	224	1.09 (0.95, 1.24)	262	1.29 (1.14, 1.45)	286	1.43 (1.26, 1.59)	345	1.74 (1.55, 1.92)	373	1.89 (1.70, 2.09)
45–64 years old	30	0.25 (0.16, 0.35)	36	0.29 (0.19, 0.39)	47	0.37 (0.26, 0.48)	53	0.41 (0.29, 0.52)	64	0.48 (0.36, 0.60)
Over 65 years	6	0.13 (0.01, 0.25)	1	0.01 (0.00, 0.05)	2	0.03 (0.00, 0.09)	5	0.09 (0.01, 0.18)	5	0.09 (0.01, 0.17)

Cont. Suppl. Table 1-12.

Variables	2011		2012		2013		2014		2015	
	n	Prevalence (95% CI)	n	Prevalence (95% CI)	n	Prevalence (95% CI)	n	Prevalence (95% CI)	n	Prevalence (95% CI)
All	3,354	6.38 (6.16, 6.60)	3,406	6.53 (6.31, 6.75)	3,500	6.70 (6.48, 6.92)	3,563	6.87 (6.64, 7.09)	3,622	7.07 (6.84, 7.30)
Adults	960	2.39 (2.23, 2.54)	1,037	2.54 (2.39, 2.70)	1,142	2.76 (2.60, 2.92)	1,241	2.96 (2.79, 3.12)	1,320	3.12 (2.95, 3.29)
0–4 years old	774	33.6 (31.2, 36.0)	751	32.7 (30.3, 35.0)	754	33.0 (30.6, 35.4)	754	33.1 (30.7, 35.4)	752	33.4 (31.0, 35.8)
5–9 years old	550	23.9 (21.9, 25.9)	511	22.1 (20.2, 24.1)	505	22.1 (20.1, 24.0)	495	21.8 (19.9, 23.8)	502	21.8 (19.9, 23.7)
10–14 years old	515	17.1 (15.6, 18.5)	529	18.5 (16.9, 20.1)	544	20.0 (18.3, 21.6)	522	20.2 (18.4, 21.9)	487	20.4 (18.6, 22.2)
15–19 years old	555	16.4 (15.1, 17.8)	578	17.3 (15.9, 18.7)	555	16.9 (15.5, 18.3)	551	17.2 (15.8, 18.7)	561	18.0 (16.5, 19.5)
20–44 years old	800	4.08 (3.80, 4.37)	851	4.37 (4.08, 4.67)	937	4.86 (4.54, 5.17)	1,016	5.30 (4.98, 5.63)	1,087	5.74 (5.40, 6.08)
45–64 years old	141	1.03 (0.85, 1.20)	170	1.21 (1.02, 1.39)	188	1.29 (1.11, 1.48)	204	1.36 (1.17, 1.55)	216	1.41 (1.22, 1.59)
Over 65 years	19	0.36 (0.20, 0.53)	16	0.28 (0.14, 0.43)	17	0.28 (0.14, 0.43)	21	0.33 (0.18, 0.48)	17	0.25 (0.12, 0.38)
Women	1,417	2.71 (2.57, 2.85)	1,435	2.73 (2.59, 2.87)	1,486	2.85 (2.07, 2.99)	1,509	2.90 (2.76, 3.05)	1,502	2.93 (2.78, 3.07)
Adults	438	1.10 (0.99, 1.20)	465	1.14 (1.03, 1.24)	511	1.24 (1.13, 1.34)	546	1.30 (1.19, 1.41)	566	1.34 (1.23, 1.45)
0–4 years old	333	14.4 (12.8, 16.0)	327	14.2 (12.6, 15.7)	325	14.2 (12.6, 15.7)	330	14.4 (12.8, 16.0)	312	13.8 (12.3, 15.4)
5–9 years old	249	10.8 (9.48, 12.1)	225	9.76 (8.48, 11.0)	228	9.98 (8.68, 11.2)	218	9.63 (8.35, 10.9)	229	9.94 (8.64, 11.2)
10–14 years old	203	6.74 (5.81, 7.66)	225	7.89 (6.86, 8.93)	211	7.73 (6.68, 8.78)	219	8.47 (7.35, 9.60)	208	8.72 (7.53, 9.91)
15–19 years old	194	5.74 (4.92, 6.55)	193	5.77 (4.95, 6.59)	211	6.43 (5.56, 7.30)	196	6.15 (5.28, 7.01)	187	5.99 (5.13, 6.85)
20–44 years old	348	1.77 (1.58, 1.96)	375	1.92 (1.72, 2.12)	406	2.10 (1.89, 2.30)	436	2.28 (2.06, 2.49)	455	2.40 (2.18, 2.62)
45–64 years old	76	0.55 (0.43, 0.68)	81	0.57 (0.45, 0.70)	95	0.65 (0.51, 0.78)	98	0.65 (0.52, 0.78)	102	0.66 (0.53, 0.79)
Over 65 years	14	0.25 (0.11, 0.40)	9	0.15 (0.04, 0.25)	10	0.16 (0.05, 0.27)	12	0.19 (0.08, 0.31)	9	0.13 (0.04, 0.23)
Men	1,937	3.67 (3.50, 3.83)	1,971	3.75 (3.58, 3.91)	2,014	3.85 (3.68, 4.02)	1,502	3.96 (3.78, 4.13)	2,120	4.14 (3.96, 4.31)
Adults	522	1.28 (1.17, 1.39)	572	1.40 (1.28, 1.51)	631	1.52 (1.40, 1.64)	566	1.65 (1.53, 1.78)	754	1.78 (1.65, 1.91)
0–4 years old	441	19.1 (17.3, 20.9)	424	18.4 (16.6, 20.1)	429	18.7 (17.0, 20.5)	312	18.6 (16.8, 20.3)	440	19.5 (17.7, 21.3)
5–9 years old	301	13.1 (11.6, 14.6)	286	12.4 (10.9, 13.8)	277	12.1 (10.6, 135)	229	12.2 (10.8, 13.6)	273	11.8 (10.4, 13.3)
10–14 years old	312	10.3 (9.18, 11.4)	304	10.6 (9.46, 11.8)	333	12.2 (10.9, 13.5)	208	11.7 (10.4, 13.0)	279	11.7 (10.3, 13.0)
15–19 years old	361	10.7 (9.61, 11.8)	385	11.5 (10.3, 12.6)	344	10.5 (9.39, 11.6)	187	11.1 (9.97, 12.2)	374	12.0 (10.7, 13.2)
20–44 years old	452	2.30 (2.09, 2.52)	476	2.44 (2.22, 2.66)	531	2.75 (2.51, 2.98)	455	3.02 (2.78, 3.27)	632	3.33 (3.07, 3.60)
45–64 years old	65	0.47 (0.35, 0.58)	89	0.63 (0.49, 0.76)	93	0.63 (0.50, 0.76)	102	0.71 (0.57, 0.84)	114	0.73 (0.60, 0.87)
Over 65 years	5	0.09 (0.01, 0.17)	7	0.12 (0.02, 0.21)	7	0.12 (0.03, 0.21)	9	0.13 (0.03, 0.23)	8	0.12 (0.03, 0.21)

a Age-standardized prevalence rates of tetralogy of fallot were calculated using age groups according to the direct method using the estimated Korean population in 2015 as a reference.

Supplementary Table 1-13. Age-standardized prevalence[a] and 95% confidence interval (CI) of Ebstein anomaly overall and by sex (per 100,000).

Variables	2006		2007		2008		2009		2010	
	n	Prevalence (95% CI)	n	Prevalence (95% CI)	n	Prevalence (95% CI)	n	Prevalence (95% CI)	n	Prevalence (95% CI)
All	308	0.68 (0.61, 0.76)	341	1.09 (1.00, 1.19)	388	0.83 (0.75, 0.91)	425	0.89 (0.81, 0.98)	467	0.96 (0.88, 1.05)
Adults	190	0.60 (0.51, 0.69)	200	0.62 (0.53, 0.71)	242	0.72 (0.62, 0.81)	267	0.77 (0.68, 0.87)	285	0.80 (0.71, 0.89)
0–4 years old	60	2.63 (1.96, 3.31)	66	4.07 (3.24, 4.89)	70	3.13 (2.39, 3.86)	75	3.35 (2.59, 4.11)	86	3.75 (2.95, 4.55)
5–9 years old	15	0.48 (0.23, 0.74)	31	1.81 (1.26, 2.37)	30	1.10 (0.70, 1.51)	31	1.15 (0.73, 1.57)	42	1.73 (1.20, 2.26)
10–14 years old	20	0.57 (0.31, 0.83)	24	1.03 (0.65, 1.41)	24	0.70 (0.40, 1.00)	27	0.82 (0.50, 1.14)	30	0.95 (0.60, 1.29)
15–19 years old	23	0.75 (0.44, 1.06)	20	0.85 (0.53, 1.16)	22	0.66 (0.37, 0.94)	25	0.75 (0.46, 1.05)	24	0.69 (0.40, 0.97)
20–44 years old	96	0.47 (0.37, 0.56)	89	0.71 (0.59, 0.82)	112	0.56 (0.45, 0.66)	114	0.57 (0.46, 0.67)	123	0.62 (0.51, 0.73)
45–64 years old	66	0.57 (0.43, 0.71)	80	1.08 (0.91, 1.25)	99	0.80 (0.64, 0.96)	119	0.93 (0.76, 1.09)	125	0.94 (0.77, 1.11)
Over 65 years	28	0.68 (0.43, 0.93)	31	1.03 (0.76, 1.30)	31	0.66 (0.43, 0.90)	34	0.70 (0.46, 0.93)	37	0.73 (0.49, 0.97)
Women	170	0.38 (0.32, 0.44)	187	0.61 (0.54, 0.68)	214	0.47 (0.40, 0.53)	251	0.54 (0.47, 0.60)	262	0.55 (0.48, 0.61)
Adults	111	0.35 (0.28, 0.42)	124	0.39 (0.32, 0.46)	148	0.44 (0.37, 0.51)	174	0.51, 0.43, 0.58)	175	0.49 (0.42, 0.57)
0–4 years old	30	1.29 (0.81, 1.77)	27	1.69 (1.15, 2.24)	32	1.43 (0.93, 1.92)	35	1.56 (1.04, 2.08)	40	1.74 (1.19, 2.29)
5–9 years old	9	0.26 (0.06, 0.46)	13	0.93 (0.53, 1.33)	11	0.39 (0.15, 0.64)	15	0.57 (0.28, 0.87)	27	1.10 (0.68, 1.53)
10–14 years old	7	0.20 (0.05, 0.36)	11	0.49 (0.22, 0.76)	11	0.33 (0.12, 0.53)	13	0.37 (0.14, 0.59)	11	0.33 (0.12, 0.53)
15–19 years old	13	0.41 (0.17, 0.64)	12	0.22 (0.05, 0.38)	12	0.34 (0.13, 0.55)	14	0.41 (0.18, 0.63)	9	0.25 (0.07, 0.42)
20–44 years old	53	0.26 (0.19, 0.33)	51	0.38 (0.30, 0.47)	61	0.30 (0.22, 0.38)	67	0.33 (0.25, 0.41)	70	0.35 (0.27, 0.43)
45–64 years old	42	0.36 (0.25, 0.47)	55	0.71 (0.57, 0.85)	66	0.53 (0.40, 0.66)	83	0.65 (0.51, 0.79)	81	0.61 (0.47, 0.74)
Over 65 years	16	0.38 (0.18, 0.57)	18	0.70 (0.47, 0.92)	21	0.44 (0.24, 0.63)	24	0.48 (0.28, 0.68)	24	0.47 (0.27, 0.66)
Men	138	0.30 (0.24, 0.35)	154	0.48 (0.42, 0.54)	174	0.36 (0.30, 0.41)	174	0.35 (0.30, 0.41)	205	0.41 (0.35, 0.47)
Adults	79	0.24 (0.19, 0.30)	76	0.23 (0.18, 0.81)	94	0.27 (0.21, 0.32)	93	0.26 (0.21, 0.31)	110	0.30 (0.24, 0.36)
0–4 years old	30	1.29 (0.81, 1.77)	39	2.32 (1.69, 2.95)	38	1.69 (1.15, 2.24)	40	1.78 (1.22, 2.34)	46	2.01 (1.42, 2.59)
5–9 years old	6	0.17 (0.01, 0.33)	18	0.84 (0.46, 1.22)	19	0.66 (0.34, 0.98)	16	0.57 (0.27, 0.88)	15	0.62 (0.30, 0.93)
10–14 years old	13	0.32 (0.16, 0.58)	13	0.49 (0.22, 0.76)	13	0.37 (0.15, 0.59)	14	0.41 (0.18, 0.64)	19	0.57 (0.30, 0.85)
15–19 years old	10	0.31 (0.11, 0.52)	8	0.63 (0.36, 0.90)	10	0.28 (0.09, 0.47)	11	0.31 (0.11, 0.51)	15	0.44 (0.21, 0.66)
20–44 years old	43	0.21 (0.14, 0.27)	38	0.32 (0.24, 0.40)	51	0.25 (0.18, 0.32)	47	0.23 (0.16, 0.30)	53	0.26 (0.19, 0.33)
45–64 years old	24	0.20 (0.12, 0.29)	25	0.36 (0.26, 0.46)	33	0.26 (0.17, 0.35)	36	0.27 (0.18, 0.37)	44	0.33 (0.23, 0.43)
Over 65 years	12	0.28 (0.12, 0.45)	13	0.31 (0.16, 0.47)	10	0.21 (0.07, 0.34)	10	0.19 (0.06, 0.32)	13	0.25 (0.11, 0.40)

Cont. Suppl. Table 1-13.

Variables	2011		2012		2013		2014		2015	
	n	Prevalence (95% CI)	n	Prevalence (95% CI)	n	Prevalence (95% CI)	n	Prevalence (95% CI)	n	Prevalence (95% CI)
All	513	1.05 (0.96, 1.14)	543	1.09 (1.00, 1.19)	594	1.18 (1.08, 1.27)	634	1.24 (1.15, 1.34)	651	1.27 (1.17, 1.37)
Adults	333	0.90 (0.80, 1.00)	348	0.92 (0.82, 1.02)	384	0.98 (0.88, 1.08)	407	1.01 (0.91, 1.11)	430	1.04 (0.94, 1.14)
0–4 years old	88	3.80 (3.00, 4.60)	94	4.07 (3.24, 4.89)	95	4.16 (3.32, 4.99)	110	4.78 (3.88, 5.68)	111	4.92 (4.00, 5.83)
5–9 years old	43	1.86 (1.30, 2.42)	42	1.81 (1.26, 2.37)	44	1.90 (1.33, 2.47)	45	1.95 (1.37, 2.53)	42	1.81 (1.26, 2.37)
10–14 years old	20	0.66 (0.37, 0.95)	30	1.03 (0.65, 1.41)	37	1.32 (0.88, 1.76)	38	1.44 (0.97, 1.91)	35	1.44 (0.95, 1.93)
15–19 years old	29	0.85 (0.53, 1.16)	29	0.85 (0.53, 1.16)	34	1.00 (0.65, 1.35)	34	1.04 (0.68, 1.39)	33	1.04 (0.67, 1.40)
20–44 years old	150	0.76 (0.64, 0.88)	139	0.71 (0.59, 0.82)	157	0.81 (0.68, 0.93)	154	0.80 (0.67, 0.93)	152	0.79 (0.67, 1.40)
45–64 years old	138	1.01 (0.84, 1.18)	152	1.08 (0.91, 1.25)	163	1.12 (0.95, 1.29)	185	1.23 (1.05, 1.41)	203	1.32 (1.14, 1.50)
Over 65 years	45	0.86 (0.61, 1.12)	57	1.03 (0.76, 1.30)	64	1.11 (0.83, 1.38)	68	1.12 (0.85, 1.39)	75	1.20 (0.92, 1.47)
Women	290	0.60 (0.53, 0.67)	300	0.61 (0.54, 0.68)	358	0.71 (0.64, 0.79)	372	0.73 (0.66, 0.81)	387	0.75 (0.68, 0.83)
Adults	210	0.57 (0.49, 0.65)	215	0.57 (0.49, 0.65)	245	0.63 (0.55, 0.71)	259	0.64 (0.56, 0.72)	282	0.68 (0.60, 0.76)
0–4 years old	37	1.56 (1.04, 2.08)	40	1.69 (1.15, 2.24)	50	2.19 (1.58, 2.80)	54	2.37 (1.73, 3.00)	48	2.10 (1.49, 2.70)
5–9 years old	22	0.93 (0.53, 1.33)	22	0.93 (0.53, 1.33)	24	1.02 (0.59, 1.44)	22	0.93 (0.52, 1.33)	22	0.93 (0.53, 1.33)
10–14 years old	10	0.33 (0.12, 0.53)	15	0.49 (0.22, 0.76)	19	0.66 (0.34, 0.97)	22	0.82 (0.47, 1.18)	17	0.70 (0.36, 1.04)
15–19 years old	11	0.31 (0.12, 0.50)	8	0.22 (0.05, 0.38)	20	0.59 (0.33, 0.86)	15	0.44 (0.20, 0.68)	18	0.56 (0.30, 0.83)
20–44 years old	86	0.43 (0.34, 0.53)	76	0.38 (0.30, 0.47)	89	0.46 (0.36, 0.55)	88	0.45 (0.35, 0.55)	89	0.46 (0.36, 0.56)
45–64 years old	91	0.66 (0.52, 0.80)	100	0.71 (0.57, 0.85)	115	0.79 (0.64, 0.93)	124	0.83 (0.68, 0.97)	137	0.89 (0.74, 1.04)
Over 65 years	33	0.62 (0.40, 0.84)	39	0.70 (0.47, 0.92)	41	0.70 (0.48, 0.91)	47	0.77 (0.55, 1.00)	56	0.89 (0.66, 1.13)
Men	223	0.44 (0.38, 0.50)	243	0.48 (0.42, 0.54)	236	0.46 (0.40, 0.52)	262	0.51 (0.45, 0.57)	264	0.51 (0.45, 0.57)
Adults	123	0.32 (0.27, 0.38)	133	0.34 (0.28, 0.40)	139	0.35 (0.29, 0.41)	148	0.36 (0.30, 0.42)	148	0.35 (0.29, 0.41)
0–4 years old	51	2.19 (1.58, 2.80)	54	2.32 (1.69, 2.95)	45	1.96 (1.39, 2.54)	56	2.41 (1.77, 3.05)	63	2.77 (2.08, 3.46)
5–9 years old	21	0.88 (0.49, 1.27)	20	0.84 (0.46, 1.22)	20	0.84 (0.45, 1.22)	23	0.97 (0.56, 1.39)	20	0.84 (0.46, 1.22)
10–14 years old	10	0.33 (0.12, 0.53)	15	0.49 (0.22, 0.76)	18	0.66 (0.35, 0.96)	16	0.57 (0.27, 0.88)	18	0.74 (0.39, 1.09)
15–19 years old	18	0.50 (0.25, 0.75)	21	0.63 (0.36, 0.90)	14	0.41 (0.18, 0.63)	19	0.56 (0.29, 0.83)	15	0.47 (0.22, 0.71)
20–44 years old	64	0.32 (0.24, 0.40)	63	0.32 (0.24, 0.40)	68	0.34 (0.26, 0.43)	66	0.34 (0.26, 0.42)	63	0.33 (0.25, 0.41)
45–64 years old	47	0.33 (0.24, 0.43)	52	0.36 (0.26, 0.46)	48	0.32 (0.23, 0.41)	61	0.40 (0.30, 0.50)	66	0.42 (0.32, 0.52)
Over 65 years	12	0.22 (0.09, 0.35)	18	0.31 (0.16, 0.47)	23	0.39 (0.23, 0.55)	21	0.33 (0.18, 0.48)	19	0.30 (0.16, 0.44)

a Age-standardized prevalence rates of ebstein anomaly were calculated using age groups according to the direct method using the estimated Korean population in 2015 as a reference.

Supplementary Table 1-14. Age-standardized prevalence[a] and 95% confidence interval (CI) of transposition of the great arteries overall and by sex (per 100,000).

Variables	2006		2007		2008		2009		2010	
	n	Prevalence (95% CI)	n	Prevalence (95% CI)	n	Prevalence (95% CI)	n	Prevalence (95% CI)	n	Prevalence (95% CI)
All	946	1.77 (1.65, 1.88)	1,013	1.90 (1.78, 2.02)	1,111	2.09 (1.97, 2.22)	1,206	2.25 (2.12, 2.38)	1,350	2.54 (2.40, 2.67)
Adults	127	0.38 (0.31, 0.45)	139	0.41 (0.34, 0.48)	147	0.42 (0.35, 0.49)	170	0.46 (0.39, 0.53)	193	0.50 (0.43, 0.58)
0–4 years old	254	11.3 (9.92, 12.7)	270	11.9 (10.5, 13.4)	277	12.3 (10.9, 13.8)	285	12.7 (11.2, 14.2)	299	13.1 (11.6, 14.6)
5–9 years old	288	9.58 (8.47, 10.6)	293	10.3 (9.19, 11.5)	299	11.1 (9.87, 12.4)	310	11.9 (10.6, 13.2)	320	13.3 (11.8, 14.8)
10–14 years old	181	5.37 (4.59, 6.16)	199	5.99 (5.15, 6.83)	234	7.23 (6.30, 8.16)	272	8.51 (7.50, 9.53)	308	9.84 (8.73, 10.9)
15–19 years old	96	3.15 (2.51, 3.78)	112	3.56 (2.90, 4.22)	154	4.79 (4.03, 5.55)	169	5.10 (4.33, 5.88)	230	6.81 (5.92, 7.69)
20–44 years old	73	0.35 (0.27, 0.43)	80	0.39 (0.30, 0.48)	90	0.44 (0.35, 0.54)	114	0.57 (0.46, 0.67)	136	0.68 (0.57, 0.80)
45–64 years old	43	0.37 (0.25, 0.48)	42	0.35 (0.24, 0.45)	38	0.30 (0.20, 0.40)	38	0.29 (0.20, 0.39)	43	0.32 (0.22, 0.42)
Over 65 years	11	0.25 (0.09, 0.41)	17	0.38 (0.19, 0.56)	19	0.41 (0.22, 0.59)	18	0.36 (0.19, 0.53)	14	0.27 (0.12, 0.42)
Women	266	0.52 (0.46, 0.59)	269	0.52 (0.46, 0.59)	285	0.55 (0.49, 0.62)	316	0.61 (0.54, 0.68)	359	0.69 (0.62, 0.76)
Adults	68	0.20 (0.15, 0.26)	63	0.19 (0.14, 0.24)	65	0.19 (0.14, 0.23)	82	0.22 (0.17, 0.27)	93	0.24 (0.19, 0.30)
0–4 years old	83	3.66 (2.87, 4.46)	78	3.44 (2.67, 4.21)	82	3.66 (2.87, 4.46)	85	3.80 (2.99, 4.61)	100	4.38 (3.51, 5.24)
5–9 years old	55	1.81 (1.33, 2.30)	58	2.04 (1.51, 2.57)	61	2.26 (1.69, 2.83)	65	2.48 (1.87, 3.09)	67	2.79 (2.12, 3.46)
10–14 years old	35	1.03 (0.68, 1.37)	43	1.28 (0.89, 1.67)	43	1.32 (0.92, 1.72)	45	1.40 (0.99, 1.81)	49	1.52 (1.09, 1.96)
15–19 years old	25	0.82 (0.49, 1.14)	27	0.85 (0.52, 1.17)	34	1.04 (0.68, 1.39)	39	1.16 (0.79, 1.53)	50	1.48 (1.07, 1.89)
20–44 years old	36	0.17 (0.11, 0.22)	32	0.15 (0.10, 0.21)	35	0.17 (0.11, 0.23)	50	0.24 (0.17, 0.31)	60	0.30 (0.22, 0.38)
45–64 years old	26	0.22 (0.13, 0.31)	20	0.16 (0.09, 0.24)	19	0.15 (0.08, 0.22)	20	0.15 (0.08, 0.22)	22	0.16 (0.09, 0.23)
Over 65 years	6	0.13 (0.01, 0.25)	11	0.24 (0.09, 0.39)	11	0.22 (0.08, 0.36)	12	0.24 (0.10, 0.38)	11	0.21 (0.08, 0.34)
Men	680	1.24 (1.14, 1.33)	744	1.37 (1.27, 1.47)	826	1.53 (1.43, 1.64)	890	1.64 (1.53, 1.75)	991	1.84 (1.72, 1.95)
Adults	59	0.17 (0.12, 0.22)	76	0.21 (0.16, 0.26)	82	0.22 (0.17, 0.27)	88	0.23 (0.18, 0.28)	100	0.25 (0.20, 0.30)
0–4 years old	171	7.60 (6.45, 8.75)	192	8.49 (7.29, 9.70)	195	8.72 (7.49, 9.94)	200	8.94 (7.70, 10.1)	199	8.76 (7.54, 9.98)
5–9 years old	233	7.76 (6.76, 8.76)	235	8.34 (7.27, 9.41)	238	8.87 (7.74, 10.0)	245	9.45 (8.26, 10.6)	253	10.5 (9.26, 11.8)
10–14 years old	146	4.34 (3.63, 5.04)	156	4.71 (3.97, 5.45)	191	5.87 (5.03, 6.71)	227	7.11 (6.18, 8.04)	259	8.27 (7.25, 9.28)
15–19 years old	71	2.33 (1.78, 2.88)	85	2.66 (2.10, 3.25)	120	3.72 (3.05, 4.39)	130	3.91 (3.23, 4.58)	180	5.33 (4.54, 6.11)
20–44 years old	37	0.17 (0.11, 0.23)	48	0.23 (0.17, 0.30)	55	0.27 (0.19, 0.34)	64	0.32 (0.24, 0.40)	76	0.38 (0.29, 0.46)
45–64 years old	17	0.14 (0.07, 0.21)	22	0.17 (0.10, 0.25)	19	0.15 (0.08, 0.22)	18	0.13 (0.07, 0.20)	21	0.15 (0.08, 0.22)
Over 65 years	5	0.12 (0.01, 0.22)	6	0.12 (0.01, 0.23)	8	0.16 (0.04, 0.28)	6	0.12 (0.02, 0.22)	3	0.04 (0.00, 0.11)

Cont. Suppl. Table 1-14.

Variables	2011		2012		2013		2014		2015	
	n	Prevalence (95% CI)	n	Prevalence (95% CI)	n	Prevalence (95% CI)	n	Prevalence (95% CI)	n	Prevalence (95% CI)
All	1,558	2.92 (2.78, 3.07)	1,645	3.11 (2.96, 3.26)	1,636	3.13 (2.97, 3.28)	1,711	3.31 (3.15, 3.47)	1,717	3.38 (3.22, 3.54)
Adults	248	0.62 (0.54, 0.70)	281	0.70 (0.62, 0.78)	284	0.69 (0.61, 0.77)	312	0.75 (0.66, 0.83)	350	0.83 (0.74, 0.92)
0–4 years old	299	12.9 (11.5, 14.4)	333	14.4 (12.9, 16.0)	331	14.4 (12.9, 16.0)	340	14.8 (13.3, 16.4)	328	14.5 (12.9, 16.1)
5–9 years old	338	14.7 (13.1, 16.3)	331	14.3 (12.8, 15.9)	304	13.3 (11.8, 14.8)	328	14.5 (12.9, 16.0)	298	12.9 (11.4, 14.4)
10–14 years old	394	13.0 (11.7, 14.3)	405	14.2 (12.8, 15.6)	384	14.1 (12.7, 15.5)	362	14.0 (12.5, 15.4)	357	14.9 (13.4, 16.5)
15–19 years old	279	8.29 (7.32, 9.26)	295	8.86 (7.85, 9.87)	333	10.1 (9.09, 11.2)	369	11.5 (10.3, 12.7)	384	12.3 (11.0, 13.5)
20–44 years old	186	0.94 (0.81, 1.08)	202	1.03 (0.89, 1.18)	209	1.08 (0.93, 1.22)	229	1.19 (1.03, 1.34)	259	1.36 (1.19, 1.53)
45–64 years old	50	0.36 (0.26, 0.46)	58	0.41 (0.30, 0.51)	55	0.37 (0.27, 0.47)	62	0.41 (0.30, 0.51)	68	0.43 (0.33, 0.54)
Over 65 years	12	0.22 (0.09, 0.35)	21	0.38 (0.21, 0.54)	20	0.33 (0.18, 0.48)	21	0.33 (0.18, 0.48)	23	0.36 (0.21, 0.51)
Women	371	0.70 (0.63, 0.78)	399	0.76 (0.69, 0.84)	399	0.77 (0.69, 0.84)	393	0.76 (0.68, 0.83)	393	0.76 (0.69, 0.84)
Adults	115	0.29 (0.24, 0.35)	134	0.33 (0.28, 0.39)	129	0.31 (0.26, 027)	144	0.34 (0.29, 0.40)	166	0.39 (0.33, 0.45)
0–4 years old	75	3.22 (2.48, 3.95)	81	3.48 (2.72, 4.25)	92	4.02 (3.20, 4.85)	82	3.57 (2.79, 4.35)	76	3.35 (2.59, 4.11)
5–9 years old	66	2.84 (2.14, 3.53)	73	3.15 (2.42, 3.87)	62	2.70 (2.03, 3.38)	64	2.79 (2.10, 3.49)	54	2.35 (1.72, 2.98)
10–14 years old	70	2.31 (1.77, 2.86)	61	2.10 (1.57, 2.64)	65	2.35 (1.77, 2.93)	58	2.23 (1.65, 2.81)	62	2.60 (1.95, 3.25)
15–19 years old	45	1.32 (0.93, 1.71)	50	1.48 (1.06, 1.89)	51	1.54 (1.11, 1.97)	45	1.38 (0.97, 1.80)	35	1.10 (0.73, 1.47)
20–44 years old	79	0.39 (0.31, 0.48)	89	0.45 (0.35, 0.55)	86	0.44 (0.34, 0.53)	94	0.48 (0.38, 0.58)	105	0.55 (0.44, 0.66)
45–64 years old	27	0.19 (0.11, 0.26)	32	0.22 (0.14, 0.30)	29	0.19 (0.12, 0.27)	34	0.22 (0.14, 0.30)	43	0.27 (0.19, 036)
Over 65 years	9	0.16 (0.05, 0.28)	13	0.22 (0.09, 0.35)	14	0.24 (0.11, 0.37)	16	0.25 (0.12, 0.38)	18	0.28 (0.15, 0.42)
Men	1,187	2.21 (2.09, 2.34)	1,246	2.34 (2.21, 2.47)	1,237	2.36 (2.22, 2.49)	1,318	2.55 (2.41, 2.69)	1,324	2.61 (2.47, 2.75)
Adults	133	0.33 (0.27, 0.38)	147	0.36 (0.30, 0.42)	155	0.37 (0.31, 0.43)	168	0.40 (0.34, 0.46)	184	0.43 (0.37, 0.49)
0–4 years old	224	9.70 (8.43, 10.9)	252	10.9 (9.60, 12.3)	239	10.4 (9.13, 11.7)	258	11.3 (9.93, 12.7)	252	11.1 (9.80, 12.5)
5–9 years old	272	11.8 (10.4, 13.2)	258	11.1 (9.81, 12.5)	242	10.6 (9.27, 11.9)	264	11.6 (10.2, 13.0)	244	10.6 (9.27, 11.9)
10–14 years old	324	10.7 (9.57, 11.9)	344	12.0 (10.7, 13.3)	319	11.7 (10.4, 13.0)	304	11.7 (10.4, 13.0)	295	12.3 (10.9, 13.7)
15–19 years old	234	6.93 (6.04, 7.83)	245	7.34 (6.42, 8.27)	282	8.61 (7.60, 9.61)	324	10.1 (9.04, 11.2)	349	11.1 (10.0, 12.3)
20–44 years old	107	0.54 (0.44, 0.64)	113	0.57 (0.46, 0.68)	123	0.63 (0.52, 0.75)	135	0.70 (0.58, 0.82)	154	0.81 (0.68, 0.93)
45–64 years old	23	0.16 (0.09, 0.23)	26	0.17 (0.10, 0.25)	26	0.17 (0.11, 0.24)	28	0.18 (0.11, 0.25)	25	0.15 (0.09, 0.22)
Over 65 years	3	0.04 (0.00, 0.11)	8	0.13 (0.03, 0.23)	6	0.09 (0.01, 0.17)	5	0.07 (0.01, 0.14)	5	0.07 (0.01, 0.14)

a Age-standardized prevalence rates of transposition of the great arteries were calculated using age groups according to the direct method using the estimated Korean population in 2015 as a reference.

Supplementary Table 1-15. Age-standardized prevalence[a] and 95% confidence interval (CI) of Eisenmenger syndrome overall and by sex (per 100,000).

Variables	2006		2007		2008		2009		2010	
	n	Prevalence (95% CI)	n	Prevalence (95% CI)	n	Prevalence (95% CI)	n	Prevalence (95% CI)	n	Prevalence (95% CI)
All	500	1.26 (1.14, 1.37)	622	1.53 (1.41, 1.66)	583	1.39 (1.28, 1.51)	572	1.29 (1.18, 1.40)	667	1.46 (1.35, 1.58)
Adults	386	1.38 (1.24, 1.52)	502	1.72 (1.56, 1.87)	496	1.60 (1.46, 1.75)	503	1.51 (1.37, 1.64)	583	1.69 (1.55, 1.83)
0–4 years old	50	2.19 (1.57, 2.81)	55	2.41 (1.77, 3.06)	48	2.14 (1.53, 2.75)	25	1.11 (0.67, 1.55)	27	1.16 (0.71, 1.61)
5–9 years old	25	0.79 (0.47, 1.12)	20	0.71 (0.39, 1.02)	13	0.44 (0.17, 0.70)	15	0.57 (0.28, 0.87)	12	0.48 (0.20, 0.77)
10–14 years old	20	0.57 (0.31, 0.83)	25	0.74 (0.44, 1.04)	14	0.41 (0.18, 0.64)	11	0.33 (0.12, 0.53)	19	0.57 (0.30, 0.85)
15–19 years old	19	0.59 (0.31, 0.88)	20	0.63 (0.35, 0.91)	12	0.34 (0.13, 0.55)	18	0.53 (0.28, 0.78)	26	0.75 (0.46, 1.05)
20–44 years old	131	0.64 (0.53, 0.75)	155	0.76 (0.64, 0.88)	169	0.84 (0.71, 0.97)	203	1.02 (0.87, 1.16)	237	1.20 (1.05, 1.35)
45–64 years old	105	0.91 (0.74, 1.09)	142	1.19 (0.99, 1.39)	134	1.09 (0.90, 1.27)	158	1.24 (1.04, 1.43)	188	1.42 (1.21, 1.62)
Over 65 years	150	3.66 (3.07, 4.25)	205	4.65 (4.01, 5.29)	193	4.18 (3.59, 4.77)	142	2.93 (2.45, 3.42)	158	3.16 (2.67, 3.66)
Women	267	0.67 (0.59, 0.76)	325	0.81 (0.72, 0.90)	330	0.79 (0.70, 0.88)	309	0.69 (0.61, 0.77)	394	0.86 (0.78, 0.95)
Adults	210	0.75 (0.65, 0.86)	279	0.95 (0.84, 1.06)	291	0.94 (0.83, 1.05)	280	0.83 (0.73, 0.92)	354	1.02 (0.91, 1.13)
0–4 years old	26	1.11 (6.71, 1.56)	20	0.84 (0.46, 1.23)	21	0.93 (0.53, 1.34)	11	0.49 (0.20, 0.78)	15	0.62 (0.29, 0.96)
5–9 years old	13	0.39 (0.16, 0.63)	6	0.17 (0.01, 034)	6	0.22 (0.04, 0.40)	5	0.17 (0.01, 0.34)	6	0.22 (0.02, 0.42)
10–14 years old	10	0.28 (0.10, 0.47)	13	0.37 (0.15, 0.58)	6	0.16 (0.01, 0.31)	4	0.12 (0.01, 0.24)	8	0.24 (0.07, 0.42)
15–19 years old	8	0.25 (0.06, 0.43)	7	0.22 (0.05, 0.38)	6	0.15 (0.01, 0.30)	9	0.25 (0.07, 0.43)	11	0.31 (0.12, 0.50)
20–44 years old	73	0.35 (0.27, 0.43)	91	0.44 (0.35, 0.54)	104	0.51 (0.41, 0.61)	126	0.63 (0.52, 0.74)	157	0.79 (0.67, 0.92)
45–64 years old	48	0.41 (0.30, 0.53)	70	0.58 (0.44, 0.72)	67	0.54 (0.41, 0.67)	80	0.62 (0.48, 0.76)	96	0.72 (0.57, 0.87)
Over 65 years	89	2.17 (1.72, 2.63)	118	2.67 (2.19, 3.16)	120	2.60 (2.13, 3.06)	74	1.52 (1.17, 1.87)	101	2.02 (1.62, 2.42)
Men	233	0.58 (0.50, 0.65)	297	0.72 (0.64, 0.81)	253	0.60 (0.52, 0.67)	263	0.59 (0.52, 0.67)	273	0.59 (0.52, 0.67)
Adults	176	0.62 (0.53, 0.72)	223	0.76 (0.66, 0.87)	205	0.66 (0.57, 1.64)	223	0.68 (0.59, 0.77)	229	0.67 (0.58, 0.75)
0–4 years old	24	1.02 (0.59, 1.45)	35	1.52 (1.00, 2.03)	27	1.20 (0.75, 1.66)	14	0.62 (0.29, 0.95)	12	0.49 (0.19, 0.79)
5–9 years old	12	0.39 (0.17, 0.62)	14	0.48 (0.22, 0.74)	7	0.22 (0.02, 0.41)	10	0.35 (0.11, 0.59)	6	0.22 (0.02, 0.42)
10–14 years old	10	0.28 (0.10, 0.47)	12	0.33 (0.12, 0.53)	8	0.20 (0.03, 0.37)	7	0.20 (0.04, 0.36)	11	0.33 (0.12, 0.53)
15–19 years old	11	0.34 (0.13, 0.56)	13	0.41 (0.18, 0.63)	6	0.15 (0.01, 0.30)	9	0.25 (0.07, 0.43)	15	0.44 (0.21, 0.66)
20–44 years old	58	0.28 (0.20, 0.35)	64	0.31 (0.23, 0.39)	65	0.32 (0.24, 0.40)	77	0.38 (0.30, 0.47)	80	0.40 (0.31, 0.49)
45–64 years old	57	0.49 (0.36, 0.62)	72	0.60 (0.46, 0.74)	67	0.54 (0.41, 0.67)	78	0.61 (0.47, 0.74)	92	0.69 (0.55, 0.84)
Over 65 years	61	1.49 (1.11, 1.86)	87	1.97 (1.56, 2.39)	73	1.58 (1.21, 1.94)	68	1.40 (1.06. 1.73)	57	1.14 (0.84, 1.43)

Cont. Suppl. Table 1-15.

Variables	2011		2012		2013		2014		2015	
	n	Prevalence (95% CI)	n	Prevalence (95% CI)	n	Prevalence (95% CI)	n	Prevalence (95% CI)	n	Prevalence (95% CI)
All	756	1.61 (1.49, 1.72)	744	1.54 (1.42, 1.65)	748	1.52 (1.41, 1.63)	794	1.58 (1.47, 1.69)	822	1.61 (1.50, 1.72)
Adults	654	1.82 (1.68, 1.96)	645	1.72 (1.59, 1.85)	673	1.74 (1.61, 1.88)	729	1.82 (1.69, 1.96)	744	1.81 (1.68, 1.95)
0–4 years old	40	1.69 (1.16, 2.23)	40	1.69 (1.15, 2.24)	23	0.98 (0.57, 1.39)	17	0.71 (0.36, 1.07)	28	1.20 (0.74, 1.66)
5–9 years old	15	0.62 (0.29, 0.95)	13	0.53 (0.22, 0.83)	9	0.35 (0.09, 0.61)	8	0.31 (0.06, 0.55)	8	0.31 (0.06, 0.55)
10–14 years old	19	0.62 (0.33, 0.90)	17	0.57 (0.29, 0.86)	15	0.53 (0.25, 0.81)	15	0.57 (0.28, 0.87)	19	0.78 (0.42, 1.14)
15–19 years old	28	0.82 (0.51, 1.12)	29	0.85 (0.53, 1.16)	28	0.85 (0.53, 1.16)	25	0.75 (0.44, 1.06)	23	0.72 (0.42, 1.02)
20–44 years old	266	1.35 (1.19, 1.52)	271	1.39 (1.22, 1.55)	258	1.33 (1.17, 1.50)	276	1.44 (1.27, 1.61)	284	1.49 (1.32, 1.67)
45–64 years old	224	1.64 (1.42, 1.85)	232	1.65 (1.44, 1.86)	254	1.75 (1.53, 1.97)	277	1.85 (1.63, 2.07)	269	1.75 (1.54, 1.96)
Over 65 years	164	3.15 (2.66, 3.63)	142	2.58 (2.16, 3.01)	161	2.80 (2.36, 3.23)	176	2.92 (2.48, 3.35)	191	3.05 (2.62, 3.49)
Women	431	0.92 (0.83, 1.01)	431	0.89 (0.80, 0.97)	424	0.86 (0.78, 0.94)	462	0.92 (0.83, 1.00)	488	0.95 (0.87, 1.04)
Adults	380	1.06 (0.95, 1.16)	375	1.00 (0.90, 1.10)	384	0.99 (0.89, 1.09)	434	1.08 (0.98, 1.19)	456	1.11 (1.01, 1.21)
0–4 years old	24	1.02 (0.61, 1.44)	25	1.07 (0.64, 1.50)	14	0.58 (0.25, 0.90)	4	0.13 (0.00, 0.30)	11	0.44 (0.15, 0.73)
5–9 years old	9	0.35 (0.09, 0.61)	6	0.22 (0.01, 0.43)	2	0.04 (0.00, 0.16)	3	0.08 (0.00, 0.23)	5	0.17 (0.00, 0.36)
10–14 years old	7	0.20 (0.03, 0.37)	11	0.37 (0.14, 0.60)	9	0.33 (0.11, 0.54)	7	0.24 (0.04, 0.44)	8	0.33 (0.09, 0.56)
15–19 years old	11	0.31 (0.12, 0.50)	14	0.41 (0.18, 0.63)	15	0.44 (0.20, 0.67)	14	0.41 (0.17, 0.64)	8	0.25 (0.07, 0.43)
20–44 years old	161	0.82 (0.69, 0.94)	155	0.79 (0.66, 0.91)	147	0.76 (0.63, 0.88)	163	0.84 (0.71, 0.97)	177	0.93 (0.79, 1.06)
45–64 years old	113	0.82 (0.67, 0.97)	132	0.93 (0.77, 1.09)	137	0.94 (0.78, 1.10)	160	1.07 (0.90, 1.23)	157	1.02 (0.86, 1.18)
Over 65 years	106	2.03 (1.65, 2.42)	88	1.59 (1.26, 1.93)	100	1.73 (1.39, 2.07)	111	1.84 (1.49, 2.18)	122	1.94 (1.60, 2.29)
Men	325	0.68 (0.61, 0.76)	313	0.64 (0.57, 0.71)	324	0.65 (0.58, 0.73)	332	0.65 (0.58, 0.72)	334	0.65 (0.58, 0.72)
Adults	274	0.76 (0.67, 0.85)	270	0.71 (0.63, 0.80)	289	0.74 (0.66, 0.83)	295	0.73 (0.65, 0.82)	288	0.70 (0.62, 0.78)
0–4 years old	16	0.67 (0.33, 1.01)	15	0.62 (0.59, 0.95)	9	0.35 (0.09, 0.61)	13	0.53 (0.22, 0.84)	17	0.71 (0.35, 1.07)
5–9 years old	6	0.22 (0.01, 0.43)	7	0.26 (0.04, 0.49)	7	0.26 (0.03, 0.49)	5	0.17 (0.00, 0.37)	3	0.08 (0.00, 0.23)
10–14 years old	12	0.37 (0.14, 0.59)	6	0.20 (0.03, 0.37)	6	0.20 (0.02, 0.38)	8	0.28 (0.07, 0.50)	11	0.45 (0.18, 0.72)
15–19 years old	17	0.50 (0.26, 0.74)	15	0.44 (0.21, 0.66)	13	0.37 (0.16, 0.59)	11	0.31 (0.11, 0.51)	15	0.47 (0.22, 0.71)
20–44 years old	105	0.53 (0.42, 0.63)	116	0.59 (0.48, 0.70)	111	0.57 (0.46, 0.67)	113	0.58 (0.47, 0.69)	107	0.56 (0.45, 0.66)
45–64 years old	111	0.81 (0.65, 0.96)	100	0.71 (0.57, 0.85)	117	0.80 (0.65, 0.95)	117	0.78 (0.64, 0.92)	112	0.72 (0.58, 0.86)
Over 65 years	58	1.11 (0.82, 1.39)	54	0.97 (0.71, 1.23)	61	1.05 (0.78, 1.31)	65	1.08 (0.81, 1.34)	69	1.09 (0.83, 1.35)

a Age-standardized prevalence rates of Eisenmenger syndrome were calculated using age groups according to the direct method using the estimated Korean population in 2015 as a reference.

Supplementary Table 1-15-1. Age-standardized prevalence[a] and 95% confidence interval (CI) of pulmonary hypertension (I27.0 and I27.2) overall and by sex (per 100,000).

Variables	2006		2007		2008		2009		2010	
	n	Prevalence (95% CI)	n	Prevalence (95% CI)	n	Prevalence (95% CI)	n	Prevalence (95% CI)	n	Prevalence (95% CI)
All	1,026	2.69 (2.52, 2.85)	1,269	3.18 (3.00, 3.36)	1,540	3.70 (3.51, 3.89)	1,636	3.81 (3.62, 4.00)	1,682	3.82 (3.63, 4.00)
Adults	894	3.32 (3.10, 3.55)	1,088	3.81 (3.58, 4.04)	1,285	4.25 (4.02, 4.49)	1,359	4.29 (4.06, 4.52)	1,364	4.14 (3.91, 4.36)
0–4 years old	67	2.95 (2.23, 3.66)	110	4.87 (3.96, 5.78)	171	7.64 (6.50, 8.79)	179	8.00 (6.83, 9.18)	220	9.66 (8.38, 10.9)
5–9 years old	15	0.48 (0.23, 0.74)	18	0.62 (0.32, 0.91)	24	0.88 (0.52, 1.24)	32	1.19 (0.76, 1.62)	31	1.28 (0.83, 1.74)
10–14 years old	20	0.57 (0.31, 0.83)	21	0.62 (0.34, 0.89)	20	0.57 (0.30, 0.85)	29	0.90 (0.57, 1.24)	26	0.82 (0.50, 1.14)
15–19 years old	30	0.97 (0.62, 1.33)	32	1.00 (0.65, 1.36)	40	1.23 (0.84, 1.61)	37	1.10 (0.74, 1.46)	41	1.19 (0.82, 0.82)
20–44 years old	206	1.00 (0.87, 1.14)	257	1.27 (1.11, 1.43)	328	1.64 (1.46, 1.81)	367	1.85 (1.66, 2.04)	368	1.87 (1.68, 2.06)
45–64 years old	337	2.94 (2.63, 3.26)	394	3.32 (2.99, 3.65)	475	3.87 (3.52, 4.22)	464	3.65 (3.31, 3.98)	494	3.75 (3.42, 4.08)
Over 65 years	351	8.61 (7.71, 9.51)	437	9.95 (9.02, 10.8)	482	10.4 (9.53, 11.4)	528	10.9 (9.99, 11.8)	502	10.0 (9.19, 10.9)
Women	668	3.51 (3.24, 3.78)	805	4.07 (3.78, 4.36)	960	4.66 (4.36, 4.96)	1,044	4.93 (4.63, 5.24)	1,092	5.03 (4.73, 5.33)
Adults	602	2.22 (2.04, 2.41)	720	2.51 (2.32, 2.70)	848	2.78 (2.59, 2.97)	914	2.88 (2.69, 3.07)	946	2.86 (2.68, 3.05)
0–4 years old	26	2.38 (1.45, 3.31)	44	4.04 (2.84, 5.24)	65	5.97 (4.50, 7.43)	76	6.98 (5.40, 8.56)	94	8.54 (6.81, 10.2)
5–9 years old	10	0.64 (0.20, 1.07)	12	0.85 (0.31, 1.33)	14	1.00 (0.43, 1.58)	17	1.28 (0.63, 1.93)	14	1.19 (0.55, 1.83)
10–14 years old	12	0.68 (0.25, 1.12)	11	0.68 (0.27, 1.10)	13	0.77 (0.31, 1.23)	19	1.20 (0.64, 1.77)	19	1.20 (0.63, 1.78)
15–19 years old	18	1.25 (0.67, 1.83)	18	1.18 (0.62, 1.75)	20	1.32 (0.73, 1.90)	18	1.12 (0.58, 1.66)	19	1.18 (0.64, 1.73)
20–44 years old	148	1.49 (1.25, 1.73)	178	1.81 (1.54, 2.08)	233	2.39 (2.09, 2.70)	251	2.60 (2.28, 2.92)	261	2.74 (2.41, 3.07)
45–64 years old	218	3.80 (3.29, 4.30)	256	4.32 (3.79, 4.85)	309	5.05 (4.48, 5.61)	302	4.77 (4.23, 5.31)	336	5.12 (4.57, 5.66)
Over 65 years	236	9.80 (8.54, 11.0)	286	11.1 (9.82, 12.4)	306	11.3 (10.1, 12.6)	361	12.8 (11.4, 14.1)	349	12.0 (10.7, 13.2)
Men	358	1.87 (1.67, 2.06)	464	2.29 (2.08, 2.50)	580	2.76 (2.53, 2.99)	592	2.70 (2.48, 2.92)	590	2.62 (2.40, 2.83)
Adults	292	1.09 (0.96, 1.22)	368	1.28 (1.14, 1.41)	437	1.46 (1.33, 1.60)	445	1.40 (1.27, 1.53)	418	1.27 (1.14, 1.39)
0–4 years old	41	3.48 (2.40, 4.56)	66	5.57 (4.21, 6.94)	106	9.15 (7.40, 10.9)	103	8.89 (7.16, 10.6)	126	10.7 (8.84, 12.6)
5–9 years old	5	0.25 (0.00, 0.53)	6	0.34 (0.01, 0.67)	10	0.68 (0.24, 1.13)	15	1.03 (0.46, 1.59)	17	1.29 (0.64, 1.94)
10–14 years old	8	0.39 (0.08, 0.70)	10	0.55 (0.20, 0.91)	7	0.39 (0.09, 0.70)	10	0.55 (0.18, 0.92)	7	0.39 (0.07, 0.71)
15–19 years old	12	0.72 (0.29, 1.14)	14	0.78 (0.34, 1.22)	20	1.14 (0.63, 1.65)	19	1.02 (0.53, 1.51)	22	1.20 (0.69, 1.71)
20–44 years old	58	0.54 (0.40, 0.69)	79	0.76 (0.59, 0.93)	95	0.92 (0.73, 1.10)	116	1.12 (0.92, 1.33)	107	1.05 (0.85, 1.25)
45–64 years old	119	2.07 (1.70, 2.45)	138	2.32 (1.93, 2.71)	166	2.70 (2.28, 3.11)	162	2.54 (2.14, 2.93)	158	2.38 (2.00, 2.75)
Over 65 years	115	6.87 (5.61, 8.13)	151	8.28 (6.96, 9.61)	176	9.15 (7.79, 10.5)	167	8.28 (7.02, 9.54)	153	7.30 (6.14, 8.47)

Cont. Suppl. Table 1-15-1.

Variables	2011		2012		2013		2014		2015	
	n	Prevalence (95% CI)	n	Prevalence (95% CI)	n	Prevalence (95% CI)	n	Prevalence (95% CI)	n	Prevalence (95% CI)
All	1,995	4.48 (4.28, 4.68)	2,076	4.52 (4.33, 4.72)	2,090	4.45 (4.26, 4.64)	2,078	4.33 (4.14, 4.51)	2,144	4.39 (4.20, 4.58)
Adults	1,677	4.92 (4.69, 5.16)	1,715	4.80 (4.57, 5.02)	1,762	4.72 (4.49, 4.94)	1,788	4.58 (4.36, 4.79)	1,871	4.64 (4.43, 4.85)
0–4 years old	211	9.26 (8.00, 10.5)	230	10.1 (8.79, 11.4)	197	8.67 (7.46, 9.89)	174	7.64 (6.50, 8.78)	153	6.71 (5.64, 7.77)
5–9 years old	39	1.59 (1.08, 2.10)	56	2.30 (1.69, 2.92)	48	1.99 (1.42, 2.56)	50	2.08 (1.50, 2.66)	51	2.13 (1.54, 2.71)
10–14 years old	30	0.95 (0.60, 1.29)	36	1.11 (0.73, 1.49)	41	1.28 (0.87, 1.68)	29	0.90 (0.57, 1.24)	31	0.99 (0.64, 1.34)
15–19 years old	38	1.10 (0.74, 1.46)	39	1.13 (0.77, 1.49)	42	1.23 (0.85, 1.60)	37	1.07 (0.71, 1.42)	38	1.10 (0.74, 1.46)
20–44 years old	416	2.11 (1.91, 2.32)	425	2.16 (1.95, 2.36)	437	2.22 (2.01, 2.43)	473	2.40 (2.19, 2.62)	458	2.33 (2.11, 2.54)
45–64 years old	564	4.28 (3.92, 4.63)	602	4.56 (4.20, 4.93)	604	4.58 (4.21, 4.94)	605	4.58 (4.22, 4.95)	668	5.06 (4.68, 5.45)
Over 65 years	697	13.9 (12.9, 15.0)	688	13.8 (12.7, 14.8)	721	14.4 (13.4, 15.5)	710	14.2 (13.1, 15.2)	745	14.9 (13.8, 16.0)
Women	1,326	6.04 (5.71, 6.37)	1,374	6.07 (5.75, 6.39)	1,389	6.00 (5.68, 6.32)	1,392	5.88 (5.57, 6.19)	1,449	6.03 (5.72, 6.34)
Adults	1,172	3.44 (3.24, 3.63)	1,206	3.36 (3.17, 3.55)	1,231	3.28 (3.10, 3.47)	1,244	3.18 (3.00. 3.35)	1,307	3.24 (3.06, 3.41)
0–4 years old	100	8.91 (7.15, 10.6)	100	8.91 (7.15, 10.6)	92	8.27 (6.57, 9.96)	90	8.08 (6.40, 9.76)	80	7.25 (5.65, 8.85)
5–9 years old	17	1.46 (0.73, 2.20)	23	2.01 (1.16, 2.86)	22	1.92 (1.09, 2.75)	20	1.74 (0.94, 2.54)	19	1.65 (0.88, 2.41)
10–14 years old	21	1.37 (0.75, 2.00)	19	1.37 (0.75, 2.00)	20	1.46 (0.78, 2.14)	14	1.12 (0.52, 1.71)	20	1.72 (0.95, 2.49)
15–19 years old	16	0.99 (0.49, 1.48)	26	1.65 (1.01, 2.29)	24	1.52 (0.89, 2.14)	24	1.52 (0.88, 2.15)	23	1.52 (0.88, 2.15)
20–44 years old	303	3.19 (2.83, 3.55)	318	3.37 (3.00, 3.74)	326	3.50 (3.12, 3.88)	345	3.74 (3.34, 4.13)	333	3.66 (3.26, 4.05)
45–64 years old	378	5.58 (5.02, 6.14)	410	5.87 (5.30, 6.44)	415	5.75 (5.20, 6.31)	418	5.61 (5.07, 6.15)	457	5.97 (5.42, 6.52)
Over 65 years	491	16.3 (14.8, 17.7)	478	15.1 (13.7, 16.4)	490	14.8 (13.5, 16.1)	481	13.9 (12.7, 15.2)	517	14.5 (13.2, 15.7)
Men	669	2.94 (2.72, 3.17)	702	3.00 (2.78, 3.22)	701	2.94 (2.72, 3.16)	686	2.81 (2.60, 3.02)	695	2.80 (2.59, 3.01)
Adults	505	1.48 (1.35, 1.61)	509	1.43 (1.30, 1.55)	531	1.43 (1.31, 1.55)	544	1.39 (1.27, 1.51)	564	1.39 (1.28, 1.51)
0–4 years old	111	9.32 (7.58, 11.0)	130	10.9 (9.09, 12.8)	105	8.89 (7.17, 10.6)	84	7.14 (5.61, 8.68)	73	6.27 (4.82, 7.72)
5–9 years old	22	1.80 (1.03, 2.57)	33	2.75 (1.80, 3.69)	26	2.15 (1.30, 2.99)	30	2.49 (1.57, 3.41)	32	2.66 (1.72, 3.60)
10–14 years old	9	0.55 (0.18, 0.93)	17	1.11 (0.56, 1.65)	21	1.43 (0.79, 2.06)	15	1.11 (0.54, 1.67)	11	0.87 (0.34, 1.39)
15–19 years old	22	1.20 (0.69, 1.72)	13	0.72 (0.32, 1.12)	18	1.02 (0.54, 1.50)	13	0.72 (0.30, 1.14)	15	0.90 (0.43, 1.37)
20–44 years old	113	1.11 (0.90, 1.32)	107	1.06 (0.86, 1.26)	111	1.10 (0.89, 1.31)	128	1.28 (1.06, 1.51)	125	1.26 (1.04, 1.48)
45–64 years old	186	2.71 (2.32, 3.10)	192	2.72 (2.34, 3.11)	189	2.60 (2.23, 2.97)	187	2.50 (2.14, 2.86)	211	2.74 (2.36, 3.11)
Over 65 years	206	9.44 (8.15, 10.7)	210	9.08 (7.85, 10.3)	231	9.44 (8.22, 10.6)	229	8.90 (7.74, 10.0)	228	8.50 (7.39, 9.61)

a Age-standardized prevalence rates of pulmonary hypertension (I27.0 primary pulmonary hypertension and I27.2 other secondary pulmonary hypertension) were calculated using age groups according to the direct method using the estimated Korean population in 2015 as a reference.

Supplementary Table 1-16. Age-standardized prevalence[a] and 95% confidence interval (CI) of double outlet right ventricle overall and by sex (per 100,000).

Variables	2006		2007		2008		2009		2010	
	n	Prevalence (95% CI)	n	Prevalence (95% CI)	n	Prevalence (95% CI)	n	Prevalence (95% CI)	n	Prevalence (95% CI)
All	584	1.10 (1.01, 1.19)	664	1.25 (1.15, 1.34)	712	1.33 (1.24, 1.43)	770	1.44 (1.34, 1.54)	822	1.54 (1.44, 1.65)
Adults	75	0.19 (0.15, 0.24)	90	0.23 (0.18, 0.28)	101	0.25 (0.20, 0.30)	120	0.30 (0.24, 0.35)	140	0.34 (0.29, 0.40)
0–4 years old	229	10.1 (8.87, 11.5)	243	10.7 (9.42, 12.1)	237	10.6 (9.25, 11.9)	245	10.9 (9.58, 12.3)	250	11.0 (9.63, 12.3)
5–9 years old	135	4.48 (3.72, 5.24)	163	5.77 (4.88, 6.66)	170	6.34 (5.39, 7.30)	170	6.56 (5.58, 7.55)	168	7.01 (5.95, 8.07)
10–14 years old	94	2.77 (2.20 (3.33)	102	3.05 (2.46, 3.65)	130	4.01 (3.31, 4.70)	140	4.38 (3.65, 5.11)	163	5.21 (4.40, 6.01)
15–19 years old	51	1.67 (1.20, 2.13)	66	2.08 (1.57, 2.58)	74	2.30 (1.77, 2.82)	95	2.87 (2.29, 3.44)	101	2.99 (2.41, 3.58)
20–44 years old	65	0.31 (0.23, 0.39)	76	0.37 (0.29, 0.46)	91	0.45 (0.36, 0.54)	109	0.54 (0.44, 0.65)	126	0.63 (0.52, 0.75)
45–64 years old	9	0.07 (0.02, 0.12)	12	0.09 (0.04, 0.15)	8	0.05 (0.01, 0.10)	9	0.06 (0.02, 0.11)	12	0.08 (0.03, 0.13)
Over 65 years	1	0.01 (0.00, 0.06)	2	0.03 (0.00, 0.09)	2	0.03 (0.00, 0.09)	2	0.03 (0.00, 0.08)	2	0.03 (0.00, 0.08)
Women	223	0.41 (0.36, 0.47)	252	0.47 (0.41, 0.53)	280	0.52 (0.46, 0.58)	308	0.57 (0.51, 0.64)	314	0.59 (0.52, 0.65)
Adults	34	0.09 (0.06, 0.12)	38	0.10 (0.06, 0.13)	39	0.10 (0.06, 0.13)	46	0.11 (0.08, 0.14)	53	0.13 (0.09, 0.16)
0–4 years old	80	3.53 (2.75, 4.31)	92	4.07 (3.23, 4.90)	99	4.42 (3.55, 5.30)	98	4.38 (3.51, 5.25)	92	4.02 (3.19, 4.85)
5–9 years old	49	1.59 (1.13, 2.05)	55	1.95 (1.43, 2.46)	60	2.21 (1.65, 2.78)	62	2.35 (1.75, 2.94)	65	2.70 (2.04, 3.36)
10–14 years old	43	1.24 (0.85, 1.62)	47	1.40 (0.99, 1.81)	57	1.73 (1.27, 2.19)	62	1.94 (1.45, 2.42)	61	1.94 (1.45, 2.43)
15–19 years old	17	0.53 (0.26, 0.80)	20	0.63 (0.35, 0.91)	25	0.75 (0.45, 1.06)	40	1.19 (0.82, 1.57)	43	1.26 (0.87, 1.64)
20–44 years old	28	0.13 (0.08, 0.18)	31	0.14 (0.09, 0.20)	34	0.16 (0.10, 0.22)	41	0.20 (0.14, 0.26)	46	0.23 (0.16, 0.30)
45–64 years old	5	0.03 (0.01, 0.07)	5	0.03 (0.01, 0.07)	3	0.01 (0.00, 0.04)	3	0.01 (0.00, 0.04)	5	0.03 (0.00, 0.06)
Over 65 years	1	0.01 (0.00, 0.06)	2	0.03 (0.00, 0.09)	2	0.03 (0.02, 0.09)	2	0.03 (0.00, 0.08)	2	0.03 (0.00, 0.08)
Men	361	0.68 (0.61, 0.75)	412	0.77 (0.70, 0.85)	432	0.81 (0.73, 0.88)	462	0.86 (0.78, 0.94)	508	0.95 (0.87,1.03)
Adults	41	0.10 (0.07, 0.13)	52	0.13 (0.09, 0.17)	62	0.15 (0.11, 0.19)	74	0.18 (0.14, 0.22)	87	0.21 (0.16, 0.25)
0–4 years old	149	6.62 (5.55, 7.68)	151	6.66 (5.59, 7.73)	138	6.17 (5.14, 7.20)	147	6.57 (5.51, 7.64)	158	6.93 (5.84, 8.02)
5–9 years old	86	2.84 (2.23, 3.44)	108	3.81 (3.09, 4.54)	110	4.08 (3.31, 4.85)	108	4.17 (3.38, 4.95)	103	4.26 (3.42, 5.09)
10–14 years old	51	1.48 (1.07, 1.90)	55	1.65 (1.21, 2.09)	73	2.23 (1.71, 2.75)	78	2.43 (1.89, 2.98)	102	3.26 (2.63, 3.90)
15–19 years old	34	1.10 (0.72, 1.48)	46	1.45 (1.02, 1.87)	49	1.51 (1.08, 1.94)	55	1.64 (1.19, 2.08)	58	1.70 (1.25, 2.14)
20–44 years old	37	0.17 (0.11, 0.23)	45	0.22 (0.15, 0.28)	57	0.28 (0.20, 0.35)	68	0.33 (0.25, 0.42)	80	0.40 (0.31, 0.49)
45–64 years old	4	0.03 (0.00, 0.06)	7	0.05 (0.01, 0.09)	5	0.03 (0.01, 0.07)	6	0.04 (0.01, 0.08)	7	0.04 (0.01, 0.08)
Over 65 years	0	0.00 (0.00, 0.00)	0	0.00 (0.00, 0.00)	0	0.00 (0.00, 0.00)	0	0.00 (0.00, 0.00)	0	0.00 (0.00, 0.00)

Cont. Suppl. Table 1-16.

Variables	2011		2012		2013		2014		2015	
	n	Prevalence (95% CI)	n	Prevalence (95% CI)	n	Prevalence (95% CI)	n	Prevalence (95% CI)	n	Prevalence (95% CI)
All	907	1.71 (1.60, 1.82)	892	1.69 (1.58, 1.80)	921	1.76 (1.65, 1.88)	991	1.91 (1.79, 2.03)	1,049	2.05 (1.93, 2.18)
Adults	153	0.37 (0.31, 0.43)	164	0.39 (0.33, 0.45)	171	0.40 (0.34, 0.46)	182	0.43 (0.36, 0.49)	204	0.48 (0.41, 0.54)
0–4 years old	274	11.8 (10.4, 13.3)	292	12.7 (11.2, 14.1)	321	14.0 (12.5, 15.5)	356	15.6 (13.9, 17.2)	386	17.1 (15.4, 18.8)
5–9 years old	183	7.94 (6.78, 9.10)	169	7.32 (6.21, 8.43)	153	6.70 (5.63, 7.76)	160	7.05 (5.95, 8.15)	161	7.01 (5.92, 8.09)
10–14 years old	176	5.83 (4.96, 6.69)	147	5.12 (4.29, 5.96)	145	5.33 (4.46, 6.20)	144	5.54 (4.62, 6.45)	152	6.36 (5.35, 7.38)
15–19 years old	121	3.59 (2.95, 4.23)	120	3.59 (2.95, 4.24)	131	4.00 (3.31, 4.69)	149	4.66 (3.91, 5.41)	146	4.66 (3.90, 5.42)
20–44 years old	136	0.69 (0.57, 0.81)	149	0.76 (0.64, 0.88)	155	0.79 (0.67, 0.92)	162	0.84 (0.71, 0.97)	188	0.99 (0.85, 1.13)
45–64 years old	14	0.09 (0.04, 0.15)	12	0.07 (0.03, 0.12)	13	0.08 (0.03, 0.13)	17	0.11 (0.05, 0.16)	13	0.07 (0.03, 0.12)
Over 65 years	3	0.04 (0.00, 0.11)	3	0.04 (0.00, 0.10)	3	0.04 (0.00, 0.10)	3	0.04 (0.00, 0.10)	3	0.04 (0.00, 0.10)
Women	362	0.68 (0.61, 0.75)	354	0.67 (0.60, 0.74)	362	0.69 (0.62, 0.76)	384	0.74 (0.66, 0.81)	405	0.79 (0.71, 0.87)
Adults	68	0.16 (0.12, 0.21)	68	0.16 (0.12, 0.20)	75	0.17 (0.13, 0.22)	74	0.17 (0.13, 0.21)	86	0.20 (0.15, 0.24)
0–4 years old	109	4.69 (3.80, 5.58)	117	5.05 (4.13, 5.97)	128	5.59 (4.61, 6.56)	147	6.44 (5.39, 7.48)	157	6.97 (5.88, 8.07)
5–9 years old	69	2.97 (2.26, 3.68)	64	2.75 (2.07, 3.43)	63	2.75 (2.06, 3.43)	55	2.39 (1.75, 3.04)	56	2.44 (1.80, 3.08)
10–14 years old	63	2.06 (1.55, 2.58)	52	1.81 (1.32, 2.31)	47	1.69 (1.20, 2.19)	51	1.94 (1.40, 2.48)	51	2.10 (1.52, 2.69)
15–19 years old	53	1.54 (1.12, 1.96)	53	1.57 (1.14, 200)	49	1.48 (1.06, 1.90)	57	1.76 (1.30, 2.23)	55	1.76 (1.29, 2.23)
20–44 years old	58	0.29 (0.21, 0.37)	59	0.29 (0.22, 0.37)	64	0.32 (0.24, 0.40)	62	0.32 (0.24, 0.40)	76	0.39 (0.30, 0.48)
45–64 years old	7	0.04 (0.01, 0.08)	7	0.04 (0.01, 0.08)	8	0.05 (0.01, 0.09)	9	0.05 (0.02, 0.09)	7	0.03 (0.01, 0.07)
Over 65 years	3	0.04 (0.00, 0.11)	2	0.03 (0.00, 0.08)	3	0.04 (0.00, 0.10)	3	0.04 (0.00, 0.10)	3	0.04 (0.00, 0.10)
Men	545	1.02 (0.94, 1.11)	538	1.02 (0.93, 1.10)	559	1.07 (0.98, 1.15)	607	1.17 (1.08, 1.26)	644	1.26 (1.16, 1.36)
Adults	85	0.20 (0.16, 0.25)	96	0.23 (0.18, 0.27)	96	0.22 (0.18, 0.27)	108	0.25 (0.20, 0.30)	118	0.27 (0.22, 0.32)
0–4 years old	165	7.15 (6.06, 8.25)	175	7.60 (6.47, 8.73)	193	8.45 (7.25, 9.65)	209	9.17 (7.92, 10.4)	229	10.1 (8.83, 11.4)
5–9 years old	114	4.97 (4.05, 5.88)	105	4.52 (3.65, 5.40)	90	3.90 (3.09, 4.72)	105	4.61 (3.72, 5.50)	105	4.57 (3.69, 5.44)
10–14 years old	113	3.72 (3.02, 4.41)	95	3.30 (2.63, 3.97)	98	3.59 (2.88, 4.31)	93	3.59 (2.86, 4.33)	101	4.21 (3.38, 5.04)
15–19 years old	68	2.01 (1.53, 2.49)	67	1.98 (1.50, 2.46)	82	2.49 (1.94, 3.03)	92	2.87 (2.27, 3.46)	91	2.90 (2.30, 3.50)
20–44 years old	78	0.39 (0.30, 0.48)	90	0.46 (0.36, 0.55)	91	0.47 (0.37, 0.56)	100	0.52 (0.41, 0.62)	112	0.58 (0.47, 0.69)
45–64 years old	7	0.04 (0.01, 0.08)	5	0.03 (0.01, 0.06)	5	0.03 (0.01, 0.06)	8	0.05 (0.01, 0.09)	6	0.03 (0.01, 0.06)
Over 65 years	0	0.00 (0.00, 0.00)	1	0.01 (0.0, 0.05)	0	0.00 (0.00, 0.00)	0	0.00 (0.00, 0.00)	0	0.00 (0.00, 0.00)

a Age-standardized prevalence rates of double outlet right ventricle were calculated using age groups according to the direct method using the estimated Korean population in 2015 as a reference.

Supplementary Table 1-17. Age-standardized prevalence[a] and 95% confidence interval (CI) of single ventricle overall and by sex (per 100,000).

Variables	2006		2007		2008		2009		2010	
	n	Prevalence (95% CI)	n	Prevalence (95% CI)	n	Prevalence (95% CI)	n	Prevalence (95% CI)	n	Prevalence (95% CI)
All	347	0.63 (0.56, 0.70)	378	0.69 (0.62, 0.76)	468	0.85 (0.78, 0.93)	504	0.92 (0.84, 1.00)	617	1.13 (1.04, 1.22)
Adults	53	0.14 (0.10, 0.18)	50	0.12 (0.08, 0.15)	61	0.14 (0.11, 0.18)	72	0.17 (0.13, 0.21)	99	0.24 (0.19, 0.28)
0–4 years old	79	3.48 (2.71, 4.26)	93	4.11 (3.27, 4.95)	111	4.96 (4.04, 5.89)	108	4.83 (3.91, 5.74)	126	5.54 (4.57, 6.51)
5–9 years old	106	3.50 (2.83, 4.18)	96	3.37 (2.69, 4.05)	118	4.39 (3.59, 5.18)	115	4.43 (3.62, 5.25)	127	5.28 (4.35, 6.20)
10–14 years old	73	2.15 (1.65, 2.64)	86	2.60 (2.05, 3.15)	114	3.51 (2.86, 4.16)	134	4.17 (3.46, 4.89)	160	5.12 (4.33, 5.92)
15–19 years old	36	1.16 (0.77, 1.55)	53	1.67 (1.21, 2.12)	64	1.98 (1.49, 2.47)	75	2.27 (1.75, 2.78)	105	3.09 (2.49, 3.68)
20–44 years old	47	0.22 (0.16, 0.29)	48	0.23 (0.17, 0.30)	59	0.29 (0.21, 036)	71	0.35 (0.27, 0.43)	98	0.49 (0.40, 0.59)
45–64 years old	1	0.01 (0.00, 0.02)	1	0.01 (0.00, 0.02)	2	0.01 (0.00, 0.03)	0	0.00 (0.00, 0.00)	1	0.01 (0.00, 0.02)
Over 65 years	5	0.12 (0.01, 0.22)	1	0.01 (0.00, 0.05)	0	0.00 (0.00, 0.00)	1	0.01 (0.00, 0.05)	0	0.00 (0.00, 0.00)
Women	128	0.22 (0.18, 0.27)	141	0.25 (0.21, 0.30)	177	0.32 (0.27, 0.37)	186	0.33 (0.28, 0.38)	233	0.43 (0.37, 0.48)
Adults	25	0.06 (0.03, 0.09)	20	0.04 (0.02, 0.07)	30	0.07 (0.04, 0.10)	32	0.07 (0.05, 0.10)	48	0.11 (0.08, 0.14)
0–4 years old	22	0.93 (0.52, 1.35)	31	1.34 (0.85, 1.82)	31	1.38 (0.89, 1.87)	33	1.47 (0.97, 1.98)	52	2.28 (1.65, 2.90)
5–9 years old	40	1.33 (0.91, 1.74)	36	1.24 (0.82, 1.66)	40	1.46 (1.00, 1.92)	36	1.37 (0.92, 1.83)	37	1.50 (1.01, 2.00)
10–14 years old	29	0.82 (0.51, 1.14)	31	0.90 (0.57, 1.24)	45	1.36 (0.95, 1.77)	51	1.57 (1.13, 2.01)	56	1.77 (1.30, 2.24)
15–19 years old	12	0.37 (0.15, 0.60)	23	0.72 (0.42, 1.02)	31	0.94 (0.60, 1.26)	34	1.00 (0.66, 1.35)	40	1.16 (0.79, 1.53)
20–44 years old	23	0.11 (0.06, 0.15)	19	0.09 (0.05, 0.13)	28	0.13 (0.08, 0.19)	32	0.16 (0.10, 0.21)	48	0.24 (0.17, 0.31)
45–64 years old	0	0.00 (0.00, 0.00)	1	0.01 (0.00, 0.02)	2	0.01 (0.00, 0.03)	0	0.00 (0.00, 0.00)	0	0.00 (0.00, 0.00)
Over 65 years	2	0.04 (0.00, 0.11)	0	0.00 (0.00, 0.00)	0	0.00 (0.00, 0.00)	0	0.00 (0.00, 0.00)	0	0.00 (0.00, 0.00)
Men	219	0.40 (0.34, 0.45)	237	0.43 (0.37, 0.48)	291	0.53 (0.47, 0.59)	318	0.58 (0.51, 0.64)	384	0.70 (0.63, 0.77)
Adults	28	0.07 (0.04, 0.10)	30	0.07 (0.04, 0.10)	31	0.07 (0.04, 0.10)	40	0.09 (0.06, 0.12)	51	0.12 (0.09, 0.15)
0–4 years old	57	2.50 (1.84, 3.16)	62	2.72 (2.04, 3.41)	80	3.57 (2.79, 4.36)	75	3.35 (2.59, 4.11)	74	3.22 (2.47, 3.96)
5–9 years old	66	2.17 (1.64, 2.70)	60	2.13 (1.59, 2.67)	78	2.88 (2.23, 3.53)	79	3.01 (2.34, 3.69)	90	3.72 (2.95, 4.50)
10–14 years old	44	1.28 (0.89, 1.66)	55	1.65 (1.21, 2.09)	69	2.10 (1.60, 2.61)	83	2.60 (2.04, 3.16)	104	3.30 (2.66, 3.94)
15–19 years old	24	0.78 (0.47, 1.10)	30	0.94 (0.60, 1.28)	33	1.00 (0.65, 2.61)	41	1.23 (0.84, 1.61)	65	1.92 (1.45, 2.39)
20–44 years old	24	0.11 (0.06, 0.16)	29	0.14 (0.09, 0.19)	31	0.14 (0.09, 0.20)	39	0.19 (0.13, 0.25)	50	0.24 (0.17, 0.32)
45–64 years old	1	0.01 (0.00, 0.02)	0	0.00 (0.00, 0.00)	0	0.00 (0.00, 0.00)	0	0.00 (0.00, 0.00)	1	0.01 (0.00, 0.02)
Over 65 years	3	0.06 (0.00, 0.14)	1	0.01 (0.00, 0.05)	0	0.00 (0.00, 0.00)	1	0.01 (0.00, 0.05)	0	0.00 (0.00, 0.00)

Cont. Suppl. Table 1-17.

Variables	2011		2012		2013		2014		2015	
	n	Prevalence (95% CI)	n	Prevalence (95% CI)	n	Prevalence (95% CI)	n	Prevalence (95% CI)	n	Prevalence (95% CI)
All	791	1.47 (1.36, 1.57)	863	1.62 (1.51, 1.72)	887	1.68 (1.57, 1.79)	974	1.87 (1.75, 1.99)	1,038	2.03 (1.91, 2.16)
Adults	140	0.33 (0.28, 0.39)	181	0.43 (0.37, 0.49)	182	0.43 (0.36, 0.49)	229	0.53 (0.46, 0.60)	277	0.65 (0.57, 0.72)
0–4 years old	157	6.79 (5.73, 7.86)	180	7.82 (6.68, 8.97)	199	8.72 (7.50, 9.93)	219	9.57 (8.29, 10.8)	222	9.84 (8.54, 11.1)
5–9 years old	146	6.34 (5.31, 7.38)	141	6.12 (5.11, 7.13)	130	5.68 (4.70, 6.66)	133	5.85 (4.85, 6.85)	111	4.83 (8.54, 11.1)
10–14 years old	199	6.61 (5.69, 7.53)	205	7.19 (6.20, 8.18)	193	7.07 (6.06, 8.07)	185	7.15 (6.12, 8.18)	185	7.77 (6.65, 8.89)
15–19 years old	149	4.41 (3.70, 5.12)	156	4.66 (3.93, 5.40)	183	5.58 (4.77, 6.39)	208	6.52 (5.64, 7.41)	243	7.79 (6.80, 8.77)
20–44 years old	135	0.68 (0.57, 0.80)	174	0.89 (0.76, 1.02)	178	0.92 (0.78, 1.05)	223	1.16 (1.01, 1.31)	269	1.42 (1.25, 1.59)
45–64 years old	4	0.02 (0.00, 0.05)	5	0.03 (0.01, 0.06)	3	0.01 (0.00, 0.04)	4	0.02 (0.01, 0.05)	7	0.03 (0.01, 0.07)
Over 65 years	1	0.01 (0.02, 0.05)	2	0.03 (0.00, 0.08)	1	0.01 (0.00, 0.04)	2	0.03 (0.00, 0.07)	1	0.01 (0.00, 0.04)
Women	306	0.56 (0.50, 0.62)	339	0.63 (0.56, 0.70)	366	0.69 (0.62, 0.76)	410	0.78 (0.71, 0.86)	437	0.85 (0.77, 0.93)
Adults	57	0.13 (0.1, 0.17)	74	0.17 (0.13, 0.21)	83	0.19 (0.15, 0.23)	103	0.24 (0.19, 0.28)	114	0.26 (0.21, 0.31)
0–4 years old	62	2.68 (2.01, 3.35)	72	3.13 (2.40, 3.85)	86	3.75 (2.95, 4.55)	98	4.29 (3.44, 5.14)	98	4.33 (3.47, 5.20)
5–9 years old	45	1.95 (1.37, 2.52)	43	1.86 (1.30, 2.42)	46	1.99 (1.41, 2.58)	53	2.30 (1.67, 2.93)	45	1.95 (1.37, 2.52)
10–14 years old	85	2.81 (2.21, 3.41)	83	2.89 (2.26, 3.52)	79	2.89 (2.25, 3.53)	69	2.64 (2.01, 3.27)	66	2.77 (2.10, 3.43)
15–19 years old	57	1.67 (1.23, 2.11)	67	1.98 (1.50, 2.46)	72	2.17 (1.66, 2.68)	87	2.71 (2.13, 3.28)	114	3.65 (2.98, 4.33)
20–44 years old	55	0.27 (0.20, 0.35)	72	0.36 (0.28, 0.45)	83	0.42 (0.33, 0.51)	101	0.52 (0.42, 0.63)	112	0.58 (0.47, 0.69)
45–64 years old	1	0.01 (0.00, 0.02)	1	0.01 (0.00, 0.02)	0	0.00 (0.00, 0.00)	1	0.01 (0.00, 0.02)	2	0.01 (0.00, 0.02)
Over 65 years	1	0.01 (0.00, 0.05)	1	0.01 (0.00, 0.05)	0	0.00 (0.00, 0.00)	1	0.01 (0.00, 0.04)	0	0.00 (0.00, 0.00)
Men	485	0.90 (0.82, 0.98)	524	0.98 (0.90, 1.06)	521	0.99 (0.90, 1.07)	564	1.08 (0.99, 1.17)	601	1.17 (1.08, 1.27)
Adults	83	0.20 (0.15, 0.24)	107	0.25 (0.20, 0.30)	99	0.23 (0.18, 0.28)	126	0.29 (0.24, 0.34)	163	0.38 (0.32, 0.44)
0–4 years old	95	4.11 (3.28, 4.94)	108	4.69 (3.80, 5.58)	113	4.92 (4.00, 5.83)	121	5.27 (4.33, 6.22)	124	5.50 (4.53, 6.47)
5–9 years old	101	4.39 (3.53, 5.25)	98	4.26 (3.41, 5.10)	84	3.63 (2.85, 4.42)	80	3.50 (2.73, 4.28)	66	2.84 (2.14, 3.53)
10–14 years old	114	3.76 (3.06, 4.45)	122	4.25 (3.49, 5.01)	114	4.17 (3.40, 4.94)	116	4.46 (3.64, 5.28)	119	4.96 (4.06, 5.86)
15–19 years old	92	2.71 (2.15, 3.27)	89	2.64 (2.09, 3.20)	111	3.37 (2.74, 4.00)	121	3.78 (3.10, 4.46)	129	4.13 (3.41, 4.84)
20–44 years old	80	0.40 (031, 0.49)	102	0.52 (0.41, 0.62)	95	0.48 (0.38, 0.58)	122	0.63 (0.52, 0.75)	157	0.82 (0.69, 0.95)
45–64 years old	3	0.01 (0.00, 0.04)	4	0.02 (0.00, 0.05)	3	0.01 (0.00, 0.04)	3	0.01 (0.00, 0.04)	5	0.02 (0.00, 0.05)
Over 65 years	0	0.00 (0.00, 0.00)	1	0.01 (0.00, 0.05)	1	0.01 (0.00, 0.04)	1	0.01 (0.00, 0.04)	1	0.01 (0.00, 0.04)

a Age-standardized prevalence rates of single ventricle were calculated using age groups according to the direct method using the estimated Korean population in 2015 as a reference.

Supplementary Table 2. Age-standardized mortality[a] and 95% confidence interval (CI) of congenital heart disease overall and by sex (per 100,000).

Variables	2007		2008		2009		2010	
	n	Mortality (95% CI)	n	Mortality (95% CI)	n	Mortality (95% CI)	n	Mortality (95% CI)
All	1,509	3.703 (3.512, 3.894)	1,235	3.006 (2.836, 3.177)	1,084	2.530 (2.377, 2.682)	1,083	2.488 (2.338, 2.638)
Adults	833	3.061 (2.850, 3.272)	703	2.504 (2.317, 2.691)	596	1.999 (1.837, 2.161)	610	1.994 (1.834, 2.153)
0–4 years old	605	26.84 (24.70, 28.98)	490	21.92 (19.97, 23.86)	447	19.99 (18.13, 21.85)	447	19.68 (17.85, 21.51)
5–9 years old	25	0.887 (0.539, 1.236)	14	0.488 (0.214, 0.762)	8	0.266 (0.502, 0.480)	5	0.177 (0.000, 0.360)
10–14 years old	29	0.868 (0.548, 1.188)	13	0.372 (0.153, 0.590)	12	0.372 (0.158, 0.585)	2	0.041 (0.000, 0.130)
15–19 years old	17	0.536 (0.278, 0.794)	15	0.441 (0.205, 0.678)	21	0.630 (0.358, 0.903)	19	0.536 (0.282, 0.789)
20–44 years old	144	0.715 (0.598, 0.832)	93	0.460 (0.365, 0.555)	82	0.410 (0.320, 0.500)	63	0.316 (0.236, 0.395)
45–64 years old	213	1.795 (1.554, 2.037)	166	1.350 (1.143, 1.556)	155	1.217 (1.024, 1.409)	149	1.130 (0.949, 1.312)
Over 65 years	476	10.83 (9.863, 11.81)	444	9.651 (8.753, 10.54)	359	7.428 (6.659, 8.198)	398	7.992 (7.206, 8.777)
Women	816	2.034 (1.891, 2.177)	637	1.582 (1.457, 1.707)	508	1.186 (1.082, 1.291)	573	1.318 (1.209, 1.427)
Adults	457	1.715 (1.556, 1.874)	392	1.410 (1.269, 1.550)	279	0.935 (0.824, 1.046)	313	1.030 (0.915, 1.145)
0–4 years old	324	14.35 (12.79, 15.92)	234	10.46 (9.125, 11.81)	217	9.707 (8.413, 11.00)	250	11.00 (9.638, 12.37)
5–9 years old	15	0.532 (0.262, 0.802)	5	0.177 (0.013, 0.341)	1	0.000 (0.000, 0.075)	2	0.044 (0.000, 0.160)
10–14 years old	14	0.413 (0.191, 0.635)	3	0.082 (0.000, 0.187)	5	0.124 (0.000, 0.261)	1	0.000 (0.000, 0.062)
15–19 years old	6	0.189 (0.036, 0.342)	3	0.063 (0.000, 0.168)	6	0.157 (0.012, 0.303)	7	0.189 (0.035, 0.343)
20–44 years old	66	0.327 (0.248, 0.406)	48	0.238 (0.170, 0.306)	44	0.221 (0.156, 0.287)	31	0.155 (0.099, 0.211)
45–64 years old	94	0.791 (0.630, 0.952)	78	0.631 (0.490, 0.773)	57	0.445 (0.329, 0.562)	63	0.472 (0.354, 0.590)
Over 65 years	297	6.758 (5.989, 7.528)	266	5.769 (5.074, 6.464)	178	3.683 (3.142, 4.225)	219	4.384 (3.801, 4.966)
Men	693	1.668 (1.541, 1.795)	598	1.424 (1.308, 1.540)	576	1.343 (1.232, 1.454)	510	1.169 (1.066, 1.272)
Adults	376	1.346 (1.208, 1.484)	311	1.092 (0.969, 1.215)	317	1.063 (0.945, 1.181)	297	0.961 (0.851, 1.071)
0–4 years old	281	12.43 (10.97, 13.89)	256	11.45 (10.04, 12.85)	230	10.28 (8.957, 11.62)	197	8.678 (7.465, 9.891)
5–9 years old	10	0.355 (0.134, 0.575)	9	0.310 (0.091, 0.530)	7	0.266 (0.065, 0.466)	3	0.088 (0.000, 0.230)
10–14 years old	15	0.413 (0.183, 0.643)	10	0.289 (0.097, 0.481)	7	0.206 (0.043, 0.369)	1	0.000 (0.000, 0.062)
15–19 years old	11	0.346 (0.139, 0.554)	12	0.346 (0.135, 0.558)	15	0.441 (0.211, 0.671)	12	0.346 (0.145, 0.548)
20–44 years old	78	0.382 (0.296, 0.468)	45	0.221 (0.156, 0.287)	38	0.188 (0.127, 0.249)	32	0.160 (0.104, 0.217)
45–64 years old	119	1.004 (0.823, 1.185)	88	0.718 (0.568, 0.868)	98	0.771 (0.618, 0.924)	86	0.651 (0.513, 0.789)
Over 65 years	179	4.079 (3.482, 4.677)	178	3.866 (3.298, 4.435)	181	3.744 (3.198, 4.291)	179	3.592 (3.065, 4.119)

Cont. Suppl. Table 2.

Variables	2011		2012		2013		2014		2015	
	n	Mortality (95% CI)	n	Mortality (95% CI)	n	Mortality (95% CI)	n	Mortality (95% CI)	n	Mortality (95% CI)
All	1,088	2.434 (2.288, 2.580)	940	2.029 (1.899, 2.160)	854	1.772 (1.653, 1.891)	793	1.601 (1.490, 1.713)	593	1.184 (1.089, 1.279)
Adults	619	1.945 (1.791, 2.099)	518	1.534 (1.402, 1.667)	396	1.107 (0.998, 1.217)	340	0.902 (0.805, 0.998)	216	0.551 (0.477, 0.624)
0–4 years old	458	19.90 (18.08, 21.73)	417	18.16 (16.41, 19.90)	450	19.72 (17.90, 21.55)	449	19.68 (17.85, 21.50)	376	16.68 (14.99, 18.37)
5–9 years old	2	0.044 (0.000, 0.165)	1	0.000 (0.000. 0.085)	0	0.000 (0.000, 0.000)	1	0.000 (0.000, 0.086)	0	0.000 (0.000, 0.000)
10–14 years old	6	0.165 (0.005, 0.325)	3	0.082 (0.000, 0.201)	4	0.124 (0.000, 0.268)	1	0.000 (0.000, 0.076)	1	0.041 (0.000, 0.123)
15–19 years old	3	0.063 (0.000, 0.164)	1	0.000 (0.000, 0.058)	4	0.094 (0.000, 0.214)	2	0.031 (0.000, 0.118)	0	0.000 (0.000, 0.000)
20–44 years old	46	0.233 (0.165, 0.301)	37	0.188 (0.127, 0.250)	30	0.155 (0.099, 0.211)	27	0.138 (0.085, 0.192)	13	0.066 (0.029, 0.103)
45–64 years old	137	1.004 (0.835, 1.173)	114	0.811 (0.661, 0.961)	90	0.618 (0.489, 0.747)	71	0.472 (0.361, 0.583)	45	0.292 (0.206, 0.378)
Over 65 years	436	8.403 (7.613, 9.192)	367	6.698 (6.011, 7.384)	276	4.795 (4.227, 5.362)	242	4.018 (3.511, 4.526)	158	2.527 (2.131, 2.922)
Women	525	1.188 (1.085, 1.291)	466	1.012 (0.920, 1.105)	421	0.874 (0.790, 0.958)	391	0.790 (0.712, 0.869)	293	0.586 (0.519, 0.653)
Adults	317	1.002 (0.893, 1.115)	276	0.817 (0.720, 0.914)	196	0.550 (0.472, 0.627)	173	0.459 (0.390, 0.528)	116	0.296 (0.242, 0.350)
0–4 years old	202	8.768 (7.557, 9.978)	187	8.141 (6.973, 9.309)	221	9.662 (8.383, 10.94)	215	9.439 (8.177, 10.70)	176	7.783 (6.627, 8.939)
5–9 years old	1	0.000 (0.000, 0.085)	1	0.000 (0.000, 0.085)	0	0.000 (0.000, 0.000)	1	0.000 (0.000, 0.086)	0	0.000 (0.000, 0.000)
10–14 years old	4	0.124 (0.000, 0.254)	2	0.041 (0.000, 0.138)	2	0.041 (0.000, 0.143)	0	0.000 (0.000, 0.000)	1	0.041 (0.000, 0.123)
15–19 years old	1	0.000 (0.000, 0.058)	0	0.000 (0.000, 0.000)	2	0.031 (0.000, 0.116)	2	0.031 (0.000, 0.118)	0	0.000 (0.000, 0.000)
20–44 years old	21	0.105 (0.059, 0.151)	25	0.127 (0.077, 0.178)	13	0.066 (0.029, 0.103)	13	0.066 (0.029, 0.103)	6	0.027 (0.002, 0.053)
45–64 years old	55	0.399 (0.292, 0.505)	46	0.325 (0.230, 0.420)	41	0.279 (0.192, 0.366)	29	0.192 (0.122, 0.263)	23	0.146 (0.084, 0.207)
Over 65 years	241	4.642 (4.056, 5.229)	205	3.744 (3.231, 4.258)	142	2.466 (2.059, 2.873)	131	2.176 (1.803, 2.550)	87	1.385 (1.091, 1.679)
Men	563	1.243 (1.139, 1.347)	474	1.014 (0.922, 1.106)	433	0.896 (0.811, 0.980)	402	0.808 (0.729, 0.888)	300	0.596 (0.528, 0.663)
Adults	302	0.938 (0.832, 1.045)	242	0.714 (0.624, 0.805)	200	0.557 (0.478, 0.627)	167	0.442 (0.375, 0.509)	100	0.254 (0.204, 0.304)
0–4 years old	256	11.09 (9.731, 12.45)	230	10.02 (8.725, 11.31)	229	10.02 (8.718, 11.32)	234	10.24 (8.927, 11.56)	200	8.857 (7.625, 10.08)
5–9 years old	1	0.000 (0.000, 0.085)	0	0.000 (0.000, 0.000)	0	0.000 (0.000, 0.000)	0	0.000 (0.000, 0.000)	0	0.000 (0.000, 0.000)
10–14 years old	2	0.041 (0.050. 0.133)	1	0.000 (0.000, 0.068)	2	0.041 (0.000, 0.143)	1	0.000 (0.000, 0.076)	0	0.000 (0.000, 0.000)
15–19 years old	2	0.031 (0.000, 0.114)	1	0.000 (0.000, 0.058)	2	0.031 (0.000, 0.116)	0	0.000 (0.000, 0.000)	0	0.000 (0.000, 0.000)
20–44 years old	25	0.127 (0.077, 0.177)	12	0.061 (0.026, 0.096)	17	0.083 (0.041, 0.125)	14	0.072 (0.033, 0.110)	7	0.033 (0.005, 0.060)
45–64 years old	82	0.598 (0.468, 0.729)	68	0.485 (0.369, 0.601)	49	0.339 (0.244, 0.434)	42	0.279 (0.194, 0.364)	22	0.139 (0.079, 0.199)
Over 65 years	195	3.760 (3.232, 4.288)	162	2.953 (2.496, 3.409)	134	2.329 (1.933, 2.724)	111	1.842 (1.498, 2.185)	71	1.126 (0.861, 1.391)

a Age-standardized mortality rates of congenital heart disease (ICD 10 codes: Q20, Q21, Q22, Q23, Q24, Q25, and Q26) were calculated using age groups according to the direct method using the estimated Korean population in 2015 as a reference.

Supplementary Table 2-1. Age-standardized mortality[a] and 95% confidence interval (CI) of congenital ventricular and/or atrial septal defects overall and by sex (per 100,000).

Variables	2007		2008		2009		2010	
	n	Mortality (95% CI)	n	Mortality (95% CI)	n	Mortality (95% CI)	n	Mortality (95% CI)
All	76	0.173 (0.133, 0.213)	62	0.141 (0.105, 0.177)	63	0.137 (0.103, 0.172)	48	0.106 (0.075, 0.136)
Adults	40	0.134 (0.091, 0.178)	33	0.108 (0.070, 0.146)	32	0.101 (0.065, 0.137)	27	0.082 (0.050, 0.113)
0–4 years old	21	0.894 (0.495, 1.293)	19	0.849 (0.467, 1.232)	17	0.760 (0.398, 1.122)	18	0.760 (0.393, 1.127)
5–9 years old	3	0.088 (0.000, 0.209)	4	0.133 (0.000, 0.279)	1	0.000 (0.000, 0.075)	1	0.000 (0.000, 0.081)
10–14 years old	7	0.206 (0.049, 0.363)	2	0.041 (0.000, 0.127)	6	0.165 (0.014, 0.316)	0	0.000 (0.000, 0.000)
15–19 years old	5	0.157 (0.017, 0.297)	4	0.094 (0.000, 0.216)	7	0.189 (0.032, 0.346)	2	0.031 (0.000, 0.113)
20–44 years old	12	0.055 (0.021, 0.089)	9	0.044 (0.014, 0.073)	7	0.033 (0.007, 0.059)	7	0.033 (0.006, 0.059)
45–64 years old	13	0.106 (0.046, 0.166)	10	0.079 (0.029, 0.130)	12	0.093 (0.039, 0.146)	7	0.046 (0.007, 0.085)
Over 65 years	15	0.334 (0.161, 0.507)	14	0.289 (0.129, 0.448)	13	0.258 (0.112, 0.405)	13	0.258 (0.116, 0.400)
Women	39	0.087 (0.059, 0.116)	33	0.076 (0.050, 0.103)	29	0.063 (0.040, 0.087)	24	0.053 (0.031, 0.075)
Adults	18	0.060 (0.030, 0.090)	19	0.062 (0.033, 0.091)	15	0.045 (0.020, 0.070)	14	0.042 (0.018, 0.065)
0–4 years old	11	0.447 (0.158, 0.736)	11	0.492 (0.200, 0.783)	7	0.313 (0.080, 0.545)	9	0.357 (0.098, 0.617)
5–9 years old	2	0.044 (0.000, 0.142)	0	0.000 (0.000, 0.000)	0	0.000 (0.000, 0.000)	1	0.000 (0.000, 0.081)
10–14 years old	5	0.124 (0.000, 0.256)	1	0.000 (0.000, 0.060)	3	0.082 (0.000, 0.189)	0	0.000 (0.000, 0.000)
15–19 years old	3	0.094 (0.000, 0.202)	2	0.031 (0.000, 0.117)	4	0.094 (0.000, 0.213)	0	0.000 (0.000, 0.000)
20–44 years old	4	0.016 (0.000, 0.036)	5	0.022 (0.000, 0.044)	4	0.016 (0.000, 0.036)	3	0.011 (0.000, 0.028)
45–64 years old	7	0.053 (0.009, 0.097)	6	0.046 (0.007, 0.085)	4	0.026 (0.000, 0.057)	3	0.019 (0.000, 0.045)
Over 65 years	7	0.152 (0.034, 0.270)	8	0.167 (0.046, 0.287)	7	0.137 (0.029, 0.244)	8	0.152 (0.040, 0.263)
Men	37	0.083 (0.055, 0.111)	29	0.064, 0.040, 0.088)	34	0.074 (0.048, 0.099)	24	0.051 (0.029, 0.072)
Adults	22	0.070 (0.039, 0.101)	14	0.046 (0.021, 0.070)	17	0.053 (0.027, 0.079)	13	0.037 (0.015, 0.058)
0–4 years old	10	0.402 (0.127, 0.677)	8	0.357 (0.109, 0.606)	10	0.447 (0.169, 0.725)	9	0.357 (0.098, 0.617)
5–9 years old	1	0.000 (0.000, 0.069)	4	0.133 (0.000, 0.279)	1	0.000 (0.000, 0.075)	0	0.000 (0.000, 0.000)
10–14 years old	2	0.041 (0.000, 0.125)	1	0.000 (0.000, 0.060)	3	0.082 (0.000, 0.189)	0	0.000 (0.000, 0.000)
15–19 years old	2	0.063 (0.000, 0.151)	2	0.031 (0.000, 0.117)	3	0.063 (0.000, 0.165)	2	0.031 (0.000, 0.113)
20–44 years old	8	0.038 (0.011, 0.066)	4	0.016 (0.000, 0.036)	3	0.011 (0.000, 0.028)	4	0.016 (0.000, 0.036)
45–64 years old	6	0.046 (0.005, 0.087)	4	0.026 (0.000, 0.058)	8	0.059 (0.016, 0.103)	4	0.026 (0.000, 0.056)
Over 65 years	8	0.167 (0.041, 0.293)	6	0.121 (0.017, 0.226)	6	0.121 (0.022, 0.221)	5	0.091 (0.003, 0.179)

Cont. Suppl. Table 2-1.

Variables	2011		2012		2013		2014		2015	
	n	Mortality (95% CI)	n	Mortality (95% CI)	n	Mortality (95% CI)	n	Mortality (95% CI)	n	Mortality (95% CI)
All	167	0.365 (0.309, 0.420)	142	0.300 (0.250, 0.349)	135	0.278 (0.231, 0.325)	130	0.262 (0.217, 0.307)	113	0.225 (0.184, 0.267)
Adults	104	0.310 (0.249, 0.370)	82	0.233 (0.182, 0.285)	76	0.205 (0.158, 0.251)	71	0.184 (0.141, 0.228)	55	0.138 (0.101, 0.175)
0–4 years old	56	2.415 (1.778, 3.053)	57	2.460 (1.815, 3.105)	57	2.460 (1.810, 3.109)	56	2.415 (1.771, 3.059)	57	2.505 (1.847, 3.162)
5–9 years old	0	0.000 (0.000, 0.000)	1	0.000 (0.000, 0.085)	0	0.000 (0.000, 0.000)	0	0.000 (0.000, 0.000)	0	0.000 (0.000, 0.000)
10–14 years old	5	0.165 (0.019, 0.311)	1	0.000 (0.000, 0.068)	0	0.000 (0.000, 0.000)	1	0.000 (0.000, 0.075)	1	0.041 (0.000, 0.123)
15–19 years old	2	0.031 (0.000, 0.113)	1	0.000 (0.000, 0.058)	2	0.031 (0.000, 0.116)	2	0.031 (0.000, 0.118)	0	0.000 (0.000, 0.000)
20–44 years old	19	0.094 (0.050, 0.138)	12	0.061 (0.026, 0.095)	16	0.077 (0.036, 0.118)	11	0.055 (0.021, 0.089)	6	0.027 (0.002, 0.053)
45–64 years old	34	0.246 (0.162, 0.330)	30	0.212 (0.136, 0.289)	24	0.159 (0.093, 0.226)	19	0.126 (0.069, 0.183)	14	0.086 (0.038, 0.134)
Over 65 years	51	0.974 (0.704, 1.244)	40	0.730 (0.503, 0.957)	36	0.062 (0.419, 0.829)	41	0.669 (0.460, 0.878)	35	0.548 (0.361, 0.734)
Women	85	0.186 (0.146, 0.226)	70	0.146 (0.111, 0.182)	69	0.140 (0.106, 0.173)	70	0.141 (0.107, 0.174)	61	0.120 (0.090, 0.151)
Adults	54	0.161 (0.117, 0.205)	41	0.115 (0.079, 0.151)	38	0.101 (0.068, 0.134)	41	0.107 (0.074, 0.140)	30	0.074 (0.046, 0.101)
0–4 years old	27	1.163 (0.720, 1.605)	28	1.207 (0.755, 1.659)	30	1.297 (0.826, 1.768)	27	1.163 (0.715, 1.610)	30	1.297 (0.820, 1.774)
5–9 years old	0	0.000 (0.000, 0.000)	1	0.000 (0.000, 0.085)	0	0.000 (0.000, 0.000)	0	0.000 (0.000, 0.000)	0	0.000 (0.000, 0.000)
10–14 years old	3	0.082 (0.000, 0.195)	0	0.000 (0.000, 0.000)	0	0.000 (0.000, 0.000)	0	0.000 (0.000, 0.000)	1	0.041 (0.000, 0.123)
15–19 years old	1	0.000 (0.000, 0.058)	0	0.000 (0.000, 0.000)	1	0.000 (0.000, 0.059)	2	0.031 (0.000, 0.118)	0	0.000 (0.000, 0.000)
20–44 years old	8	0.038 (0.010, 0.067)	7	0.033 (0.006, 0.059)	8	0.038 (0.010, 0.067)	7	0.033 (0.006, 0.060)	3	0.011 (0.000, 0.029)
45–64 years old	18	0.126 (0.065, 0.187)	13	0.086 (0.035, 0.136)	12	0.079 (0.032, 0.126)	9	0.059 (0.020, 0.099)	9	0.053 (0.014, 0.091)
Over 65 years	28	0.532 (0.332, 0.732)	21	0.380 (0.216, 0.544)	18	0.304 (0.159, 0.449)	25	0.411 (0.247, 0.574)	18	0.289 (0.155, 0.422)
Men	82	0.176 (0.137, 0.215)	72	0.151 (0.115, 0.186)	66	0.136 (0.103, 0.168)	60	0.119 (0.088, 0.149)	52	0.102 (0.074, 0.131)
Adults	50	0.146 (0.104, 0.187)	41	0.115 (0.079, 0.151)	38	0.101 (0.068, 0.134)	30	0.077 (0.049, 0.105)	25	0.061 (0.036, 0.086)
0–4 years old	29	1.252 (0.793, 1.711)	29	1.252 (0.792, 1.712)	27	1.163 (0.716, 1.610)	29	1.252 (0.789, 1.716)	27	1.163 (0.710, 1.615)
5–9 years old	0	0.000 (0.000, 0.000)	0	0.000 (0.000, 0.000)	0	0.000 (0.000, 0.000)	0	0.000 (0.000, 0.000)	0	0.000 (0.000, 0.000)
10–14 years old	2	0.041 (0.000, 0.133)	1	0.000 (0.000, 0.068)	0	0.000 (0.000, 0.000)	1	0.000 (0.000, 0.075)	0	0.000 (0.000, 0.000)
15–19 years old	1	0.000 (0.000, 0.058)	1	0.000 (0.000, 0.058)	1	0.000 (0.000, 0.059)	0	0.000 (0.000, 0.000)	0	0.000 (0.000, 0.000)
20–44 years old	11	0.055 (0.022, 0.088)	5	0.022 (0.000, 0.044)	8	0.038 (0.010, 0.067)	4	0.016 (0.000, 0.037)	3	0.011 (0.000, 0.029)
45–64 years old	16	0.113 (0.055, 0.170)	17	0.119 (0.061, 0.177)	12	0.079 (0.032, 0.126)	10	0.066 (0.024, 0.108)	5	0.026 (0.000, 0.055)
Over 65 years	23	0.441 (0.260, 0.622)	19	0.334 (0.178, 0.491)	18	0.304 (0.159, 0.449)	16	0.258 (0.128, 0.389)	17	0.258 (0.128, 0.388)

a Age-standardized mortality rates of congenital ventricular and/or atrial septal defects were calculated using age groups according to the direct method using the estimated Korean population in 2015 as a reference.

Supplementary Table 2-1-1. Age-standardized mortality[a] and 95% confidence interval (CI) of congenital ventricular septal defects overall and by sex (per 100,000).

Variables	2011		2012		2013		2014		2015	
	n	Mortality (95% CI)	n	Mortality (95% CI)	n	Mortality (95% CI)	n	Mortality (95% CI)	n	Mortality (95% CI)
All	57	0.121 (0.089, 0.153)	46	0.094 (0.066, 0.122)	52	0.104 (0.075, 0.132)	45	0.089 (0.063, 0.115)	43	0.085 (0.059, 0.110)
Adults	34	0.099 (0.065, 0.133)	23	0.061 (0.035, 0.088)	23	0.060 (0.035, 0.086)	19	0.047 (0.025, 0.069)	18	0.044 (0.023, 0.065)
0–4 years old	21	0.894 (0.504, 1.285)	21	0.894 (0.503, 1.286)	28	1.207 (0.752, 1.663)	24	1.028 (0.607, 1.450)	24	1.028 (0.620, 1.455)
5–9 years old	0	0.000 (0.000, 0.000)	1	0.000 (0.000, 0.085)	0	0.000 (0.000, 0.000)	0	0.000 (0.000, 0.000)	0	0.000 (0.000, 0.000)
10–14 years old	2	0.041 (0.000, 0.133)	0	0.000 (0.000, 0.000)	0	0.000 (0.000, 0.000)	1	0.000 (0.000, 0.075)	1	0.041 (0.000, 0.123)
15–19 years old	0	0.000 (0.000, 0.000)	1	0.000 (0.000, 0.058)	1	0.000 (0.000, 0.059)	1	0.000 (0.000, 0.061)	0	0.000 (0.000, 0.000)
20–44 years old	10	0.049 (0.182, 0.081)	6	0.027 (0.003, 0.052)	7	0.033 (0.006, 0.606)	4	0.016 (0.000, 0.037)	2	0.005 (0.000, 0.020)
45–64 years old	8	0.053 (0.012, 0.093)	8	0.053 (0.013, 0.092)	6	0.039 (0.006, 0.073)	3	0.019 (0.000, 0.042)	5	0.026 (0.000, 0.055)
Over 65 years	16	0.304 (0.153, 0.455)	9	0.152 (0.044, 0.259)	10	0.167 (0.059, 0.275)	12	0.197 (0.084, 0.310)	11	0.167 (0.062, 0.271)
Women	29	0.062 (0.039, 0.086)	23	0.048 (0.028, 0.068)	26	0.052 (0.031, 0.072)	22	0.043 (0.025, 0.062)	25	0.049 (0.030, 0.069)
Adults	18	0.052 (0.026, 0.077)	13	0.035 (0.015, 0.056)	13	0.032 (0.014, 0.051)	10	0.024 (0.008, 0.041)	11	0.027 (0.010, 0.043)
0–4 years old	9	0.357 (0.102, 0.613)	9	0.357 (0.101, 0.614)	13	0.536 (0.226, 0.847)	11	0.447 (0.161, 0.732)	13	0.536 (0.222, 0.850)
5–9 years old	0	0.000 (0.000, 0.000)	1	0.000 (0.000, 0.085)	0	0.000 (0.000, 0.000)	0	0.000 (0.000, 0.000)	0	0.000 (0.000, 0.000)
10–14 years old	2	0.041 (0.000, 0.133)	0	0.000 (0.000, 0.000)	0	0.000 (0.000, 0.000)	0	0.000 (0.000, 0.000)	1	0.041 (0.000, 0.123)
15–19 years old	0	0.000 (0.000, 0.000)	0	0.000 (0.000, 0.000)	0	0.000 (0.000, 0.000)	1	0.000 (0.000, 0.061)	0	0.000 (0.000, 0.000)
20–44 years old	4	0.016 (0.000, 0.036)	3	0.011 (0.000, 0.028)	4	0.016 (0.000, 0.036)	2	0.005 (0.000, 0.020)	2	0.005 (0.000, 0.020)
45–64 years old	3	0.019 (0.000, 0.044)	4	0.026 (0.000, 0.054)	4	0.026 (0.000, 0.053)	1	0.006 (0.000, 0.019)	3	0.013 (0.000, 0.035)
Over 65 years	11	0.197 (0.072, 0.323)	6	0.106 (0.018, 0.194)	5	0.076 (0.000, 0.152)	7	0.106 (0.020, 0.192)	6	0.091 (0.014, 0.168)
Men	28	0.058 (0.036, 0.080)	23	0.046 (0.027, 0.065)	26	0.052 (0.031, 0.072)	23	0.045 (0.026, 0.064)	18	0.035 (0.019, 0.052)
Adults	16	0.044 (0.021, 0.066)	10	0.025 (0.008, 0.042)	10	0.025 (0.008, 0.042)	9	0.022 (0.007, 0.037)	7	0.017 (0.004, 0.030)
0–4 years old	12	0.492 (0.196, 0.787)	12	0.492 (0.196, 0.787)	15	0.626 (0.293, 0.959)	13	0.536 (0.226, 0.847)	11	0.447 (0.158, 0.736)
5–9 years old	0	0.000 (0.000, 0.000)	0	0.000 (0.000, 0.000)	0	0.000 (0.000, 0.000)	0	0.000 (0.000, 0.000)	0	0.000 (0.000, 0.000)
10–14 years old	0	0.000 (0.000, 0.000)	0	0.000 (0.000, 0.000)	0	0.000 (0.000, 0.000)	1	0.000 (0.000, 0.075)	0	0.000 (0.000, 0.000)
15–19 years old	0	0.000 (0.000, 0.000)	1	0.000 (0.000, 0.058)	1	0.000 (0.000, 0.059)	0	0.000 (0.000, 0.000)	0	0.000 (0.000, 0.000)
20–44 years old	6	0.027 (0.003, 0.052)	3	0.011 (0.000, 0.028)	3	0.011 (0.000, 0.028)	2	0.005 (0.000, 0.020)	0	0.000 (0.000, 0.000)
45–64 years old	5	0.033 (0.001, 0.065)	4	0.026 (0.000, 0.054)	2	0.013 (0.000, 0.032)	2	0.013 (0.000, 0.031)	2	0.006 (0.000, 0.024)
Over 65 years	5	0.091 (0.006, 0.175)	3	0.045 (0.000, 0.107)	5	0.076 (0.000, 0.152)	5	0.076 (0.003, 0.149)	5	0.076 (0.005, 0.146)

a Age-standardized mortality rates of congenital ventricular septal defect were calculated using age groups according to the direct method using the estimated Korean population in 2015 as a reference.

Supplementary Table 2-1-2. Age-standardized mortality[a] and 95% confidence interval (CI) of congenital atrial septal defects overall and by sex (per 100,000).

Variables	2011		2012		2013		2014		2015	
	n	Mortality (95% CI)	n	Mortality (95% CI)	n	Mortality (95% CI)	n	Mortality (95% CI)	n	Mortality (95% CI)
All	107	0.235 (0.190, 0.280)	92	0.197 (0.156, 0.237)	81	0.168 (0.131, 0.204)	83	0.166 (0.130, 0.203)	67	0.132 (0.100, 0.164)
Adults	68	0.203 (0.154, 0.252)	57	0.164 (0.121, 0.207)	52	0.139 (0.100, 0.178)	50	0.129 (0.093, 0.166)	36	0.088 (0.059, 0.118)
0–4 years old	34	1.476 (0.979, 1.973)	35	1.520 (1.015, 2.026)	28	1.207 (0.752, 1.663)	32	1.386 (0.899, 1.873)	31	1.342 (0.856, 1.827)
5–9 years old	0	0.000 (0.000, 0.000)	0	0.000 (0.000, 0.000)	0	0.000 (0.000, 0.000)	0	0.000 (0.000, 0.000)	0	0.000 (0.000, 0.000)
10–14 years old	3	0.082 (0.000, 0.195)	0	0.000 (0.000, 0.000)	0	0.000 (0.000, 0.000)	0	0.000 (0.000, 0.000)	0	0.000 (0.000, 0.000)
15–19 years old	2	0.031 (0.000, 0.113)	0	0.000 (0.000, 0.000)	1	0.000 (0.000, 0.059)	1	0.000 (0.000, 0.061)	0	0.000 (0.000, 0.000)
20–44 years old	9	0.044 (0.014, 0.074)	6	0.027 (0.003, 0.052)	9	0.044 (0.013, 0.074)	7	0.033 (0.006, 0.060)	4	0.016 (0.000, 0.037)
45–64 years old	26	0.186 (0.112, 0.259)	22	0.152 (0.087, 0.218)	17	0.113 (0.057, 0.169)	16	0.106 (0.053, 0.159)	9	0.053 (0.014, 0.091)
Over 65 years	33	0.624 (0.406, 0.841)	29	0.517 (0.324, 0.710)	26	0.441 (0.267, 0.615)	27	0.441 (0.271, 0.611)	23	0.365 (0.214, 0.516)
Women	55	0.119 (0.087, 0.151)	45	0.094 (0.066, 0.122)	41	0.084 (0.057, 0.110)	46	0.091 (0.064, 0.118)	35	0.069 (0.046, 0.092)
Adults	35	0.104 (0.069, 0.139)	27	0.077 (0.047, 0.106)	24	0.063 (0.037, 0.089)	29	0.074 (0.047, 0.102)	18	0.044 (0.023, 0.065)
0–4 years old	18	0.760 (0.399, 1.121)	18	0.760 (0.398, 1.122)	16	0.671 (0.326, 1.015)	16	0.671 (0.326, 1.015)	17	0.715 (0.356, 1.074)
5–9 years old	0	0.000 (0.000, 0.000)	0	0.000 (0.000, 0.000)	0	0.000 (0.000, 0.000)	0	0.000 (0.000, 0.000)	0	0.000 (0.000, 0.000)
10–14 years old	1	0.000 (0.000, 0.065)	0	0.000 (0.000, 0.000)	0	0.000 (0.000, 0.000)	0	0.000 (0.000, 0.000)	0	0.000 (0.000, 0.000)
15–19 years old	1	0.000 (0.000, 0.058)	0	0.000 (0.000, 0.000)	1	0.000 (0.000, 0.059)	1	0.000 (0.000, 0.061)	0	0.000 (0.000, 0.000)
20–44 years old	4	0.016 (0.000, 0.036)	4	0.016 (0.000, 0.036)	4	0.016 (0.000, 0.036)	5	0.022 (0.000, 0.045)	1	0.000 (0.000, 0.010)
45–64 years old	15	0.106 (0.050, 0.162)	9	0.059 (0.017, 0.101)	7	0.046 (0.010, 0.082)	8	0.053 (0.015, 0.090)	6	0.033 (0.001, 0.064)
Over 65 years	16	0.304 (0.153, 0.455)	14	0.243 (0.109, 0.377)	13	0.021 (0.090, 0.336)	16	0.258 (0.128, 0.389)	11	0.167 (0.062, 0.271)
Men	52	0.113 (0.082, 0.144)	47	0.100 (0.071, 0.129)	40	0.082 (0.056, 0.107)	37	0.073 (0.049, 0.097)	32	0.063 (0.041, 0.085)
Adults	33	0.099 (0.064, 0.133)	30	0.084 (0.053, 0.116)	28	0.073 (0.045, 0.101)	21	0.052 (0.028, 0.076)	18	0.044 (0.023, 0.065)
0–4 years old	16	0.671 (0.330, 1.011)	17	0.715 (0.363, 1.067)	12	0.492 (0.194, 0.790)	16	0.671 (0.326, 1.015)	14	0.581 (0.255, 0.907)
5–9 years old	0	0.000 (0.000, 0.000)	0	0.000 (0.000, 0.000)	0	0.000 (0.000, 0.000)	0	0.000 (0.000, 0.000)	0	0.000 (0.000, 0.000)
10–14 years old	2	0.041 (0.000, 0.133)	0	0.000 (0.000, 0.000)	0	0.000 (0.000, 0.000)	0	0.000 (0.000, 0.000)	0	0.000 (0.000, 0.000)
15–19 years old	1	0.000 (0.000, 0.058)	0	0.000 (0.000, 0.000)	0	0.000 (0.000, 0.000)	0	0.000 (0.000, 0.000)	0	0.000 (0.000, 0.000)
20–44 years old	5	0.022 (0.000, 0.044)	2	0.005 (0.000, 0.019)	5	0.022 (0.000, 0.044)	2	0.005 (0.000, 0.020)	3	0.011 (0.000, 0.029)
45–64 years old	11	0.079 (0.032, 0.127)	13	0.086 (0.035, 0.136)	10	0.066 (0.235, 0.109)	8	0.053 (0.015, 0.090)	3	0.013 (0.000, 0.035)
Over 65 years	17	0.319 (0.163, 0.475)	15	0.274 (0.135, 0.412)	13	0.213 (0.090, 0.336)	11	0.182 (0.074, 0.290)	12	0.182 (0.073, 0.291)

a Age-standardized mortality rates of congenital atrial septal defect were calculated using age groups according to the direct method using the estimated Korean population in 2015 as a reference.

Supplementary Table 2-2. Age-standardized mortality[a] and 95% confidence interval (CI) of patent ductus arteries overall and by sex (per 100,000).

Variables	2007		2008		2009		2010	
	n	Mortality (95% CI)	n	Mortality (95% CI)	n	Mortality (95% CI)	n	Mortality (95% CI)
All	30	0.066 (0.042, 0.091)	23	0.049 (0.029, 0.070)	23	0.049 (0.028, 0.070)	21	0.042 (0.023, 0.062)
Adults	15	0.049 (0.023, 0.075)	11	0.032 (0.012, 0.053)	11	0.032 (0.012, 0.052)	8	0.023 (0.006, 0.040)
0–4 years old	10	0.402 (0.127, 0.677)	10	0.447 (0.169, 0.724)	10	0.447 (0.169, 0.725)	10	0.402 (0.129, 0.675)
5–9 years old	2	0.044 (0.000, 0.142)	1	0.000 (0.000, 0.073)	0	0.000 (0.000, 0.000)	0	0.000 (0.000, 0.000)
10–14 years old	3	0.082 (0.000, 0.185)	1	0.000 (0.000, 0.060)	0	0.000 (0.000, 0.000)	1	0.000 (0.000, 0.062)
15–19 years old	0	0.000 (0.000, 0.000)	0	0.000 (0.000, 0.000)	2	0.031 (0.000, 0.115)	2	0.031 (0.000, 0.113)
20–44 years old	5	0.022 (0.000, 0.044)	5	0.022 (0.000, 0.044)	4	0.016 (0.000, 0.036)	2	0.005 (0.000, 0.019)
45–64 years old	5	0.039 (0.002, 0.076)	3	0.019 (0.000, 0.047)	4	0.026 (0.000, 0.057)	3	0.019 (0.000, 0.045)
Over 65 years	5	0.106 (0.006, 0.206)	3	0.060 (0.000, 0.134)	3	0.060 (0.000, 0.131)	3	0.045 (0.000, 0.113)
Women	18	0.039 (0.020, 0.059)	10	0.020 (0.007, 0.034)	12	0.026 (0.011, 0.042)	12	0.022 (0.008, 0.036)
Adults	10	0.033 (0.011, 0.054)	4	0.010 (0.000, 0.022)	7	0.021 (0.005, 0.037)	4	0.010 (0.000, 0.021)
0–4 years old	5	0.178 (0.000, 0.373)	5	0.223 (0.027, 0.419)	5	0.223 (0.027, 0.420)	5	0.178 (0.000, 0.372)
5–9 years old	1	0.000 (0.000, 0.069)	1	0.000 (0.000, 0.073)	0	0.000 (0.000, 0.000)	0	0.000 (0.000, 0.000)
10–14 years old	2	0.041 (0.000, 0.125)	0	0.000 (0.000, 0.000)	0	0.000 (0.000, 0.000)	1	0.000 (0.000, 0.062)
15–19 years old	0	0.000 (0.000, 0.000)	0	0.000 (0.000, 0.000)	0	0.000 (0.000, 0.000)	2	0.031 (0.000, 0.113)
20–44 years old	3	0.011 (0.000, 0.027)	2	0.005 (0.000, 0.019)	2	0.005 (0.000, 0.019)	2	0.005 (0.000, 0.019)
45–64 years old	3	0.019 (0.000, 0.048)	1	0.006 (0.000, 0.022)	3	0.019 (0.000, 0.046)	1	0.006 (0.000, 0.021)
Over 65 years	4	0.076 (0.000, 0.165)	1	0.015 (0.000, 0.057)	2	0.030 (0.000, 0.087)	1	0.015 (0.000, 0.054)
Men	12	0.025 (0.010, 0.039)	13	0.026 (0.011, 0.042)	11	0.022 (0.008, 0.036)	9	0.018 (0.005, 0.031)
Adults	5	0.013 (0.000, 0.028)	7	0.021 (0.005, 0.038)	4	0.010 (0.000, 0.022)	4	0.010 (0.000, 0.023)
0–4 years old	5	0.178 (0.000, 0.373)	5	0.223 (0.027, 0.419)	5	0.223 (0.027, 0.420)	5	0.178 (0.000, 0.372)
5–9 years old	1	0.000 (0.000, 0.069)	0	0.000 (0.000, 0.000)	0	0.000 (0.000, 0.000)	0	0.000 (0.000, 0.000)
10–14 years old	1	0.000 (0.000, 0.059)	1	0.000 (0.000, 0.060)	0	0.000 (0.000, 0.000)	0	0.000 (0.000, 0.000)
15–19 years old	0	0.000 (0.000, 0.000)	0	0.000 (0.000, 0.000)	2	0.031 (0.000, 0.115)	0	0.000 (0.000, 0.000)
20–44 years old	2	0.005 (0.000, 0.019)	3	0.011 (0.000, 0.028)	2	0.005 (0.000, 0.019)	0	0.000 (0.000, 0.000)
45–64 years old	2	0.013 (0.000, 0.036)	2	0.013 (0.000, 0.035)	1	0.006 (0.000, 0.022)	2	0.013 (0.000, 0.034)
Over 65 years	1	0.015 (0.000, 0.059)	2	0.030 (0.000, 0.090)	1	0.015 (0.000, 0.055)	2	0.030 (0.000, 0.086)

Cont. Suppl. Table 2-2.

Variables	2011		2012		2013		2014		2015	
	n	Mortality (95% CI)	n	Mortality (95% CI)	n	Mortality (95% CI)	n	Mortality (95% CI)	n	Mortality (95% CI)
All	21	0.044 (0.025, 0.064)	19	0.038 (0.020, 0.056)	15	0.030 (0.014, 0.045)	15	0.027 (0.012, 0.042)	12	0.021 (0.008, 0.035)
Adults	9	0.026 (0.007, 0.044)	8	0.020 (0.004, 0.036)	5	0.012 (0.000, 0.024)	5	0.012 (0.001, 0.023)	2	0.002 (0.000, 0.009)
0–4 years old	10	0.402 (0.133, 0.672)	10	0.402 (0.132, 0.672)	10	0.402 (0.130, 0.674)	10	0.402 (0.130, 0.674)	10	0.402 (0.127, 0.678)
5–9 years old	2	0.044 (0.000, 0.165)	0	0.000 (0.000, 0.000)	0	0.000 (0.000, 0.000)	0	0.000 (0.000, 0.000)	0	0.000 (0.000, 0.000)
10–14 years old	0	0.000 (0.000, 0.000)	1	0.000 (0.000, 0.068)	0	0.000 (0.000, 0.000)	0	0.000 (0.000, 0.000)	0	0.000 (0.000, 0.000)
15–19 years old	0	0.000 (0.000, 0.000)	0	0.000 (0.000, 0.000)	0	0.000 (0.000, 0.000)	0	0.000 (0.000, 0.000)	0	0.000 (0.000, 0.000)
20–44 years old	1	0.000 (0.000, 0.010)	2	0.005 (0.000, 0.019)	0	0.000 (0.000, 0.000)	1	0.000 (0.000, 0.010)	1	0.000 (0.000, 0.010)
45–64 years old	3	0.019 (0.000, 0.044)	1	0.006 (0.000, 0.020)	2	0.013 (0.000, 0.032)	2	0.013 (0.000, 0.031)	1	0.000 (0.000, 0.012)
Over 65 years	5	0.091 (0.006, 0.175)	5	0.091 (0.011, 0.171)	3	0.045 (0.000, 0.104)	2	0.030 (0.000, 0.076)	0	0.000 (0.000, 0.000)
Women	14	0.030 (0.014, 0.046)	12	0.024 (0.010, 0.038)	9	0.018 (0.005, 0.030)	8	0.013 (0.002, 0.024)	6	0.009 (0.000, 0.019)
Adults	8	0.023 (0.006, 0.040)	6	0.015 (0.001, 0.002)	4	0.010 (0.000, 0.021)	3	0.007 (0.000, 0.016)	1	0.000 (0.000, 0.004)
0–4 years old	5	0.178 (0.000, 0.369)	5	0.178 (0.000, 0.369)	5	0.178 (0.000, 0.371)	5	0.178 (0.000, 0.371)	5	0.178 (0.000, 0.373)
5–9 years old	1	0.000 (0.000, 0.085)	0	0.000 (0.000, 0.000)	0	0.000 (0.000, 0.000)	0	0.000 (0.000, 0.000)	0	0.000 (0.000, 0.000)
10–14 years old	0	0.000 (0.000, 0.000)	1	0.000 (0.000, 0.068)	0	0.000 (0.000, 0.000)	0	0.000 (0.000, 0.000)	0	0.000 (0.000, 0.000)
15–19 years old	0	0.000 (0.000, 0.000)	0	0.000 (0.000, 0.000)	0	0.000 (0.000, 0.000)	0	0.000 (0.000, 0.000)	0	0.000 (0.000, 0.000)
20–44 years old	1	0.000 (0.000, 0.010)	2	0.005 (0.000, 0.019)	0	0.000 (0.000, 0.000)	1	0.000 (0.000, 0.010)	0	0.000 (0.000, 0.000)
45–64 years old	3	0.019 (0.000, 0.044)	1	0.006 (0.000, 0.020)	1	0.006 (0.000, 0.020)	1	0.006 (0.000, 0.019)	1	0.000 (0.000, 0.012)
Over 65 years	4	0.076 (0.000, 0.151)	3	0.045 (0.000, 0.107)	3	0.045 (0.000, 0.104)	1	0.015 (0.000, 0.047)	0	0.000 (0.000, 0.000)
Men	7	0.014 (0.003, 0.024)	7	0.014 (0.003, 0.024)	6	0.010 (0.000, 0.019)	7	0.013 (0.003, 0.024)	6	0.009 (0.000, 0.019)
Adults	1	0.002 (0.000, 0.009)	2	0.005 (0.000, 0.013)	1	0.002 (0.000, 0.007)	2	0.004 (0.000, 0.012)	1	0.000 (0.000, 0.004)
0–4 years old	5	0.178 (0.000, 0.369)	5	0.178 (0.000, 0.369)	5	0.178 (0.000, 0.371)	5	0.178 (0.000, 0.371)	5	0.178 (0.000, 0.373)
5–9 years old	1	0.000 (0.000, 0.085)	0	0.000 (0.000, 0.000)	0	0.000 (0.000, 0.000)	0	0.000 (0.000, 0.000)	0	0.000 (0.000, 0.000)
10–14 years old	0	0.000 (0.000, 0.000)	0	0.000 (0.000, 0.000)	0	0.000 (0.000, 0.000)	0	0.000 (0.000, 0.000)	0	0.000 (0.000, 0.000)
15–19 years old	0	0.000 (0.000, 0.000)	0	0.000 (0.000, 0.000)	0	0.000 (0.000, 0.000)	0	0.000 (0.000, 0.000)	0	0.000 (0.000, 0.000)
20–44 years old	0	0.000 (0.000, 0.000)	0	0.000 (0.000, 0.000)	0	0.000 (0.000, 0.000)	0	0.000 (0.000, 0.000)	1	0.000 (0.000, 0.010)
45–64 years old	0	0.000 (0.000, 0.000)	0	0.000 (0.000, 0.000)	1	0.006 (0.000, 0.020)	1	0.006 (0.000, 0.019)	0	0.000 (0.000, 0.000)
Over 65 years	1	0.015 (0.000, 0.053)	2	0.030 (0.000, 0.081)	0	0.000 (0.000, 0.000)	1	0.015 (0.000, 0.047)	0	0.000 (0.000, 0.000)

a Age-standardized mortality rates of patent ductus arteries were calculated using age groups according to the direct method using the estimated Korean population in 2015 as a reference.

Supplementary Table 2-3. Age-standardized mortality[a] and 95% confidence interval (CI) of pulmonary artery stenosis overall and by sex (per 100,000).

Variables	2007		2008		2009		2010	
	n	Mortality (95% CI)	n	Mortality (95% CI)	n	Mortality (95% CI)	n	Mortality (95% CI)
All	28	0.060 (0.036, 0.084)	19	0.043 (0.023, 0.064)	14	0.028 (0.012, 0.044)	17	0.036 (0.019, 0.054)
Adults	9	0.033 (0.011, 0.055)	9	0.029 (0.008, 0.050)	5	0.016 (0.001, 0.030)	10	0.029 (0.010, 0.047)
0–4 years old	14	0.581 (0.255, 0.907)	9	0.402 (0.139, 0.665)	8	0.357 (0.109, 0.606)	5	0.178 (0.000, 0.372)
5–9 years old	3	0.088 (0.000, 0.209)	1	0.000 (0.000, 0.073)	1	0.000 (0.000, 0.075)	0	0.000 (0.000, 0.000)
10–14 years old	2	0.041 (0.000, 0.125)	0	0.000 (0.000, 0.000)	0	0.000 (0.000, 0.000)	0	0.000 (0.000, 0.000)
15–19 years old	0	0.000 (0.000, 0.000)	0	0.000 (0.000, 0.000)	0	0.000 (0.000, 0.000)	2	0.031 (0.000, 0.113)
20–44 years old	2	0.005 (0.000, 0.019)	1	0.000 (0.000, 0.009)	1	0.000 (0.000, 0.009)	3	0.011 (0.000, 0.028)
45–64 years old	1	0.006 (0.000, 0.023)	3	0.019 (0.000, 0.047)	2	0.013 (0.000, 0.035)	4	0.026 (0.000, 0.056)
Over 65 years	6	0.121 (0.012, 0.231)	5	0.106 (0.011, 0.201)	2	0.030 (0.000, 0.087)	3	0.045 (0.000, 0.113)
Women	13	0.027 (0.011, 0.043)	9	0.020 (0.006, 0.034)	5	0.010 (0.000, 0.020)	5	0.010 (0.000, 0.020)
Adults	5	0.016 (0.000, 0.032)	5	0.016 (0.001, 0.031)	1	0.002 (0.000, 0.009)	4	0.010 (0.000, 0.022)
0–4 years old	6	0.223 (0.010, 0.436)	3	0.134 (0.000, 0.286)	4	0.178 (0.003, 0.354)	1	0.000 (0.000, 0.086)
5–9 years old	1	0.000 (0.000, 0.069)	1	0.000 (0.000, 0.073)	0	0.000 (0.000, 0.000)	0	0.000 (0.000, 0.000)
10–14 years old	1	0.000 (0.000, 0.059)	0	0.000 (0.000, 0.000)	0	0.000 (0.000, 0.000)	0	0.000 (0.000, 0.000)
15–19 years old	0	0.000 (0.000, 0.000)	0	0.000 (0.000, 0.000)	0	0.000 (0.000, 0.000)	0	0.000 (0.000, 0.000)
20–44 years old	2	0.005 (0.000, 0.019)	1	0.000 (0.000, 0.009)	0	0.000 (0.000, 0.000)	1	0.000 (0.000, 0.009)
45–64 years old	0	0.000 (0.000, 0.000)	2	0.013 (0.000, 0.035)	0	0.000 (0.000, 0.000)	2	0.013 (0.000, 0.034)
Over 65 years	3	0.060 (0.000, 0.138)	2	0.030 (0.000, 0.090)	1	0.015 (0.000, 0.055)	1	0.015 (0.000, 0.054)
Men	15	0.033 (0.016, 0.050)	10	0.022 (0.007, 0.037)	9	0.018 (0.005, 0.031)	12	0.024 (0.009, 0.039)
Adults	4	0.013 (0.000, 0.029)	4	0.013 (0.000, 0.028)	4	0.010 (0.000, 0.023)	6	0.015 (0.001, 0.030)
0–4 years old	8	0.313 (0.066, 0.559)	6	0.268 (0.053, 0.483)	4	0.178 (0.003, 0.354)	4	0.134 (0.000, 0.307)
5–9 years old	2	0.044 (0.000, 0.142)	0	0.000 (0.000, 0.000)	1	0.000 (0.000, 0.075)	0	0.000 (0.000, 0.000)
10–14 years old	1	0.000 (0.000, 0.059)	0	0.000 (0.000, 0.000)	0	0.000 (0.000, 0.000)	0	0.000 (0.000, 0.000)
15–19 years old	0	0.000 (0.000, 0.000)	0	0.000 (0.000, 0.000)	0	0.000 (0.000, 0.000)	2	0.003 (0.000, 0.113)
20–44 years old	0	0.000 (0.000, 0.000)	0	0.000 (0.000, 0.000)	1	0.000 (0.000, 0.009)	2	0.005 (0.000, 0.019)
45–64 years old	1	0.006 (0.000, 0.023)	1	0.006 (0.000, 0.022)	2	0.013 (0.000, 0.035)	2	0.013 (0.000, 0.034)
Over 65 years	3	0.060 (0.000, 0.138)	3	0.060 (0.000, 0.134)	1	0.015 (0.000, 0.055)	2	0.030 (0.000, 0.086)

Cont. Suppl. Table 2-3.

Variables	2011		2012		2013		2014		2015	
	n	Mortality (95% CI)	n	Mortality (95% CI)	n	Mortality (95% CI)	n	Mortality (95% CI)	n	Mortality (95% CI)
All	12	0.026 (0.011, 0.041)	15	0.032 (0.015, 0.048)	11	0.022 (0.008, 0.035)	4	0.007 (0.000, 0.015)	10	0.019 (0.007, 0.032)
Adults	7	0.020 (0.004, 0.036)	10	0.028 (0.010, 0.046)	4	0.010 (0.000, 0.021)	2	0.004 (0.000, 0.012)	4	0.009 (0.000, 0.019)
0–4 years old	5	0.178 (0.000, 0.369)	5	0.178 (0.000, 0.369)	6	0.223 (0.012, 0.434)	2	0.044 (0.000, 0.166)	6	0.223 (0.010, 0.437)
5–9 years old	0	0.000 (0.000, 0.000)	0	0.000 (0.000, 0.000)	0	0.000 (0.000, 0.000)	0	0.000 (0.000, 0.000)	0	0.000 (0.000, 0.000)
10–14 years old	0	0.000 (0.000, 0.000)	0	0.000 (0.000, 0.000)	1	0.000 (0.000, 0.072)	0	0.000 (0.000, 0.000)	0	0.000 (0.000, 0.000)
15–19 years old	0	0.000 (0.000, 0.000)	0	0.000 (0.000, 0.000)	0	0.000 (0.000, 0.000)	0	0.000 (0.000, 0.000)	0	0.000 (0.000, 0.000)
20–44 years old	1	0.000 (0.000, 0.010)	2	0.005 (0.000, 0.019)	0	0.000 (0.000, 0.000)	0	0.000 (0.000, 0.000)	1	0.000 (0.000, 0.010)
45–64 years old	2	0.013 (0.000, 0.033)	2	0.013 (0.000, 0.033)	1	0.006 (0.000, 0.020)	1	0.006 (0.000, 0.019)	0	0.000 (0.000, 0.000)
Over 65 years	4	0.076 (0.000, 0.151)	6	0.106 (0.018, 0.194)	3	0.045 (0.000, 0.104)	1	0.015 (0.000, 0.047)	3	0.045 (0.000, 0.100)
Women	9	0.018 (0.005, 0.031)	9	0.018 (0.005, 0.030)	6	0.012 (0.002, 0.021)	2	0.003 (0.000, 0.009)	4	0.007 (0.000, 0.015)
Adults	5	0.013 (0.000, 0.026)	6	0.015 (0.001, 0.029)	2	0.005 (0.000, 0.012)	1	0.002 (0.000, 0.007)	2	0.004 (0.000, 0.012)
0–4 years old	4	0.134 (0.000, 0.304)	3	0.089 (0.000, 0.237)	3	0.089 (0.000, 0.238)	1	0.000 (0.000, 0.086)	2	0.044 (0.000, 0.167)
5–9 years old	0	0.000 (0.000, 0.000)	0	0.000 (0.000, 0.000)	0	0.000 (0.000, 0.000)	0	0.000 (0.000, 0.000)	0	0.000 (0.000, 0.000)
10–14 years old	0	0.000 (0.000, 0.000)	0	0.000 (0.000, 0.000)	1	0.000 (0.000, 0.072)	0	0.000 (0.000, 0.000)	0	0.000 (0.000, 0.000)
15–19 years old	0	0.000 (0.000, 0.000)	0	0.000 (0.000, 0.000)	0	0.000 (0.000, 0.000)	0	0.000 (0.000, 0.000)	0	0.000 (0.000, 0.000)
20–44 years old	1	0.000 (0.000, 0.010)	2	0.005 (0.000, 0.019)	0	0.000 (0.000, 0.000)	0	0.000 (0.000, 0.000)	0	0.000 (0.000, 0.000)
45–64 years old	1	0.006 (0.000, 0.021)	0	0.000 (0.000, 0.000)	1	0.006 (0.000, 0.020)	0	0.000 (0.000, 0.000)	0	0.000 (0.000, 0.000)
Over 65 years	3	0.045 (0.000, 0.111)	4	0.060 (0.000, 0.132)	1	0.015 (0.000, 0.049)	1	0.015 (0.000, 0.047)	2	0.030 (0.000, 0.074)
Men	3	0.006 (0.000, 0.013)	6	0.012 (0.001, 0.022)	5	0.010 (0.000, 0.019)	2	0.001 (0.000, 0.007)	6	0.009 (0.000, 0.019)
Adults	2	0.005 (0.000, 0.026)	4	0.010 (0.000, 0.021)	2	0.005 (0.000, 0.013)	1	0.002 (0.000, 0.007)	2	0.004 (0.000, 0.011)
0–4 years old	1	0.000 (0.000, 0.085)	2	0.044 (0.000, 0.165)	3	0.089 (0.000, 0.238)	1	0.000 (0.000, 0.086)	4	0.134 (0.000, 0.308)
5–9 years old	0	0.000 (0.000, 0.000)	0	0.000 (0.000, 0.000)	0	0.000 (0.000, 0.000)	0	0.000 (0.000, 0.000)	0	0.000 (0.000, 0.000)
10–14 years old	0	0.000 (0.000, 0.000)	0	0.000 (0.000, 0.000)	0	0.000 (0.000, 0.000)	0	0.000 (0.000, 0.000)	0	0.000 (0.000, 0.000)
15–19 years old	0	0.000 (0.000, 0.000)	0	0.000 (0.000, 0.000)	0	0.000 (0.000, 0.000)	0	0.000 (0.000, 0.000)	0	0.000 (0.000, 0.000)
20–44 years old	0	0.000 (0.000, 0.000)	0	0.000 (0.000, 0.000)	0	0.000 (0.000, 0.000)	0	0.000 (0.000, 0.000)	1	0.000 (0.000, 0.010)
45–64 years old	1	0.006 (0.000, 0.021)	2	0.013 (0.000, 0.033)	0	0.000 (0.000, 0.000)	1	0.006 (0.000, 0.019)	0	0.000 (0.000, 0.000)
Over 65 years	1	0.015 (0.000, 0.053)	2	0.030 (0.000, 0.081)	2	0.030 (0.000, 0.078)	0	0.000 (0.000, 0.000)	1	0.015 (0.000, 0.046)

a Age-standardized mortality rates of pulmonary artery stenosis were calculated using age groups according to the direct method using the estimated Korean population in 2015 as a reference.

Supplementary Table 2-4. Age-standardized mortality[a] and 95% confidence interval (CI) of Coarctation of aorta overall and by sex (per 100,000).

Variables	2007		2008		2009		2010	
	n	Mortality (95% CI)	n	Mortality (95% CI)	n	Mortality (95% CI)	n	Mortality (95% CI)
All	16	0.037 (0.018, 0.056)	13	0.026 (0.011, 0.042)	10	0.020 (0.006, 0.034)	14	0.028 (0.013, 0.043)
Adults	7	0.024 (0.005, 0.044)	5	0.016 (0.001, 0.031)	5	0.016 (0.001, 0.030)	4	0.010 (0.000, 0.022)
0–4 years old	8	0.313 (0.066, 0.559)	6	0.268 (0.053, 0.483)	3	0.134 (0.000, 0.286)	7	0.268 (0.039, 0.497)
5–9 years old	0	0.000 (0.000, 0.000)	1	0.000 (0.000, 0.073)	2	0.044 (0.000,0.151)	1	0.000 (0.000, 0.081)
10–14 years old	1	0.000 (0.000, 0.059)	1	0.000 (0.000, 0.060)	0	0.000 (0.000, 0.000)	0	0.000 (0.000, 0.000)
15–19 years old	0	0.000 (0.000, 0.000)	0	0.000 (0.000, 0.000)	0	0.000 (0.000, 0.000)	2	0.031 (0.000, 0.113)
20–44 years old	0	0.000 (0.000, 0.000)	2	0.005 (0.000, 0.019)	1	0.000 (0.000, 0.009)	1	0.000 (0.000, 0.009)
45–64 years old	4	0.033 (0.000, 0.066)	0	0.000 (0.000, 0.000)	2	0.013 (0.000, 0.035)	2	0.013 (0.000, 0.034)
Over 65 years	3	0.060 (0.000, 0.138)	3	0.060 (0.000, 0.134)	2	0.030 (0.000, 0.087)	1	0.015 (0.000, 0.054)
Women	10	0.023 (0.007, 0.038)	6	0.012 (0.001, 0.022)	4	0.008 (0.000, 0.018)	8	0.016 (0.004, 0.028)
Adults	5	0.019 (0.002, 0.036)	3	0.008 (0.000, 0.018)	3	0.008 (0.000, 0.019)	2	0.005 (0.000, 0.014)
0–4 years old	4	0.134 (0.000, 0.308)	3	0.134 (0.000, 0.286)	1	0.044 (0.043, 0.132)	4	0.134 (0.000, 0.307)
5–9 years old	0	0.000 (0.000, 0.000)	0	0.000 (0.000, 0.000)	0	0.000 (0.000, 0.000)	1	0.000 (0.000, 0.081)
10–14 years old	1	0.000 (0.000, 0.059)	0	0.000 (0.000, 0.000)	0	0.000 (0.000, 0.000)	0	0.000 (0.000, 0.000)
15–19 years old	0	0.000 (0.000, 0.000)	0	0.000 (0.000, 0.000)	0	0.000 (0.000, 0.000)	1	0.000 (0.000, 0.058)
20–44 years old	0	0.000 (0.000, 0.000)	2	0.005 (0.000, 0.019)	0	0.000 (0.000, 0.000)	0	0.000 (0.000, 0.000)
45–64 years old	2	0.013 (0.000, 0.036)	0	0.000 (0.000, 0.000)	1	0.006 (0.000, 0.022)	1	0.006 (0.000, 0.021)
Over 65 years	3	0.060 (0.000, 0138)	1	0.015 (0.000, 0.057)	2	0.030 (0.000, 0.087)	1	0.015 (0.000, 0.054)
Men	6	0.012 (0.001, 0.023)	7	0.014 (0.002, 0.026)	6	0.010 (0.000, 0.019)	6	0.010 (0.000, 0.019)
Adults	2	0.005 (0.000, 0.015)	2	0.005 (0.000, 0.016)	2	0.005 (0.000, 0.013)	2	0.005 (0.000, 0.012)
0–4 years old	4	0.134 (0.000, 0.308)	3	0.134 (0.000, 0.286)	2	0.089 (0.000, 0.213)	3	0.089 (0.000, 0.239)
5–9 years old	0	0.000 (0.000, 0.000)	1	0.000 (0.000, 0.073)	2	0.044 (0.000, 0.151)	0	0.000 (0.000, 0.000)
10–14 years old	0	0.000 (0.000, 0.000)	1	0.000 (0.000, 0.060)	0	0.000 (0.000, 0.000)	0	0.000 (0.000, 0.000)
15–19 years old	0	0.000 (0.000, 0.000)	0	0.000 (0.000, 0.000)	0	0.000 (0.000, 0.000)	1	0.000 (0.000, 0.058)
20–44 years old	0	0.000 (0.000, 0.000)	0	0.000 (0.000, 0.000)	1	0.000 (0.000, 0.009)	1	0.000 (0.000, 0.009)
45–64 years old	2	0.013 (0.000, 0.036)	0	0.000 (0.000, 0.000)	1	0.006 (0.000, 0.022)	1	0.006 (0.000, 0.021)
Over 65 years	0	0.000 (0.000, 0.000)	2	0.030 (0.000, 0.090)	0	0.000 (0.000, 0.000)	0	0.000 (0.000, 0.000)

Cont. Suppl. Table 2-4.

Variables	2011		2012		2013		2014		2015	
	n	Mortality (95% CI)	n	Mortality (95% CI)	n	Mortality (95% CI)	n	Mortality (95% CI)	n	Mortality (95% CI)
All	7	0.012 (0.002, 0.022)	13	0.026 (0.011, 0.041)	8	0.016 (0.004, 0.027)	9	0.015 (0.004, 0.027)	9	0.017 (0.006, 0.029)
Adults	1	0.000 (0.000, 0.004)	5	0.012 (0.000, 0.026)	2	0.005 (0.000, 0.013)	1	0.000 (0.000, 0.013)	1	0.002 (0.000, 0.007)
0–4 years old	6	0.223 (0.014, 0.432)	8	0.313 (0.071, 0.554)	6	0.223 (0.012, 0.434)	8	0.313 (0.069, 0.556)	8	0.313 (0.066, 0.559)
5–9 years old	0	0.000 (0.000, 0.000)	0	0.000 (0.000, 0.000)	0	0.000 (0.000, 0.000)	0	0.000 (0.000, 0.000)	0	0.000 (0.000, 0.000)
10–14 years old	0	0.000 (0.000, 0.000)	0	0.000 (0.000, 0.000)	0	0.000 (0.000, 0.000)	0	0.000 (0.000, 0.000)	0	0.000 (0.000, 0.000)
15–19 years old	0	0.000 (0.000, 0.000)	0	0.000 (0.000, 0.000)	0	0.000 (0.000, 0.000)	0	0.000 (0.000, 0.000)	0	0.000 (0.000, 0.000)
20–44 years old	1	0.000 (0.000, 0.010)	0	0.000 (0.000, 0.000)	0	0.000 (0.000, 0.000)	1	0.000 (0.000, 0.010)	0	0.000 (0.000, 0.000)
45–64 years old	0	0.000 (0.000, 0.000)	1	0.006 (0.000, 0.020)	0	0.000 (0.000, 0.000)	0	0.000 (0.000, 0.000)	0	0.000 (0.000, 0.000)
Over 65 years	0	0.000 (0.000, 0.000)	4	0.060 (0.000, 0.132)	2	0.030 (0.000, 0.078)	0	0.000 (0.000, 0.000)	1	0.015 (0.000, 0.046)
Women	4	0.006 (0.000, 0.013)	5	0.010 (0.000, 0.019)	3	0.004 (0.000, 0.010)	4	0.005 (0.000, 0.013)	5	0.009 (0.001, 0.018)
Adults	0	0.000 (0.000, 0.000)	2	0.005 (0.000, 0.013)	0	0.000 (0.000, 0.000)	0	0.000 (0.000, 0.000)	1	0.002 (0.000, 0.007)
0–4 years old	4	0.134 (0.000, 0.304)	3	0.089 (0.000, 0.237)	3	0.089 (0.000, 0.238)	4	0.134 (0.000, 0.306)	4	0.134 (0.000, 0.308)
5–9 years old	0	0.000 (0.000, 0.000)	0	0.000 (0.000, 0.000)	0	0.000 (0.000, 0.000)	0	0.000 (0.000, 0.000)	0	0.000 (0.000, 0.000)
10–14 years old	0	0.000 (0.000, 0.000)	0	0.000 (0.000, 0.000)	0	0.000 (0.000, 0.000)	0	0.000 (0.000, 0.000)	0	0.000 (0.000, 0.000)
15–19 years old	0	0.000 (0.000, 0.000)	0	0.000 (0.000, 0.000)	0	0.000 (0.000, 0.000)	0	0.000 (0.000, 0.000)	0	0.000 (0.000, 0.000)
20–44 years old	0	0.000 (0.000, 0.000)	0	0.000 (0.000, 0.000)	0	0.000 (0.000, 0.000)	0	0.000 (0.000, 0.000)	0	0.000 (0.000, 0.000)
45–64 years old	0	0.000 (0.000, 0.000)	0	0.000 (0.000, 0.000)	0	0.000 (0.000, 0.000)	0	0.000 (0.000, 0.000)	0	0.000 (0.000, 0.000)
Over 65 years	0	0.000 (0.000, 0.000)	2	0.030 (0.000, 0.081)	0	0.000 (0.000, 0.000)	0	0.000 (0.000, 0.000)	1	0.015 (0.000, 0.046)
Men	3	0.004 (0.000, 0.010)	8	0.016 (0.004, 0.027)	5	0.010 (0.000, 0.019)	5	0.007 (0.000, 0.016)	4	0.005 (0.000, 0.013)
Adults	1	0.000 (0.000, 0.026)	3	0.007 (0.000, 0.017)	2	0.005 (0.000, 0.013)	1	0.000 (0.000, 0.004)	0	0.000 (0.000, 0.000)
0–4 years old	2	0.044 (0.000, 0.165)	5	0.178 (0.000, 0.369)	3	0.089 (0.000, 0.238)	4	0.313 (0.066, 0.559)	4	0.134 (0.000, 0.308)
5–9 years old	0	0.000 (0.000, 0.000)	0	0.000 (0.000, 0.000)	0	0.000 (0.000, 0.000)	0	0.000 (0.000, 0.000)	0	0.000 (0.000, 0.000)
10–14 years old	0	0.000 (0.000, 0.000)	0	0.000 (0.000, 0.000)	0	0.000 (0.000, 0.000)	0	0.000 (0.000, 0.000)	0	0.000 (0.000, 0.000)
15–19 years old	0	0.000 (0.000, 0.000)	0	0.000 (0.000, 0.000)	0	0.000 (0.000, 0.000)	0	0.000 (0.000, 0.000)	0	0.000 (0.000, 0.000)
20–44 years old	1	0.000 (0.000, 0.010)	0	0.000 (0.000, 0.000)	0	0.000 (0.000, 0.000)	1	0.000 (0.000, 0.010)	0	0.000 (0.000, 0.000)
45–64 years old	0	0.000 (0.000, 0.000)	1	0.006 (0.000, 0.020)	0	0.000 (0.000, 0.000)	0	0.000 (0.000, 0.000)	0	0.000 (0.000, 0.000)
Over 65 years	0	0.000 (0.000, 0.000)	2	0.030 (0.000, 0.081)	2	0.030 (0.000, 0.078)	0	0.000 (0.000, 0.000)	0	0.000 (0.000, 0.000)

a Age-standardized mortality rates of Coarctation of aorta were calculated using age groups according to the direct method using the estimated Korean population in 2015 as a reference.

Supplementary Table 2-5. Age-standardized mortality[a] and 95% confidence interval (CI) of pulmonary venous connection overall and by sex (per 100,000).

Variables	2007		2008		2009		2010	
	n	Mortality (95% CI)	n	Mortality (95% CI)	n	Mortality (95% CI)	n	Mortality (95% CI)
All	6	0.010 (0.000, 0.020)	5	0.008 (0.000, 0.017)	8	0.016 (0.004, 0.028)	5	0.008 (0.000, 0.017)
Adults	0	0.000 (0.000, 0.000)	0	0.000 (0.000, 0.000)	1	0.002 (0.000, 0.008)	0	0.000 (0.000, 0.000)
0–4 years old	6	0.223 (0.010, 0.436)	4	0.178 (0.003, 0.354)	6	0.268 (0.053, 0.483)	5	0.178 (0.000, 0.372)
5–9 years old	0	0.000 (0.000, 0.000)	1	0.000 (0.000, 0.073)	0	0.000 (0.000, 0.000)	0	0.000 (0.000, 0.000)
10–14 years old	0	0.000 (0.000, 0.000)	0	0.000 (0.000, 0.000)	0	0.000 (0.000, 0.000)	0	0.000 (0.000, 0.000)
15–19 years old	0	0.000 (0.000, 0.000)	0	0.000 (0.000, 0.000)	1	0.000 (0.000, 0.059)	0	0.000 (0.000, 0.000)
20–44 years old	0	0.000 (0.000, 0.000)	0	0.000 (0.000, 0.000)	0	0.000 (0.000, 0.000)	0	0.000 (0.000, 0.000)
45–64 years old	0	0.000 (0.000, 0.000)	0	0.000 (0.000, 0.000)	1	0.006 (0.000, 0.022)	0	0.000 (0.000, 0.000)
Over 65 years	0	0.000 (0.000, 0.000)	0	0.000 (0.000, 0.000)	0	0.000 (0.000, 0.000)	0	0.000 (0.000, 0.000)
Women	3	0.004 (0.000, 0.011)	2	0.002 (0.000, 0.007)	3	0.006 (0.000, 0.013)	3	0.004 (0.000, 0.010)
Adults	0	0.000 (0.000, 0.000)	0	0.000 (0.000, 0.000)	1	0.002 (0.000, 0.008)	0	0.000 (0.000, 0.000)
0–4 years old	3	0.089 (0.000, 0.240)	1	0.044 (0.000, 0.132)	2	0.089 (0.000, 0.213)	3	0.089 (0.000, 0.239)
5–9 years old	0	0.000 (0.000, 0.000)	1	0.000 (0.000, 0.073)	0	0.000 (0.000, 0.000)	0	0.000 (0.000, 0.000)
10–14 years old	0	0.000 (0.000, 0.000)	0	0.000 (0.000, 0.000)	0	0.000 (0.000, 0.000)	0	0.000 (0.000, 0.000)
15–19 years old	0	0.000 (0.000, 0.000)	0	0.000 (0.000, 0.000)	0	0.000 (0.000, 0.000)	0	0.000 (0.000, 0.000)
20–44 years old	0	0.000 (0.000, 0.000)	0	0.000 (0.000, 0.000)	0	0.000 (0.000, 0.000)	0	0.000 (0.000, 0.000)
45–64 years old	0	0.000 (0.000, 0.000)	0	0.000 (0.000, 0.000)	1	0.006 (0.000, 0.022)	0	0.000 (0.000, 0.000)
Over 65 years	0	0.000 (0.000, 0.000)	0	0.000 (0.000, 0.000)	0	0.000 (0.000, 0.000)	0	0.000 (0.000, 0.000)
Men	3	0.004 (0.000, 0.011)	3	0.006 (0.000, 0.013)	5	0.008 (0.000, 0.017)	2	0.002 (0.000, 0.007)
Adults	0	0.000 (0.000, 0.000)	0	0.000 (0.000, 0.000)	0	0.000 (0.000, 0.000)	0	0.000 (0.000, 0.000)
0–4 years old	3	0.089 (0.000, 0.240)	3	0.134 (0.000, 0.286)	4	0.178 (0.003, 0.354)	2	0.044 (0.000, 0.166)
5–9 years old	0	0.000 (0.000, 0.000)	0	0.000 (0.000, 0.000)	0	0.000 (0.000, 0.000)	0	0.000 (0.000, 0.000)
10–14 years old	0	0.000 (0.000, 0.000)	0	0.000 (0.000, 0.000)	0	0.000 (0.000, 0.000)	0	0.000 (0.000, 0.000)
15–19 years old	0	0.000 (0.000, 0.000)	0	0.000 (0.000, 0.000)	1	0.000 (0.000, 0.059)	0	0.000 (0.000, 0.000)
20–44 years old	0	0.000 (0.000, 0.000)	0	0.000 (0.000, 0.000)	0	0.000 (0.000, 0.000)	0	0.000 (0.000, 0.000)
45–64 years old	0	0.000 (0.000, 0.000)	0	0.000 (0.000, 0.000)	0	0.000 (0.000, 0.000)	0	0.000 (0.000, 0.000)
Over 65 years	0	0.000 (0.000, 0.000)	0	0.000 (0.000, 0.000)	0	0.000 (0.000, 0.000)	0	0.000 (0.000, 0.000)

Cont. Suppl. Table 2-5.

Variables	2011		2012		2013		2014		2015	
	n	Mortality (95% CI)	n	Mortality (95% CI)	n	Mortality (95% CI)	n	Mortality (95% CI)	n	Mortality (95% CI)
All	8	0.014 (0.003, 0.025)	7	0.012 (0.001, 0.022)	7	0.012 (0.001, 0.022)	6	0.009 (0.000, 0.019)	3	0.003 (0.000, 0.010)
Adults	0	0.000 (0.000, 0.000)	0	0.000 (0.000, 0.000)	0	0.000 (0.000, 0.000)	0	0.000 (0.000, 0.000)	0	0.000 (0.000, 0.000)
0–4 years old	8	0.313 (0.072, 0.554)	7	0.268 (0.424, 0.494)	7	0.268 (0.040, 0.496)	6	0.223 (0.012, 0.434)	3	0.089 (0.000, 0.240)
5–9 years old	0	0.000 (0.000, 0.000)	0	0.000 (0.000, 0.000)	0	0.000 (0.000, 0.000)	0	0.000 (0.000, 0.000)	0	0.000 (0.000, 0.000)
10–14 years old	0	0.000 (0.000, 0.000)	0	0.000 (0.000, 0.000)	0	0.000 (0.000, 0.000)	0	0.000 (0.000, 0.000)	0	0.000 (0.000, 0.000)
15–19 years old	0	0.000 (0.000, 0.000)	0	0.000 (0.000, 0.000)	0	0.000 (0.000, 0.000)	0	0.000 (0.000, 0.000)	0	0.000 (0.000, 0.000)
20–44 years old	0	0.000 (0.000, 0.000)	0	0.000 (0.000, 0.000)	0	0.000 (0.000, 0.000)	0	0.000 (0.000, 0.000)	0	0.000 (0.000, 0.000)
45–64 years old	0	0.000 (0.000, 0.000)	0	0.000 (0.000, 0.000)	0	0.000 (0.000, 0.000)	0	0.000 (0.000, 0.000)	0	0.000 (0.000, 0.000)
Over 65 years	0	0.000 (0.000, 0.000)	0	0.000 (0.000, 0.000)	0	0.000 (0.000, 0.000)	0	0.000 (0.000, 0.000)	0	0.000 (0.000, 0.000)
Women	2	0.002 (0.000, 0.007)	1	0.000 (0.000, 0.003)	4	0.006 (0.000, 0.013)	3	0.003 (0.000, 0.010)	1	0.000 (0.000, 0.003)
Adults	0	0.000 (0.000, 0.000)	0	0.000 (0.000, 0.000)	0	0.000 (0.000, 0.000)	0	0.000 (0.000, 0.000)	0	0.000 (0.000, 0.000)
0–4 years old	2	0.044 (0.000, 0.165)	1	0.000 (0.000, 0.085)	4	0.134 (0.000, 0.306)	3	0.089 (0.000, 0.238)	1	0.000 (0.000, 0.087)
5–9 years old	0	0.000 (0.000, 0.000)	0	0.000 (0.000, 0.000)	0	0.000 (0.000, 0.000)	0	0.000 (0.000, 0.000)	0	0.000 (0.000, 0.000)
10–14 years old	0	0.000 (0.000, 0.000)	0	0.000 (0.000, 0.000)	0	0.000 (0.000, 0.000)	0	0.000 (0.000, 0.000)	0	0.000 (0.000, 0.000)
15–19 years old	0	0.000 (0.000, 0.000)	0	0.000 (0.000, 0.000)	0	0.000 (0.000, 0.000)	0	0.000 (0.000, 0.000)	0	0.000 (0.000, 0.000)
20–44 years old	0	0.000 (0.000, 0.000)	0	0.000 (0.000, 0.000)	0	0.000 (0.000, 0.000)	0	0.000 (0.000, 0.000)	0	0.000 (0.000, 0.000)
45–64 years old	0	0.000 (0.000, 0.000)	0	0.000 (0.000, 0.000)	0	0.000 (0.000, 0.000)	0	0.000 (0.000, 0.000)	0	0.000 (0.000, 0.000)
Over 65 years	0	0.000 (0.000, 0.000)	0	0.000 (0.000, 0.000)	0	0.000 (0.000, 0.000)	0	0.000 (0.000, 0.000)	0	0.000 (0.000, 0.000)
Men	6	0.010 (0.000, 0.019)	6	0.010 (0.000, 0.019)	3	0.003 (0.000, 0.010)	3	0.003 (0.000, 0.010)	2	0.001 (0.000, 0.007)
Adults	0	0.000 (0.000, 0.000)	0	0.000 (0.000, 0.000)	0	0.000 (0.000, 0.000)	0	0.000 (0.000, 0.000)	0	0.000 (0.000, 0.000)
0–4 years old	6	0.223 (0.014, 0.432)	6	0.223 (0.014, 0.432)	3	0.089 (0.000, 0.238)	3	0.089 (0.000, 0.238)	2	0.044 (0.000, 0.167)
5–9 years old	0	0.000 (0.000, 0.000)	0	0.000 (0.000, 0.000)	0	0.000 (0.000, 0.000)	0	0.000 (0.000, 0.000)	0	0.000 (0.000, 0.000)
10–14 years old	0	0.000 (0.000, 0.000)	0	0.000 (0.000, 0.000)	0	0.000 (0.000, 0.000)	0	0.000 (0.000, 0.000)	0	0.000 (0.000, 0.000)
15–19 years old	0	0.000 (0.000, 0.000)	0	0.000 (0.000, 0.000)	0	0.000 (0.000, 0.000)	0	0.000 (0.000, 0.000)	0	0.000 (0.000, 0.000)
20–44 years old	0	0.000 (0.000, 0.000)	0	0.000 (0.000, 0.000)	0	0.000 (0.000, 0.000)	0	0.000 (0.000, 0.000)	0	0.000 (0.000, 0.000)
45–64 years old	0	0.000 (0.000, 0.000)	0	0.000 (0.000, 0.000)	0	0.000 (0.000, 0.000)	0	0.000 (0.000, 0.000)	0	0.000 (0.000, 0.000)
Over 65 years	0	0.000 (0.000, 0.000)	0	0.000 (0.000, 0.000)	0	0.000 (0.000, 0.000)	0	0.000 (0.000, 0.000)	0	0.000 (0.000, 0.000)

a Age-standardized mortality rates of pulmonary venous connection were calculated using age groups according to the direct method using the estimated Korean population in 2015 as a reference.

Supplementary Table 2-6. Age-standardized mortality[a] and 95% confidence interval (CI) of congenital tricuspid stenosis overall and by sex (per 100,000).

Variables	2007		2008		2009		2010	
	n	Mortality (95% CI)	n	Mortality (95% CI)	n	Mortality (95% CI)	n	Mortality (95% CI)
All	11	0.023 (0.008, 0.037)	4	0.008 (0.000, 0.132)	10	0.022 (0.081, 0.037)	7	0.016 (0.004, 0.028)
Adults	2	0.005 (0.000, 0.016)	0	0.000 (0.000, 0.000)	5	0.016 (0.001, 0.030)	4	0.013 (0.000, 0.026)
0–4 years old	8	0.313 (0.066, 0.559)	4	0.178 (0.003, 0.354)	5	0.223 (0.027, 0.420)	3	0.089 (0.000, 0.239)
5–9 years old	1	0.000 (0.000, 0.069)	0	0.000 (0.000, 0.000)	0	0.000 (0.000, 0.000)	0	0.000 (0.000, 0.000)
10–14 years old	0	0.000 (0.000, 0.000)	0	0.000 (0.000, 0.000)	0	0.000 (0.000, 0.000)	0	0.000 (0.000, 0.000)
15–19 years old	0	0.000 (0.000, 0.000)	0	0.000 (0.000, 0.000)	0	0.000 (0.000, 0.000)	0	0.000 (0.000, 0.000)
20–44 years old	0	0.000 (0.000, 0.000)	0	0.000 (0.000, 0.000)	1	0.000 (0.000, 0.009)	0	0.000 (0.000, 0.000)
45–64 years old	0	0.000 (0.000, 0.000)	0	0.000 (0.000, 0.000)	1	0.006 (0.000, 0.022)	1	0.006 (0.000, 0.021)
Over 65 years	2	0.030 (0.000, 0.093)	0	0.000 (0.000, 0.000)	3	0.060 (0.000, 0.131)	3	0.045 (0.000, 0.113)
Women	7	0.014 (0.002, 0.026)	1	0.002 (0.000, 0.006)	7	0.016 (0.003, 0.029)	3	0.006 (0.000, 0.013)
Adults	2	0.005 (0.000, 0.016)	0	0.000 (0.000, 0.000)	4	0.013 (0.000, 0.027)	1	0.002 (0.000, 0.009)
0–4 years old	4	0.134 (0.000, 0.308)	1	0.044 (0.000, 0.132)	3	0.134 (0.000, 0.286)	2	0.044 (0.000, 0.166)
5–9 years old	1	0.000 (0.000, 0.069)	0	0.000 (0.000, 0.000)	0	0.000 (0.000, 0.000)	0	0.000 (0.000, 0.000)
10–14 years old	0	0.000 (0.000, 0.000)	0	0.000 (0.000, 0.000)	0	0.000 (0.000, 0.000)	0	0.000 (0.000, 0.000)
15–19 years old	0	0.000 (0.000, 0.000)	0	0.000 (0.000, 0.000)	0	0.000 (0.000, 0.000)	0	0.000 (0.000, 0.000)
20–44 years old	0	0.000 (0.000, 0.000)	0	0.000 (0.000, 0.000)	0	0.000 (0.000, 0.000)	0	0.000 (0.000, 0.000)
45–64 years old	0	0.000 (0.000, 0.000)	0	0.000 (0.000, 0.000)	1	0.006 (0.000, 0.022)	0	0.000 (0.000, 0.000)
Over 65 years	2	0.030 (0.000, 0.093)	0	0.000 (0.000, 0.000)	3	0.060 (0.000, 0.131)	1	0.015 (0.000, 0.054)
Men	4	0.006 (0.000, 0.014)	3	0.006 (0.000, 0.013)	3	0.004 (0.000, 0.010)	4	0.008 (0.000, 0.017)
Adults	0	0.000 (0.000, 0.000)	0	0.000 (0.000, 0.000)	1	0.000 (0.000, 0.004)	3	0.007 (0.000, 0.009)
0–4 years old	4	0.134 (0.000, 0.308)	3	0.134 (0.000, 0.286)	2	0.089 (0.000, 0.213)	1	0.000 (0.000, 0.086)
5–9 years old	0	0.000 (0.000, 0.000)	0	0.000 (0.000, 0.000)	0	0.000 (0.000, 0.000)	0	0.000 (0.000, 0.000)
10–14 years old	0	0.000 (0.000, 0.000)	0	0.000 (0.000, 0.000)	0	0.000 (0.000, 0.000)	0	0.000 (0.000, 0.000)
15–19 years old	0	0.000 (0.000, 0.000)	0	0.000 (0.000, 0.000)	0	0.000 (0.000, 0.000)	0	0.000 (0.000, 0.000)
20–44 years old	0	0.000 (0.000, 0.000)	0	0.000 (0.000, 0.000)	1	0.000 (0.000, 0.009)	0	0.000 (0.000, 0.000)
45–64 years old	0	0.000 (0.000, 0.000)	0	0.000 (0.000, 0.000)	0	0.000 (0.000, 0.000)	1	0.006 (0.000, 0.021)
Over 65 years	0	0.000 (0.000, 0.000)	0	0.000 (0.000, 0.000)	0	0.000 (0.000, 0.000)	2	0.030 (0.000, 0.086)

Cont. Suppl. Table 2-6.

Variables	2011		2012		2013		2014		2015	
	n	Mortality (95% CI)	n	Mortality (95% CI)	n	Mortality (95% CI)	n	Mortality (95% CI)	n	Mortality (95% CI)
All	5	0.010 (0.000, 0.019)	4	0.006 (0.000, 0.013)	7	0.014 (0.003, 0.024)	2	0.001 (0.000, 0.007)	6	0.011 (0.002, 0.021)
Adults	1	0.002 (0.000, 0.009)	1	0.000 (0.000, 0.004)	3	0.007 (0.000, 0.017)	0	0.000 (0.000, 0.000)	2	0.004 (0.000, 0.012)
0–4 years old	4	0.134 (0.000, 0.304)	3	0.089 (0.000, 0.237)	4	0.134 (0.000, 0.306)	2	0.044 (0.000, 0.166)	4	0.134 (0.000, 0.308)
5–9 years old	0	0.000 (0.000, 0.000)	0	0.000 (0.000, 0.000)	0	0.000 (0.000, 0.000)	0	0.000 (0.000, 0.000)	0	0.000 (0.000, 0.000)
10–14 years old	0	0.000 (0.000, 0.000)	0	0.000 (0.000, 0.000)	0	0.000 (0.000, 0.000)	0	0.000 (0.000, 0.000)	0	0.000 (0.000, 0.000)
15–19 years old	0	0.000 (0.000, 0.000)	0	0.000 (0.000, 0.000)	0	0.000 (0.000, 0.000)	0	0.000 (0.000, 0.000)	0	0.000 (0.000, 0.000)
20–44 years old	0	0.000 (0.000, 0.000)	1	0.000 (0.000, 0.010)	0	0.000 (0.000, 0.000)	0	0.000 (0.000, 0.000)	0	0.000 (0.000, 0.000)
45–64 years old	0	0.000 (0.000, 0.000)	0	0.000 (0.000, 0.000)	0	0.000 (0.000, 0.000)	0	0.000 (0.000, 0.000)	0	0.000 (0.000, 0.000)
Over 65 years	1	0.015 (0.000, 0.053)	0	0.000 (0.000, 0.000)	3	0.045 (0.000, 0.104)	0	0.000 (0.000, 0.000)	2	0.030 (0.000, 0.074)
Women	2	0.002 (0.000, 0.007)	2	0.002 (0.000, 0.007)	4	0.008 (0.000, 0.016)	0	0.000 (0.000, 0.000)	4	0.007 (0.000, 0.015)
Adults	0	0.000 (0.000, 0.000)	0	0.000 (0.000, 0.000)	1	0.002 (0.000, 0.008)	0	0.000 (0.000, 0.000)	2	0.004 (0.000, 0.012)
0–4 years old	2	0.044 (0.000, 0.165)	2	0.006 (0.000, 0.013)	3	0.089 (0.000, 0.238)	0	0.000 (0.000, 0.000)	2	0.044 (0.000, 0.167)
5–9 years old	0	0.000 (0.000, 0.000)	0	0.000 (0.000, 0.000)	0	0.000 (0.000, 0.000)	0	0.000 (0.000, 0.000)	0	0.000 (0.000, 0.000)
10–14 years old	0	0.000 (0.000, 0.000)	0	0.000 (0.000, 0.000)	0	0.000 (0.000, 0.000)	0	0.000 (0.000, 0.000)	0	0.000 (0.000, 0.000)
15–19 years old	0	0.000 (0.000, 0.000)	0	0.000 (0.000, 0.000)	0	0.000 (0.000, 0.000)	0	0.000 (0.000, 0.000)	0	0.000 (0.000, 0.000)
20–44 years old	0	0.000 (0.000, 0.000)	0	0.000 (0.000, 0.000)	0	0.000 (0.000, 0.000)	0	0.000 (0.000, 0.000)	0	0.000 (0.000, 0.000)
45–64 years old	0	0.000 (0.000, 0.000)	0	0.000 (0.000, 0.000)	0	0.000 (0.000, 0.000)	0	0.000 (0.000, 0.000)	0	0.000 (0.000, 0.000)
Over 65 years	0	0.000 (0.000, 0.000)	0	0.000 (0.000, 0.000)	1	0.015 (0.000, 0.049)	0	0.000 (0.000, 0.000)	2	0.030 (0.000, 0.074)
Men	3	0.006 (0.000, 0.013)	2	0.002 (0.000, 0.007)	3	0.006 (0.000, 0.013)	2	0.001 (0.000, 0.007)	2	0.001 (0.000, 0.007)
Adults	1	0.002 (0.000, 0.009)	1	0.000 (0.000, 0.004)	2	0.005 (0.000, 0.013)	0	0.000 (0.000, 0.000)	0	0.000 (0.000, 0.000)
0–4 years old	2	0.044 (0.000, 0.165)	1	0.000 (0.000 (0.085)	1	0.000 (0.000, 0.086)	2	0.044 (0.000, 0.166)	2	0.044 (0.000, 0.167)
5–9 years old	0	0.000 (0.000, 0.000)	0	0.000 (0.000, 0.000)	0	0.000 (0.000, 0.000)	0	0.000 (0.000, 0.000)	0	0.000 (0.000, 0.000)
10–14 years old	0	0.000 (0.000, 0.000)	0	0.000 (0.000, 0.000)	0	0.000 (0.000, 0.000)	0	0.000 (0.000, 0.000)	0	0.000 (0.000, 0.000)
15–19 years old	0	0.000 (0.000, 0.000)	0	0.000 (0.000, 0.000)	0	0.000 (0.000, 0.000)	0	0.000 (0.000, 0.000)	0	0.000 (0.000, 0.000)
20–44 years old	0	0.000 (0.000, 0.000)	1	0.000 (0.000, 0.010)	0	0.000 (0.000, 0.000)	0	0.000 (0.000, 0.000)	0	0.000 (0.000, 0.000)
45–64 years old	0	0.000 (0.000, 0.000)	0	0.000 (0.000, 0.000)	0	0.000 (0.000, 0.000)	0	0.000 (0.000, 0.000)	0	0.000 (0.000, 0.000)
Over 65 years	1	0.015 (0.000, 0.053)	0	0.000 (0.000, 0.000)	2	0.030 (0.000, 0.078)	0	0.000 (0.000, 0.000)	0	0.000 (0.000, 0.000)

a Age-standardized mortality rates of congenital tricuspid stenosis were calculated using age groups according to the direct method using the estimated Korean population in 2015 as a reference.

Supplementary Table 2-7. Age-standardized mortality[a] and 95% confidence interval (CI) of congenital stenosis of aortic valve overall and by sex (per 100,000).

Variables	2007		2008		2009		2010	
	n	Mortality (95% CI)	n	Mortality (95% CI)	n	Mortality (95% CI)	n	Mortality (95% CI)
All	12	0.031 (0.013, 0.049)	10	0.024 (0.011, 0.201)	6	0.014 (0.001, 0.027)	11	0.026 (0.010, 0.042)
Adults	9	0.033 (0.010, 0.055)	9	0.029 (0.008, 0.050)	6	0.018 (0.001, 0.035)	8	0.002 (0.007, 0.045)
0–4 years old	3	0.089 (0.000, 0.240)	1	0.044 (0.000, 0.132)	0	0.000 (0.000, 0.000)	3	0.089 (0.000, 0.239)
5–9 years old	0	0.000 (0.000, 0.000)	0	0.000 (0.000, 0.000)	0	0.000 (0.000, 0.000)	0	0.000 (0.000, 0.000)
10–14 years old	0	0.000 (0.000, 0.000)	0	0.000 (0.000, 0.000)	0	0.000 (0.000, 0.000)	0	0.000 (0.000, 0.000)
15–19 years old	0	0.000 (0.000, 0.000)	0	0.000 (0.000, 0.000)	0	0.000 (0.000, 0.000)	0	0.000 (0.000, 0.000)
20–44 years old	1	0.000 (0.000, 0.009)	1	0.000 (0.000, 0.009)	0	0.000 (0.000, 0.000)	0	0.000 (0.000, 0.000)
45–64 years old	3	0.019 (0.000, 0.048)	3	0.019 (0.000, 0.047)	1	0.006 (0.000, 0.022)	1	0.006 (0.000, 0.021)
Over 65 years	5	0.106 (0.006, 0.206)	5	0.106 (0.011, 0.201)	5	0.091 (0.000, 0.182)	7	0.137 (0.032, 0.241)
Women	3	0.008 (0.000, 0.018)	4	0.008 (0.000, 0.018)	5	0.012 (0.000, 0.024)	7	0.016 (0.004, 0.028)
Adults	3	0.011 (0.000, 0.025)	3	0.008 (0.000, 0.019)	5	0.016 (0.000, 0.031)	4	0.013 (0.000, 0.026)
0–4 years old	0	0.000 (0.000, 0.000)	1	0.044 (0.000, 0.132)	0	0.000 (0.000, 0.000)	3	0.089 (0.000, 0.239)
5–9 years old	0	0.000 (0.000, 0.000)	0	0.000 (0.000, 0.000)	0	0.000 (0.000, 0.000)	0	0.000 (0.000, 0.000)
10–14 years old	0	0.000 (0.000, 0.000)	0	0.000 (0.000, 0.000)	0	0.000 (0.000, 0.000)	0	0.000 (0.000, 0.000)
15–19 years old	0	0.000 (0.000, 0.000)	0	0.000 (0.000, 0.000)	0	0.000 (0.000, 0.000)	0	0.000 (0.000, 0.000)
20–44 years old	0	0.000 (0.000, 0.000)	1	0.000 (0.000, 0.009)	0	0.000 (0.000, 0.000)	0	0.000 (0.000, 0.000)
45–64 years old	0	0.000 (0.000, 0.000)	0	0.000 (0.000, 0.000)	1	0.006 (0.000, 0.022)	1	0.006 (0.000, 0.021)
Over 65 years	3	0.060 (0.000, 0.138)	2	0.030 (0.000, 0.090)	4	0.076 (0.000, 0.157)	3	0.045 (0.000, 0.113)
Men	9	0.020 (0.006, 0.035)	6	0.014 (0.001, 0.027)	1	0.002 (0.000, 0.007)	4	0.010 (0.000, 0.020)
Adults	6	0.019 (0.002, 0.036)	6	0.018 (0.001, 0.035)	1	0.002 (0.000, 0.009)	4	0.013 (0.000, 0.026)
0–4 years old	3	0.089 (0.000, 0.240)	0	0.000 (0.000, 0.000)	0	0.000 (0.000, 0.000)	0	0.000 (0.000, 0.000)
5–9 years old	0	0.000 (0.000, 0.000)	0	0.000 (0.000, 0.000)	0	0.000 (0.000, 0.000)	0	0.000 (0.000, 0.000)
10–14 years old	0	0.000 (0.000, 0.000)	0	0.000 (0.000, 0.000)	0	0.000 (0.000, 0.000)	0	0.000 (0.000, 0.000)
15–19 years old	0	0.000 (0.000, 0.000)	0	0.000 (0.000, 0.000)	0	0.000 (0.000, 0.000)	0	0.000 (0.000, 0.000)
20–44 years old	1	0.000 (0.000, 0.009)	0	0.000 (0.000, 0.000)	0	0.000 (0.000, 0.000)	0	0.000 (0.000, 0.000)
45–64 years old	3	0.019 (0.000, 0.048)	3	0.019 (0.000, 0.047)	0	0.000 (0.000, 0.000)	0	0.000 (0.000, 0.000)
Over 65 years	2	0.030 (0.000, 0.093)	3	0.060 (0.000, 0.134)	1	0.015 (0.000, 0.055)	4	0.076 (0.000, 0.154)

Cont. Suppl. Table 2-7.

Variables	2011 n	2011 Mortality (95% CI)	2012 n	2012 Mortality (95% CI)	2013 n	2013 Mortality (95% CI)	2014 n	2014 Mortality (95% CI)	2015 n	2015 Mortality (95% CI)
All	10	0.022 (0.007, 0.037)	12	0.026 (0.010, 0.041)	5	0.010 (0.000, 0.019)	13	0.025 (0.011, 0.040)	8	0.015 (0.004, 0.027)
Adults	8	0.023 (0.005, 0.041)	11	0.030 (0.011, 0.050)	4	0.010 (0.000, 0.021)	8	0.019 (0.004, 0.035)	5	0.012 (0.000, 0.023)
0–4 years old	2	0.044 (0.000, 0.165)	1	0.000 (0.000, 0.085)	1	0.000 (0.000, 0.086)	5	0.178 (0.000, 0.371)	3	0.089 (0.000, 0.240)
5–9 years old	0	0.000 (0.000, 0.000)	0	0.000 (0.000, 0.000)	0	0.000 (0.000, 0.000)	0	0.000 (0.000, 0.000)	0	0.000 (0.000, 0.000)
10–14 years old	0	0.000 (0.000, 0.000)	0	0.000 (0.000, 0.000)	0	0.000 (0.000, 0.000)	0	0.000 (0.000, 0.000)	0	0.000 (0.000, 0.000)
15–19 years old	0	0.000 (0.000, 0.000)	0	0.000 (0.000, 0.000)	0	0.000 (0.000, 0.000)	0	0.000 (0.000, 0.000)	0	0.000 (0.000, 0.000)
20–44 years old	0	0.000 (0.000, 0.000)	1	0.000 (0.000, 0.010)	0	0.000 (0.000, 0.000)	0	0.000 (0.000, 0.000)	0	0.000 (0.000, 0.000)
45–64 years old	1	0.006 (0.000, 0.021)	2	0.013 (0.000, 0.033)	0	0.000 (0.000, 0.000)	0	0.000 (0.000, 0.000)	0	0.000 (0.000, 0.000)
Over 65 years	7	0.121 (0.021, 0.221)	8	0.137 (0.035, 0.238)	4	0.060 (0.000, 0.129)	8	0.121 (0.029, 0.214)	5	0.076 (0.005, 0.146)
Women	3	0.006 (0.000, 0.014)	8	0.016 (0.003, 0.028)	4	0.008 (0.000, 0.016)	9	0.017 (0.005, 0.030)	4	0.007 (0.000, 0.016)
Adults	2	0.005 (0.000, 0.014)	7	0.020 (0.005, 0.035)	3	0.007 (0.000, 0.017)	5	0.012 (0.000, 0.024)	3	0.007 (0.000, 0.016)
0–4 years old	1	0.000 (0.000, 0.085)	1	0.000 (0.000, 0.085)	1	0.000 (0.000, 0.086)	4	0.134 (0.000, 0.306)	1	0.000 (0.000, 0.087)
5–9 years old	0	0.000 (0.000, 0.000)	0	0.000 (0.000, 0.000)	0	0.000 (0.000, 0.000)	0	0.000 (0.000, 0.000)	0	0.000 (0.000, 0.000)
10–14 years old	0	0.000 (0.000, 0.000)	0	0.000 (0.000, 0.000)	0	0.000 (0.000, 0.000)	0	0.000 (0.000, 0.000)	0	0.000 (0.000, 0.000)
15–19 years old	0	0.000 (0.000, 0.000)	0	0.000 (0.000, 0.000)	0	0.000 (0.000, 0.000)	0	0.000 (0.000, 0.000)	0	0.000 (0.000, 0.000)
20–44 years old	0	0.000 (0.000, 0.000)	1	0.000 (0.000, 0.010)	0	0.000 (0.000, 0.000)	0	0.000 (0.000, 0.000)	0	0.000 (0.000, 0.000)
45–64 years old	0	0.000 (0.000, 0.000)	1	0.006 (0.000, 0.020)	0	0.000 (0.000, 0.000)	0	0.000 (0.000, 0.000)	0	0.000 (0.000, 0.000)
Over 65 years	2	0.030 (0.000, 0.083)	5	0.091 (0.011, 0.171)	3	0.045 (0.000, 0.104)	5	0.076 (0.003, 0.149)	3	0.045 (0.000, 0.100)
Men	7	0.016 (0.003, 0.028)	4	0.008 (0.000, 0.017)	1	0.002 (0.000, 0.006)	4	0.007 (0.000, 0.016)	4	0.007 (0.000, 0.015)
Adults	6	0.018 (0.002, 0.033)	4	0.010 (0.000, 0.022)	1	0.002 (0.000, 0.008)	3	0.007 (0.000, 0.016)	2	0.004 (0.000, 0.012)
0–4 years old	1	0.000 (0.000, 0.085)	0	0.000 (0.000, 0.000)	0	0.000 (0.000, 0.000)	1	0.000 (0.000, 0.086)	2	0.044 (0.000, 0.167)
5–9 years old	0	0.000 (0.000, 0.000)	0	0.000 (0.000, 0.000)	0	0.000 (0.000, 0.000)	0	0.000 (0.000, 0.000)	0	0.000 (0.000, 0.000)
10–14 years old	0	0.000 (0.000, 0.000)	0	0.000 (0.000, 0.000)	0	0.000 (0.000, 0.000)	0	0.000 (0.000, 0.000)	0	0.000 (0.000, 0.000)
15–19 years old	0	0.000 (0.000, 0.000)	0	0.000 (0.000, 0.000)	0	0.000 (0.000, 0.000)	0	0.000 (0.000, 0.000)	0	0.000 (0.000, 0.000)
20–44 years old	0	0.000 (0.000, 0.000)	0	0.000 (0.000, 0.000)	0	0.000 (0.000, 0.000)	0	0.000 (0.000, 0.000)	0	0.000 (0.000, 0.000)
45–64 years old	1	0.006 (0.000, 0.021)	1	0.006 (0.000, 0.020)	0	0.000 (0.000, 0.000)	0	0.000 (0.000, 0.000)	0	0.000 (0.000, 0.000)
Over 65 years	5	0.091 (0.006, 0.175)	3	0.045 (0.000, 0.107)	1	0.015 (0.000, 0.049)	3	0.045 (0.000, 0.102)	2	0.030 (0.000, 0.074)

a Age-standardized mortality rates of congenital stenosis of aortic valve were calculated using age groups according to the direct method using the estimated Korean population in 2015 as a reference.

Supplementary Table 2-8. Age-standardized mortality[a] and 95% confidence interval (CI) of congenital insufficiency of aortic valve overall and by sex (per 100,000).

Variables	2007		2008		2009		2010	
	n	Mortality (95% CI)	n	Mortality (95% CI)	n	Mortality (95% CI)	n	Mortality (95% CI)
All	13	0.035 (0.015, 0.055)	15	0.039 (0.018, 0.060)	17	0.039 (0.019, 0.058)	14	0.034 (0.016, 0.053)
Adults	13	0.046 (0.019, 0.073)	13	0.046 (0.019, 0.072)	16	0.048 (0.023, 0.073)	13	0.042 (0.019, 0.065)
0–4 years old	0	0.000 (0.000, 0.000)	1	0.044 (0.000, 0.132)	1	0.044 (0.000, 0.132)	0	0.000 (0.000, 0.000)
5–9 years old	0	0.000 (0.000, 0.000)	0	0.000 (0.000, 0.000)	0	0.000 (0.000, 0.000)	0	0.000 (0.000, 0.000)
10–14 years old	0	0.000 (0.000, 0.000)	1	0.000 (0.000, 0.060)	0	0.000 (0.000, 0.000)	0	0.000 (0.000, 0.000)
15–19 years old	0	0.000 (0.000, 0.000)	0	0.000 (0.000, 0.000)	0	0.000 (0.000, 0.000)	1	0.000 (0.000, 0.058)
20–44 years old	0	0.000 (0.000, 0.000)	0	0.000 (0.000, 0.000)	4	0.016 (0.000, 0.036)	0	0.000 (0.000, 0.000)
45–64 years old	7	0.053 (0.009, 0.097)	4	0.026 (0.000, 0.058)	6	0.046 (0.008, 0.084)	6	0.039 (0.003, 0.076)
Over 65 years	6	0.121 (0.012, 0.231)	9	0.182 (0.054, 0.310)	6	0.121 (0.022, 0.221)	7	0.137 (0.032, 0.241)
Women	5	0.014 (0.001, 0.027)	6	0.016 (0.003, 0.030)	6	0.012 (0.001. 0.023)	6	0.014 (0.002, 0.026)
Adults	5	0.019 (0.002, 0.036)	6	0.021 (0.003, 0.039)	5	0.013 (0.000, 0.026)	5	0.015 (0.001, 0.030)
0–4 years old	0	0.000 (0.000, 0.000)	0	0.000 (0.000, 0.000)	1	0.044 (0.000, 0.132)	0	0.000 (0.000, 0.000)
5–9 years old	0	0.000 (0.000, 0.000)	0	0.000 (0.000, 0.000)	0	0.000 (0.000, 0.000)	0	0.000 (0.000, 0.000)
10–14 years old	0	0.000 (0.000, 0.000)	0	0.000 (0.000, 0.000)	0	0.000 (0.000, 0.000)	0	0.000 (0.000, 0.000)
15–19 years old	0	0.000 (0.000, 0.000)	0	0.000 (0.000, 0.000)	0	0.000 (0.000, 0.000)	1	0.000 (0.000, 0.058)
20–44 years old	0	0.000 (0.000, 0.000)	0	0.000 (0.000, 0.000)	2	0.005 (0.000, 0.019)	0	0.000 (0.000, 0.000)
45–64 years old	2	0.013 (0.000, 0.036)	2	0.013 (0.000, 0.035)	2	0.013 (0.000, 0.035)	2	0.013 (0.000, 0.034)
Over 65 years	3	0.060 (0.000, 0.138)	4	0.076 (0.000, 0.161)	1	0.015 (0.000, 0.055)	3	0.045 (0.000, 0.113)
Men	8	0.020 (0.005, 0.036)	9	0.022 (0.007, 0.038)	11	0.026 (0.010, 0.043)	8	0.018 (0.004, 0.032)
Adults	8	0.027 (0.006, 0.048)	7	0.024 (0.005, 0.043)	11	0.034 (0.013, 0.056)	8	0.023 (0.005, 0.041)
0–4 years old	0	0.000 (0.000, 0.000)	1	0.044 (0.000, 0.132)	0	0.000 (0.000, 0.000)	0	0.000 (0.000, 0.000)
5–9 years old	0	0.000 (0.000, 0.000)	0	0.000 (0.000, 0.000)	0	0.000 (0.000, 0.000)	0	0.000 (0.000, 0.000)
10–14 years old	0	0.000 (0.000, 0.000)	1	0.000 (0.000, 0.060)	0	0.000 (0.000, 0.000)	0	0.000 (0.000, 0.000)
15–19 years old	0	0.000 (0.000, 0.000)	0	0.000 (0.000, 0.000)	0	0.000 (0.000, 0.000)	0	0.000 (0.000, 0.000)
20–44 years old	0	0.000 (0.000, 0.000)	0	0.000 (0.000, 0.000)	2	0.005 (0.000, 0.019)	0	0.000 (0.000, 0.000)
45–64 years old	5	0.039 (0.002, 0.076)	2	0.013 (0.000, 0.035)	4	0.026 (0.000, 0.057)	4	0.026 (0.000, 0.056)
Over 65 years	3	0.060 (0.000, 0.138)	5	0.106 (0.011, 0.201)	5	0.091 (0.000, 0.182)	4	0.076 (0.000, 0.154)

Cont. Suppl. Table 2-8.

Variables	2011		2012		2013		2014		2015	
	n	Mortality (95% CI)	n	Mortality (95% CI)	n	Mortality (95% CI)	n	Mortality (95% CI)	n	Mortality (95% CI)
All	17	0.038 (0.019, 0.057)	14	0.030 (0.013, 0.046)	18	0.038 (0.020, 0.055)	12	0.023 (0.009, 0.038)	16	0.031 (0.015, 0.047)
Adults	16	0.046 (0.022, 0.071)	14	0.038 (0.017, 0.059)	17	0.045 (0.023, 0.067)	11	0.027 (0.010, 0.044)	14	0.034 (0.015, 0.053)
0–4 years old	1	0.000 (0.000, 0.085)	0	0.000 (0.000, 0.000)	1	0.000 (0.000, 0.086)	1	0.000 (0.000, 0.086)	2	0.044 (0.000, 0.167)
5–9 years old	0	0.000 (0.000, 0.000)	0	0.000 (0.000, 0.000)	0	0.000 (0.000, 0.000)	0	0.000 (0.000, 0.000)	0	0.000 (0.000, 0.000)
10–14 years old	0	0.000 (0.000, 0.000)	0	0.000 (0.000, 0.000)	0	0.000 (0.000, 0.000)	0	0.000 (0.000, 0.000)	0	0.000 (0.000, 0.000)
15–19 years old	0	0.000 (0.000, 0.000)	0	0.000 (0.000, 0.000)	0	0.000 (0.000, 0.000)	0	0.000 (0.000, 0.000)	0	0.000 (0.000, 0.000)
20–44 years old	1	0.000 (0.000, 0.010)	2	0.005 (0.000, 0.019)	2	0.005 (0.000, 0.019)	0	0.000 (0.000, 0.000)	0	0.000 (0.000, 0.000)
45–64 years old	7	0.046 (0.008, 0.084)	6	0.039 (0.005, 0.074)	7	0.046 (0.010, 0.082)	4	0.026 (0.000, 0.052)	5	0.026 (0.000, 0.055)
Over 65 years	8	0.152 (0.045, 0.259)	6	0.106 (0.018, 0.194)	8	0.137 (0.040, 0.233)	7	0.106 (0.020, 0.192)	9	0.137 (0.042, 0.231)
Women	7	0.016 (0.003, 0.028)	3	0.006 (0.000, 0.013)	8	0.016 (0.004, 0.028)	3	0.005 (0.000, 0.013)	7	0.013 (0.003, 0.024)
Adults	6	0.018 (0.003, 0.033)	3	0.007 (0.000, 0.017)	8	0.020 (0.005, 0.035)	3	0.007 (0.000, 0.016)	6	0.014 (0.002, 0.027)
0–4 years old	1	0.000 (0.000, 0.085)	0	0.000 (0.000, 0.000)	0	0.000 (0.000, 0.000)	0	0.000 (0.000, 0.000)	1	0.000 (0.000, 0.087)
5–9 years old	0	0.000 (0.000, 0.000)	0	0.000 (0.000, 0.000)	0	0.000 (0.000, 0.000)	0	0.000 (0.000, 0.000)	0	0.000 (0.000, 0.000)
10–14 years old	0	0.000 (0.000, 0.000)	0	0.000 (0.000, 0.000)	0	0.000 (0.000, 0.000)	0	0.000 (0.000, 0.000)	0	0.000 (0.000, 0.000)
15–19 years old	0	0.000 (0.000, 0.000)	0	0.000 (0.000, 0.000)	0	0.000 (0.000, 0.000)	0	0.000 (0.000, 0.000)	0	0.000 (0.000, 0.000)
20–44 years old	0	0.000 (0.000, 0.000)	0	0.000 (0.000, 0.000)	1	0.000 (0.000, 0.010)	0	0.000 (0.000, 0.000)	0	0.000 (0.000, 0.000)
45–64 years old	2	0.013 (0.000, 0.033)	2	0.013 (0.000, 0.033)	3	0.019 (0.000, 0.043)	1	0.006 (0.000, 0.019)	2	0.006 (0.000, 0.024)
Over 65 years	4	0.076 (0.000, 0.151)	1	0.015 (0.000, 0.051)	4	0.060 (0.000, 0.129)	2	0.030 (0.000, 0.076)	4	0.060 (0.000, 0.123)
Men	10	0.030 (0.013, 0.046)	11	0.024 (0.009, 0.038)	10	0.020 (0.006, 0.033)	9	0.017 (0.005, 0.030)	9	0.017 (0.005, 0.029)
Adults	10	0.028 (0.009, 0.047)	11	0.030 (0.012, 0.049)	9	0.022 (0.006, 0.038)	8	0.019 (0.005, 0.034)	8	0.019 (0.005, 0.033)
0–4 years old	0	0.000 (0.000, 0.000)	0	0.000 (0.000, 0.000)	1	0.000 (0.000, 0.086)	1	0.000 (0.000, 0.086)	1	0.000 (0.000, 0.087)
5–9 years old	0	0.000 (0.000, 0.000)	0	0.000 (0.000, 0.000)	0	0.000 (0.000, 0.000)	0	0.000 (0.000, 0.000)	0	0.000 (0.000, 0.000)
10–14 years old	0	0.000 (0.000, 0.000)	0	0.000 (0.000, 0.000)	0	0.000 (0.000, 0.000)	0	0.000 (0.000, 0.000)	0	0.000 (0.000, 0.000)
15–19 years old	0	0.000 (0.000, 0.000)	0	0.000 (0.000, 0.000)	0	0.000 (0.000, 0.000)	0	0.000 (0.000, 0.000)	0	0.000 (0.000, 0.000)
20–44 years old	1	0.000 (0.000, 0.010)	2	0.005 (0.000, 0.019)	1	0.000 (0.000, 0.010)	0	0.000 (0.000, 0.000)	0	0.000 (0.000, 0.000)
45–64 years old	5	0.033 (0.001, 0.065)	4	0.026 (0.000, 0.054)	4	0.026 (0.000, 0.053)	3	0.019 (0.000, 0.042)	3	0.013 (0.000, 0.035)
Over 65 years	4	0.076 (0.000, 0.151)	5	0.091 (0.011, 0.171)	4	0.060 (0.000, 0.129)	5	0.076 (0.003, 0.149)	5	0.076 (0.005, 0.146)

a Age-standardized mortality rates of congenital insufficiency of aortic valve were calculated using age groups according to the direct method using the estimated Korean population in 2015 as a reference.

Supplementary Table 2-9. Age-standardized mortality[a] and 95% confidence interval (CI) of congenital mitral stenosis overall and by sex (per 100,000).

Variables	2007		2008		2009		2010	
	n	Mortality (95% CI)	n	Mortality (95% CI)	n	Mortality (95% CI)	n	Mortality (95% CI)
All	22	0.058 (0.033, 0.083)	20	0.049 (0.027, 0.072)	8	0.016 (0.003, 0.029)	10	0.022 (0.007, 0.037)
Adults	18	0.066 (0.034, 0.097)	15	0.051 (0.024, 0.078)	4	0.013 (0.000, 0.026)	6	0.018 (0.002, 0.034)
0–4 years old	4	0.134 (0.000, 0.308)	5	0.223 (0.027, 0.419)	3	0.134 (0.000, 0.286)	3	0.089 (0.000, 0.239)
5–9 years old	0	0.000 (0.000, 0.000)	0	0.000 (0.000, 0.000)	1	0.000 (0.000, 0.075)	0	0.000 (0.000, 0.000)
10–14 years old	0	0.000 (0.000, 0.000)	0	0.000 (0.000, 0.000)	0	0.000 (0.000, 0.000)	0	0.000 (0.000, 0.000)
15–19 years old	0	0.000 (0.000, 0.000)	0	0.000 (0.000, 0.000)	0	0.000 (0.000, 0.000)	1	0.000 (0.000, 0.058)
20–44 years old	2	0.005 (0.000, 0.019)	1	0.000 (0.000, 0.009)	0	0.000 (0.000, 0.000)	0	0.000 (0.000, 0.000)
45–64 years old	5	0.039 (0.002, 0.076)	6	0.046 (0.007, 0.085)	2	0.013 (0.000, 0.035)	2	0.013 (0.000, 0.034)
Over 65 years	11	0.243 (0.095, 0.391)	8	0.167 (0.046, 0.287)	2	0.030 (0.000, 0.087)	4	0.076 (0.000, 0.154)
Women	13	0.035 (0.015, 0.055)	10	0.024 (0.009, 0.040)	5	0.010 (0.000, 0.020)	7	0.014 (0.002, 0.026)
Adults	12	0.044 (0.018, 0.069)	7	0.024 (0.006, 0.042)	2	0.005 (0.000, 0.014)	3	0.007 (0.000, 0.019)
0–4 years old	1	0.000 (0.000, 0.087)	3	0.134 (0.000, 0.286)	3	0.134 (0.000, 0.286)	3	0.089 (0.000, 0.239)
5–9 years old	0	0.000 (0.000, 0.000)	0	0.000 (0.000, 0.000)	0	0.000 (0.000, 0.000)	0	0.000 (0.000, 0.000)
10–14 years old	0	0.000 (0.000, 0.000)	0	0.000 (0.000, 0.000)	0	0.000 (0.000, 0.000)	0	0.000 (0.000, 0.000)
15–19 years old	0	0.000 (0.000, 0.000)	0	0.000 (0.000, 0.000)	0	0.000 (0.000, 0.000)	1	0.000 (0.000, 0.058)
20–44 years old	2	0.005 (0.000, 0.019)	1	0.000 (0.000, 0.009)	0	0.000 (0.000, 0.000)	0	0.000 (0.000, 0.000)
45–64 years old	2	0.013 (0.000, 0.036)	2	0.013 (0.000, 0.035)	1	0.006 (0.000, 0.022)	1	0.006 (0.000, 0.021)
Over 65 years	8	0.167 (0.041, 0.293)	4	0.076 (0.000, 0.161)	1	0.015 (0.000, 0.055)	2	0.030 (0.000, 0.086)
Men	9	0.023 (0.007, 0.038)	10	0.024 (0.008, 0.041)	3	0.006 (0.000, 0.014)	3	0.006 (0.000, 0.014)
Adults	6	0.022 (0.003, 0.040)	8	0.027 (0.007, 0.047)	2	0.005 (0.000, 0.014)	3	0.007 (0.000, 0.019)
0–4 years old	3	0.089 (0.000, 0.240)	2	0.089 (0.000, 0.213)	0	0.000 (0.000, 0.000)	0	0.000 (0.000, 0.000)
5–9 years old	0	0.000 (0.000, 0.000)	0	0.000 (0.000, 0.000)	1	0.000 (0.000, 0.075)	0	0.000 (0.000, 0.000)
10–14 years old	0	0.000 (0.000, 0.000)	0	0.000 (0.000, 0.000)	0	0.000 (0.000, 0.000)	0	0.000 (0.000, 0.000)
15–19 years old	0	0.000 (0.000, 0.000)	0	0.000 (0.000, 0.000)	0	0.000 (0.000, 0.000)	0	0.000 (0.000, 0.000)
20–44 years old	0	0.000 (0.000, 0.000)	0	0.000 (0.000, 0.000)	0	0.000 (0.000, 0.000)	0	0.000 (0.000, 0.000)
45–64 years old	3	0.019 (0.000, 0.048)	4	0.026 (0.000, 0.058)	1	0.006 (0.000, 0.022)	1	0.006 (0.000, 0.021)
Over 65 years	3	0.060 (0.000, 0.138)	4	0.076 (0.000, 0.161)	1	0.015 (0.000, 0.055)	2	0.030 (0.000, 0.086)

Cont. Suppl. Table 2-9.

Variables	2011		2012		2013		2014		2015	
	n	Mortality (95% CI)	n	Mortality (95% CI)	n	Mortality (95% CI)	n	Mortality (95% CI)	n	Mortality (95% CI)
All	8	0.018 (0.004, 0.031)	6	0.012 (0.001, 0.023)	4	0.008 (0.000, 0.016)	6	0.009 (0.000, 0.019)	6	0.009 (0.000, 0.019)
Adults	7	0.020 (0.004, 0.037)	4	0.010 (0.000, 0.022)	3	0.007 (0.000, 0.017)	2	0.004 (0.000, 0.012)	2	0.004 (0.000, 0.011)
0–4 years old	1	0.000 (0.000, 0.085)	2	0.044 (0.000, 0.165)	1	0.000 (0.000, 0.086)	4	0.134 (0.000, 0.306)	4	0.134 (0.000, 0.308)
5–9 years old	0	0.000 (0.000, 0.000)	0	0.000 (0.000, 0.000)	0	0.000 (0.000, 0.000)	0	0.000 (0.000, 0.000)	0	0.000 (0.000, 0.000)
10–14 years old	0	0.000 (0.000, 0.000)	0	0.000 (0.000, 0.000)	0	0.000 (0.000, 0.000)	0	0.000 (0.000, 0.000)	0	0.000 (0.000, 0.000)
15–19 years old	0	0.000 (0.000, 0.000)	0	0.000 (0.000, 0.000)	0	0.000 (0.000, 0.000)	0	0.000 (0.000, 0.000)	0	0.000 (0.000, 0.000)
20–44 years old	0	0.000 (0.000, 0.000)	0	0.000 (0.000, 0.000)	0	0.000 (0.000, 0.000)	1	0.000 (0.000, 0.010)	1	0.000 (0.000, 0.010)
45–64 years old	1	0.006 (0.000, 0.021)	0	0.000 (0.000, 0.000)	1	0.006 (0.000, 0.020)	0	0.000 (0.000, 0.000)	0	0.000 (0.000, 0.000)
Over 65 years	6	0.106 (0.013, 0.199)	4	0.060 (0.000, 0.132)	2	0.030 (0.000, 0.078)	1	0.015 (0.000, 0.047)	1	0.015 (0.000, 0.046)
Women	5	0.012 (0.001, 0.022)	3	0.006 (0.000, 0.013)	1	0.002 (0.000, 0.006)	2	0.001 (0.000, 0.007)	1	0.000 (0.000, 0.003)
Adults	4	0.013 (0.000, 0.025)	2	0.005 (0.000, 0.013)	1	0.002 (0.000, 0.008)	0	0.000 (0.000, 0.000)	0	0.000 (0.000, 0.000)
0–4 years old	1	0.000 (0.000, 0.085)	1	0.000 (0.000, 0.085)	0	0.000 (0.000, 0.000)	2	0.044 (0.000, 0.166)	1	0.000 (0.000, 0.087)
5–9 years old	0	0.000 (0.000, 0.000)	0	0.000 (0.000, 0.000)	0	0.000 (0.000, 0.000)	0	0.000 (0.000, 0.000)	0	0.000 (0.000, 0.000)
10–14 years old	0	0.000 (0.000, 0.000)	0	0.000 (0.000, 0.000)	0	0.000 (0.000, 0.000)	0	0.000 (0.000, 0.000)	0	0.000 (0.000, 0.000)
15–19 years old	0	0.000 (0.000, 0.000)	0	0.000 (0.000, 0.000)	0	0.000 (0.000, 0.000)	0	0.000 (0.000, 0.000)	0	0.000 (0.000, 0.000)
20–44 years old	0	0.000 (0.000, 0.000)	0	0.000 (0.000, 0.000)	0	0.000 (0.000, 0.000)	0	0.000 (0.000, 0.000)	0	0.000 (0.000, 0.000)
45–64 years old	0	0.000 (0.000, 0.000)	0	0.000 (0.000, 0.000)	0	0.000 (0.000, 0.000)	0	0.000 (0.000, 0.000)	0	0.000 (0.000, 0.000)
Over 65 years	4	0.076 (0.000, 0.151)	2	0.030 (0.000, 0.081)	1	0.015 (0.000, 0.049)	0	0.000 (0.000, 0.000)	0	0.000 (0.000, 0.000)
Men	3	0.006 (0.000, 0.014)	3	0.006 (0.000, 0.013)	3	0.006 (0.000, 0.013)	4	0.005 (0.000, 0.013)	5	0.007 (0.000, 0.016)
Adults	3	0.007 (0.000, 0.018)	2	0.005 (0.000, 0.013)	2	0.005 (0.000, 0.012)	2	0.004 (0.000, 0.012)	2	0.004 (0.000, 0.011)
0–4 years old	0	0.000 (0.000, 0.000)	1	0.000 (0.000, 0.085)	1	0.000 (0.000, 0.086)	2	0.044 (0.000, 0.166)	3	0.089 (0.000, 0.240)
5–9 years old	0	0.000 (0.000, 0.000)	0	0.000 (0.000, 0.000)	0	0.000 (0.000, 0.000)	0	0.000 (0.000, 0.000)	0	0.000 (0.000, 0.000)
10–14 years old	0	0.000 (0.000, 0.000)	0	0.000 (0.000, 0.000)	0	0.000 (0.000, 0.000)	0	0.000 (0.000, 0.000)	0	0.000 (0.000, 0.000)
15–19 years old	0	0.000 (0.000, 0.000)	0	0.000 (0.000, 0.000)	0	0.000 (0.000, 0.000)	0	0.000 (0.000, 0.000)	0	0.000 (0.000, 0.000)
20–44 years old	0	0.000 (0.000, 0.000)	0	0.000 (0.000, 0.000)	0	0.000 (0.000, 0.000)	1	0.000 (0.000, 0.010)	1	0.000 (0.000, 0.010)
45–64 years old	1	0.006 (0.000, 0.021)	0	0.000 (0.000, 0.000)	1	0.000 (0.000, 0.020)	0	0.000 (0.000, 0.000)	0	0.000 (0.000, 0.000)
Over 65 years	2	0.030 (0.000, 0.083)	2	0.030 (0.000, 0.081)	1	0.000 (0.000, 0.049)	1	0.015 (0.000, 0.047)	1	0.015 (0.000, 0.046)

a Age-standardized mortality rates of congenital mitral stenosis were calculated using age groups according to the direct method using the estimated Korean population in 2015 as a reference.

Supplementary Table 2-10. Age-standardized mortality[a] and 95% confidence interval (CI) of malformation of coronary vessels overall and by sex (per 100,000).

Variables	2007		2008		2009		2010	
	n	Mortality (95% CI)	n	Mortality (95% CI)	n	Mortality (95% CI)	n	Mortality (95% CI)
All	55	0.131 (0.095, 0.167)	50	0.120 (0.086, 0.154)	49	0.115 (0.081, 0.148)	47	0.112 (0.079, 0.144)
Adults	34	0.121 (0.079, 0.162)	31	0.108 (0.069, 0.147)	36	0.117 (0.078, 0.157)	34	0.111 (0.073, 0.149)
0–4 years old	12	0.492 (0.190, 0.793)	13	0.581 (0.265, 0.898)	7	0.313 (0.080, 0.545)	10	0.402 (0.129, 0.675)
5–9 years old	3	0.088 (0.000, 0.209)	1	0.000 (0.000, 0.073)	0	0.000 (0.000, 0.000)	2	0.044 (0.000, 0.160)
10–14 years old	4	0.082 (0.000, 0.201)	3	0.082 (0.000, 0.187)	4	0.124 (0.000, 0.247)	0	0.000 (0.000, 0.000)
15–19 years old	2	0.063 (0.000, 0.151)	2	0.031 (0.000, 0.117)	2	0.031 (0.000, 0.115)	1	0.000 (0.000, 0.058)
20–44 years old	6	0.027 (0.003, 0.051)	5	0.022 (0.000, 0.044)	5	0.022 (0.000, 0.044)	2	0.005 (0.000, 0.029)
45–64 years old	12	0.099 (0.042, 0.157)	7	0.053 (0.010, 0.095)	10	0.073 (0.024, 0.122)	9	0.066 (0.021, 0.111)
Over 65 years	16	0.350 (0.171,0.528)	19	0.411 (0.225, 0.596)	21	0.426 (0.240, 0.612)	23	0.456 (0.267, 0.645)
Women	29	0.071 (0.044, 0.098)	19	0.047 (0.025, 0.070)	23	0.055 (0.031, 0.079)	22	0.051 (0.028, 0.073)
Adults	17	0.063 (0.032, 0.094)	14	0.048 (0.021, 0.075)	19	0.064 (0.035, 0.093)	16	0.050 (0.024, 0.076)
0–4 years old	8	0.313 (0.066, 0.559)	5	0.223 (0.027, 0.419)	2	0.089 (0.000, 0.213)	6	0.223 (0.012, 0.435)
5–9 years old	2	0.044 (0.000, 0.142)	0	0.000 (0.000, 0.000)	0	0.000 (0.000, 0.000)	0	0.000 (0.000, 0.000)
10–14 years old	2	0.041 (0.000, 0.125)	0	0.000 (0.000, 0.000)	1	0.000 (0.000, 0.061)	0	0.000 (0.000, 0.000)
15–19 years old	0	0.000 (0.000, 0.000)	0	0.000 (0.000, 0.000)	1	0.000 (0.000, 0.059)	0	0.000 (0.000, 0.000)
20–44 years old	1	0.000 (0.000, 0.009)	1	0.000 (0.000,0.009)	2	0.005 (0.000, 0.019)	2	0.005 (0.000, 0.019)
45–64 years old	6	0.046 (0.005, 0.087)	3	0.019 (0.000, 0.047)	5	0.033 (0.000, 0.067)	3	0.019 (0.000, 0.045)
Over 65 years	10	0.213 (0.071, 0.354)	10	0.213 (0.078, 0.347)	12	0.243 (0.102, 0.384)	11	0.213 (0.082, 0.343)
Men	26	0.060 (0.036, 0.084)	31	0.070 (0.044, 0.096)	26	0.059 (0.036, 0.083)	25	0.059 (0.035, 0.083)
Adults	17	0.057 (0.029, 0.085)	17	0.057 (0.029, 0.085)	17	0.053 (0.026, 0.080)	18	0.058 (0.030, 0.086)
0–4 years old	4	0.134 (0.000, 0.308)	8	0.357 (0.109, 0.606)	5	0.223 (0.027, 0.420)	4	0.134 (0.000, 0.307)
5–9 years old	1	0.000 (0.000, 0.069)	1	0.000 (0.000, 0.073)	0	0.000 (0.000, 0.000)	2	0.044 (0.000, 0.160)
10–14 years old	2	0.041 (0.000, 0.125)	3	0.082 (0.000, 0.187)	3	0.082 (0.000, 0.189)	0	0.000 (0.000, 0.000)
15–19 years old	2	0.063 (0.000, 0.151)	2	0.031 (0.000, 0.117)	1	0.000 (0.000, 0.059)	1	0.000 (0.000, 0.058)
20–44 years old	5	0.022 (0.000, 0.044)	4	0.016 (0.000, 0.036)	3	0.011 (0.000, 0.028)	0	0.000 (0.000, 0.000)
45–64 years old	6	0.046 (0.005, 0.087)	4	0.026 (0.000, 0.058)	5	0.033 (0.000, 0.067)	6	0.039 (0.003, 0.076)
Over 65 years	6	0.121 (0.012, 0.231)	9	0.182 (0.054, 0.310)	9	0.182 (0.060, 0.304)	12	0.228 (0.091, 0.364)

Cont. Suppl. Table 2-10.

Variables	2011		2012		2013		2014		2015	
	n	Mortality (95% CI)	n	Mortality (95% CI)	n	Mortality (95% CI)	n	Mortality (95% CI)	n	Mortality (95% CI)
All	42	0.095 (0.065, 0.124)	45	0.098 (0.069, 0.128)	32	0.068 (0.044, 0.092)	39	0.079 (0.054, 0.104)	25	0.049 (0.029, 0.069)
Adults	30	0.091 (0.057, 0.124)	36	0.105 (0.070, 0.139)	26	0.070 (0.042, 0.099)	28	0.072 (0.045, 0.099)	18	0.044 (0.023, 0.065)
0–4 years old	12	0.492 (0.196, 0.787)	9	0.357 (0.101, 0.614)	5	0.178 (0.000, 0.371)	11	0.447 (0.161, 0.732)	7	0.268 (0.037, 0.498)
5–9 years old	0	0.000 (0.000, 0.000)	0	0.000 (0.000, 0.000)	0	0.000 (0.000, 0.000)	0	0.000 (0.000, 0.000)	0	0.000 (0.000, 0.000)
10–14 years old	0	0.000 (0.000, 0.000)	0	0.000 (0.000, 0.000)	1	0.000 (0.000, 0.072)	0	0.000 (0.000, 0.000)	0	0.000 (0.000, 0.000)
15–19 years old	0	0.000 (0.000, 0.000)	0	0.000 (0.000, 0.000)	0	0.000 (0.000, 0.000)	0	0.000 (0.000, 0.000)	0	0.000 (0.000, 0.000)
20–44 years old	3	0.011 (0.000, 0.028)	3	0.011 (0.000, 0.028)	2	0.005 (0.000, 0.019)	3	0.111 (0.000, 0.028)	0	0.000 (0.000, 0.000)
45–64 years old	7	0.046 (0.008, 0.084)	11	0.073 (0.026, 0.119)	6	0.039 (0.006, 0.073)	9	0.059 (0.020, 0.099)	6	0.033 (0.001, 0.064)
Over 65 years	20	0.380 (0.211, 0.549)	22	0.395 (0.227, 0.563)	18	0.304 (0.159, 0.449)	16	0.258 (0.128, 0.389)	12	0.182 (0.073, 0.291)
Women	18	0.040 (0.021, 0.060)	21	0.046 (0.026, 0.066)	14	0.030 (0.013, 0.046)	19	0.037 (0.020, 0.055)	8	0.015 (0.004, 0.026)
Adults	12	0.036 (0.014, 0.058)	16	0.046 (0.022, 0.069)	12	0.032 (0.013, 0.052)	13	0.032 (0.013, 0.051)	2	0.004 (0.000, 0.012)
0–4 years old	6	0.223 (0.014, 0.432)	5	0.178 (0.000, 0.369)	1	0.000 (0.000, 0.086)	6	0.223 (0.012, 0.434)	6	0.223 (0.010, 0.437)
5–9 years old	0	0.000 (0.000, 0.000)	0	0.000 (0.000, 0.000)	0	0.000 (0.000, 0.000)	0	0.000 (0.000, 0.000)	0	0.000 (0.000, 0.000)
10–14 years old	0	0.000 (0.000, 0.000)	0	0.000 (0.000, 0.000)	1	0.000 (0.000, 0.072)	0	0.000 (0.000, 0.000)	0	0.000 (0.000, 0.000)
15–19 years old	0	0.000 (0.000, 0.000)	0	0.000 (0.000, 0.000)	0	0.000 (0.000, 0.000)	0	0.000 (0.000, 0.000)	0	0.000 (0.000, 0.000)
20–44 years old	0	0.000 (0.000, 0.000)	1	0.000 (0.000, 0.010)	0	0.000 (0.000, 0.000)	1	0.000 (0.000, 0.010)	0	0.000 (0.000, 0.000)
45–64 years old	2	0.013 (0.000, 0.033)	4	0.026 (0.000, 0.054)	2	0.013 (0.000, 0.032)	4	0.026 (0.000, 0.052)	0	0.000 (0.000, 0.000)
Over 65 years	10	0.182 (0.063, 0.301)	11	0.197 (0.079, 0.316)	10	0.167 (0.059, 0.275)	8	0.121 (0.029, 0.214)	2	0.030 (0.000, 0.074)
Men	24	0.052 (0.030, 0.074)	24	0.052 (0.030, 0.073)	18	0.038 (0.020, 0.055)	20	0.039 (0.021, 0.057)	5	0.009 (0.008, 0.018)
Adults	18	0.052 (0.026, 0.077)	20	0.056 (0.031, 0.082)	14	0.038 (0.017, 0.058)	15	0.037 (0.017, 0.057)	4	0.009 (0.000, 0.020)
0–4 years old	6	0.223 (0.014, 0.432)	4	0.134 (0.000, 0.304)	4	0.134 (0.000, 0.306)	5	0.178 (0.000, 0.371)	1	0.000 (0.000, 0.087)
5–9 years old	0	0.000 (0.000, 0.000)	0	0.000 (0.000, 0.000)	0	0.000 (0.000, 0.000)	0	0.000 (0.000, 0.000)	0	0.000 (0.000, 0.000)
10–14 years old	0	0.000 (0.000, 0.000)	0	0.000 (0.000, 0.000)	0	0.000 (0.000, 0.000)	0	0.000 (0.000, 0.000)	0	0.000 (0.000, 0.000)
15–19 years old	0	0.000 (0.000, 0.000)	0	0.000 (0.000, 0.000)	0	0.000 (0.000, 0.000)	0	0.000 (0.000, 0.000)	0	0.000 (0.000, 0.000)
20–44 years old	3	0.011 (0.000, 0.028)	2	0.005 (0.000, 0.019)	2	0.005 (0.000, 0.019)	2	0.005 (0.000, 0.020)	0	0.000 (0.000, 0.000)
45–64 years old	5	0.033 (0.001, 0.065)	7	0.046 (0.009, 0.083)	4	0.026 (0.000, 0.053)	5	0.033 (0.003, 0.062)	0	0.000 (0.000, 0.000)
Over 65 years	10	0.182 (0.063, 0.302)	11	0.197 (0.079, 0.316)	8	0.137 (0.040, 0.233)	8	0.121 (0.029, 0.214)	4	0.060 (0.000, 0.123)

a Age-standardized mortality rates of malformation of coronary vessels were calculated using age groups according to the direct method using the estimated Korean population in 2015 as a reference.

Supplementary Table 2-11. Age-standardized mortality[a] and 95% confidence interval (CI) of stenosis or malformation of aorta overall and by sex (per 100,000).

Variables	2007		2008		2009		2010	
	n	Mortality (95% CI)	n	Mortality (95% CI)	n	Mortality (95% CI)	n	Mortality (95% CI)
All	43	0.117 (0.081, 0.152)	37	0.097 (0.065, 0.129)	30	0.078 (0.049, 0.106)	41	0.098 (0.067, 0.128)
Adults	34	0.129 (0.085, 0.173)	33	0.116 (0.075, 0.157)	26	0.091 (0.055, 0.126)	34	0.108 (0.071, 0.145)
0–4 years old	7	0.268 (0.038, 0.498)	1	0.044 (0.000, 0.132)	3	0.134 (0.000, 0.286)	6	0.223 (0.021, 0.435)
5–9 years old	1	0.00 (0.000, 0.069)	1	0.000 (0.000, 0.073)	0	0.000 (0.000, 0.000)	1	0.000 (0.000, 0.081)
10–14 years old	0	0.000 (0.000, 0.000)	2	0.041 (0.000, 0.127)	1	0.000 (0.000, 0.061)	0	0.000 (0.000, 0.000)
15–19 years old	1	0.031 (0.000, 0.094)	0	0.000 (0.000, 0.000)	0	0.000 (0.000, 0.000)	0	0.000 (0.000, 0.000)
20–44 years old	3	0.011 (0.00, 0.027)	3	0.011 (0.000, 0.028)	0	0.000 (0.000, 0.000)	3	0.011 (0.000, 0.028)
45–64 years old	8	0.066 (0.019, 0.113)	8	0.059 (0.014, 0.105)	5	0.033 (0.000, 0.067)	12	0.086 (0.034, 0.138)
Over 65 years	23	0.517 (0.303, 0.731)	22	0.471 (0.272, 0.671)	21	0.426 (0.240, 0.612)	19	0.380 (0.208, 0.552)
Women	20	0.054 (0.029, 0.078)	17	0.045 (0.022, 0.068)	13	0.034 (0.015, 0.054)	22	0.053 (0.030, 0.075)
Adults	17	0.063 (0.032, 0.094)	16	0.057 (0.027, 0.086)	12	0.042 (0.018, 0.067)	17	0.055 (0.028, 0.072)
0–4 years old	3	0.089 (0.000, 0.240)	1	0.044 (0.000, 0.132)	1	0.044 (0.000, 0.132)	5	0.178 (0.000, 0.372)
5–9 years old	0	0.000 (0.000, 0.000)	0	0.000 (0.000, 0.000)	0	0.000 (0.000, 0.000)	0	0.000 (0.000, 0.000)
10–14 years old	0	0.000 (0.000, 0.000)	0	0.000 (0.000, 0.000)	0	0.000 (0.000, 0.000)	0	0.000 (0.000, 0.000)
15–19 years old	0	0.000 (0.000, 0.000)	0	0.000 (0.000, 0.000)	0	0.000 (0.000, 0.000)	0	0.000 (0.000, 0.000)
20–44 years old	2	0.005 (0.000, 0.019)	2	0.005 (0.000, 0.019)	0	0.000 (0.000, 0.000)	0	0.000 (0.000, 0.000)
45–64 years old	4	0.033 (0.000, 0.066)	0	0.000 (0.000, 0.000)	0	0.000 (0.000, 0.000)	7	0.046 (0.007, 0.085)
Over 65 years	11	0.243 (0.095, 0.391)	14	0.289 (0.129, 0.448)	12	0.243 (0.102, 0.384)	10	0.197 (0.073, 0.322)
Men	23	0.060 (0.034, 0.086)	20	0.049 (0.027, 0.072)	17	0.041 (0.020, 0.061)	19	0.044 (0.024, 0.065)
Adults	17	0.063 (0.031, 0.094)	17	0.059 (0.030, 0.088)	14	0.048 (0.022, 0.073)	17	0.052 (0.027, 0.078)
0–4 years old	4	0.134 (0.000, 0.308)	0	0.000 (0.000, 0.000)	2	0.089 (0.000, 0.213)	1	0.000 (0.000, 0.086)
5–9 years old	1	0.000 (0.000, 0.069)	1	0.000 (0.000, 0.073)	0	0.000 (0.000, 0.000)	1	0.000 (0.000, 0.081)
10–14 years old	0	0.000 (0.000, 0.000)	2	0.041 (0.000, 0.127)	1	0.000 (0.000, 0.06)	0	0.000 (0.000, 0.000)
15–19 years old	1	0.031 (0.000, 0.094)	0	0.000 (0.000, 0.000)	0	0.000 (0.000, 0.000)	0	0.000 (0.000, 0.000)
20–44 years old	1	0.000 (0.000, 0.019)	1	0.000 (0.000, 0.009)	0	0.000 (0.000, 0.000)	3	0.011 (0.000, 0.028)
45–64 years old	4	0.033 (0.000, 0.066)	8	0.059 (0.014, 0.105)	5	0.033 (0.000, 0.067)	5	0.033 (0.000, 0.066)
Over 65 years	12	0.258 (0.104, 0.413)	8	0.167 (0.046, 0.287)	9	0.183 (0.06, 0.304)	9	0.167 (0.049, 0.285)

Cont. Suppl. Table 2-11.

Variables	2011		2012		2013		2014		2015	
	n	Mortality (95% CI)	n	Mortality (95% CI)	n	Mortality (95% CI)	n	Mortality (95% CI)	n	Mortality (95% CI)
All	39	0.091 (0.061, 0.120)	28	0.062 (0.038, 0.086)	31	0.066 (0.042, 0.089)	26	0.053 (0.032, 0.074)	22	0.043 (0.024, 0.062)
Adults	32	0.101 (0.066, 0.137)	24	0.069 (0.040, 0.098)	23	0.065 (0.038, 0.092)	21	0.054 (0.030, 0.079)	16	0.039 (0.019, 0.059)
0–4 years old	7	0.268 (0.043, 0.493)	4	0.134 (0.000, 0.304)	7	0.268 (0.040, 0.496)	5	0.178 (0.000, 0.371)	6	0.223 (0.010, 0.437)
5–9 years old	0	0.000 (0.000, 0.000)	0	0.000 (0.000, 0.000)	0	0.000 (0.000, 0.000)	0	0.000 (0.000, 0.000)	0	0.000 (0.000, 0.000)
10–14 years old	0	0.000 (0.000, 0.000)	0	0.000 (0.000, 0.000)	1	0.000 (0.000, 0.07)	0	0.000 (0.000, 0.000)	0	0.000 (0.000, 0.000)
15–19 years old	0	0.000 (0.000, 0.000)	0	0.000 (0.000, 0.000)	0	0.000 (0.000, 0.000)	0	0.000 (0.000, 0.000)	0	0.000 (0.000, 0.000)
20–44 years old	2	0.005 (0.000, 0.019)	1	0.000 (0.000, 0.010)	0	0.000 (0.000, 0.000)	0	0.000 (0.000, 0.000)	0	0.000 (0.000, 0.000)
45–64 years old	5	0.033 (0.001, 0.065)	5	0.033 (0.001, 0.064)	3	0.019 (0.000, 0.043)	6	0.039 (0.007, 0.072)	1	0.000 (0.000, 0.012)
Over 65 years	25	0.471 (0.282, 0.660)	18	0.319 (0.167, 0.471)	20	0.334 (0.182, 0.487)	15	0.243 (0.117, 0.369)	15	0.228(0.106, 0.350)
Women	18	0.042 (0.022, 0.063)	12	0.026 (0.010, 0.041)	12	0.026 (0.010, 0.041)	10	0.019 (0.006, 0.032)	12	0.023 (0.009, 0.037)
Adults	15	0.046 (0.022, 0.071)	11	0.030 (0.011, 0.050)	11	0.030 (0.011. 0.048)	8	0.019 (0.005, 0.034)	8	0.019 (0.005, 0.034)
0–4 years old	3	0.089 (0.000, 0.237)	1	0.000 (0.000, 0.085)	1	0.000 (0.000, 0.086)	2	0.044 (0.000, 0.166)	4	0.134 (0.000, 0.308)
5–9 years old	0	0.000 (0.000, 0.000)	0	0.000 (0.000, 0.000)	0	0.000 (0.000, 0.000)	0	0.000 (0.000, 0.000)	0	0.000 (0.000, 0.000)
10–14 years old	0	0.000 (0.000, 0.000)	0	0.000 (0.000, 0.000)	0	0.000 (0.000, 0.000)	0	0.000 (0.000, 0.000)	0	0.000 (0.000, 0.000)
15–19 years old	0	0.000 (0.000, 0.000)	0	0.000 (0.000, 0.000)	0	0.000 (0.000, 0.000)	0	0.000 (0.000, 0.000)	0	0.000 (0.000, 0.000)
20–44 years old	0	0.000 (0.000, 0.000)	1	0.000 (0.000, 0.01)	0	0.000 (0.000, 0.000)	0	0.000 (0.000, 0.000)	0	0.000 (0.000, 0.000)
45–64 years old	1	0.006 (0.000, 0.021)	2	0.013 (0.000, 0.033)	2	0.013 (0.000, 0.032)	2	0.013 (0.000, 0.031)	1	0.000 (0.000, 0.012)
Over 65 years	14	0.258 (0.117, 0.400)	8	0.137 (0.035, 0.238)	9	0.152 (0.049, 0.254)	6	0.091 (0.011, 0.171)	7	0.106 (0.023, 0.189)
Men	21	0.048 (0.027, 0.069)	16	0.036 (0.018, 0.054)	19	0.040 (0.021, 0.058)	16	0.031 (0.015, 0.048)	10	0.019 (0.006, 0.032)
Adults	17	0.052 (0.026, 0.077)	13	0.038 (0.017, 0.059)	12	0.032 (0.013, 0.052)	13	0.032 (0.013, 0.051)	8	0.019 (0.005, 0.034)
0–4 years old	4	0.134 (0.000, 0.304)	3	0.089 (0.000, 0.237)	6	0.223 (0.012, 0.434)	3	0.089 (0.000, 0.238)	2	0.044 (0.000, 0.167)
5–9 years old	0	0.000 (0.000, 0.000)	0	0.000 (0.000, 0.000)	0	0.000 (0.000, 0.000)	0	0.000 (0.000, 0.000)	0	0.000 (0.000, 0.000)
10–14 years old	0	0.000 (0.000, 0.000)	0	0.000 (0.000, 0.000)	1	0.000 (0.000, 0.072)	0	0.000 (0.000, 0.000)	0	0.000 (0.000, 0.000)
15–19 years old	0	0.000 (0.000, 0.000)	0	0.000 (0.000, 0.000)	0	0.000 (0.000, 0.000)	0	0.000 (0.000, 0.000)	0	0.000 (0.000, 0.000)
20–44 years old	2	0.005 (0.000, 0.019)	0	0.000 (0.000, 0.000)	0	0.000 (0.000, 0.000)	0	0.000 (0.000, 0.000)	0	0.000 (0.000, 0.000)
45–64 years old	4	0.026 (0.000, 0.055)	3	0.019 (0.000, 0.044)	1	0.006 (0.000, 0.020)	4	0.026 (0.000, 0.052)	0	0.000 (0.000, 0.000)
Over 65 years	11	0.197 (0.072, 0.323)	10	0.182 (0.069, 0.296)	11	0.182 (0.069, 0.295)	9	0.137 (0.039, 0.234)	8	0.121 (0.032, 0.210)

a Age-standardized mortality rates of stenosis or malformation of aorta were calculated using age groups according to the direct method using the estimated Korean population in 2015 as a reference.

Supplementary Table 2-12. Age-standardized mortality[a] and 95% confidence interval (CI) of Tetralogy of Fallot overall and by sex (per 100,000).

Variables	2007		2008		2009		2010	
	n	Mortality (95% CI)	n	Mortality (95% CI)	n	Mortality (95% CI)	n	Mortality (95% CI)
All	15	0.031 (0.014, 0.048)	8	0.016 (0.004, 0.029)	14	0.028 (0.013, 0.044)	11	0.020 (0.007, 0.033)
Adults	8	0.024 (0.006, 0.043)	3	0.008 (0.000, 0.019)	5	0.013 (0.000, 0.026)	4	0.007 (0.000, 0.018)
0–4 years old	4	0.134 (0.000, 0.308)	4	0.017 (0.003, 0.354)	7	0.313 (0.080, 0.545)	7	0.268 (0.039, 0.497)
5–9 years old	2	0.044 (0.000, 0.142)	0	0.000 (0.000, 0.000)	1	0.000 (0.000, 0.075)	0	0.000 (0.000, 0.000)
10–14 years old	0	0.000 (0.000, 0.000)	0	0.000 (0.000, 0.000)	0	0.000 (0.000, 0.000)	0	0.000 (0.000, 0.000)
15–19 years old	1	0.031 (0.000, 0.094)	1	0.000 (0.000, 0.061)	1	0.000 (0.000, 0.059)	0	0.000 (0.000, 0.000)
20–44 years old	4	0.016 (0.000, 0.036)	1	0.000 (0.000, 0.009)	2	0.005 (0.000, 0.019)	3	0.011 (0.000, 0.028)
45–64 years old	1	0.006 (0.000, 0.023)	1	0.006 (0.000, 0.022)	3	0.019 (0.000, 0.046)	1	0.006 (0.000, 0.021)
Over 65 years	3	0.060 (0.000, 0.138)	1	0.015 (0.000, 0.057)	0	0.000 (0.000, 0.000)	0	0.000 (0.000, 0.000)
Women	7	0.014 (0.002, 0.026)	4	0.008 (0.000, 0017)	5	0.008 (0.000, 0.016)	6	0.010 (0.000, 0.019)
Adults	3	0.008 (0.000, 0.020)	3	0.008 (0.000, 0.019)	2	0.002 (0.000, 0.018)	2	0.002 (0.000, 0.009)
0–4 years old	2	0.044 (0.000, 0.167)	1	0.044 (0.000, 0.132)	3	0.134 (0.000, 0.286)	4	0.134 (0.000, 0.307)
5–9 years old	1	0.000 (0.000, 0.069)	0	0.000 (0.000, 0.000)	0	0.000 (0.000, 0.000)	0	0.000 (0.000, 0.000)
10–14 years old	0	0.000 (0.000, 0.000)	0	0.000 (0.000, 0.000)	0	0.000 (0.000, 0.000)	0	0.000 (0.000, 0.000)
15–19 years old	1	0.031 (0.000, 0.094)	0	0.000 (0.000, 0.000)	0	0.000 (0.000, 0.000)	0	0.000 (0.000, 0.000)
20–44 years old	1	0.000 (0.000, 0.009)	1	0.000 (0.000, 0.009)	2	0.005 (0.000, 0.019)	2	0.005 (0.000, 0.019)
45–64 years old	0	0.000 (0.000, 0.000)	1	0.006 (0.000, 0.022)	0	0.000 (0.000, 0.000)	0	0.000 (0.000, 0.000)
Over 65 years	2	0.030 (0.000, 0.093)	1	0.015 (0.000, 0.057)	0	0.000 (0.000, 0.000)	0	0.000 (0.000, 0.000)
Men	8	0.016 (0.004, 0.028)	4	0.006 (0.000, 0.014)	9	0.018 (0.005, 0.031)	5	0.010 (0.001, 0.019)
Adults	5	0.013 (0000, 0.027)	0	0.000 (0.000, 0.000)	3	0.008 (0.000, 0.018)	2	0.005 (0.000, 0.012)
0–4 years old	2	0.044 (0.000, 0.167)	3	0.134 (0.000, 0.286)	4	0.178 (0.003, 0.354)	3	0.089 (0.000, 0.239)
5–9 years old	1	0.000 (0.000, 0.069)	0	0.000 (0.000, 0.000)	1	0.000 (0.000, 0.075)	0	0.000 (0.000, 0.000)
10–14 years old	0	0.000 (0.000, 0.000)	0	0.000 (0.000, 0.000)	0	0.000 (0.000, 0.000)	0	0.000 (0.000, 0.000)
15–19 years old	0	0.000 (0.000, 0.000)	1	0.000 (0.000, 0.061)	1	0.000 (0.000, 0.059)	0	0.000 (0.000, 0.000)
20–44 years old	3	0.011 (0.000, 0.027)	0	0.000 (0.000, 0.000)	0	0.000 (0.000, 0.000)	1	0.000 (0.000, 0.009)
45–64 years old	1	0.006 (0.000, 0.023)	0	0.000 (0.000, 0.000)	3	0.019 (0.000, 0.046)	1	0.006 (0.000, 0.021)
Over 65 years	1	0.015 (0.000, 0.059)	0	0.000 (0.000, 0.000)	0	0.000 (0.000, 0.000)	0	0.000 (0.000, 0.000)

Cont. Suppl. Table 2-12.

Variables	2011		2012		2013		2014		2015	
	n	Mortality (95% CI)	n	Mortality (95% CI)	n	Mortality (95% CI)	n	Mortality (95% CI)	n	Mortality (95% CI)
All	13	0.026 (0.011, 0.041)	6	0.012 (0.002, 0.021)	12	0.024 (0.010, 0.037)	5	0.007 (0.000, 0.016)	4	0.005 (0.000, 0.013)
Adults	5	0.013 (0.000, 0.026)	2	0.005 (0.000, 0.012)	5	0.012 (0.000, 0.024)	2	0.004 (0.000, 0.012)	0	0.000 (0.000, 0.000)
0–4 years old	7	0.268 (0.043, 0.493)	4	0.134 (0.000, 0.304)	7	0.268 (0.040, 0.496)	3	0.089 (0.000, 0.238)	4	0.134 (0.000, 0.308)
5–9 years old	0	0.000 (0.000, 0.000)	0	0.000 (0.000, 0.000)	0	0.000 (0.000, 0.000)	0	0.000 (0.000, 0.000)	0	0.000 (0.000, 0.000)
10–14 years old	0	0.000 (0.000, 0.000)	0	0.000 (0.000, 0.000)	0	0.000 (0.000, 0.000)	0	0.000 (0.000, 0.000)	0	0.000 (0.000, 0.000)
15–19 years old	1	0.000 (0.000, 0.058)	0	0.000 (0.000, 0.000)	0	0.000 (0.000, 0.000)	0	0.000 (0.000, 0.000)	0	0.000 (0.000, 0.000)
20–44 years old	1	0.000 (0.000, 0.101)	0	0.000 (0.000, 0.000)	1	0.000 (0.000, 0.010)	1	0.000 (0.000, 0.010)	0	0.000 (0.000, 0.000)
45–64 years old	2	0.013 (0.000, 0.033)	2	0.013 (0.000, 0.033)	2	0.013 (0.000, 0.032)	0	0.000 (0.000, 0.000)	0	0.000 (0.000, 0.000)
Over 65 years	2	0.030 (0.000, 0.083)	0	0.000 (0.000, 0.000)	2	0.030 (0.000, 0.078)	1	0.015 (0.000, 0.047)	0	0.000 (0.000, 0.000)
Women	4	0.008 (0.000, 0.016)	4	0.008 (0.000, 0.016)	6	0.012 (0.002, 0.021)	2	0.001 (0.000, 0.007)	2	0.001 (0.000, 0.007)
Adults	1	0.002 (0.000, 0.009)	2	0.005 (0.000, 0.012)	2	0.005 (0.000, 0.012)	0	0.000 (0.000, 0.000)	0	0.000 (0.000, 0.000)
0–4 years old	3	0.089 (0.000, 0.237)	2	0.044 (0.000, 0.165)	4	0.134 (0.000, 0.306)	2	0.044 (0.000, 0.166)	2	0.044 (0.000, 0.167)
5–9 years old	0	0.000 (0.000, 0.000)	0	0.000 (0.000, 0.000)	0	0.000 (0.000, 0.000)	0	0.000 (0.000, 0.000)	0	0.000 (0.000, 0.000)
10–14 years old	0	0.000 (0.000, 0.000)	0	0.000 (0.000, 0.000)	0	0.000 (0.000, 0.000)	0	0.000 (0.000, 0.000)	0	0.000 (0.000, 0.000)
15–19 years old	0	0.000 (0.000, 0.000)	0	0.000 (0.000, 0.000)	0	0.000 (0.000, 0.000)	0	0.000 (0.000, 0.000)	0	0.000 (0.000, 0.000)
20–44 years old	0	0.000 (0.000, 0.000)	0	0.000 (0.000, 0.000)	0	0.000 (0.000, 0.000)	0	0.000 (0.000, 0.000)	0	0.000 (0.000, 0.000)
45–64 years old	0	0.000 (0.000, 0.000)	2	0.013 (0.000, 0.033)	1	0.006 (0.000, 0.020)	0	0.000 (0.000, 0.000)	0	0.000 (0.000, 0.000)
Over 65 years	1	0.015 (0.000, 0.053)	0	0.000 (0.000, 0.000)	1	0.015 (0.000, 0.049)	0	0.000 (0.000, 0.000)	0	0.000 (0.000, 0.000)
Men	9	0.018 (0.005, 0.030)	2	0.002 (0.000, 0.007)	6	0.012 (0.002, 0.021)	3	0.005 (0.000, 0.012)	2	0.001 (0.000, 0.007)
Adults	4	0.010 (0.000, 0.021)	0	0.000 (0.000, 0.000)	3	0.007 (0.000, 0.012)	2	0.004 (0.000, 0.012)	0	0.000 (0.000, 0.000)
0–4 years old	4	0.134 (0.000, 0.304)	2	0.044 (0.000, 0.165)	3	0.089 (0.000, 0.238)	1	0.000 (0.000, 0.086)	2	0.044 (0.000, 0.167)
5–9 years old	0	0.000 (0.000, 0.000)	0	0.000 (0.000, 0.000)	0	0.000 (0.000, 0.000)	0	0.000 (0.000, 0.000)	0	0.000 (0.000, 0.000)
10–14 years old	0	0.000 (0.000, 0.000)	0	0.000 (0.000, 0.000)	0	0.000 (0.000, 0.000)	0	0.000 (0.000, 0.000)	0	0.000 (0.000, 0.000)
15–19 years old	1	0.000 (0.000, 0.058)	0	0.000 (0.000, 0.000)	0	0.000 (0.000, 0.000)	0	0.000 (0.000, 0.000)	0	0.000 (0.000, 0.000)
20–44 years old	1	0.000 (0.000, 0.010)	0	0.000 (0.000, 0.000)	1	0.000 (0.000, 0.010)	1	0.000 (0.000, 0.010)	0	0.000 (0.000, 0.000)
45–64 years old	2	0.013 (0.000, 0.033)	0	0.000 (0.000, 0.000)	1	0.006 (0.000, 0.020)	0	0.000 (0.000, 0.000)	0	0.000 (0.000, 0.000)
Over 65 years	1	0.015 (0.000, 0.053)	0	0.000 (0.000, 0.000)	1	0.015 (0.000, 0.049)	1	0.015 (0.000, 0.047)	0	0.000 (0.000, 0.000)

a Age-standardized mortality rates of tetralogy of fallot were calculated using age groups according to the direct method using the estimated Korean population in 2015 as a reference.

Supplementary Table 2-13. Age-standardized mortality[a] and 95% confidence interval (CI) of Ebstein anomaly overall and by sex (per 100,000).

Variables	2007		2008		2009		2010	
	n	Mortality (95% CI)	n	Mortality (95% CI)	n	Mortality (95% CI)	n	Mortality (95% CI)
All	13	0.031 (0.012, 0.049)	11	0.024 (0.009, 0.040)	7	0.016 (0.003, 0.029)	7	0.014 (0.002, 0.026)
Adults	11	0.035 (0.012, 0.058)	8	0.024 (0.005, 0.043)	5	0.016 (0.001, 0.030)	3	0.007 (0.000, 0.019)
0–4 years old	2	0.044 (0.000, 0.167)	2	0.089 (0.000, 0.213)	2	0.089 (0.000, 0.213)	4	0.134 (0.000, 0.307)
5–9 years old	0	0.000 (0.000, 0.000)	1	0.000 (0.000, 0.073)	0	0.000 (0.000, 0.000)	0	0.000 (0.000, 0.000)
10–14 years old	0	0.000 (0.000, 0.000)	0	0.000 (0.000, 0.000)	0	0.000 (0.000, 0.000)	0	0.000 (0.000, 0.000)
15–19 years old	0	0.000 (0.000, 0.000)	0	0.000 (0.000, 0.000)	0	0.000 (0.000, 0.000)	0	0.000 (0.000, 0.000)
20–44 years old	3	0.011 (0.000, 0.027)	1	0.000 (0.000, 0.009)	1	0.000 (0.000, 0.009)	0	0.000 (0.000, 0.000)
45–64 years old	3	0.019 (0.000, 0.048)	5	0.039 (0.004, 0.075)	1	0.006 (0.000, 0.022)	1	0.006 (0.000, 0.021)
Over 65 years	5	0.106 (0.006, 0.206)	2	0.030 (0.000, 0.090)	3	0.060 (0.000, 0.131)	2	0.030 (0.000, 0.086)
Women	6	0.014 (0.001, 0.028)	6	0.014 (0.001, 0.027)	3	0.006 (0.000, 0.013)	4	0.008 (0.000, 0.017)
Adults	5	0.016 (0.000, 0.033)	5	0.016 (0.000, 0.031)	2	0.005 (0.000, 0.013)	2	0.005 (0.000, 0.014)
0–4 years old	1	0.000 (0.000, 0.087)	1	0.044 (0.000, 0.132)	1	0.044 (0.000, 0.132)	2	0.044 (0.000, 0.166)
5–9 years old	0	0.000 (0.000, 0.000)	0	0.000 (0.000, 0.000)	0	0.000 (0.000, 0.000)	0	0.000 (0.000, 0.000)
10–14 years old	0	0.000 (0.000, 0.000)	0	0.000 (0.000, 0.000)	0	0.000 (0.000, 0.000)	0	0.000 (0.000, 0.000)
15–19 years old	0	0.000 (0.000, 0.000)	0	0.000 (0.000, 0.000)	0	0.000 (0.000, 0.000)	0	0.000 (0.000, 0.000)
20–44 years old	1	0.000 (0.000, 0.009)	0	0.000 (0.000, 0.000)	1	0.000 (0.000, 0.009)	0	0.000 (0.000, 0.000)
45–64 years old	0	0.000 (0.000, 0.000)	3	0.019 (0.000, 0.047)	0	0.000 (0.000, 0.000)	0	0.000 (0.000, 0.000)
Over 65 years	4	0.076 (0.000, 0.165)	2	0.030 (0.000, 0.090)	1	0.015 (0.000, 0.055)	2	0.030 (0.000, 0.086)
Men	7	0.016 (0.003, 0.029)	5	0.010 (0.000, 0.019)	4	0.008 (0.000, 0.018)	3	0.006 (0.000, 0.013)
Adults	6	0.019 (0.003, 0.035)	3	0.008 (0.000, 0.018)	3	0.008 (0.000, 0.019)	1	0.002 (0.000, 0.008)
0–4 years old	1	0.000 (0.000, 0.087)	1	0.044 (0.000, 0.132)	1	0.044 (0.000, 0.132)	2	0.044 (0.000, 0.166)
5–9 years old	0	0.000 (0.000, 0.000)	1	0.000 (0.000, 0.073)	0	0.000 (0.000, 0.000)	0	0.000 (0.000, 0.000)
10–14 years old	0	0.000 (0.000, 0.000)	0	0.000 (0.000, 0.000)	0	0.000 (0.000, 0.000)	0	0.000 (0.000, 0.000)
15–19 years old	0	0.000 (0.000, 0.000)	0	0.000 (0.000, 0.000)	0	0.000 (0.000, 0.000)	0	0.000 (0.000, 0.000)
20–44 years old	2	0.005 (0.000, 0.019)	1	0.000 (0.000, 0.009)	0	0.000 (0.000, 0.000)	0	0.000 (0.000, 0.000)
45–64 years old	3	0.019 (0.000, 0.048)	2	0.013 (0.000, 0.035)	1	0.006 (0.000, 0.022)	1	0.006 (0.000, 0.021)
Over 65 years	1	0.015 (0.000, 0.059)	0	0.000 (0.000, 0.000)	2	0.030 (0.000, 0.087)	0	0.000 (0.000, 0.000)

Cont. Suppl. Table 2-13.

Variables	2011		2012		2013		2014		2015	
	n	Mortality (95% CI)	n	Mortality (95% CI)	n	Mortality (95% CI)	n	Mortality (95% CI)	n	Mortality (95% CI)
All	8	0.018 (0.005, 0.031)	7	0.014 (0.002, 0.025)	3	0.006 (0.000, 0.013)	2	0.003 (0.000, 0.009)	4	0.005 (0.000, 0.013)
Adults	6	0.018 (0.003, 0.033)	4	0.010 (0.000, 0.021)	1	0.002 (0.000, 0.008)	1	0.002 (0.000, 0.007)	2	0.002 (0.000, 0.008)
0–4 years old	2	0.044 (0.000, 0.165)	3	0.089 (0.000, 0.237)	2	0.044 (0.000, 0.166)	1	0.000 (0.000, 0.086)	2	0.044 (0.000, 0.167)
5–9 years old	0	0.000 (0.000, 0.000)	0	0.000 (0.000, 0.000)	0	0.000 (0.000, 0.000)	0	0.000 (0.000, 0.000)	0	0.000 (0.000, 0.000)
10–14 years old	0	0.000 (0.000, 0.000)	0	0.000 (0.000, 0.000)	0	0.000 (0.000, 0.000)	0	0.000 (0.000, 0.000)	0	0.000 (0.000, 0.000)
15–19 years old	0	0.000 (0.000, 0.000)	0	0.000 (0.000, 0.000)	0	0.000 (0.000, 0.000)	0	0.000 (0.000, 0.000)	0	0.000 (0.000, 0.000)
20–44 years old	0	0.000 (0.000, 0.000)	0	0.000 (0.000, 0.000)	0	0.000 (0.000, 0.000)	0	0.000 (0.000, 0.000)	2	0.005 (0.000, 0.020)
45–64 years old	3	0.019 (0.000, 0.044)	2	0.013 (0.000, 0.033)	0	0.000 (0.000, 0.000)	0	0.000 (0.000, 0.000)	0	0.000 (0.000, 0.000)
Over 65 years	3	0.045 (0.000, 0.111)	2	0.030 (0.000, 0.081)	1	0.015 (0.000, 0.049)	1	0.015 (0.000, 0.047)	0	0.000 (0.000, 0.000)
Women	5	0.010 (0.000, 0.020)	3	0.006 (0.000, 0.013)	2	0.002 (0.00, 0.007)	2	0.003 (0.000, 0.009)	1	0.000 (0.000, 0.003)
Adults	4	0.010 (0.000, 0.022)	2	0.005 (0.000, 0.013)	0	0.000 (0.000, 0.000)	1	0.002 (0.000, 0.007)	1	0.000 (0.000, 0.004)
0–4 years old	1	0.000 (0.000, 0.085)	1	0.000 (0.000, 0.085)	2	0.044 (0.000, 0.166)	1	0.000 (0.000, 0.086)	0	0.000 (0.000, 0.000)
5–9 years old	0	0.000 (0.000, 0.000)	0	0.000 (0.000, 0.000)	0	0.000 (0.000, 0.000)	0	0.000 (0.000, 0.000)	0	0.000 (0.000, 0.000)
10–14 years old	0	0.000 (0.000, 0.000)	0	0.000 (0.000, 0.000)	0	0.000 (0.000, 0.000)	0	0.000 (0.000, 0.000)	0	0.000 (0.000, 0.000)
15–19 years old	0	0.000 (0.000, 0.000)	0	0.000 (0.000, 0.000)	0	0.000 (0.000, 0.000)	0	0.000 (0.000, 0.000)	0	0.000 (0.000, 0.000)
20–44 years old	0	0.000 (0.000, 0.000)	0	0.000 (0.000, 0.000)	0	0.000 (0.000, 0.000)	0	0.000 (0.000, 0.000)	1	0.000 (0.010, 0.907)
45–64 years old	1	0.006 (0.000, 0.021)	1	0.006 (0.000, 0.020)	0	0.000 (0.000, 0.000)	0	0.000 (0.000, 0.000)	0	0.000 (0.000, 0.000)
Over 65 years	3	0.045 (0.000, 0.111)	1	0.015 (0.000, 0.051)	0	0.000 (0.000, 0.000)	1	0.015 (0.000, 0.047)	0	0.000 (0.000, 0.000)
Men	3	0.006 (0.000, 0.013)	4	0.008 (0.000, 0.016)	1	0.002 (0.000, 0.006)	0	0.000 (0.000, 0.000)	3	0.003 (0.000, 0.010)
Adults	2	0.005 (0.000, 0.132)	2	0.005 (0.000, 0.013)	1	0.002 (0.000, 0.008)	0	0.000 (0.000, 0.000)	1	0.000 (0.000, 0.004)
0–4 years old	1	0.000 (0.000, 0.085)	2	0.044 (0.000, 0.165)	0	0.000 (0.000, 0.000)	0	0.000 (0.000, 0.000)	2	0.044 (0.000, 0.167)
5–9 years old	0	0.000 (0.000, 0.000)	0	0.000 (0.000, 0.000)	0	0.000 (0.000, 0.000)	0	0.000 (0.000, 0.000)	0	0.000 (0.000, 0.000)
10–14 years old	0	0.000 (0.000, 0.000)	0	0.000 (0.000, 0.000)	0	0.000 (0.000, 0.000)	0	0.000 (0.000, 0.000)	0	0.000 (0.000, 0.000)
15–19 years old	0	0.000 (0.000, 0.000)	0	0.000 (0.000, 0.000)	0	0.000 (0.000, 0.000)	0	0.000 (0.000, 0.000)	0	0.000 (0.000, 0.000)
20–44 years old	0	0.000 (0.000, 0.000)	0	0.000 (0.000, 0.000)	0	0.000 (0.000, 0.000)	0	0.000 (0.000, 0.000)	1	0.000 (0.000, 0.010)
45–64 years old	2	0.013 (0.000, 0.033)	1	0.006 (0.000, 0.020)	0	0.000 (0.000, 0.000)	0	0.000 (0.000, 0.000)	0	0.000 (0.000, 0.000)
Over 65 years	0	0.000 (0.000, 0.000)	1	0.015 (0.000, 0.051)	1	0.015 (0.000, 0.049)	0	0.000 (0.000, 0.000)	0	0.000 (0.000, 0.000)

a Age-standardized mortality rates of ebstein anomaly were calculated using age groups according to the direct method using the estimated Korean population in 2015 as a reference.

Supplementary Table 2-14. Age-standardized mortality[a] and 95% confidence interval (CI) of transposition of the great arteries overall and by sex (per 100,000).

Variables	2007		2008		2009		2010	
	n	Mortality (95% CI)	n	Mortality (95% CI)	n	Mortality (95% CI)	n	Mortality (95% CI)
All	10	0.023 (0.007, 0.038)	12	0.029 (0.011, 0.046)	11	0.024 (0.009, 0.040)	10	0.020 (0.007, 0.033)
Adults	7	0.022 (0.003, 0.040)	7	0.024 (0.005, 0.043)	6	0.018 (0.002, 0.035)	4	0.010 (0.000, 0.022)
0–4 years old	3	0.089 (0.000, 0.240)	5	0.223 (0.027, 0.419)	5	0.223 (0.027, 0.420)	6	0.223 (0.012, 0.435)
5–9 years old	0	0.000 (0.000, 0.000)	0	0.000 (0.000, 0.000)	0	0.000 (0.000, 0.000)	0	0.000 (0.000, 0.000)
10–14 years old	0	0.000 (0.000, 0.000)	0	0.000 (0.000, 0.000)	0	0.000 (0.000, 0.000)	0	0.000 (0.000, 0.000)
15–19 years old	0	0.000 (0.000, 0.000)	0	0.000 (0.000, 0.000)	0	0.000 (0.000, 0.000)	0	0.000 (0.000, 0.000)
20–44 years old	3	0.011 (0.000, 0.027)	0	0.000 (0.000, 0.000)	1	0.000 (0.000, 0.009)	1	0.000 (0.000, 0.009)
45–64 years old	0	0.000 (0.000, 0.000)	2	0.013 (0.000, 0.035)	1	0.006 (0.000, 0.022)	2	0.013 (0.000, 0.034)
Over 65 years	4	0.076 (0.000, 0.165)	5	0.106 (0.011, 0.201)	4	0.076 (0.000, 0.157)	1	0.015 (0.000, 0.054)
Women	5	0.010 (0.000, 0.021)	7	0.016 (0.002, 0.030)	5	0.010 (0.000, 0.020)	5	0.010 (0.000, 0.019)
Adults	4	0.011 (0.000, 0.024)	5	0.016 (0.000, 0.032)	3	0.008 (0.000, 0.019)	2	0.005 (0.000, 0.013)
0–4 years old	1	0.000 (0.000, 0.087)	2	0.089 (0.000, 0.213)	2	0.089 (0.000, 0.213)	3	0.089 (0.000, 0.239)
5–9 years old	0	0.000 (0.000, 0.000)	0	0.000 (0.000, 0.000)	0	0.000 (0.000, 0.000)	0	0.000 (0.000, 0.000)
10–14 years old	0	0.000 (0.000, 0.000)	0	0.000 (0.000, 0.000)	0	0.000 (0.000, 0.000)	0	0.000 (0.000, 0.000)
15–19 years old	0	0.000 (0.000, 0.000)	0	0.000 (0.000, 0.000)	0	0.000 (0.000, 0.000)	0	0.000 (0.000, 0.000)
20–44 years old	2	0.005 (0.000, 0.019)	0	0.000 (0.000, 0.000)	1	0.000 (0.000, 0.009)	1	0.000 (0.000, 0.009)
45–64 years old	0	0.000 (0.000, 0.000)	1	0.006 (0.000, 0.022)	0	0.000 (0.000, 0.000)	0	0.000 (0.000, 0.000)
Over 65 years	2	0.030 (0.000, 0.093)	4	0.076 (0.000, 0.161)	2	0.030 (0.000, 0.087)	1	0.015 (0.000, 0.054)
Men	5	0.010 (0.000, 0.021)	5	0.010 (0.000, 0.020)	6	0.012 (0.000, 0.023)	5	0.010 (0.000, 0.019)
Adults	3	0.008 (0.000, 0.020)	2	0.005 (0.000, 0.015)	3	0.008 (0.000, 0.019)	2	0.005 (0.000, 0.013)
0–4 years old	2	0.044 (0.000, 0.167)	3	0.134 (0.000, 0.286)	3	0.134 (0.000, 0.286)	3	0.089 (0.000, 0.239)
5–9 years old	0	0.000 (0.000, 0.000)	0	0.000 (0.000, 0.000)	0	0.000 (0.000, 0.000)	0	0.000 (0.000, 0.000)
10–14 years old	0	0.000 (0.000, 0.000)	0	0.000 (0.000, 0.000)	0	0.000 (0.000, 0.000)	0	0.000 (0.000, 0.000)
15–19 years old	0	0.000 (0.000, 0.000)	0	0.000 (0.000, 0.000)	0	0.000 (0.000, 0.000)	0	0.000 (0.000, 0.000)
20–44 years old	1	0.000 (0.000, 0.009)	0	0.000 (0.000, 0.000)	0	0.000 (0.000, 0.000)	0	0.000 (0.000, 0.000)
45–64 years old	0	0.000 (0.000, 0.000)	1	0.006 (0.000, 0.022)	1	0.006 (0.000, 0.022)	2	0.013 (0.000, 0.034)
Over 65 years	2	0.030 (0.000, 0.093)	1	0.015 (0.000, 0.057)	2	0.030 (0.000, 0.087)	0	0.000 (0.000, 0.000)

Cont. Suppl. Table 2-14.

Variables	2011		2012		2013		2014		2015	
	n	Mortality (95% CI)	n	Mortality (95% CI)	n	Mortality (95% CI)	n	Mortality (95% CI)	n	Mortality (95% CI)
All	9	0.018 (0.006, 0.030)	7	0.014 (0.002, 0.025)	9	0.018 (0.005, 0.030)	10	0.019 (0.007, 0.032)	4	0.007 (0.000, 0.015)
Adults	3	0.007 (0.000, 0.018)	3	0.007 (0.000, 0.018)	3	0.007 (0.000, 0.017)	3	0.007 (0.000, 0.016)	1	0.002 (0.000, 0.012)
0–4 years old	5	0.178 (0.000, 0.369)	4	0.134 (0.000, 0.304)	6	0.223 (0.012, 0.434)	7	0.268 (0.040, 0.496)	3	0.089 (0.000, 0.240)
5–9 years old	0	0.000 (0.000, 0.000)	0	0.000 (0.000, 0.000)	0	0.000 (0.000, 0.000)	0	0.000 (0.000, 0.000)	0	0.000 (0.000, 0.000)
10–14 years old	1	0.000 (0.000, 0.065)	0	0.000 (0.000, 0.000)	0	0.000 (0.000, 0.000)	0	0.000 (0.000, 0.000)	0	0.000 (0.000, 0.000)
15–19 years old	0	0.000 (0.000, 0.000)	0	0.000 (0.000, 0.000)	0	0.000 (0.000, 0.000)	0	0.000 (0.000, 0.000)	0	0.000 (0.000, 0.000)
20–44 years old	0	0.000 (0.000, 0.000)	0	0.000 (0.000, 0.000)	0	0.000 (0.000, 0.000)	0	0.000 (0.000, 0.000)	0	0.000 (0.000, 0.000)
45–64 years old	2	0.013 (0.000, 0.033)	0	0.000 (0.000, 0.000)	1	0.006 (0.000, 0.020)	1	0.006 (0.000, 0.019)	0	0.000 (0.000, 0.000)
Over 65 years	1	0.015 (0.000, 0.053)	3	0.045 (0.000, 0.107)	2	0.030 (0.000, 0.078)	2	0.030 (0.000, 0.076)	1	0.015 (0.000, 0.046)
Women	3	0.004 (0.000, 0.010)	4	0.008 (0.000, 0.016)	3	0.006 (0.000, 0.012)	3	0.005 (0.000, 0.012)	1	0.000 (0.000, 0.003)
Adults	1	0.002 (0.000, 0.008)	2	0.005 (0.000, 0.013)	1	0.002 (0.000, 0.007)	1	0.002 (0.000, 0.007)	0	0.000 (0.000, 0.000)
0–4 years old	1	0.000 (0.000, 0.085)	2	0.044 (0.000, 0.165)	2	0.044 (0.000, 0.166)	2	0.044 (0.000, 0.166)	1	0.000 (0.000, 0.087)
5–9 years old	0	0.000 (0.000, 0.000)	0	0.000 (0.000, 0.000)	0	0.000 (0.000, 0.000)	0	0.000 (0.000, 0.000)	0	0.000 (0.000, 0.000)
10–14 years old	1	0.000 (0.000, 0.065)	0	0.000 (0.000, 0.000)	0	0.000 (0.000, 0.000)	0	0.000 (0.000, 0.000)	0	0.000 (0.000, 0.000)
15–19 years old	0	0.000 (0.000, 0.000)	0	0.000 (0.000, 0.000)	0	0.000 (0.000, 0.000)	0	0.000 (0.000, 0.000)	0	0.000 (0.000, 0.000)
20–44 years old	0	0.000 (0.000, 0.000)	0	0.000 (0.000, 0.000)	0	0.000 (0.000, 0.000)	0	0.000 (0.000, 0.000)	0	0.000 (0.000, 0.000)
45–64 years old	1	0.006 (0.000, 0.021)	0	0.000 (0.000, 0.000)	1	0.006 (0.000, 0.020)	0	0.000 (0.000, 0.000)	0	0.000 (0.000, 0.000)
Over 65 years	0	0.000 (0.000, 0.000)	2	0.030 (0.000, 0.081)	0	0.000 (0.000, 0.000)	1	0.015 (0.000, 0.047)	0	0.000 (0.000, 0.000)
Men	6	0.012 (0.001, 0.022)	3	0.006 (0.000, 0.013)	6	0.012 (0.002, 0.021)	7	0.013 (0.003, 0.024)	3	0.005 (0.000, 0.012)
Adults	2	0.005 (0.000, 0.013)	1	0.002 (0.000, 0.008)	2	0.005 (0.000, 0.013)	2	0.004 (0.000, 0.012)	1	0.002 (0.000, 0.007)
0–4 years old	4	0.134 (0.000, 0.304)	2	0.044 (0.000, 0.165)	4	0.134 (0.000, 0.306)	5	0.178 (0.000, 0.371)	2	0.044 (0.000, 0.167)
5–9 years old	0	0.000 (0.000, 0.000)	0	0.000 (0.000, 0.000)	0	0.000 (0.000, 0.000)	0	0.000 (0.000, 0.000)	0	0.000 (0.000, 0.000)
10–14 years old	0	0.000 (0.000, 0.000)	0	0.000 (0.000, 0.000)	0	0.000 (0.000, 0.000)	0	0.000 (0.000, 0.000)	0	0.000 (0.000, 0.000)
15–19 years old	0	0.000 (0.000, 0.000)	0	0.000 (0.000, 0.000)	0	0.000 (0.000, 0.000)	0	0.000 (0.000, 0.000)	0	0.000 (0.000, 0.000)
20–44 years old	0	0.000 (0.000, 0.000)	0	0.000 (0.000, 0.000)	0	0.000 (0.000, 0.000)	0	0.000 (0.000, 0.000)	0	0.000 (0.000, 0.000)
45–64 years old	1	0.006 (0.000, 0.021)	0	0.000 (0.000, 0.000)	0	0.000 (0.000, 0.000)	1	0.006 (0.000, 0.019)	0	0.000 (0.000, 0.000)
Over 65 years	1	0.015 (0.000, 0.053)	1	0.015 (0.000, 0.051)	2	0.030 (0.000, 0.078)	1	0.015 (0.000, 0.047)	1	0.015 (0.000, 0.046)

a Age-standardized mortality rates of transposition of the great arteries were calculated using age groups according to the direct method using the estimated Korean population in 2015 as a reference.

Supplementary Table 2-15. Age-standardized mortality[a] and 95% confidence interval (CI) of Eisenmenger syndrome overall and by sex (per 100,000).

Variables	2007		2008		2009		2010	
	n	Mortality (95% CI)	n	Mortality (95% CI)	n	Mortality (95% CI)	n	Mortality (95% CI)
All	37	0.092 (0.061, 0.122)	32	0.078 (0.050, 0.107)	31	0.074 (0.047, 0.100)	25	0.059 (0.035, 0.083)
Adults	34	0.112 (0.073, 0.152)	32	0.103 (0.066, 0.140)	30	0.093 (0.059, 0.128)	24	0.074 (0.043, 0.104)
0–4 years old	1	0.000 (0.000, 0.087)	0	0.000 (0.000, 0.000)	1	0.044 (0.000, 0.132)	1	0.000 (0.000, 0.086)
5–9 years old	0	0.000 (0.000, 0.000)	0	0.000 (0.000, 0.000)	0	0.000 (0.000, 0.000)	0	0.000 (0.000, 0.000)
10–14 years old	1	0.000 (0.000, 0.059)	0	0.000 (0.000, 0.000)	0	0.000 (0.000, 0.000)	0	0.000 (0.000, 0.000)
15–19 years old	1	0.031 (0.000, 0.094)	0	0.000 (0.000, 0.000)	0	0.000 (0.000, 0.000)	0	0.000 (0.000, 0.000)
20–44 years old	10	0.044 (0.013, 0.075)	9	0.044 (0.014, 0.073)	8	0.038 (0.010, 0.066)	3	0.011 (0.000, 0.028)
45–64 years old	13	0.106 (0.046, 0.166)	11	0.086 (0.033, 0.139)	10	0.073 (0.024, 0.122)	9	0.066 (0.021, 0.111)
Over 65 years	11	0.243 (0.095, 0.391)	12	0.258 (0.111, 0.406)	12	0.243 (0.102, 0.384)	12	0.228 (0.091, 0.364)
Women	21	0.050 (0.027, 0.073)	17	0.041 (0.020, 0.062)	16	0.037 (0.018, 0.055)	13	0.030 (0.013, 0.047)
Adults	19	0.063 (0.033, 0.092)	17	0.054 (0.027, 0.081)	15	0.045 (0.021, 0.069)	12	0.037 (0.015, 0.058)
0–4 years old	1	0.000 (0.000, 0.087)	0	0.000 (0.000, 0.000)	1	0.044 (0.000, 0.132)	1	0.000 (0.000, 0.086)
5–9 years old	0	0.000 (0.000, 0.000)	0	0.000 (0.000, 0.000)	0	0.000 (0.000, 0.000)	0	0.000 (0.000, 0.000)
10–14 years old	1	0.000 (0.000, 0.059)	0	0.000 (0.000, 0.000)	0	0.000 (0.000, 0.000)	0	0.000 (0.000, 0.000)
15–19 years old	0	0.000 (0.000, 0.000)	0	0.000 (0.000, 0.000)	0	0.000 (0.000, 0.000)	0	0.000 (0.000, 0.000)
20–44 years old	6	0.027 (0.003, 0.051)	5	0.022 (0.000, 0.044)	5	0.022 (0.000, 0.044)	2	0.005 (0.000, 0.019)
45–64 years old	7	0.053 (0.009, 0.097)	5	0.039 (0.004, 0.075)	4	0.026 (0.000, 0.057)	4	0.026 (0.000, 0.056)
Over 65 years	6	0.121 (0.012, 0.231)	7	0.137 (0.024, 0.249)	6	0.121 (0.022, 0.221)	6	0.106 (0.010, 0.202)
Men	16	0.039 (0.019, 0.060)	15	0.037 (0.018, 0.056)	15	0.037 (0.018, 0.055)	12	0.028 (0.011, 0.045)
Adults	15	0.049 (0.023, 0.076)	15	0.048 (0.023, 0.074)	15	0.048 (0.023, 0.072)	12	0.037 (0.015, 0.058)
0–4 years old	0	0.000 (0.000, 0.000)	0	0.000 (0.000, 0.000)	0	0.000 (0.000, 0.000)	0	0.000 (0.000, 0.000)
5–9 years old	0	0.000 (0.000, 0.000)	0	0.000 (0.000, 0.000)	0	0.000 (0.000, 0.000)	0	0.000 (0.000, 0.000)
10–14 years old	0	0.000 (0.000, 0.000)	0	0.000 (0.000, 0.000)	0	0.000 (0.000, 0.000)	0	0.000 (0.000, 0.000)
15–19 years old	1	0.031 (0.000, 0.094)	0	0.000 (0.000, 0.000)	0	0.000 (0.000, 0.000)	0	0.000 (0.000, 0.000)
20–44 years old	4	0.016 (0.000, 0.036)	4	0.016 (0.000, 0.036)	3	0.011 (0.000, 0.028)	1	0.000 (0.000, 0.009)
45–64 years old	6	0.046 (0.005, 0.087)	6	0.046 (0.007, 0.085)	6	0.046 (0.008, 0.084)	5	0.033 (0.000, 0.066)
Over 65 years	5	0.106 (0.006, 0.206)	5	0.106 (0.011, 0.201)	6	0.121 (0.022, 0.221)	6	0.106 (0.010, 0.202)

Cont. Suppl. Table 2-15.

Variables	2011		2012		2013		2014		2015	
	n	Mortality (95% CI)	n	Mortality (95% CI)	n	Mortality (95% CI)	n	Mortality (95% CI)	n	Mortality (95% CI)
All	29	0.064 (0.040, 0.089)	20	0.042 (0.022, 0.061)	23	0.048 (0.027, 0.068)	21	0.041 (0.023, 0.060)	15	0.029 (0.014, 0.045)
Adults	27	0.080 (0.050, 0.111)	18	0.051 (0.027, 0.075)	21	0.055 (0.031, 0.080)	21	0.052 (0.029, 0.075)	15	0.037 (0.017, 0.056)
0–4 years old	2	0.044 (0.000, 0.165)	1	0.000 (0.000, 0.085)	0	0.000 (0.000, 0.000)	0	0.000 (0.000, 0.000)	0	0.000 (0.000, 0.000)
5–9 years old	0	0.000 (0.000, 0.000)	0	0.000 (0.000, 0.000)	0	0.000 (0.000, 0.000)	0	0.000 (0.000, 0.000)	0	0.000 (0.000, 0.000)
10–14 years old	0	0.000 (0.000, 0.000)	1	0.000 (0.000, 0.068)	0	0.000 (0.000, 0.000)	0	0.000 (0.000, 0.000)	0	0.000 (0.000, 0.000)
15–19 years old	0	0.000 (0.000, 0.000)	0	0.000 (0.000, 0.000)	2	0.031 (0.000, 0.116)	0	0.000 (0.000, 0.000)	0	0.000 (0.000, 0.000)
20–44 years old	5	0.022 (0.000, 0.044)	4	0.016 (0.000, 0.036)	3	0.011 (0.000, 0.028)	4	0.016 (0.000, 0.037)	2	0.005 (0.000, 0.020)
45–64 years old	9	0.059 (0.016, 0.103)	4	0.026 (0.000, 0.054)	9	0.059 (0.019, 0.100)	6	0.039 (0.007, 0.072)	5	0.026 (0.000, 0.055)
Over 65 years	13	0.243 (0.107, 0.379)	10	0.182 (0.069, 0.296)	9	0.152 (0.049, 0.254)	11	0.182 (0.074, 0.290)	8	0.121 (0.032, 0.210)
Women	12	0.026 (0.010, 0.042)	13	0.026 (0.010, 0.041)	11	0.022 (0.008, 0.035)	10	0.019 (0.006, 0.032)	9	0.015 (0.004, 0.027)
Adults	12	0.033 (0.013, 0.054)	12	0.030 (0.011, 0.049)	10	0.025 (0.008, 0.042)	10	0.024 (0.008, 0.041)	9	0.019 (0.005, 0.034)
0–4 years old	0	0.000 (0.000, 0.000)	0	0.000 (0.000, 0.000)	0	0.000 (0.000, 0.000)	0	0.000 (0.000, 0.000)	0	0.000 (0.000, 0.000)
5–9 years old	0	0.000 (0.000, 0.000)	0	0.000 (0.000, 0.000)	0	0.000 (0.000, 0.000)	0	0.000 (0.000, 0.000)	0	0.000 (0.000, 0.000)
10–14 years old	0	0.000 (0.000, 0.000)	1	0.000 (0.000, 0.068)	0	0.000 (0.000, 0.000)	0	0.000 (0.000, 0.000)	0	0.000 (0.000, 0.000)
15–19 years old	0	0.000 (0.000, 0.000)	0	0.000 (0.000, 0.000)	1	0.000 (0.000, 0.059)	0	0.000 (0.000, 0.000)	0	0.000 (0.000, 0.000)
20–44 years old	3	0.011 (0.000, 0.028)	4	0.016 (0.000, 0.036)	1	0.000 (0.000, 0.010)	1	0.000 (0.000, 0.010)	2	0.005 (0.000, 0.020)
45–64 years old	4	0.026 (0.000, 0.055)	3	0.019 (0.000, 0.044)	4	0.026 (0.000, 0.053)	3	0.019 (0.000, 0.042)	4	0.019 (0.000, 0.045)
Over 65 years	5	0.091 (0.006, 0.175)	5	0.091 (0.011, 0.171)	5	0.076 (0.000, 0.152)	6	0.091 (0.011, 0.171)	3	0.045 (0.000, 0.100)
Men	17	0.038 (0.019, 0.057)	7	0.016 (0.004, 0.028)	12	0.024 (0.009, 0.038)	11	0.021 (0.008, 0.035)	6	0.011 (0.001, 0.021)
Adults	15	0.044 (0.021, 0.067)	6	0.017 (0.003, 0.032)	11	0.027 (0.010, 0.045)	11	0.027 (0.010, 0.044)	6	0.014 (0.002, 0.027)
0–4 years old	2	0.044 (0.000, 0.165)	1	0.000 (0.000, 0.085)	0	0.000 (0.000, 0.000)	0	0.000 (0.000, 0.000)	0	0.000 (0.000, 0.000)
5–9 years old	0	0.000 (0.000, 0.000)	0	0.000 (0.000, 0.000)	0	0.000 (0.000, 0.000)	0	0.000 (0.000, 0.000)	0	0.000 (0.000, 0.000)
10–14 years old	0	0.000 (0.000, 0.000)	0	0.000 (0.000, 0.000)	0	0.000 (0.000, 0.000)	0	0.000 (0.000, 0.000)	0	0.000 (0.000, 0.000)
15–19 years old	0	0.000 (0.000, 0.000)	0	0.000 (0.000, 0.000)	1	0.000 (0.000, 0.059)	0	0.000 (0.000, 0.000)	0	0.000 (0.000, 0.000)
20–44 years old	2	0.005 (0.000, 0.019)	0	0.000 (0.000, 0.000)	2	0.005 (0.000, 0.019)	3	0.011 (0.000, 0.028)	0	0.000 (0.000, 0.000)
45–64 years old	5	0.033 (0.001, 0.065)	1	0.006 (0.000, 0.020)	5	0.033 (0.002, 0.063)	3	0.019 (0.000, 0.042)	1	0.000 (0.000, 0.012)
Over 65 years	8	0.152 (0.045, 0.259)	5	0.091 (0.011, 0.171)	4	0.060 (0.000, 0.129)	5	0.076 (0.003, 0.149)	5	0.076 (0.005, 0.146)

a Age-standardized mortality rates of Eisenmenger syndrome were calculated using age groups according to the direct method using the estimated Korean population in 2015 as a reference.

Supplementary Table 2-16. Age-standardized mortality[a] and 95% confidence interval (CI) of double outlet right ventricle overall and by sex (per 100,000).

Variables	2007		2008		2009		2010	
	n	Mortality (95% CI)	n	Mortality (95% CI)	n	Mortality (95% CI)	n	Mortality (95% CI)
All	9	0.016 (0.004, 0.029)	8	0.014 (0.003, 0.025)	5	0.010 (0.001, 0.019)	3	0.004 (0.000, 0.010)
Adults	3	0.008 (0.000, 0.018)	1	0.000 (0.000, 0.004)	2	0.005 (0.000, 0.013)	0	0.000 (0.000, 0.000)
0–4 years old	4	0.134 (0.000, 0.308)	6	0.268 (0.053, 0.483)	3	0.134 (0.000, 0.286)	3	0.089 (0.000, 0.239)
5–9 years old	1	0.000 (0.000, 0.069)	0	0.000 (0.000, 0.000)	0	0.000 (0.000, 0.000)	0	0.000 (0.000, 0.000)
10–14 years old	1	0.000 (0.000, 0.059)	1	0.000 (0.000, 0.060)	0	0.000 (0.000, 0.000)	0	0.000 (0.000, 0.000)
15–19 years old	0	0.000 (0.000, 0.000)	0	0.000 (0.000, 0.000)	0	0.000 (0.000, 0.000)	0	0.000 (0.000, 0.000)
20–44 years old	2	0.005 (0.000, 0.019)	1	0.000 (0.000, 0.009)	1	0.000 (0.000, 0.009)	0	0.000 (0.000, 0.000)
45–64 years old	0	0.000 (0.000, 0.000)	0	0.000 (0.000, 0.000)	1	0.006 (0.000, 0.022)	0	0.000 (0.000, 0.000)
Over 65 years	1	0.015 (0.000, 0.059)	0	0.000 (0.000, 0.000)	0	0.000 (0.000, 0.000)	0	0.000 (0.000, 0.000)
Women	5	0.010 (0.000, 0.020)	4	0.006 (0.000, 0.013)	2	0.004 (0.000, 0.009)	1	0.000 (0.000, 0.003)
Adults	2	0.005 (0.000, 0.014)	0	0.000 (0.000, 0.000)	0	0.000 (0.000, 0.000)	0	0.000 (0.000, 0.000)
0–4 years old	2	0.044 (0.000, 0.167)	3	0.134 (0.000, 0.286)	2	0.089 (0.000, 0.213)	1	0.000 (0.000, 0.086)
5–9 years old	1	0.000 (0.000, 0.069)	0	0.000 (0.000, 0.000)	0	0.000 (0.000, 0.000)	0	0.000 (0.000, 0.000)
10–14 years old	0	0.000 (0.000, 0.000)	1	0.000 (0.000, 0.060)	0	0.000 (0.000, 0.000)	0	0.000 (0.000, 0.000)
15–19 years old	0	0.000 (0.000, 0.000)	0	0.000 (0.000, 0.000)	0	0.000 (0.000, 0.000)	0	0.000 (0.000, 0.000)
20–44 years old	1	0.000 (0.000, 0.009)	0	0.000 (0.000, 0.000)	0	0.000 (0.000, 0.000)	0	0.000 (0.000, 0.000)
45–64 years old	0	0.000 (0.000, 0.000)	0	0.000 (0.000, 0.000)	0	0.000 (0.000, 0.000)	0	0.000 (0.000, 0.000)
Over 65 years	1	0.015 (0.000, 0.059)	0	0.000 (0.000, 0.000)	0	0.000 (0.000, 0.000)	0	0.000 (0.000, 0.000)
Men	4	0.006 (0.000, 0.013)	4	0.006 (0.000, 0.014)	3	0.006 (0.000, 0.013)	2	0.002 (0.000, 0.007)
Adults	1	0.000 (0.000, 0.004)	1	0.000 (0.000, 0.004)	2	0.005 (0.000, 0.013)	0	0.000 (0.000, 0.000)
0–4 years old	2	0.044 (0.000, 0.167)	3	0.134 (0.000, 0.286)	1	0.044 (0.000, 0.132)	2	0.044 (0.000, 0.166)
5–9 years old	0	0.000 (0.000, 0.000)	0	0.000 (0.000, 0.000)	0	0.000 (0.000, 0.000)	0	0.000 (0.000, 0.000)
10–14 years old	1	0.000 (0.000, 0.059)	0	0.000 (0.000, 0.000)	0	0.000 (0.000, 0.000)	0	0.000 (0.000, 0.000)
15–19 years old	0	0.000 (0.000, 0.000)	0	0.000 (0.000, 0.000)	0	0.000 (0.000, 0.000)	0	0.000 (0.000, 0.000)
20–44 years old	1	0.000 (0.000, 0.009)	1	0.000 (0.000, 0.009)	1	0.000 (0.000, 0.009)	0	0.000 (0.000, 0.000)
45–64 years old	0	0.000 (0.000, 0.000)	0	0.000 (0.000, 0.000)	1	0.006 (0.000, 0.022)	0	0.000 (0.000, 0.000)
Over 65 years	0	0.000 (0.000, 0.000)	0	0.000 (0.000, 0.000)	0	0.000 (0.000, 0.000)	0	0.000 (0.000, 0.000)

Cont. Suppl. Table 2-16.

Variables	2011		2012		2013		2014		2015	
	n	Mortality (95% CI)	n	Mortality (95% CI)	n	Mortality (95% CI)	n	Mortality (95% CI)	n	Mortality (95% CI)
All	9	0.018 (0.006, 0.030)	4	0.006 (0.000, 0.013)	5	0.008 (0.000, 0.016)	4	0.005 (0.000, 0.013)	5	0.007 (0.000, 0.016)
Adults	3	0.007 (0.000, 0.017)	0	0.000 (0.000, 0.000)	0	0.000 (0.000, 0.000)	0	0.000 (0.000, 0.000)	0	0.000 (0.000, 0.000)
0–4 years old	6	0.223 (0.014, 0.432)	4	0.134 (0.000, 0.304)	4	0.134 (0.000, 0.306)	4	0.134 (0.000, 0.306)	5	0.178 (0.000, 0.373)
5–9 years old	0	0.000 (0.000, 0.000)	0	0.000 (0.000, 0.000)	0	0.000 (0.000, 0.000)	0	0.000 (0.000, 0.000)	0	0.000 (0.000, 0.000)
10–14 years old	0	0.000 (0.000, 0.000)	0	0.000 (0.000, 0.000)	1	0.000 (0.000, 0.072)	0	0.000 (0.000, 0.000)	0	0.000 (0.000, 0.000)
15–19 years old	0	0.000 (0.000, 0.000)	0	0.000 (0.000, 0.000)	0	0.000 (0.000, 0.000)	0	0.000 (0.000, 0.000)	0	0.000 (0.000, 0.000)
20–44 years old	1	0.000 (0.000, 0.010)	0	0.000 (0.000, 0.000)	0	0.000 (0.000, 0.000)	0	0.000 (0.000, 0.000)	0	0.000 (0.000, 0.000)
45–64 years old	1	0.006 (0.000, 0.021)	0	0.000 (0.000, 0.000)	0	0.000 (0.000, 0.000)	0	0.000 (0.000, 0.000)	0	0.000 (0.000, 0.000)
Over 65 years	1	0.015 (0.000, 0.053)	0	0.000 (0.000, 0.000)	0	0.000 (0.000, 0.000)	0	0.000 (0.000, 0.000)	0	0.000 (0.000, 0.000)
Women	6	0.012 (0.002, 0.022)	2	0.002 (0.000, 0.007)	2	0.002 (0.000, 0.007)	2	0.001 (0.000, 0.007)	2	0.001 (0.000, 0.007)
Adults	3	0.007 (0.000, 0.017)	0	0.000 (0.000, 0.000)	0	0.000 (0.000, 0.000)	0	0.000 (0.000, 0.000)	0	0.000 (0.000, 0.000)
0–4 years old	3	0.089 (0.000, 0.237)	2	0.044 (0.000, 0.165)	2	0.044 (0.000, 0.166)	2	0.044 (0.000, 0.166)	2	0.044 (0.000, 0.167)
5–9 years old	0	0.000 (0.000, 0.000)	0	0.000 (0.000, 0.000)	0	0.000 (0.000, 0.000)	0	0.000 (0.000, 0.000)	0	0.000 (0.000, 0.000)
10–14 years old	0	0.000 (0.000, 0.000)	0	0.000 (0.000, 0.000)	0	0.000 (0.000, 0.000)	0	0.000 (0.000, 0.000)	0	0.000 (0.000, 0.000)
15–19 years old	0	0.000 (0.000, 0.000)	0	0.000 (0.000, 0.000)	0	0.000 (0.000, 0.000)	0	0.000 (0.000, 0.000)	0	0.000 (0.000, 0.000)
20–44 years old	1	0.000 (0.000, 0.010)	0	0.000 (0.000, 0.000)	0	0.000 (0.000, 0.000)	0	0.000 (0.000, 0.000)	0	0.000 (0.000, 0.000)
45–64 years old	1	0.006 (0.000, 0.021)	0	0.000 (0.000, 0.000)	0	0.000 (0.000, 0.000)	0	0.000 (0.000, 0.000)	0	0.000 (0.000, 0.000)
Over 65 years	1	0.015 (0.000, 0.053)	0	0.000 (0.000, 0.000)	0	0.000 (0.000, 0.000)	0	0.000 (0.000, 0.000)	0	0.000 (0.000, 0.000)
Men	3	0.004 (0.000, 0.010)	2	0.002 (0.000, 0.007)	3	0.004 (0.000, 0.010)	2	0.001 (0.000, 0.007)	3	0.003 (0.000, 0.010)
Adults	0	0.000 (0.000, 0.000)	0	0.000 (0.000, 0.000)	0	0.000 (0.000, 0.000)	0	0.000 (0.000, 0.000)	0	0.000 (0.000, 0.000)
0–4 years old	3	0.089 (0.000, 0.237)	2	0.044 (0.000, 0.165)	2	0.044 (0.000, 0.166)	2	0.044 (0.000, 0.166)	3	0.089 (0.000, 0.240)
5–9 years old	0	0.000 (0.000, 0.000)	0	0.000 (0.000, 0.000)	0	0.000 (0.000, 0.000)	0	0.000 (0.000, 0.000)	0	0.000 (0.000, 0.000)
10–14 years old	0	0.000 (0.000, 0.000)	0	0.000 (0.000, 0.000)	1	0.000 (0.000, 0.072)	0	0.000 (0.000, 0.000)	0	0.000 (0.000, 0.000)
15–19 years old	0	0.000 (0.000, 0.000)	0	0.000 (0.000, 0.000)	0	0.000 (0.000, 0.000)	0	0.000 (0.000, 0.000)	0	0.000 (0.000, 0.000)
20–44 years old	0	0.000 (0.000, 0.000)	0	0.000 (0.000, 0.000)	0	0.000 (0.000, 0.000)	0	0.000 (0.000, 0.000)	0	0.000 (0.000, 0.000)
45–64 years old	0	0.000 (0.000, 0.000)	0	0.000 (0.000, 0.000)	0	0.000 (0.000, 0.000)	0	0.000 (0.000, 0.000)	0	0.000 (0.000, 0.000)
Over 65 years	0	0.000 (0.000, 0.000)	0	0.000 (0.000, 0.000)	0	0.000 (0.000, 0.000)	0	0.000 (0.000, 0.000)	0	0.000 (0.000, 0.000)

a Age-standardized mortality rates of double outlet right ventricle were calculated using age groups according to the direct method using the estimated Korean population in 2015 as a reference.

Supplementary Table 2-17. Age-standardized mortality[a] and 95% confidence interval (CI) of single ventricle overall and by sex (per 100,000).

Variables	2007		2008		2009		2010	
	n	Mortality (95% CI)	n	Mortality (95% CI)	n	Mortality (95% CI)	n	Mortality (95% CI)
All	6	0.010 (0.006, 0.020)	3	0.006 (0.000, 0.013)	9	0.016 (0.004, 0.028)	8	0.014 (0.003, 0.025)
Adults	1	0.000 (0.000, 0.004)	0	0.000 (0.000, 0.000)	1	0.000 (0.000, 0.004)	1	0.000 (0.000, 0.004)
0–4 years old	4	0.134 (0.000, 0.308)	3	0.134 (0.000, 0.286)	7	0.313 (0.080, 0.545)	6	0.223 (0.012, 0.435)
5–9 years old	0	0.000 (0.000, 0.000)	0	0.000 (0.000, 0.000)	0	0.000 (0.000, 0.000)	0	0.000 (0.000, 0.000)
10–14 years old	0	0.000 (0.000, 0.000)	0	0.000 (0.000, 0.000)	0	0.000 (0.000, 0.000)	0	0.000 (0.000, 0.000)
15–19 years old	1	0.000 (0.000, 0.094)	0	0.000 (0.000, 0.000)	1	0.000 (0.000, 0.059)	1	0.000 (0.000, 0.058)
20–44 years old	1	0.000 (0.000, 0.009)	0	0.000 (0.000, 0.000)	1	0.000 (0.000, 0.009)	1	0.000 (0.000, 0.009)
45–64 years old	0	0.000 (0.000, 0.000)	0	0.000 (0.000, 0.000)	0	0.000 (0.000, 0.000)	0	0.000 (0.000, 0.000)
Over 65 years	0	0.000 (0.000, 0.000)	0	0.000 (0.000, 0.000)	0	0.000 (0.000, 0.000)	0	0.000 (0.000, 0.000)
Women	3	0.004 (0.000, 0.011)	2	0.004 (0.000, 0.009)	3	0.006 (0.000, 0.013)	3	0.004 (0.000, 0.010)
Adults	0	0.000 (0.000, 0.000)	0	0.000 (0.000, 0.000)	0	0.000 (0.000, 0.000)	0	0.000 (0.000, 0.000)
0–4 years old	2	0.044 (0.000, 0.167)	2	0.089 (0.000, 0.213)	3	0.134 (0.000, 0.286)	3	0.089 (0.000, 0.239)
5–9 years old	0	0.000 (0.000, 0.000)	0	0.000 (0.000, 0.000)	0	0.000 (0.000, 0.000)	0	0.000 (0.000, 0.000)
10–14 years old	0	0.000 (0.000, 0.000)	0	0.000 (0.000, 0.000)	0	0.000 (0.000, 0.000)	0	0.000 (0.000, 0.000)
15–19 years old	1	0.031 (0.000, 0.094)	0	0.000 (0.000, 0.000)	0	0.000 (0.000, 0.000)	0	0.000 (0.000, 0.000)
20–44 years old	0	0.000 (0.000, 0.000)	0	0.000 (0.000, 0.000)	0	0.000 (0.000, 0.000)	0	0.000 (0.000, 0.000)
45–64 years old	0	0.000 (0.000, 0.000)	0	0.000 (0.000, 0.000)	0	0.000 (0.000, 0.000)	0	0.000 (0.000, 0.000)
Over 65 years	0	0.000 (0.000, 0.000)	0	0.000 (0.000, 0.000)	0	0.000 (0.000, 0.000)	0	0.000 (0.000, 0.000)
Men	3	0.004 (0.000, 0.011)	1	0.002 (0.000, 0.006)	6	0.010 (0.000, 0.019)	5	0.008 (0.000, 0.016)
Adults	1	0.000 (0.000, 0.004)	0	0.000 (0.000, 0.000)	1	0.000 (0.000, 0.004)	1	0.000 (0.000, 0.004)
0–4 years old	2	0.044 (0.000, 0.167)	1	0.044 (0.000, 0.132)	4	0.178 (0.003, 0.354)	3	0.089 (0.000, 0.239)
5–9 years old	0	0.000 (0.000, 0.000)	0	0.000 (0.000, 0.000)	0	0.000 (0.000, 0.000)	0	0.000 (0.000, 0.000)
10–14 years old	0	0.000 (0.000, 0.000)	0	0.000 (0.000, 0.000)	0	0.000 (0.000, 0.000)	0	0.000 (0.000, 0.000)
15–19 years old	0	0.000 (0.000, 0.000)	0	0.000 (0.000, 0.000)	1	0.000 (0.000, 0.059)	1	0.000 (0.000, 0.058)
20–44 years old	1	0.000 (0.000, 0.009)	0	0.000 (0.000, 0.000)	1	0.000 (0.000, 0.000)	1	0.000 (0.000, 0.009)
45–64 years old	0	0.000 (0.000, 0.000)	0	0.000 (0.000, 0.000)	0	0.000 (0.000, 0.000)	0	0.000 (0.000, 0.000)
Over 65 years	0	0.000 (0.000, 0.000)	0	0.000 (0.000, 0.000)	0	0.000 (0.000, 0.000)	0	0.000 (0.000, 0.000)

Cont. Suppl. Table 2-17.

Variables	2011		2012		2013		2014		2015	
	n	Mortality (95% CI)	n	Mortality (95% CI)	n	Mortality (95% CI)	n	Mortality (95% CI)	n	Mortality (95% CI)
All	5	0.008 (0.000, 0.016)	6	0.010 (0.000, 0.019)	4	0.006 (0.000, 0.013)	4	0.005 (0.000, 0.013)	5	0.007 (0.000, 0.016)
Adults	0	0.000 (0.000, 0.000)	0	0.000 (0.000, 0.000)	0	0.000 (0.000, 0.000)	0	0.000 (0.000, 0.000)	0	0.000 (0.000, 0.000)
0–4 years old	5	0.178 (0.000, 0.369)	6	0.223 (0.014, 0.432)	4	0.134 (0.000, 0.306)	4	0.134 (0.000, 0.306)	5	0.178 (0.000, 0.373)
5–9 years old	0	0.000 (0.000, 0.000)	0	0.000 (0.000, 0.000)	0	0.000 (0.000, 0.000)	0	0.000 (0.000, 0.000)	0	0.000 (0.000, 0.000)
10–14 years old	0	0.000 (0.000, 0.000)	0	0.000 (0.000, 0.000)	0	0.000 (0.000, 0.000)	0	0.000 (0.000, 0.000)	0	0.000 (0.000, 0.000)
15–19 years old	0	0.000 (0.000, 0.000)	0	0.000 (0.000, 0.000)	0	0.000 (0.000, 0.000)	0	0.000 (0.000, 0.000)	0	0.000 (0.000, 0.000)
20–44 years old	0	0.000 (0.000, 0.000)	0	0.000 (0.000, 0.000)	0	0.000 (0.000, 0.000)	0	0.000 (0.000, 0.000)	0	0.000 (0.000, 0.000)
45–64 years old	0	0.000 (0.000, 0.000)	0	0.000 (0.000, 0.000)	0	0.000 (0.000, 0.000)	0	0.000 (0.000, 0.000)	0	0.000 (0.000, 0.000)
Over 65 years	0	0.000 (0.000, 0.000)	0	0.000 (0.000, 0.000)	0	0.000 (0.000, 0.000)	0	0.000 (0.000, 0.000)	0	0.000 (0.000, 0.000)
Women	2	0.002 (0.000, 0.007)	2	0.002 (0.000, 0.007)	3	0.004 (0.000, 0.010)	1	0.000 (0.000, 0.003)	3	0.003 (0.000, 0.010)
Adults	0	0.000 (0.000, 0.000)	0	0.000 (0.000, 0.000)	0	0.000 (0.000, 0.000)	0	0.000 (0.000, 0.000)	0	0.000 (0.000, 0.000)
0–4 years old	2	0.044 (0.000, 0.165)	2	0.044 (0.000, 0.165)	3	0.089 (0.000, 0.238)	1	0.000 (0.000, 0.086)	3	0.089 (0.000, 0.240)
5–9 years old	0	0.000 (0.000, 0.000)	0	0.000 (0.000, 0.000)	0	0.000 (0.000, 0.000)	0	0.000 (0.000, 0.000)	0	0.000 (0.000, 0.000)
10–14 years old	0	0.000 (0.000, 0.000)	0	0.000 (0.000, 0.000)	0	0.000 (0.000, 0.000)	0	0.000 (0.000, 0.000)	0	0.000 (0.000, 0.000)
15–19 years old	0	0.000 (0.000, 0.000)	0	0.000 (0.000, 0.000)	0	0.000 (0.000, 0.000)	0	0.000 (0.000, 0.000)	0	0.000 (0.000, 0.000)
20–44 years old	0	0.000 (0.000, 0.000)	0	0.000 (0.000, 0.000)	0	0.000 (0.000, 0.000)	0	0.000 (0.000, 0.000)	0	0.000 (0.000, 0.000)
45–64 years old	0	0.000 (0.000, 0.000)	0	0.000 (0.000, 0.000)	0	0.000 (0.000, 0.000)	0	0.000 (0.000, 0.000)	0	0.000 (0.000, 0.000)
Over 65 years	0	0.000 (0.000, 0.000)	0	0.000 (0.000, 0.000)	0	0.000 (0.000, 0.000)	0	0.000 (0.000, 0.000)	0	0.000 (0.000, 0.000)
Men	3	0.004 (0.000, 0.010)	4	0.006 (0.000, 0.013)	1	0.000 (0.000, 0.003)	3	0.003 (0.000, 0.010)	2	0.001 (0.000, 0.007)
Adults	0	0.000 (0.000, 0.000)	0	0.000 (0.000, 0.000)	0	0.000 (0.000, 0.000)	0	0.000 (0.000, 0.000)	0	0.000 (0.000, 0.000)
0–4 years old	3	0.089 (0.000, 0.237)	4	0.134 (0.000, 0.304)	1	0.000 (0.000, 0.086)	3	0.089 (0.000, 0.238)	2	0.044 (0.000, 0.167)
5–9 years old	0	0.000 (0.000, 0.000)	0	0.000 (0.000, 0.000)	0	0.000 (0.000, 0.000)	0	0.000 (0.000, 0.000)	0	0.000 (0.000, 0.000)
10–14 years old	0	0.000 (0.000, 0.000)	0	0.000 (0.000, 0.000)	0	0.000 (0.000, 0.000)	0	0.000 (0.000, 0.000)	0	0.000 (0.000, 0.000)
15–19 years old	0	0.000 (0.000, 0.000)	0	0.000 (0.000, 0.000)	0	0.000 (0.000, 0.000)	0	0.000 (0.000, 0.000)	0	0.000 (0.000, 0.000)
20–44 years old	0	0.000 (0.000, 0.000)	0	0.000 (0.000, 0.000)	0	0.000 (0.000, 0.000)	0	0.000 (0.000, 0.000)	0	0.000 (0.000, 0.000)
45–64 years old	0	0.000 (0.000, 0.000)	0	0.000 (0.000, 0.000)	0	0.000 (0.000, 0.000)	0	0.000 (0.000, 0.000)	0	0.000 (0.000, 0.000)
Over 65 years	0	0.000 (0.000, 0.000)	0	0.000 (0.000, 0.000)	0	0.000 (0.000, 0.000)	0	0.000 (0.000, 0.000)	0	0.000 (0.000, 0.000)

a Age-standardized mortality rates of single ventricle were calculated using age groups according to the direct method using the estimated Korean population in 2015 as a reference.

Supplementary Table 3. Five-year survival rates and 95% confidence interval of congenital heart disease in Korean adults

Variables	Overall	Age group			Sex	
		20-44 years old	45-64 years old	Over 65 years old	Male	Female
CHD	0.91 (0.90, 0.92)	0.98 (0.97, 0.99)	0.95 (0.94, 0.95)	0.73 (0.72, 0.74)	0.91 (0.90, 0.92)	0.92 (0.91, 0.93)
VSD and/or ASD	0.93 (0.92, 0.94)	0.98 (0.97, 0.99)	0.95 (0.94, 0.96)	0.75 (0.74, 0.77)	0.93 (0.92, 0.94)	0.94 (0.93, 0.95)
VSD	0.94 (0.93, 0.95)	0.98 (0.74, 0.99)	0.95 (0.93, 0.96)	0.70 (0.64, 0.75)	0.94 (0.93, 0.95)	0.94 (0.93, 0.95)
ASD	0.93 (0.92, 0.94)	0.98 (0.97, 0.99)	0.96 (0.95, 0.97)	0.78 (0.75, 0.80)	0.92 (0.91, 0.93)	0.93 (0.92, 0.94)
PDA	0.95 (0.94, 0.96)	0.97 (0.96, 0.98)	0.95 (0.94, 0.97)	0.79 (0.72, 0.84)	0.94 (0.91, 0.96)	0.95 (0.94, 0.96)
PAS	0.92 (0.89, 0.92)	0.97 (0.94, 0.98)	0.91 (0.85, 0.94)	0.67 (0.57, 0.75)	0.90 (0.85, 0.93)	0.90 (0.86, 0.93)
CoA	0.91 (0.87, 0.93)	0.97 (0.93, 0.98)	0.89 (0.79, 0.94)	0.76 (0.63, 0.85)	0.91 (0.86, 0.94)	0.90 (0.85, 0.94)
AS	0.75 (0.71, 0.79)	0.95 (0.87, 0.98)	0.91 (0.84, 0.95)	0.60 (0.53, 0.66)	0.79 (0.73, 0.84)	0.71 (0.65, 0.77)
AR	0.93 (0.92, 0.94)	0.99 (0.98, 0.99)	0.94 (0.92, 0.95)	0.83 (0.80, 0.86)	0.92 (0.91, 0.94)	0.93 (0.91, 0.95)
MS	0.85 (0.81, 0.89)	0.97 (0.93, 0.99)	0.90 (0.81, 0.94)	0.63 (0.51, 0.72)	0.85 (0.78, 0.90)	0.86 (0.80, 0.90)
Malformation of coronary vessels	0.94 (0.93, 0.94)	0.98 (0.97, 0.99)	0.97 (0.96, 0.97)	0.84 (0.82, 0.86)	0.94 (0.93, 0.94)	0.94 (0.93, 0.95)
Stenosis or malformation of aorta	0.79 (0.77, 0.80)	0.96 (0.94, 0.98)	0.93 (0.91, 0.95)	0.62 (0.60, 0.65)	0.80 (0.77, 0.82)	0.78 (0.76, 0.80)
TOF	0.96 (0.95, 0.97)	0.97 (0.96, 0.98)	0.94 (0.89, 0.96)	0.65 (0.37, 0.82)	0.95 (0.93, 0.97)	0.97 (0.95, .084)
Ebstein anomaly	0.92 (0.89, 0.94)	0.97 (0.93, 0.98)	0.93 (0.88, 0.96)	0.77 (0.65, 0.85)	0.90 (0.85, 0.94)	0.93 (0.89, 0.95)
TGV	0.85 (0.80, 0.89)	0.97 (0.92, 0.99)	0.87 (0.76, 0.93)	0.59 (0.43, 0.71)	0.82 (0.72, 0.88)	0.89 (0.82, 0.93)
Eisenmenger syndrome	0.61 (0.58, 0.63)	0.84 (0.80, 0.87)	0.69 (0.64, 0.73)	0.46 (0.42, 0.79)	0.56 (0.52, 0.60)	0.64 (0.61, 0.67)

CHD congenital heart disease; VSD and/or ASD ventricular septal defect and/or atrial septal defect; PDA patent ductus arteriosus; PAS pulmonary artery stenosis; CoA Coarctation of aorta; AS congenital stenosis of aortic valve; AR congenital insufficiency of aortic valve; MS congenital mitral stenosis; TOF Tetralogy of Fallot; TGV Transposition of great vessels.

Supplementary Table 4. Distribution of echocardiography devices from 2005 through 2012 according to the National Health Insurance Service of Korea

Medical institution classification	Year							
	2005	2006	2007	2008	2009	2010	2011	2012
Total	1,445	1,542	2,002	2,103	2,239	2,342	1,286	21,301
Tertiary hospital	132	143	212	221	241	257	323	1,881
General hospital	264	270	345	359	375	405	391	2,661
Hospital	210	238	296	336	347	359	207	3,066
Clinic	837	890	1,146	1,183	1,272	1,316	363	13,578
Dental hospital	0	0	0	0	0	0	0	3
Dental clinic	0	0	0	0	0	0	0	1
Midwife	0	0	0	0	0	0	0	5
Health medical center	0	0	0	1	1	1	0	26
Health center	1	1	1	2	1	1	1	55
Health center branch	0	0	0	0	0	0	0	1
Community health care services	0	0	0	0	0	0	0	0
Oriental medicine hospital	0	0	0	0	0	1	1	24
Oriental medicine clinic	1	0	2	2	2	2	0	0
